# Metagenomic and metabolomic analyses unveil dysbiosis of gut microbiota in chronic heart failure patients

**DOI:** 10.1038/s41598-017-18756-2

**Published:** 2018-01-12

**Authors:** Xiao Cui, Lei Ye, Jing Li, Ling Jin, Wenjie Wang, Shuangyue Li, Minghui Bao, Shouling Wu, Lifeng Li, Bin Geng, Xin Zhou, Jian Zhang, Jun Cai

**Affiliations:** 1 Fuwai Hospital, State Key Laboratory of Cardiovascular Diseases, National Center for Cardiovascular Diseases, Chinese Academy of Medical Sciences and Peking Union Medical College, Beijing, 100037 P.R. China; 2 Novogene Bioinformatics Institute, Beijing, 100000 P.R. China; 3 Department of Cardiology, Beijing Chao Yang Hospital, Capital Medical University, Beijing, 100020 China; 4 Department of Cardiology, Kailuan General Hospital, Hebei Union University, Tangshan, 063000 P.R. China; 5 Tianjin Key Laboratory of Cardiovascular Remodeling and Target Organ Injury, Pingjin Hospital Heart Center, Tianjin, 300162 P.R. China

## Abstract

Previous studies suggested a possible gut microbiota dysbiosis in chronic heart failure (CHF). However, direct evidence was lacking. In this study, we investigated the composition and metabolic patterns of gut microbiota in CHF patients to provide direct evidence and comprehensive understanding of gut microbiota dysbiosis in CHF. We enrolled 53 CHF patients and 41 controls. Metagenomic analyses of faecal samples and metabolomic analyses of faecal and plasma samples were then performed. We found that the composition of gut microbiota in CHF was significantly different from controls. *Faecalibacterium prausnitzii* decrease and *Ruminococcus gnavus* increase were the essential characteristics in CHF patients’ gut microbiota. We also observed an imbalance of gut microbes involved in the metabolism of protective metabolites such as butyrate and harmful metabolites such as trimethylamine N-oxide in CHF patients. Metabolic features of both faecal and plasma samples from CHF patients also significantly changed. Moreover, alterations in faecal and plasma metabolic patterns correlated with gut microbiota dysbiosis in CHF. Taken together, we found that CHF was associated with distinct gut microbiota dysbiosis and pinpointed the specific core bacteria imbalance in CHF, along with correlations between changes in certain metabolites and gut microbes.

## Introduction

Chronic heart failure (CHF) is an end-stage syndrome of many cardiovascular diseases, associated with structural and/or functional abnormalities of heart, leading to insufficient blood perfusion to meet the body’s requirements^1^. About 23 million people suffer from heart failure worldwide, giving rise to heavy global health and economic burdens^2,3^. The causation of CHF varies, including ischaemic and non-ischaemic ones, without agreed single classification based on the etiology. Previous studies have suggested important impacts of inflammation and immune dysfunction on the pathogenesis of heart failure^4^. Gut’s roles in CHF have also been discussed for years, especially the involvement in chronic inflammation and malnutrition in CHF^5^. Besides of the classic recognition of the intestinal hypoperfusion, barrier dysfunction and bacteria translocation, whether there existed a dysbiosis of gut microbiota in CHF was unclear^6,7^.

According to the updated estimation, the number of microbes that inhabiting in and on our human body was similar to that of human cells, among which gut microbes account for a large proportion^8^. Recently, emerging evidences have suggested gut microbiota disequilibrium could greatly influence hosts’ pathophysiologic states through various mechanisms such as immune and metabolic alterations^9,10^. Our previous study highlighted that gut microbiota dysbiosis contributed to the development of hypertension^11^. A recent study showed that dietary intervention could prevent the development hypertension and heart failure in hypertensive mice through changing their gut microbiota^12^. Whether gut microbiota dysbiosis could contribute to CHF through triggering systematic inflammation as well as producing trimethylamine N-oxide (TMAO) and some other uremic toxins has naturally drawn researchers’ attention^13^. Studies have shown that levels of TMAO, a gut microbes-dependent metabolite, elevated and showed predictive value for poor prognosis in studies on both chronic and acute heart failure patients^14^. Using culturing method, researchers found more pathogenic bacteria from CHF patients’ faecal samples and associated with chronic inflammation in CHF^15^. Consistently, a nationwide analysis in the United States showed higher rates of *Clostridium difficile* infection in heart failure patients and associated with markedly higher in-hospital mortality^16^. However, changes in microbial metabolites could only be indirect evidence for gut microbiota dysbiosis. Meanwhile, about 80% of gut microbes could not be cultured yet^17^. In view of this, direct evidence for gut microbiota dysbiosis in CHF patients was still lacking.

In the present study, we performed metagenomic analyses of faecal samples from CHF patients, in combination with faecal and plasma metabolomic analyses, to provide direct evidence and comprehensive understanding of gut microbiota dysbiosis in CHF.

## Results

### Clinical characteristics of subjects

We consecutively recruited 53 CHF (ischaemic cardiomyopathy, ICM, n = 29; dilated cardiomyopathy, DCM, n = 24) patients and 41 individuals as controls. Clinical characteristics of all subjects were shown in Table 1. The majority of CHF patients were with poor cardiac function that 51% of them were in the New York Heart Association functional classification (NYHA) III; 43% in NYHA IV, 6% in NYHA II and none in NYHA I. There was no significant difference between CHF patients and controls in body mass index, blood pressure, history of smoking and history of alcohol drinking. None of the subjects had inflammatory bowel diseases, irritable bowel syndrome, autoimmune diseases, liver diseases, renal diseases or cancer, but more CHF patients were comorbid with hypertension, hyperlipidaemia and diabetes compared with controls. Serum levels of white blood cells, C-reactive protein and creatinine were higher in CHF patients. None of the subjects used antibiotics or probiotics in the last 1 month, but CHF patients were taking more medications including angiotensin converting enzyme inhibitor or angiotensin receptor blocker, β receptor blocker, digoxin, diuretics, statins, aspirin and proton pump inhibitors (PPIs) compared with controls.

**Table 1:** Demographic and clinical characteristics of all subjects.

	CHF	Control	p value
Age (year)	58.08 ± 13.30	53.73 ± 5.94	0.06
Gender (%)			0.73
Male	83	78	
Female	17	22	
BMI (kg/m^2^)	24.42 ± 4.53	25.24 ± 3.32	0.34
**Blood pressure (mmHg)**			
Systolic blood pressure	114 (98, 130)	117 (110, 120)	0.93
Diastolic blood pressure	76 (69, 84)	75 (70, 80)	0.52
Heart rate (bpm)	77 (68, 87)	70 (63, 77)	0.01
**NYHA (%)**			
I	0	—	—
II	6	—	—
III	51	—	—
IV	43	—	—
LVEF %	29.79 ± 6.54	—	—
**Comorbidities (%)**			
Inflammatory bowel diseases	0	0	—
Irritable bowel syndrome	0	0	—
Autoimmune diseases	0	0	—
Liver diseases	0	0	—
Renal diseases	0	0	—
Cancer	0	0	—
Hypertension	57	0	<0.01
Hyperlipidaemia	51	7	<0.01
Diabetes	28	4	<0.01
Smoking (%)	57	54	0.24
Alcohol drinking (%)	42	29	0.20
**Medication (%)**			
ACEI/ARB	60	0	<0.01
β receptor blocker	81	0	<0.01
digoxin	66	0	<0.01
diuretics	91	0	<0.01
statins	64	0	<0.01
aspirin	57	4	<0.01
PPIs	42	0	<0.01
WBC*10^9^	7.02 (6.12, 8.65)	5.78 (5.15, 7.10)	<0.01
PLT*10^9^	196.08 ± 65.92	242.68 ± 57.32	<0.01
CRP (mg/L)	3.36 (2.30, 8.49)	2.00 (1.00, 3.00)	<0.01
CREA (mmol/L)	93.04 (77.41, 108.92)	71.00 (64.25, 90.50)	<0.01
BUN	7.92 (6.24, 10.15)	5.50 (4.80, 6.60)	<0.01
ALT (U/L)	20 (15, 33)	19 (12, 26)	0.15
Na^+^ (mmol/L)	139.60 (137.57, 141.05)	140.00 (139.00, 142.98)	0.06
K^+^ (mmol/L)	4.13 ± 0.38	4.21 ± 0.38	0.36
FBG (mmol/L)	5.59 (4.90, 7.26)	5.20 (4.81, 5.58)	0.04
TG (mmol/L)	1.18 (0.83, 1.67)	1.05 (0.89, 1.72)	0.91
CHOL (mmol/L)	3.68 (3.10, 4.19)	5.32 (4.67, 6.19)	<0.01
HDL (mmol/L)	0.93 ± 0.31	1.25 ± 0.25	<0.01
LDL (mmol/L)	2.26 (1.68, 2.87)	2.72 (2.30, 2.96)	0.02

Values are expressed as mean ± standard deviation, median (first quartile, third quartile), or %. CHF = chronic heart failure; BMI = body mass index; NYHA = the New York Heart Association functional classification; LVEF = left ventricular ejection fraction; ACEI = angiotensin converting enzyme inhibitor; ARB = angiotensin receptor blocker; PPIs = proton pump inhibitors; WBC = white blood cell; PLT = platelet; CRP = C-reactive protein; CREA = serum creatinine; BUN = blood urea nitrogen; ALT = alanine aminotransferase; FBG = fasting blood glucose; TG = triglycerides; CHOL = cholesterol; HDL = high density lipoprotein; LDL = low density lipoprotein.

### Compositional alteration of gut microbiota in CHF

**Figure 1:**
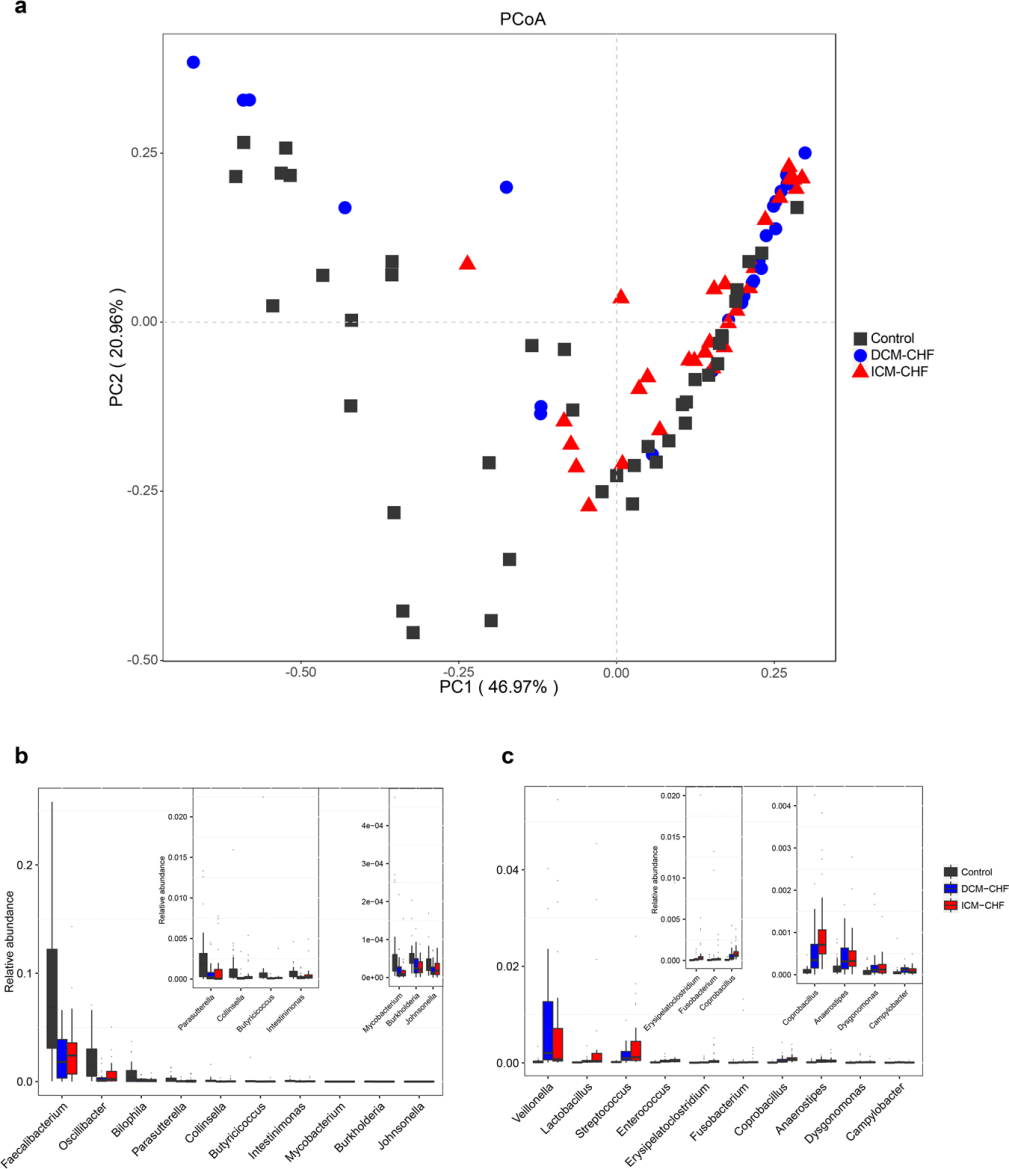
Compositional and structural shifts of gut microbiota in CHF patients. **(a)** Principal coordinates analysis of beta-diversity analysis based on Bray Curtis distances of 86 genera differentially enriched across controls, DCM- and ICM-induced CHF patients. The  represents control. The  represents DCM-induced CHF. The  represents ICM-induced CHF. (**b**,**c**) Boxplot of top ten genera differentially enriched in CHF patients (b) and controls (b). Black, controls; blue, DCM-induced CHF patients; red, ICM- induced CHF patients.

**Figure 2:**
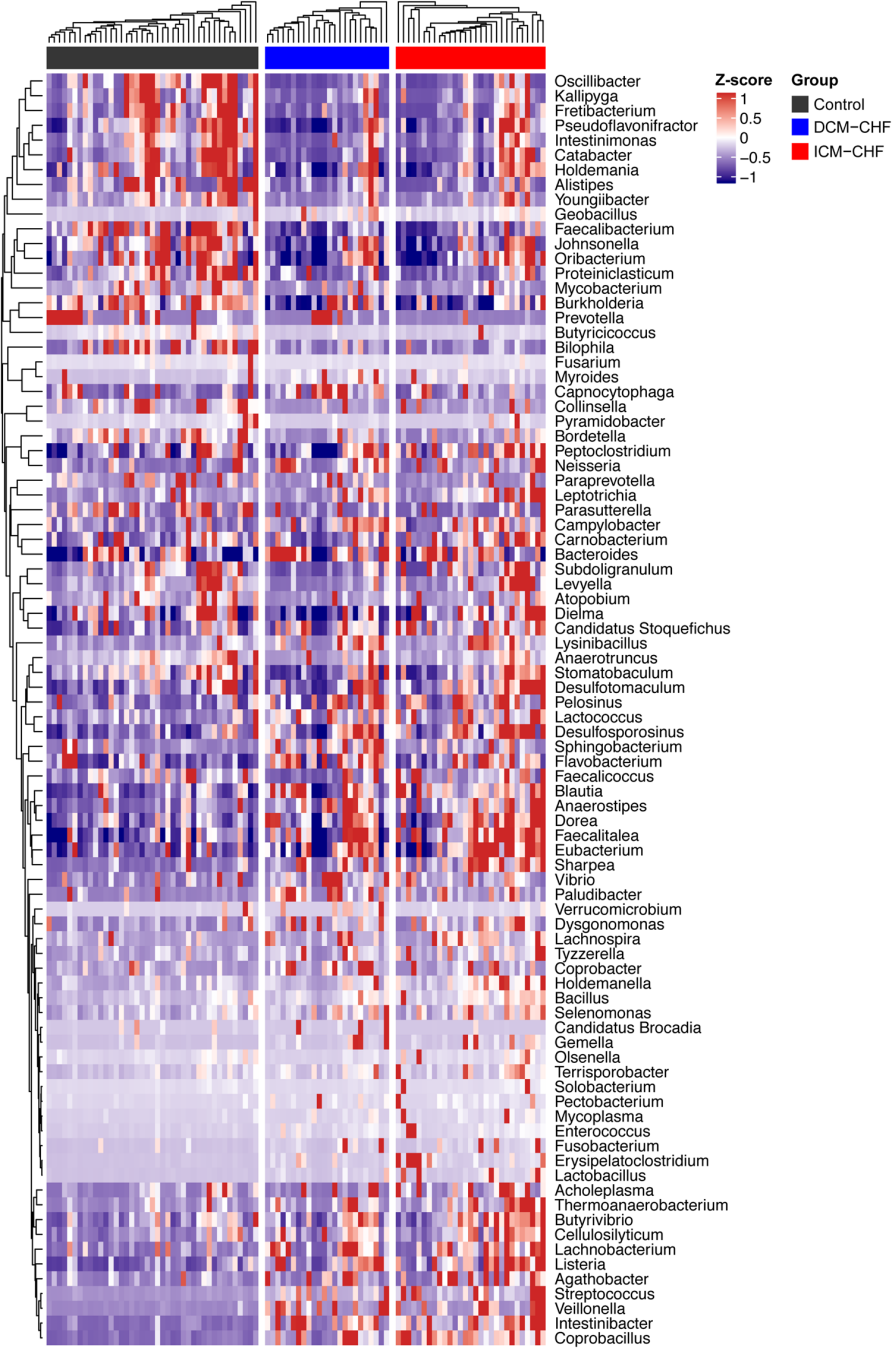
Gut bacteria differentially enriched across DCM-induced CHF, ICM-induced CHF and controls at genus level. Heatmap of genera differentially enriched across controls, DCM- and ICM-induced CHF patients. The abundance profiles were transformed into Z scores. Black, enriched in controls; blue, enriched in DCM-induced CHF patients; red, enriched in ICM- induced CHF patients.

According to beta-diversity analysis based on Bray Curtis distances of 86 genera differentially enriched across groups, the structures of gut microbiota in CHF were significantly different from controls (F = 5.66, p = 0.003, R^2^ = 0.0580), but quite similar between DCM- and ICM-induced CHF (F = 2.01, p = 0.101, R^2^ = 0.0380, Fig. 1a). Principal component analysis (PCA) based on abundances of the microbes showed consistent results (Supplementary Fig. S1). The levels of C-reactive protein and creatinine were positively correlated with CHF-enriched gut microbes, while high density lipoprotein positively correlated with control-enriched gut microbes (Supplementary Fig. S2). The top ten gut bacteria differentially enriched between CHF patients and controls at genus level were displayed in Fig. 1b and c. Some genera such as *Ruminococcus*, *Acinetobacter* and *Veillonella* increased (Fig. 1b), whereas some (such as *Alistipes*, *Faecalibacterium* and *Oscillibacter*) decreased in CHF (Fig. 1c). 86 genera were differentially enriched between CHF patients and controls. Among them, there were 54 genera differentially enriched between DCM-induced CHF and controls, and 61 genera between ICM-induced CHF and controls. However, there were only eight genera differentially enriched between DCM- and ICM-induced CHF, which were all of relatively low abundances (Supplementary Fig. S3). Gut bacteria differentially enriched across DCM-induced CHF, ICM-induced CHF and controls at genus level were shown in Fig. 2. These results suggested that CHF exhibited similar changes in the gut microbiota composition, no matter whether the causation was DCM or ICM. Considering the concern about the possible effects of PPIs and statins usage on the gut microbiota, we further analysed the possible influences of PPIs and statins on the gut microbiota by performing PERMANOVA on the beta diversity of gut microbiota across controls, CHF patients with PPIs and CHF patients without PPIs, and that across controls, CHF patients with statins and CHF patients without statins^18,19^. The results showed that the difference in the beta diversity based on Bray Curtis distances of gut microbiota remained significant between CHF and controls, no matter whether the CHF subjects used or did not use PPIs or statins, while no significant different between CHF subjects with and without PPIs or statins (CHF with PPI vs. control: F = 2.46, p = 0.047, R^2^ = 0.0387; CHF without PPI vs. control, F = 5.91, p = 0.001, R^2^ = 0.0779; CHF with PPIs vs. CHF without PPIs, F = 0.64, p = 0.648, R^2^ = 0.0124; CHF with statins vs. control, F = 4.62, p = 0.005, R^2^ = 0.0595; CHF without statins vs. control, F = 3.77, p = 0.012, R^2^ = 0.0611; CHF with statins vs. CHF without statins, F = 0.49, p = 0.765, R^2^ = 0.0095, Supplementary Fig. S4).

Here, we identified 481,946 different genes in relative abundance among groups. According to their abundance variations, we clustered these genes and obtained 439 distinct co-abundance groups (CAGs). In consistence with the differences in genera composition, there were 312 CAGs differentially enriched between DCM-induced CHF and controls, and 320 CAGs between ICM-induced CHF and controls, while only 64 CAGs between DCM- and ICM-induced CHF.

### Core gut bacterium species in CHF

**Figure 3:**
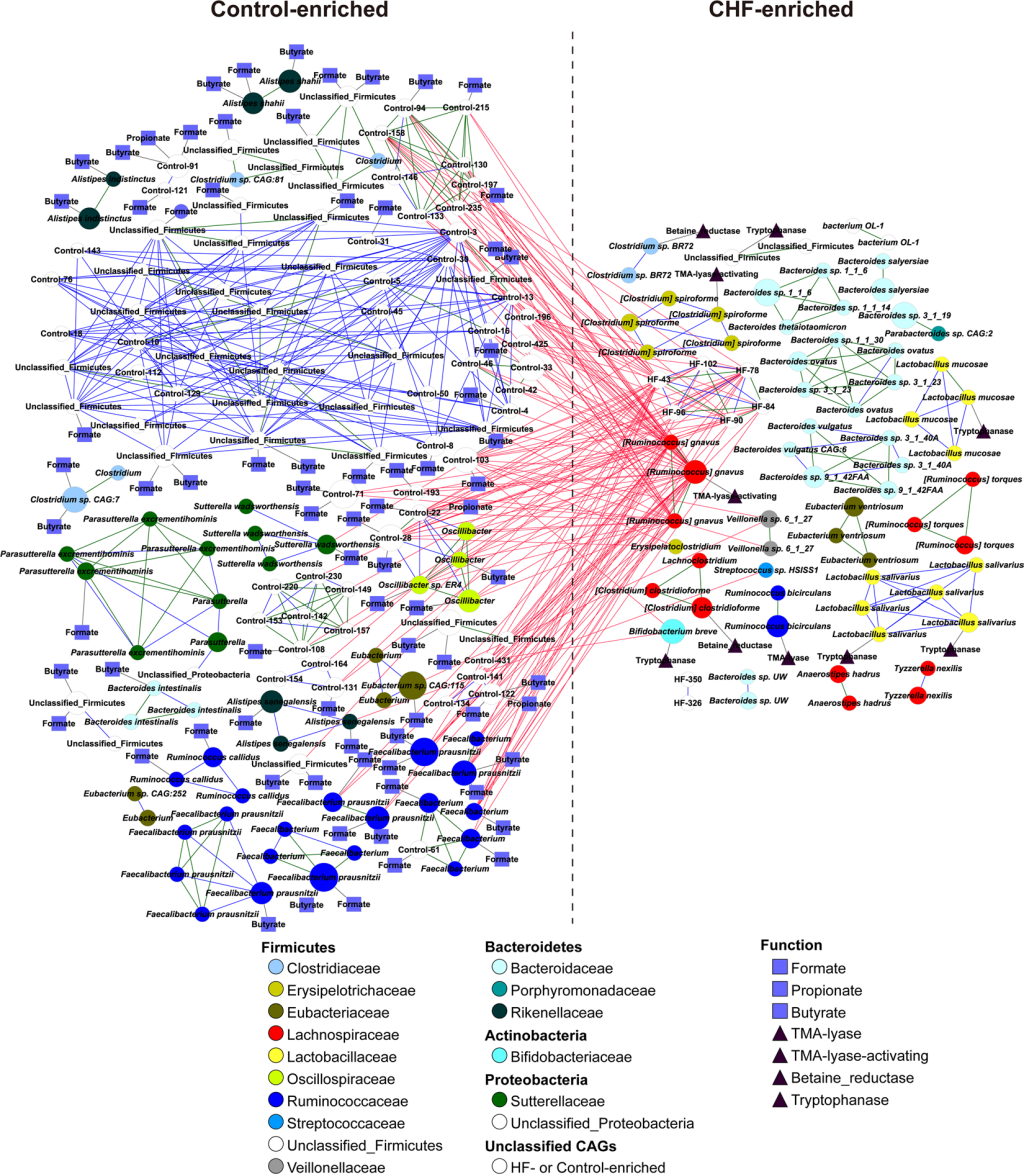
CAGs differentially enriched between CHF patients and controls. The direction of enrichment was determined by Wilcoxon rank sum test (p < 0.05). Sizes of the nodes were in proportion with each CAGs’ gene numbers. CAGs within the same family were in the same colour. Edges between nodes represented Spearman’s correlation >0.9 (green), between 0.8 and 0.9 (blue) or <−0.55 (red). The  represented the presence of the genes encoding choline TMA-lyase, choline TMA-lyase-activating enzyme, betaine reductase or tryptophanase. The  represented the presence of the genes encoding butyrate-acetoacetate CoA transferase, propionate CoA-transferase or formate-tetrahydrofolate ligase.

Based on the Spearman’s correlation, we built a co-occurrence network of marker CAGs and performed taxonomic assignments of them (Fig. 3). Spearman’s correlation coefficients, p values and q values as correction of p values for multiple testing were shown in Supplementary Table S1. The network showed the differential enrichments of bacteria such as *Ruminococcus gnavus*, *Streptococcus sp*. and *Veillonella sp* in CHF patients, by contrast, *Faecalibacterium prausnitzii*, *Oscillibacter sp*. and *Sutterella wadsworthensis* in controls. The core node of the network in CHF was *R*. *gnavus*. The abundances of bacteria enriched in controls were inversely correlated with that of *R*. *gnavus*. However, in controls, *F*. *prausnitzii* was the core bacterium, whose abundance inversely correlated with CHF-enriched gut bacteria. This CAG enrichment network analysis suggested that *F*. *prausnitzii* decrease and *R*. *gnavus* increase were the essential characteristics in the gut microbiota of CHF patients.

### Functional alteration of gut microbiota in CHF

**Figure 4:**
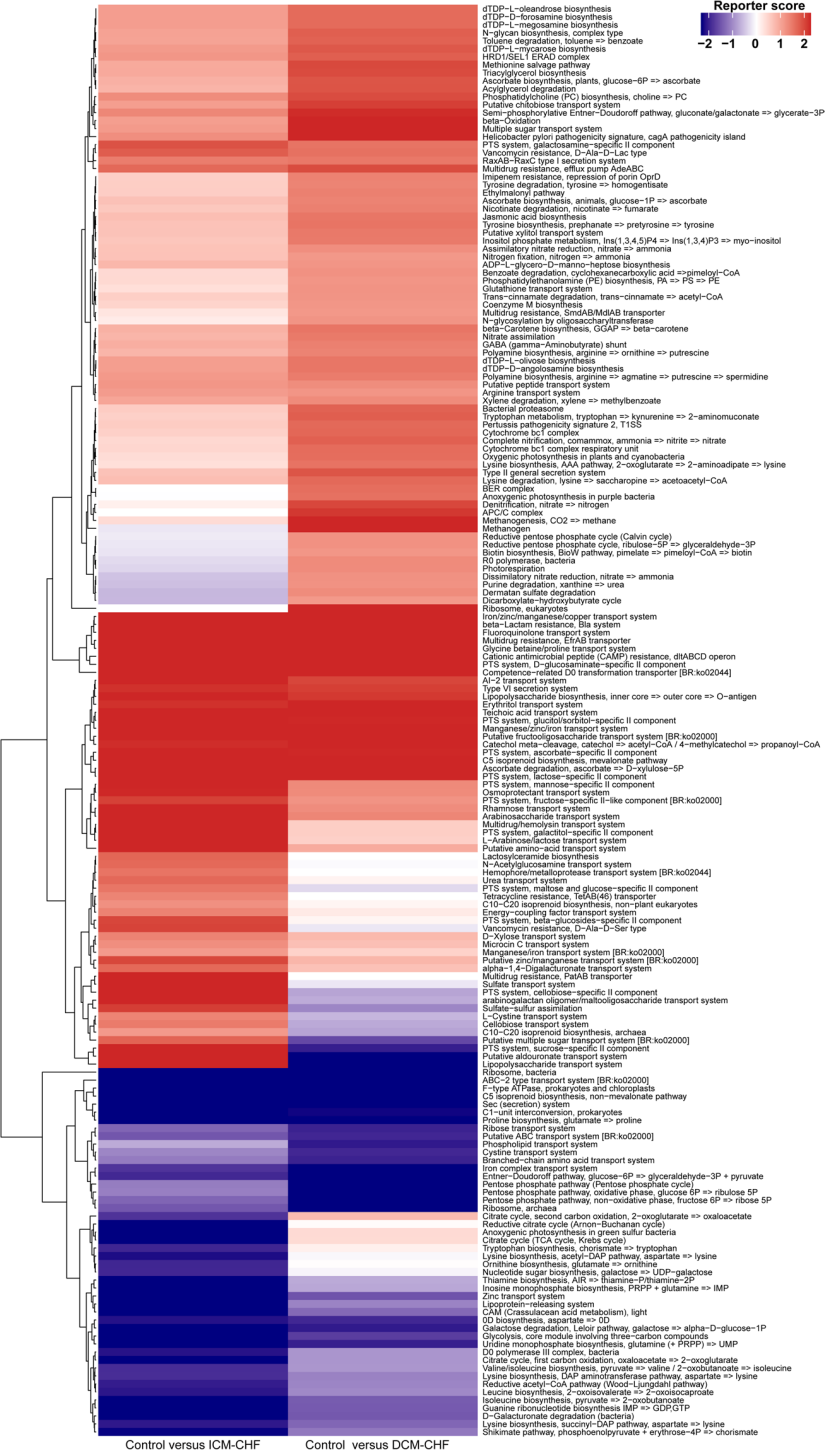
Overview of functional shifts of the gut microbiota in CHF patients. Heatmap and hierarchical clustering of KO modules enriched across controls, DCM- and ICM- induced CHF patients. Modules differentially enriched across groups were identified on the basis of the reporter score derived from each KO’s Z-score. Blue, enriched in controls; red, enriched in CHF patients.

To characterize the distinct functions of the gut microbiota, we performed functional annotations of the metagenome to KEGG modules. As Fig. 4 shown, microbial genes for phosphotransferase systems increased in CHF patients’ gut microbiota. In contrast, those for synthesizing and transporting amino acids significantly reduced in the disordered microbiota of CHF patients. Genes for nucleotide sugar biosynthesis and iron transport system also reduced in CHF patients compared with controls (Fig. 4).

**Figure 5:**
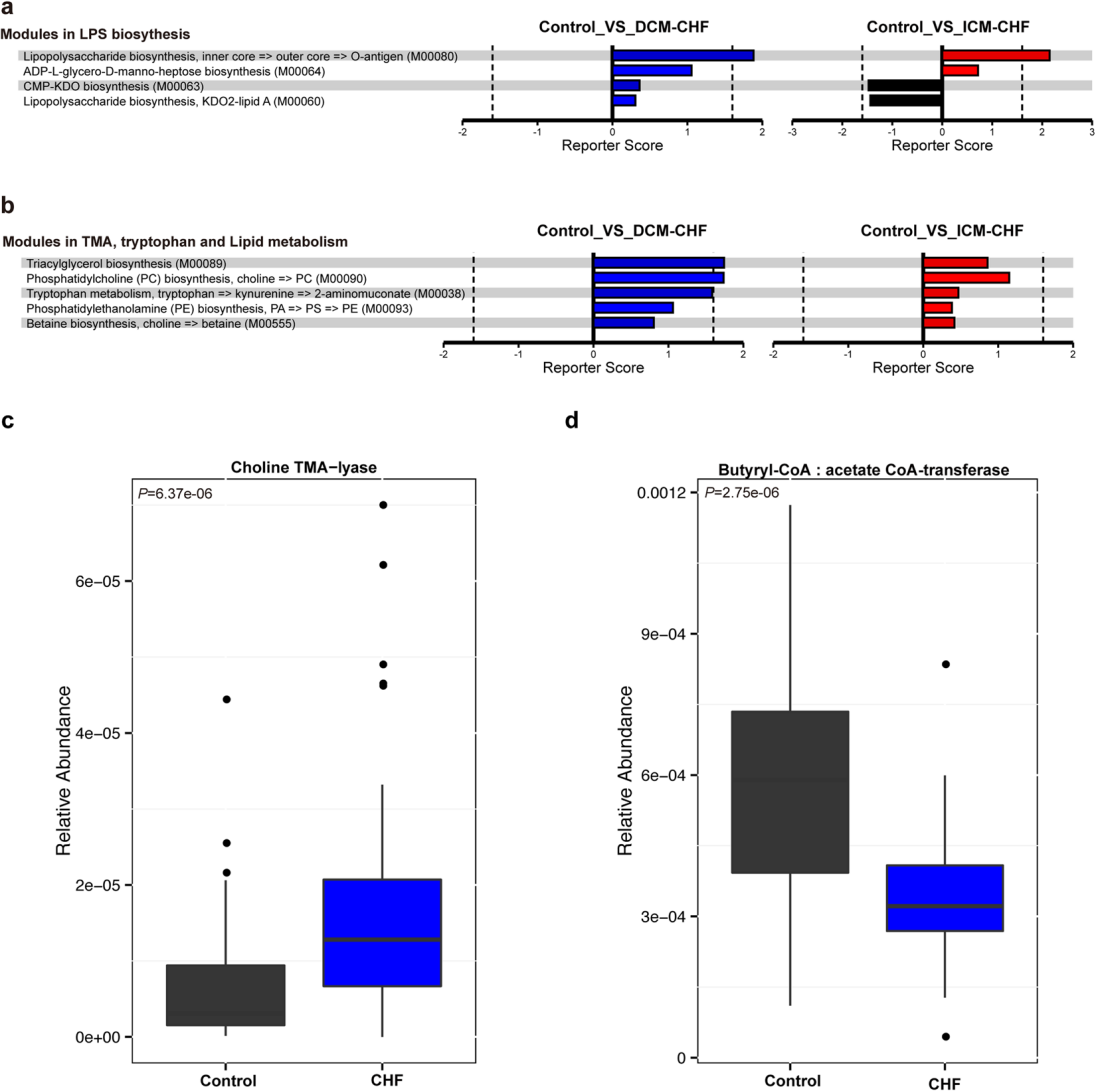
Important functional shifts of the gut microbiota in CHF patients. (**a**,**b**) Modules in lipopolysaccharide biosynthesis (**a**), TMA, tryptophan and lipid metabolism (**b**). Black, enriched in controls; blue, enriched in DCM-induced CHF patients; red, enriched in ICM- induced CHF patients. **(c**,**d)** Group level abundance shifts of choline TMA-lyase (**c**), butyrate-acetoacetate CoA transferase (**d**) between CHF patients and controls by Wilcoxon rank test. Black, enriched in controls; blue, enriched in CHF patients.

More interestingly, we observed an elevation in microbial genes for lipopolysaccharide biosynthesis, tryptophan, lipid metabolism, especially TMAO generation in CHF patients’ gut microbiota (Fig. 5a,b). Microbial genes for choline TMA-lyase, the key enzyme for the generation of TMAO which is a cardiac harmful metabolite, significantly upregulated in CHF patients (Fig. 5c). Meanwhile, microbial genes for butyrate-acetoacetate CoA transferase, the key enzyme for the generation of butyrate which is a protective metabolic fatty acid, significantly reduced in CHF patients’ gut microbiota(Fig. 5d). To attain a more comprehensive view of the CAG enrichment network described above, we further performed functional annotations of those CAGs. Intriguingly, bacteria involved in short chain fatty acid metabolism such as formate, propionate and butyrate producing were reduced in CHF compared with controls (Fig. 3). These functional shifts of microbial metagenome indicated a correlation between CHF and an imbalance of gut microbes involved in the metabolism of protective metabolites such as butyrate and harmful metabolites such as TMAO.

### Faecal and plasma metabolomics in CHF

Considering the distinct metabolic functions between CHF and controls based on functional annotations of microbial metagenome, we further performed metabolic profiling of faecal and plasma samples from subjects by using high-throughput liquid chromatography-mass spectrometry (LC/MS). The faecal and plasma samples were respectively subjected to LC/MS analysis in both positive ion mode (ES+) and negative ion mode (ES−). After eliminating the impurity peaks and duplicate identifications, we identified a total of 10184 chromatographic peaks for faecal samples and a total of 4103 chromatographic peaks for plasma samples for further analyses. The standard deviations of peak area of faecal and plasma metabolites in CHF, controls and quality controls in both ES+ and ES− were shown in Supplementary Tables S2–S5, respectively. The combination of variable importance in the projection (VIP) value from orthogonal partial least-squares discriminant analysis (OPLS-DA) model >1 and p value < 0.05 based on the peak areas were used to identify differentially enriched compounds. The m/z value of these compounds was then used to identify the metabolites corresponding to the featured peak in Metlin database. The identification confidence of the data to certain levels in both ES+ and ES− were also shown in Supplementary Tables S2–S5, respectively^20^.

**Figure 6:**
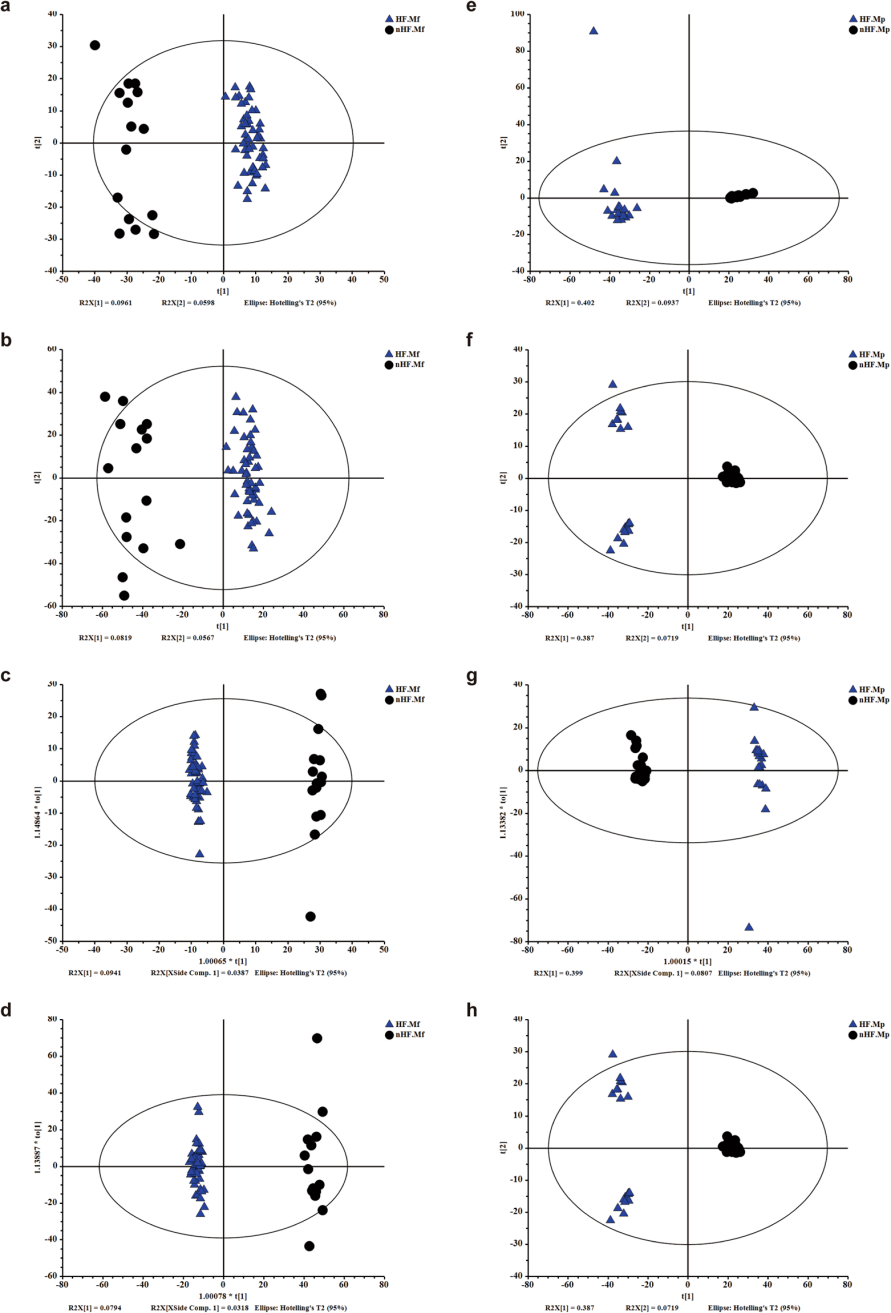
Metabolomic analyses of faecal and plasma samples of CHF patients and controls. (**a**,**b**) The PCA scores plot based on faecal metabolic profiles in ES+ (**a**) and ES− (**b**). **(c**,**d)** The OPLS-DA scores plot based on faecal metabolic profiles in ES+ (**c**) and ES− (**d**). **(e**,**f)** The PCA scores plot based on plasma metabolic profiles in ES+ (**e**) and ES− (**f**). **(g**,**h)** The OPLS-DA scores plot based on plasma metabolic profiles in ES+ (**g**) and ES− (**h**). The  represents metabolic profiles of CHF patients. The  represents metabolic profiles of controls. ES+ = positive ion mode; ES− = negative ion mode.

PCA of faecal metabolites showed significant difference between CHF and controls (Fig. 6a,b). To discriminate the metabolic profiles between groups, we performed partial least-squares discriminant analysis (PLS-DA) of the data (Supplementary Fig. S5). The validation of this model showed no overfitting phenomenon, which represented that this model could well describe the samples and could be applied in further data analysis (Supplementary Fig. S5). We further performed OPLS-DA (Fig. 6c,d). We identified that 25 differentially enriched metabolites in ES+ and 192 differentially enriched metabolites in ES− from faecal samples between CHF and controls, among which 9 metabolites were identified in both the ES+ and ES−. Among them, 2 metabolites including para-Tolyl octanoate significantly increased in CHF patients, while other 206 metabolites such as niacin, cinnamic acid and orotic acid significantly decreased in CHF compared with controls.

Plasma metabolites were also significantly different between CHF and controls according to PCA (Fig. 6e,f). Then, we also performed PLS-DA to discriminate the metabolic profiles between groups (Supplementary Fig. S5). The validation of this model showed no overfitting phenomenon, representing that this model could well describe the samples and could be applied in further data analysis (Supplementary Fig. S5). OPLS-DA was then performed (Fig. 6g,h). Finally, we identified 45 differentially enriched metabolites in ES+ and 69 differentially enriched metabolites in ES− from plasma samples between CHF and controls, among which 6 metabolites were identified in both the ES+ and ES−. Among them, 49 metabolites such as sphingosine 1−phosphate significantly increased, while other 59 metabolites such as ricinoleic acid significantly decreased in CHF compared with controls.

### Metabolites changes correlated with microbial genera

**Figure 7:**
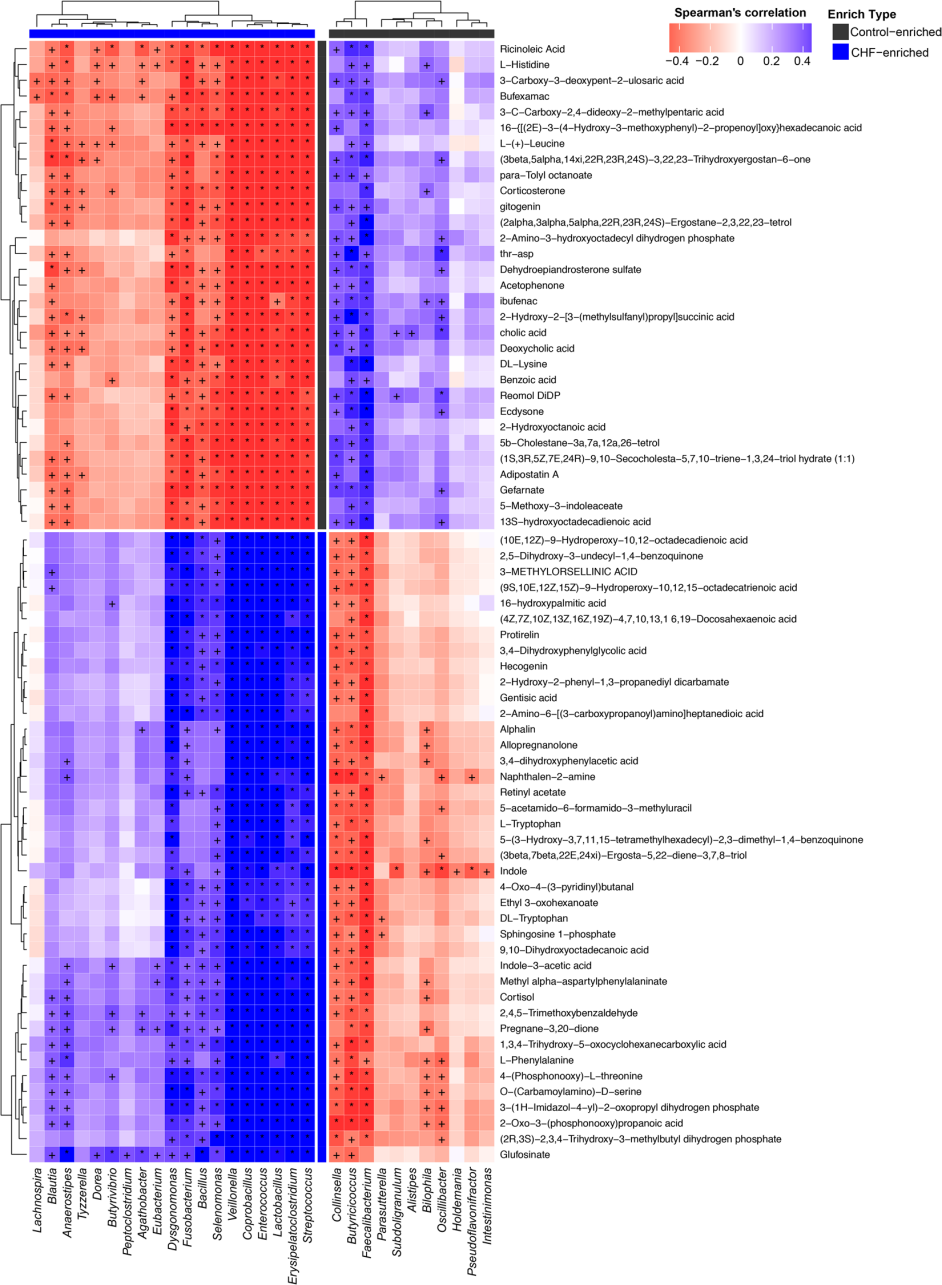
Correlations between plasma metabolic patterns and genera. Spearman’s correlation coefficients between the abundance of top 35 differentially enriched genera and the level of plasmatic metabolic patterns were calculated. Those with low correlation (|r| < 0.6) were not shown. Red, negative correlation; blue, positive correlation, ^+^q < 0.05, *q < 0.01. The enriched type of each genera and metabolic patterns was coloured according to its direction of enrichment. Black, enriched in controls; blue, enriched in CHF patients.

Next, we analysed possible correlations between altered faecal and plasma metabolites and microbial genera based on Spearman’s correlation. Spearman’s correlation coefficients, p values and q values as the correction of p values for multiple testing were shown in Supplementary Tables S6 and S7, respectively. Faecal decreased metabolites such as niacin, cinnamic acid and orotic acid were negatively correlated with CHF-enriched bacteria *Veillonella*, but positively correlated with controls-enriched bacteria such as *Faecalibacterium*, *Butyricicoccus* and *Oscillibacter* (Supplementary Fig. S6). Moreover, high plasma sphingosine 1-phosphate was positively correlated with several CHF-enriched bacteria such as *Veillonella*, *Coprobacillus* and *Streptococcus*, while plasma reduced metabolites such as ricinoleic acid were positively correlated with bacteria enriched in controls such as *Butyricicoccus* (Fig. 7).

## Discussion

In this study, we provided the evidence for gut microbiota dysbiosis in CHF patients. The composition of gut microbiota in CHF was significantly different from controls, while quite similar between CHF subgroups of different causations. Meaningfully, we found that *F*. *prausnitzii* decrease and *R*. *gnavus* increase were the essential characteristics in CHF patients’ gut microbiota. By functional analyses of microbial metagenome, we observed an imbalance of gut microbes involved in the metabolism of protective metabolites such as butyrate and harmful metabolites such as TMAO in CHF patients, which might contribute to the pathogenesis of CHF. Metabolic features of both faecal and plasma samples from CHF patients also significantly changed. Moreover, alterations in faecal and plasma metabolic patterns correlated with gut microbiota dysbiosis in CHF. These findings suggested an aberrant gut microbiota in CHF patients.

Our findings extended the current knowledge of gut’s roles in CHF. A previous metabolomic research involving 720 patients with stable heart failure observed higher TMAO levels in heart failure patients and higher plasma TMAO levels were associated with a 3.4-fold increased mortality risk^14^. In consistence with that, through functional annotations of metagenome, we found that microbial genes for choline TMA-lyase, the key enzyme for the generation of TMAO, significantly upregulated in CHF patients. That TMAO could directly lead to progressive renal tubulointerstitial fibrosis and dysfunction might be one of the underlying mechanisms in aggravating CHF progression^21^. Endotoxemia-induced systematic inflammation is known to be involved in the pathophysiology of heart failure^13^. In consistence with that, we observed an enrichment of gut microbial genes involved in lipopolysaccharide biosynthesis in CHF patients. A previous study showed that more pathogenic bacteria such as *Campylobacter*, *Shigella* and *Salmonella* could be cultured from the faeces of CHF patients^15^. Considering that one important function of healthy gut microbiota is to protect intestine against the colonization of pathogenic bacteria, this could be reasonably explained by gut microbiota dysbiosis increasing the risks of pathogenic infection^22^.

More importantly, we found that *F*. *prausnitzii* decrease and *R*. *gnavus* increase were the essential characteristics in the gut microbiota of CHF patients. *F. prausnitzii*, one of the most abundant butyrate-producing species, has been identified as an important anti-inflammatory commensal bacterium^23,24^. Loss or lack of *F. prausnitzii* and its significant functions in anti-inflammation might aggravate the chronic inflammation. Previous studies showed that elderly population had lower levels of *F*. *prausnitzii* than young adults^25,26^. A recent published study found that *F. prausnitzii* was less abundant in older heart failure patients than in younger patients^27^. This could correlate with further aggravating inflammatory status and poor prognosis in elderly CHF patients considering that inflammation is independently accompanied with adverse outcome in elderly CHF patients^28^. Moreover, through functional annotations of microbial metagenome, we observed decreases of other butyrate-producing bacteria and butyryl-CoA: acetate CoA-transferase genes in CHF compared with controls. A reduction of butyrate-producing bacteria could be regarded as a biomarker for injured gut microbiota and supplement of them as probiotics showed therapeutic benefits in several diseases^29–32^. Butyryl-CoA: acetate CoA-transferase is crucial in the synthesis of butyrate^23^. Butyrate is of great importance in anti-inflammation and maintaining intestinal barrier integrity. It could modulate intestinal macrophages’ function, downregulating lipopolysaccharide-induced pro-inflammatory mediators, such as nitric oxide, IL-6 and IL-12^33^. It could also induce the differentiation of regulatory T cells, which suppress both inflammatory responses and heart failure progression^34,35^. Furthermore, butyrate can stabilize hypoxia-inducible factor, which plays a fundamental role in maintaining barrier integrity^36^. Considering these, the reduction in butyrate production might participate to the chronic inflammation aggravation in CHF. At the same time, we found that *R*. *gnavus* significantly increased in CHF patients. Previous studies on inflammatory bowel diseases have indicated a pro-inflammatory property of *R. gnavus*^37–39^. Implantation of *R. gnavus* to gnotobiotic mice increased the levels of IFN-γ, IL-17 and IL-22^38^. *R. gnavus* lysates can preferentially stimulate bacterial antigen-specific Th1 and Th17 cell-mediated immune responses^40^. Based on the above, we provide not only the direct evidence of dysbiosis of gut microbiota in CHF, but also a comprehensive understanding of the associations between gut microbiota dysbiosis and certain function alterations.

Combining metabolomic data, we found that CHF-enriched bacteria such as *Veillonella* were inversely correlated with cardiovascular protective metabolites such as niacin, cinnamic acid and orotic acid^41–45^. Dietary supplement of orotic acid could boost both the fatty acid oxidation and the uptake of glucose, giving rise to the energy supply and appreciable alterations on myocardial contractile function^45^. Sphingosine 1-phosphate has been identified as a mediator in multiple pathological processes in cardiovascular system, including activating pro-inflammatory responses in the cardiomyocyte and causing cardiac dysfunction and remodeling^46^. In consistence with this, we observed a significant elevation of sphingosine 1-phosphate in the plasma samples of CHF patients. Furthermore, we discovered a positive correlation between the high sphingosine 1-phosphate levels and several CHF-enriched bacteria such as *Veillonella*, *Coprobacillus* and *Streptococcus*. Meanwhile, plasma levels of metabolites such as ricinoleic acid, which owns an anti-inflammatory property, significantly reduced in CHF patients. Moreover, levels of ricinoleic acid inversely correlated with gut bacterium enriched in CHF patients, while positively correlated with those enriched in non-CHF populations^47^. Based on the above, we observed an increase of cardiovascular harmful metabolites such as sphingosine 1-phosphate and a decrease of cardiovascular protective metabolites such as orotic acid in CHF patients, and correlations between theses metabolites and some gut microbes.

We acknowledge some limitations of this study. First, there was representative limitation. CHF patients included in this study were in-hospital patients from large hospitals who were admitted to hospitals because of unstable illness status, that the majority of them had poor cardiac function (51% in NYHA III, 43% in NYHA IV, 6% in NYHA II and none in NYHA I). So, we could only prudently draw conclusions on associations between severe CHF and gut dysbiosis. Second, there were several confounding factors. CHF are frequently accompanied by comorbidities, such as hypertension, hyperlipidaemia and diabetes^48^. Although we excluded subjects with inflammatory bowel diseases, irritable bowel syndrome, autoimmune diseases, liver diseases, renal diseases or cancer, we did not exclude CHF patients comorbid with hyperlipidaemia or diabetes, considering the pathogenesis of CHF as the end stage of many metabolic and cardiovascular diseases. Although we excluded subjects who used antibiotics or probiotics in the last 1 month, subjects who took medicine for CHF were not excluded for ethical reasons. Exercise and dietary information were not collected and corrected in this study, either. These could result in confounding effects, in view of that hyperlipidaemia, diabetes, exercise, diet and other medication usage could also have influences on gut microbiota^12,18,19,49–51^. Previous studies have reported associations between PPIs, statins and gut microbiota^18,19^. A previous population-based metagenomics analysis study revealed the associations between the microbiome and 126 factors including PPIs and statins^18^. However, in another study suggesting a decrease in the Shannon’s diversity of gut microbiota, the researchers also reported that the decrease in species richness and Shannon diversity was not statistically significant in each cohort (Cohort 1, general population, p = 0.85; Cohort 2, patients with IBD, p = 0.16; Cohort 3, IBS case-control study, p = 0.53) after correcting confounders that related to certain bowel diseases and symptoms^19^. Considering the considerable numbers of PPIs and statins users among the CHF subjects in our present study, we also explored the possible influences of PPIs and statins on gut microbiota. The results showed that the changes in the beta diversity of gut microbiota in CHF subjects remained significant compared with controls, no matter whether the CHF subjects used or did not use PPIs or statins, while no significant difference between CHF subjects with and without PPIs or statins, suggesting that the significant difference in the beta diversity of gut microbiota between CHF patients and controls was not due to the effects of PPIs and statins. Finally, conclusions that could be drawn from our data were associations rather than causal relationships. Further study to validate the causal relationship between gut microbiota and CHF are still needed.

Taken together, we found that CHF was associated with distinct gut microbiota dysbiosis and pinpointed the specific core bacteria imbalance in CHF, along with correlations between changes in certain metabolites and gut microbes. These findings provided the direct evidence to validate the hypothesis of gut microbiota dysbiosis in CHF and a comprehensive understanding of the correlation between CHF and gut microbiota dysbiosis, which is fundamental for further investigations on the interaction between gut microbiota and CHF. Follow-up studies are needed to examine the causal relationship, to further investigate the specific mechanisms involved and to explore relevant intervention strategies.

## Methods

### Study cohort

53 chronic heart failure patients (CHF; ischaemic cardiomyopathy, ICM, n = 29; dilated cardiomyopathy, DCM, n = 24) were consecutively enrolled from patients who were admitted to Fuwai Hospital (n = 37), Chaoyang Hospital (n = 9) and Pingjin Hospital (n = 7). CHF patients met all the inclusion criteria, including age >18 years old, medical history of CHF either from ICM or DCM for more than 6 months, the New York Heart Association functional classification II to IV and left ventricular ejection fraction $\le$40%. Individuals with history of acute coronary syndrome in the last 6 months, comorbidities (inflammatory bowel diseases, irritable bowel syndrome, autoimmune diseases, liver diseases, renal diseases or cancer), or use of antibiotics or probiotics in the last 1 month were excluded. DCM was defined as ventricular dilatation with left ventricular systolic dysfunction not caused by hypertension, valve disease or coronary artery disease^52^. ICM was defined as left ventricular systolic dysfunction caused by myocardial infarction or significant coronary stenosis (≥75% stenosis of left main or proximal left anterior descending artery, or ≥75% stenosis of two or more epicardial vessels)^53^. Demographic and clinical characteristics were obtained through face-to-face survey and checking hospital records or medical examination records. This study was conducted in North China among Han population who shared similar diet patterns. Among the 53 CHF patients included, faecal samples were available from each subjects and used for metagenomic analyses and faecal metabolomic analyses. 20 of them kindly provided their plasma samples, which were used for plasma metabolomic analyses. Controls were all enrolled from Kailuan cohort who received biennial medical examination in Kailuan General Hospital^54^. The metagenomic sequencing data of 41 faecal samples, faecal metabolomic data of 15 samples and plasma metabolomic data of 30 samples were available from our previous study and used as controls in the present study^11^. The investigation conforms with the principles outlined in the Declaration of Helsinki. The research protocol was approved by the ethics committee of Fuwai Hospital, Chaoyang Hospital, Pingjin Hospital and Kailuan General Hospital. Written informed consents were obtained from all subjects.

### Sample collection and DNA extraction

Faecal samples freshly collected from each subject were immediately frozen at −20 °C, and transported to the laboratory with ice pack. Bacterial DNA was extracted at Novogene Bioinformatics Technology Co., Ltd using TIANGEN kits according to the manufacturer’s recommendations.

### Metagenomic sequencing and gene catalogue construction

All samples were paired-end sequenced on the Illumina platform (insert size 350 bp, read length 150 bp) at Novogene Bioinformatics Technology Co., Ltd. After quality control, reads that aligned to the human genome (alignment with SOAP2^55^, Version 2.21, parameters: -s 135, -l 30, -v 7, -m 200, -x 400) were also removed. The set of high-quality reads was then used for further analysis.

The assembly of reads was executed by SOAPdenovo2^56^ (Version 2.04, parameters: -d 1 -M 3 -R -u -F). For each sample, we used series of k-mer values (from 49 to 87), and chose optimal one with the longest N50 value for the remaining scaffolds^57^. We aligned clean reads to scaffolds using SOAP2 (Version 2.21, parameters: -m 200 -x 400 -s 135). Unused reads from each sample were assembled with the same parameters. Genes (minimum length of 100 nucleotides) were predicted on scaftigs (i.e., continuous sequences within scaffolds) longer than 500 bp using MetaGeneMark^58^ (prokaryotic GeneMark.hmm version 2.10). A non-redundant gene catalogue was then constructed with CD-HIT^59^ (version 4.5.8, parameters: -G 0 -aS 0.9 -g 1 -d 0 -c 0.95) using a sequence identity cut-off of 0.95, with a minimum coverage cut-off of 0.9 for the shorter sequences.

To assess the abundance of genes, reads were realigned to the gene catalogue with SOAP2 using parameters: -m 200 -x 400 -s 142. Only genes with $\ge$2 mapped reads were determined to be present in a sample to eliminate the incorrectly identification^60^. The abundance of a gene was calculated by counting the number of reads that aligned to the gene and normalized by the gene length.

### Taxonomic annotation and abundance profiling

To access the taxonomic assignments of genes, genes were aligned to the integrated NR database using DIAMOND^61^ (Version 0.7.9.58, default parameter except for -k 50–sensitive -e 0.00001). As previously described^60^, for each gene, the significant matches which were defined by e-values ≤ 10 * e-value of the top hit were retained to distinguish taxonomic groups. The taxonomical level of each gene was determined by the lowest common ancestor-based algorithm that was implemented in MEGAN^62^. The abundance of a taxonomic group was calculated by summing the abundance of genes annotated to a feature.

### Co-abundance gene groups (CAGs)

To identify marker genes that are associated with disease, genes that showed significant difference in relative abundance between any of the two groups were identified (Benjamin–Hochberg q-value < 0.05, Wilcoxon rank sum test). As previously described^63^, marker genes were then clustered according to their abundance variation across all samples. Clusters with more than 50 genes were called CAGs and used for further analysis. CAG abundance profiles were calculated as the average gene depth signal weighted by gene length.

Taxonomic assignments of the CAGs were performed according to the taxonomy of their tracer genes, as previously described^49^. Briefly, if more than 90% genes in the CAG were assigned to the species’ genome with more than 95% identity and 70% overlap of query, these CAGs were assigned as species. Smilarly, assigning an CAG to a genus requires more than 80% of its genes to align with a genome with more than 85% identity in both DNA and protein sequences.

### Co-occurrence network of marker CAGs

The marker CAGs were identified with wilcoxon rank sum test(Benjamin–Hochberg q-value < 0.05) between any of the two groups. Marker CAGs were then clustered in all samples according to Spearman’s correlation index. The co-occurrence network was plotted using Cytoscape (Version 3.2.1).

### Functional Annotation

All genes in our catalogue were aligned to the KEGG database (Release 73.1, with genes of plants and animals excluded) by DIAMOND (Version 0.7.9.58, default parameter except for -k 50 -sensitive -e 0.00001). Each protein was assigned to the KEGG orthology (KO) by the highest scoring annotated hits containing at least one HSP scoring over 60 bits^64^. Differentially enriched modules between groups were identified as previously described, according to their reporter score from the Z-scores of individual KOs^65^.

The protein sequences of Butyrate-acetoacetate-CoA transferase, choline TMA-lyase-activating enzyme, choline TMA-lyase, betaine reductase and tryptophanase were downloaded from NCBI database. The non-redundant gene catalogue was aligned to these sequences by using BLASTP (best-hit with E-value < 1E-5, identity >40% and coverage >50%)^66,67^.

### Metabolomic analyses based on LC/MS

50 mg faecal samples were transferred into Centrifuge Tubes(1.5 mL) by pipette. All faecal samples were extracted and precipitated protein with 800 μL of methanol, and 10 μL of internal standard (2.9 mg/mL, DL-o-Chlorophenylalanine) was added. The samples were grinded at 65 KHz for 90 s, and centrifuged at 12000 rpm and 4 °C for 15 min. 200 μL of supernatant was transferred to vial for anaylsis. The plasma metabolic profiles were performed on LC/MS platform (Thermo, Ultimate 3000LC, Orbitrap Elite) using Hypergod C18 (100 × 4.6 mm 3 μm) column. For chromatographic separation conditions, the column temperature was 40 °C; flow rate, 0.3 mL/min; mobile phase A, water +0.1% formic acid; mobile phase B, acetonitrile +0.1% formic acid; injection volume, 4 ml; automatic injector temperature, 4 °C.

The plasma samples were thawed at room temperature, 100 μL of them was then transferred into Centrifuge Tubes(1.5 mL) by pipette. All samples were extracted and precipitated protein with 300 μL of methanol, and 10 μL of internal standard(2.9 mg/mL, DL-o-Chlorophenylalanine) was added. The samples were vortexed for 30 s, and centrifuged at 12000 rpm and 4 °C for 15 min. 200 μL of supernatant was transferred to vial for analysis. The plasma metabolic profiles were performed on LC/MS platform (Thermo, Ultimate 3000LC, Orbitrap Elite) using Hypergod C18 (100 × 4.6 mm 3 μm) column. For chromatographic separation conditions, the column temperature was 40 °C; flow rate, 0.3 mL/min; mobile phase A, water +0.1% formic acid; mobile phase B, acetonitrile +0.1% formic acid; injection volume, 4 μL; automatic injector temperature, 4 °C.

For both faecal and plasma samples, heater temp of 300 °C, sheath gas flow rate of 45arb, aux gas flow rate of 15arb, sweep gas flow rate of 1arb, spray voltage of 3.0KV, capillary temp of 350 °C and S-lens RF level of 30% were set for positive ion mode (ES+). Heater temp of 300 °C, sheath gas flow rate of 45arb, aux gas flow rate of 15arb, sweep gas flow rate of 1arb, spray voltage of 3.2KV, capillary temp of 350 °C and S-lens RF level of 60% were set for negative ion mode (ES−).

All metabolomic data was performed feature extraction and preprocessed with Compound Discoverer 2.0 software (Thermo), and then normalized and edited into two-dimensional data matrix by excel 2010 software, including Retention time (RT), Compound Molecular Weight (compMW), Observations (samples) and peak areas. Multivariate Analysis (MVA) using SIMCA-P software (Umetrics AB, Umea, Sweden). Compounds significantly different between groups were obtained at a variable influence on projection (VIP) > 1, and p value < 0.05 based on the peak areas. The m/z value of these compounds was used to identify the metabolites corresponding to the featured peak in Metlin database. For metabolites detected in both ES+ and ES−, the data in the mode with higher VIP were retained for further analysis.

### Statistical analysis

Quantitative demographic and clinical characteristics data with normal distribution was expressed as mean ± standard deviation; t-test was used for comparison between groups. Quantitative demographic and clinical characteristics data with non-normal distribution was expressed as median (first quartile, third quartile); Wilcoxon rank sum test was used for comparison between groups. Qualitative demographic and clinical characteristics data was presented as percentage; Chi-square test was used for comparison between groups. All statistical tests were 2-sided and p < 0.05 was regarded as significant. Statistical analyses of demographic and clinical characteristics data were performed using SPSS (Version 20.0.0).

Beta-diversity analysis based on Bray Curtis distances and the visualization by using principal coordinates analysis were performed using the Vegan package in R software (Version 2.15.3) and PERMANOVA test was used for testing the significance of difference across groups. Principal component analysis was analysed using the FactoMineR package in R software (Version 2.15.3). Canonical correspondence analysis was analysed using the Vegan package in R software (Version 3.2.1). Differential abundance of gene, genera, KO and metabolites was tested by Wilcoxon rank sum test, and p values were corrected for multiple testing with Benjamin & Hochberg method. Only genera with an average relative abundance above 10^−5^ and existing in any five subjects were considered in the analyses. Spearman’s correlation coefficients between marker CAGs were calculated in R software (Version 3.2.1), and p values were corrected for multiple testing with Benjamin & Hochberg method. The co-occurrence network was plotted using Cytoscape (Version 3.2.1). Spearman’s correlation coefficients between differentially enriched genera and metabolites were calculated in R software (Version 3.2.1) and visualized using ComplexHeatmap package in R software(Version 3.2.1), and p values were corrected for multiple testing with Benjamin & Hochberg method.

### Data Availability

The datasets generated during and/or analysed during the current study are available from the corresponding author on reasonable request.

## Electronic supplementary material

Supplementary Information
