# Supplementary material for: Metagenomic and metabolomic analyses unveil dysbiosis of gut microbiota in chronic heart failure patients
Source: Sci Rep. 2018 Jan 12;8:635. doi: 10.1038/s41598-017-18756-2 (PMC5766622; doi:10.1038/s41598-017-18756-2)
Supplement: Supplementary file 1 — Supplementary Information [file 41598_2017_18756_MOESM1_ESM.pdf]

Metagenomic and metabolomic analyses unveil dysbiosis of gut microbiota in chronic heart failure patients

Xiao Cui[1], Lei Ye[2], Jing Li[3], Ling Jin[1], Wenjie Wang[1], Shuangyue Li[1], Minghui Bao[3], Shouling Wu[4], Lifeng Li[2], Bin Geng[1], Xin Zhou[5], Jian Zhang[1*], Jun Cai[1*]

[1]Fuwai Hospital, State Key Laboratory of Cardiovascular Diseases, National Center for Cardiovascular Diseases, Chinese Academy of Medical Sciences and Peking Union Medical College, Beijing 100037, P.R. China

[2]Novogene Bioinformatics Institute, Beijing 100000, P.R. China

[3]Department of Cardiology, Beijing Chao Yang Hospital, Capital Medical University, Beijing 100020, China

[4]Department of Cardiology, Kailuan General Hospital, Hebei Union University, Tangshan 063000, P.R. China

[5]Tianjin Key Laboratory of Cardiovascular Remodeling and Target Organ Injury, Pingjin Hospital Heart Center, Tianjin 300162, P.R. China

[*]Correspondence should be address to:

Jun Cai, Tel: +86 138 1061 5602; Fax: +86 010 8839 8530; Email: caijun@fuwaihospital.org; Fuwai Hospital, State Key Laboratory of Cardiovascular Diseases, National Center for Cardiovascular Diseases, Chinese Academy of Medical Sciences and Peking Union Medical College, Xicheng District, North Lishi Road, No. 167, Beijing 100037, China

Jian Zhang, Tel: +86 139 1110 2015; Fax: +86 010 8839 8530; Email: fwzhangjian62@126.com; Fuwai Hospital, State Key Laboratory of Cardiovascular

Diseases, National Center for Cardiovascular Diseases, Chinese Academy of Medical Sciences and Peking Union Medical College, Xicheng District, North Lishi Road, No. 167, Beijing 100037, China

Supplementary figures and figure legends

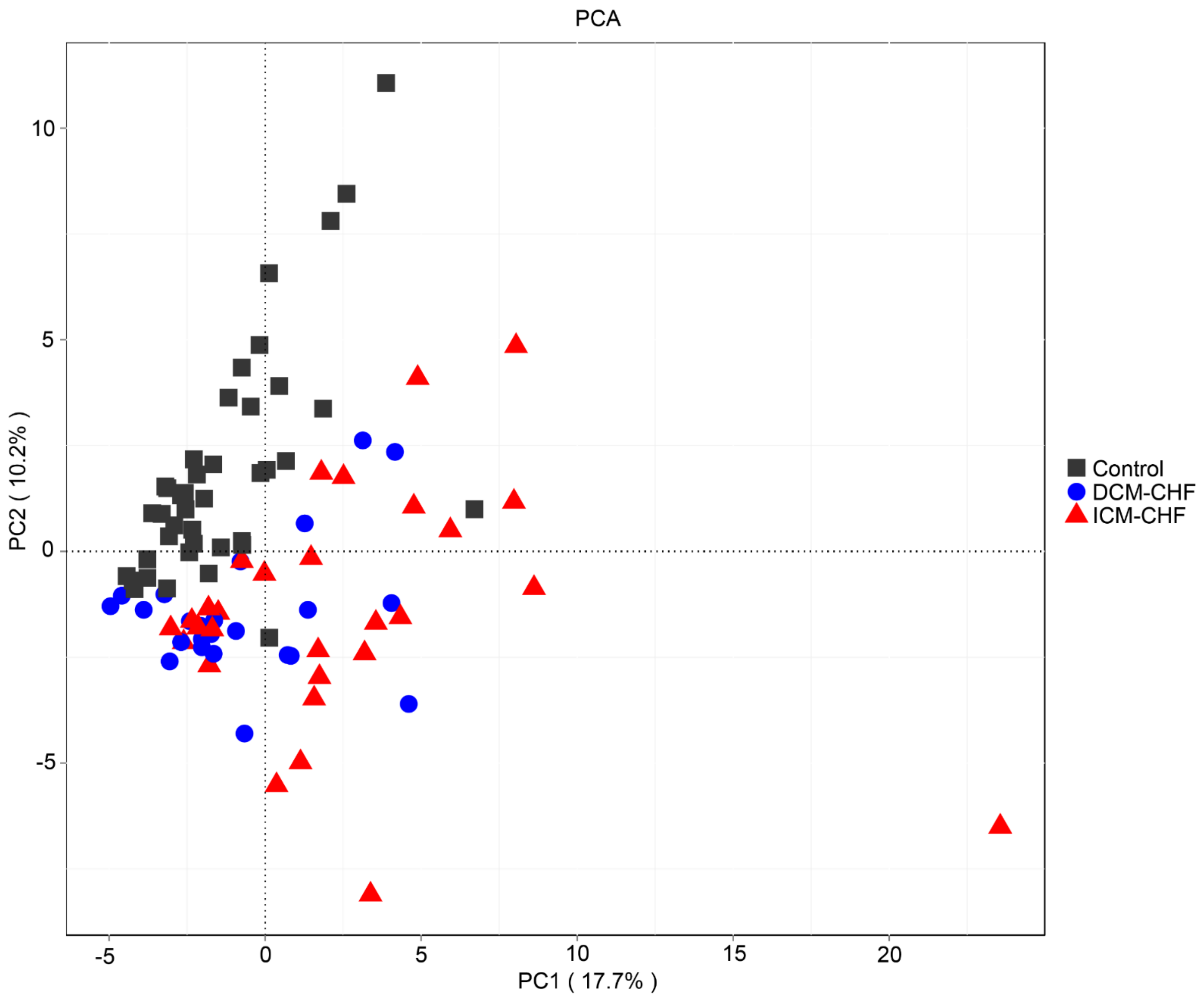

Supplementary Fig. S1. Principal component analysis of the 86 genera differentially enriched across controls, DCM- and ICM-induced CHF patients. The ■ represents control. The ● represents DCM-induced CHF. The ▲ represents ICM-induced CHF. CHF = chronic heart failure; DCM = dilated cardiomyopathy; ICM = ischaemic cardiomyopathy.

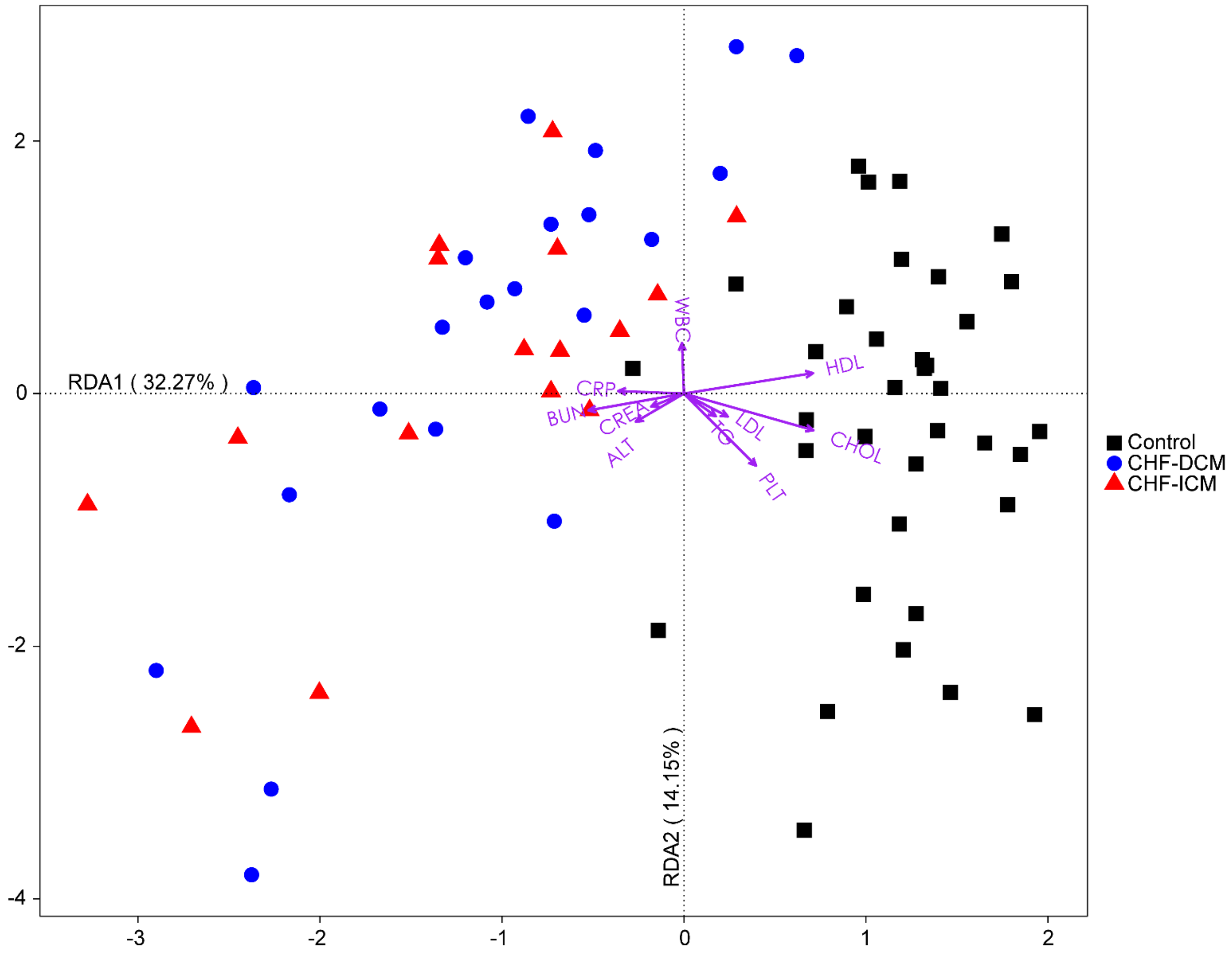

Supplementary Fig. S2. Influence of several serum indexes on the gut microbiota. Canonical correspondence analysis for influence of several serum indexes on gut microbiota differentially enriched across controls, DCM- and ICM-induced CHF patients. The ■ represents control. The ● represents DCM-induced CHF. The ▲ represents ICM-induced CHF. CHF = chronic heart failure; DCM = dilated cardiomyopathy; ICM = ischaemic cardiomyopathy; WBC = white blood cell; PLT = platelet; CRP = C-reactive protein; BUN = blood urea nitrogen; CREA = serum creatinine; ALT = alanine aminotransferase; CHOL = cholesterol; TG = triglyceride; HDL = high density lipoprotein; LDL = low density lipoprotein.

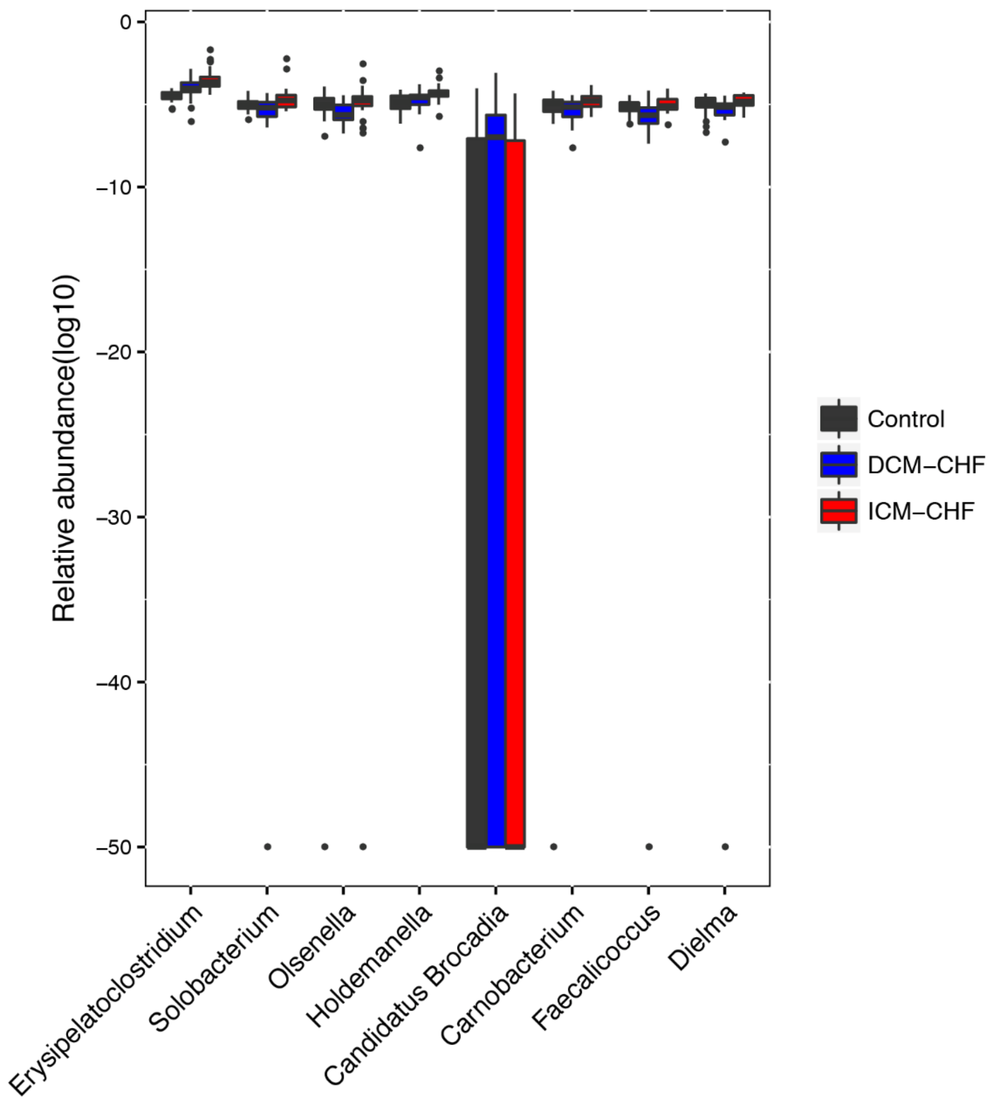

Supplementary Fig. S3. Genera differentially enriched between DCM- and ICM-induced CHF. Boxplot of all 8 genera differentially enriched between DCM- and ICM-induced CHF. Black, controls; blue, DCM-induced CHF patients; red, ICM- induced CHF patients. CHF = chronic heart failure; DCM = dilated cardiomyopathy; ICM = ischaemic cardiomyopathy.

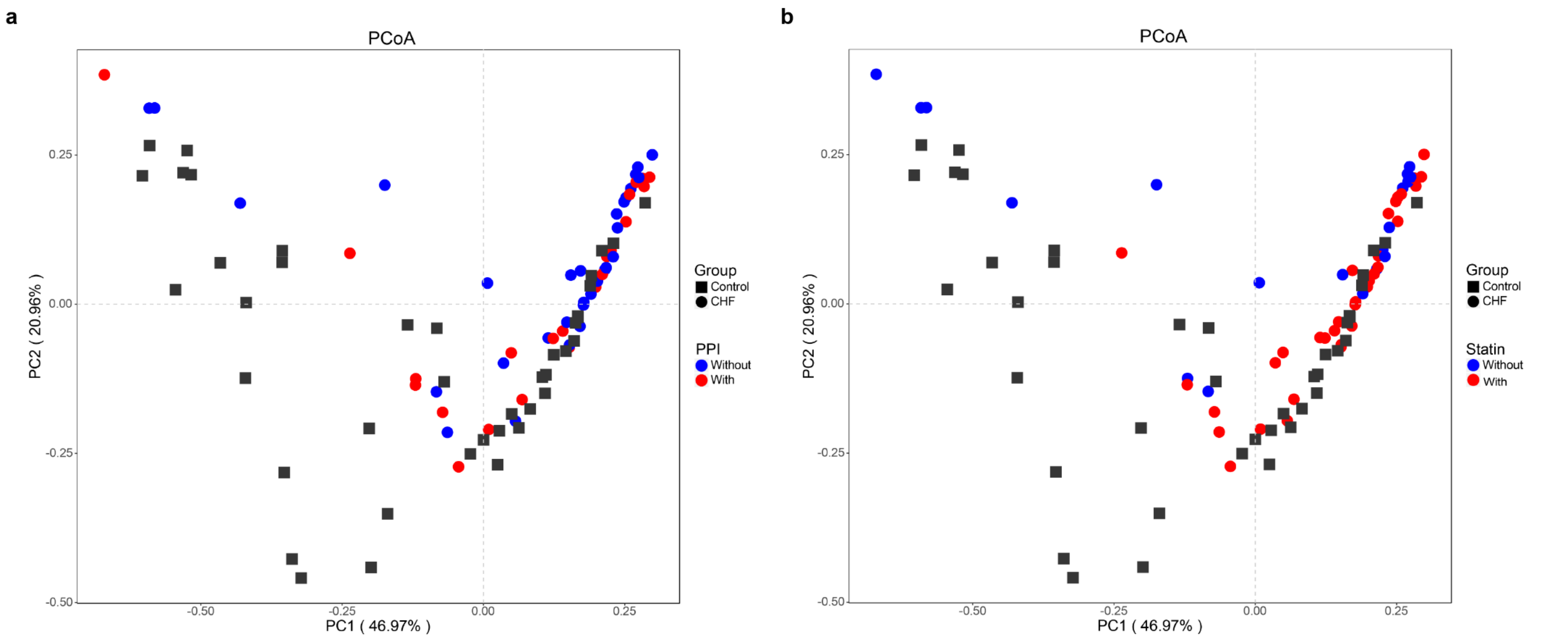

Supplementary Fig. S4. Principal coordinates analysis of beta-diversity analysis based on Bray Curtis distances of 86 genera differentially enriched between controls and CHF patients with and without PPIs or statins usage. (a) Principal coordinates analysis of beta-diversity analysis based on Bray Curtis distances of 86 genera differentially enriched between controls and CHF patients with and without PPIs usage. (b) Principal coordinates

analysis of beta-diversity analysis based on Bray Curtis distances of 86 genera differentially enriched between controls and CHF patients with and without statins usage. The ● represents CHF subjects without PPIs or statins usage. The ● represents CHF subjects with PPIs or statins usage.

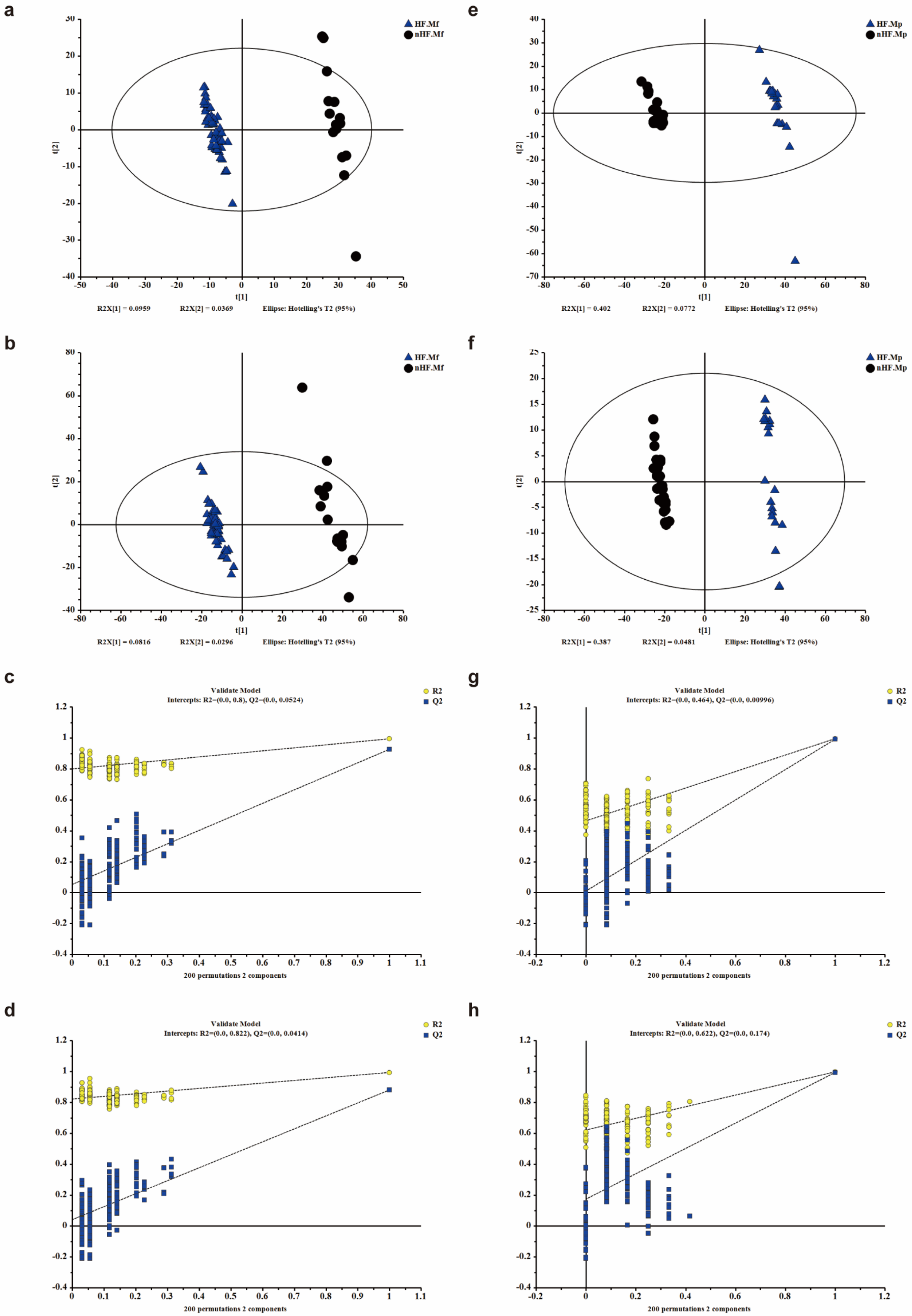
a
HF.Mf
nHF.Mf
t[1]
t[2]
R2X[1] = 0.0959
R2X[2] = 0.0369
Ellipse: Hotelling's T2 (95%)
b
HF.Mf
nHF.Mf
R2X[1] = 0.0816
R2X[2] = 0.0296
Ellipse: Hotelling's T2 (95%)
c
Validate Model
Intercepts: R2=(0.0, 0.8), Q2=(0.0, 0.0524)
R2
Q2
200 permutations 2 components
d
Validate Model
Intercepts: R2=(0.0, 0.822), Q2=(0.0, 0.0414)
200 permutations 2 components
e
HF.Mp
nHF.Mp
R2X[1] = 0.402
R2X[2] = 0.0772
Ellipse: Hotelling's T2 (95%)
f
HF.Mp
nHF.Mp
R2X[1] = 0.387
R2X[2] = 0.0481
Ellipse: Hotelling's T2 (95%)
g
Validate Model
Intercepts: R2=(0.0, 0.464), Q2=(0.0, 0.00996)
200 permutations 2 components
h
Validate Model
Intercepts: R2=(0.0, 0.622), Q2=(0.0, 0.174)
200 permutations 2 components

Supplementary Fig. S5. Metabolomic analyses of faecal and plasma samples. (a, b) The PLS-DA scores plot based on faecal metabolic profiles in ES+ (a) and ES- (b). (c, d) Validation of PLS-DA model based on faecal metabolic profiles in ES+ (c) and ES- (d), showing no overfitting phenomenon, which reprsented that this model could well describe the samples and could be applied in further data analysis. (e, f) The PLS-DA scores plot based on plasma metabolic profiles in ES+ (e) and ES- (f). (g, h) Validation of PLS-DA model based on faecal metabolic profiles in ES+ (g) and ES- (h), showing no overfitting phenomenon. The ▲ represents metabolic profiles of CHF patients. The ● represents metabolic profiles of controls. ES+ = positive ion mode; ES- = negative ion mode; CHF = chronic heart failure.

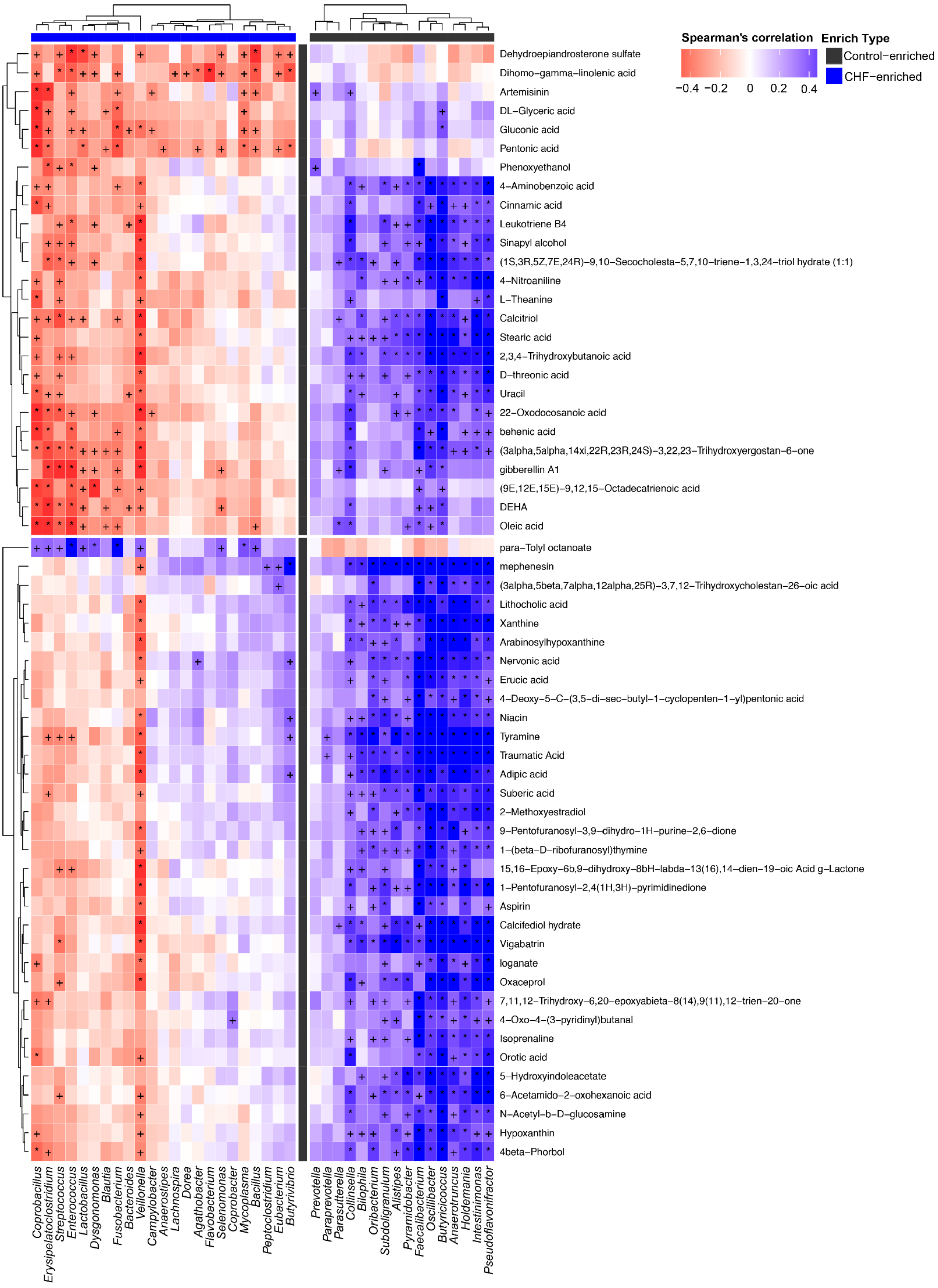

Spearman's correlation
−0.4 −0.2 0 0.2 0.4
Enrich Type
Control−enriched
CHF−enriched
Dehydroepiandrosterone sulfate
Dihomo−gamma−linolenic acid
Artemisinin
DL−Glyceric acid
Gluconic acid
Pentonic acid
Phenoxyethanol
4−Aminobenzoic acid
Cinnamic acid
Leukotriene B4
Sinapyl alcohol
(1S,3R,5Z,7E,24R)−9,10−Secocholesta−5,7,10−triene−1,3,24−triol hydrate (1:1)
4−Nitroaniline
L−Theanine
Calcitriol
Stearic acid
2,3,4−Trihydroxybutanoic acid
D−threonic acid
Uracil
22−Oxodocosanoic acid
behenic acid
(3alpha,5alpha,14xi,22R,23R,24S)−3,22,23−Trihydroxyergostan−6−one
gibberellin A1
(9E,12E,15E)−9,12,15−Octadecatrienoic acid
DEHA
Oleic acid
para−Tolyl octanoate
mephenesin
(3alpha,5beta,7alpha,12alpha,25R)−3,7,12−Trihydroxycholestan−26−oic acid
Lithocholic acid
Xanthine
Arabinosylhypoxanthine
Nervonic acid
Erucic acid
4−Deoxy−5−C−(3,5−di−sec−butyl−1−cyclopenten−1−yl)pentonic acid
Niacin
Tyramine
Traumatic Acid
Adipic acid
Suberic acid
2−Methoxyestradiol
9−Pentofuranosyl−3,9−dihydro−1H−purine−2,6−dione
1−(beta−D−ribofuranosyl)thymine
15,16−Epoxy−6b,9−dihydroxy−8bH−labda−13(16),14−dien−19−oic Acid g−Lactone
1−Pentofuranosyl−2,4(1H,3H)−pyrimidinedione
Aspirin
Calcifediol hydrate
Vigabatrin
loganate
Oxaceprol
7,11,12−Trihydroxy−6,20−epoxyabieta−8(14),9(11),12−trien−20−one
4−Oxo−4−(3−pyridinyl)butanal
Isoprenaline
Orotic acid
5−Hydroxyindoleacetate
6−Acetamido−2−oxohexanoic acid
N−Acetyl−b−D−glucosamine
Hypoxanthin
4beta−Phorbol
Coprobacillus
Erysipelatoclostridium
Streptococcus
Enterococcus
Lactobacillus
Dysgonomonas
Blautia
Fusobacterium
Bacteroides
Veillonella
Campylobacter
Anaerostipes
Lachnospira
Dorea
Agathobacter
Flavobacterium
Selenomonas
Coprobacter
Mycoplasma
Bacillus
Peptoclostridium
Eubacterium
Butyrivibrio
Prevotella
Paraprevotella
Parasutterella
Collinsella
Bilophila
Oribacterium
Subdoligranulum
Alistipes
Pyramidobacter
Faecalibacterium
Oscillibacter
Butyricicoccus
Anaerotruncus
Holdemania
Intestinimonas
Pseudoflavonifractor

Supplementary Fig. S6. Correlation between faecal metabolic patterns and genera. Spearman's correlation coefficients were calculated between the relative abundance of top 40 differentially enriched genera and the level of faecal metabolic patterns. Those with low correlation ($|r| < 0.5$) were not shown. Red, negative correlation; blue, positive correlation, +, $q < 0.05$, *, $q < 0.01$. The enriched type of each genera and metabolic patterns was coloured according to its direction of enrichment. Black, enriched in controls; Blue, enriched in CHF patients.

Supplementary tables and table legends

marker CAGs vs. marker CAGs	SCC index	p value	q value
Control-3-vs-Control-4	0.833	2.14E-25	6.88E-25
Control-3-vs-Control-5	0.871	3.60E-30	1.46E-29
Control-3-vs-Unclassified_Firmicutes	0.858	1.27E-16	3.11E-16
Control-3-vs-Control-8	0.844	1.30E-26	4.41E-26
Control-3-vs-Control-10	0.897	2.01E-34	9.90E-34
Control-3-vs-Control-13	0.907	2.71E-36	1.48E-35
Control-3-vs-Control-16	0.82	5.14E-24	1.55E-23
Control-3-vs-Unclassified_Firmicutes	0.862	1.27E-16	3.11E-16
Control-3-vs-Control-18	0.813	2.41E-23	7.06E-23
Control-3-vs-Unclassified_Firmicutes	0.849	1.27E-16	3.11E-16
Control-3-vs-Unclassified_Firmicutes	0.868	1.27E-16	3.11E-16
Control-3-vs-Unclassified_Firmicutes	0.8	1.27E-16	3.11E-16
Control-3-vs-Unclassified_Firmicutes	0.844	1.27E-16	3.11E-16
Control-3-vs-Unclassified_Firmicutes	0.813	1.27E-16	3.11E-16
Control-3-vs-[Ruminococcus] gnavus	-0.559	4.00E-09	5.86E-09
Control-3-vs-Unclassified_Firmicutes	0.828	1.27E-16	3.11E-16
Control-3-vs-Unclassified_Firmicutes	0.814	1.27E-16	3.11E-16
Control-3-vs-Unclassified_Firmicutes	0.801	1.27E-16	3.11E-16
Control-3-vs-[Ruminococcus] gnavus	-0.554	4.00E-09	5.86E-09
Control-3-vs-[Ruminococcus] gnavus	-0.561	4.00E-09	5.86E-09
Control-3-vs-Control-129	0.85	2.70E-27	9.58E-27
Control-4-vs-Control-5	0.843	1.63E-26	5.44E-26

Control-4-vs-Control-10	0.848	3.66E-27	1.26E-26
Control-4-vs-Control-13	0.844	1.39E-26	4.69E-26
Control-4-vs-Control-16	0.816	1.35E-23	4.00E-23
Control-4-vs-Control-18	0.824	1.87E-24	5.84E-24
Control-4-vs-Control-30	0.817	1.12E-23	3.33E-23
Control-4-vs-Unclassified_Firmicutes	0.82	1.59E-15	3.85E-15
Control-4-vs-[Ruminococcus] gnavus	-0.553	1.07E-08	1.41E-08
Control-5-vs-Control-8	0.836	1.13E-25	3.71E-25
Control-5-vs-Control-10	0.907	2.74E-36	1.48E-35
Control-5-vs-Control-13	0.858	2.34E-28	8.74E-28
Control-5-vs-Control-16	0.903	1.97E-35	1.03E-34
Control-5-vs-Control-18	0.853	1.07E-27	3.93E-27
Unclassified_Firmicutes-vs-Control-13	0.835	2.22E-15	4.81E-15
Unclassified_Firmicutes-vs-Control-129	0.863	2.13E-15	4.71E-15
Oscillibacter-vs-Oscillibacter sp. ER4	0.968	6.13E-05	6.85E-05
Oscillibacter-vs-Control-22	0.883	4.93E-06	5.86E-06
Oscillibacter-vs-Control-28	0.875	2.94E-08	3.77E-08
Oscillibacter-vs-[Ruminococcus] gnavus	-0.627	0.003533	0.003652
Oscillibacter-vs-[Ruminococcus] gnavus	-0.626	0.003533	0.003652
Oscillibacter-vs-[Ruminococcus] gnavus	-0.622	0.003533	0.003652
Control-8-vs-Control-10	0.858	2.23E-28	8.42E-28
Control-8-vs-Control-16	0.868	1.09E-29	4.38E-29
Control-8-vs-Control-18	0.832	3.07E-25	9.75E-25
Unclassified_Firmicutes-vs-Control-30	0.808	4.14E-17	1.09E-16
Unclassified_Firmicutes-vs-Control-76	0.867	2.11E-15	4.71E-15

Control-10-vs-Control-13	0.862	6.71E-29	2.62E-28
Control-10-vs-Control-16	0.896	3.28E-34	1.55E-33
Control-10-vs-Control-18	0.834	1.70E-25	5.50E-25
Control-10-vs-Unclassified_Firmicutes	0.856	1.20E-14	2.56E-14
Control-10-vs-Unclassified_Firmicutes	0.8	1.20E-14	2.56E-14
Control-10-vs-Control-129	0.805	1.42E-22	4.05E-22
Unclassified_Firmicutes-vs-Control-76	0.855	2.11E-15	4.71E-15
Control-13-vs-Control-16	0.803	2.19E-22	6.19E-22
Control-13-vs-Unclassified_Firmicutes	0.801	2.22E-15	4.81E-15
Control-13-vs-Control-18	0.829	5.85E-25	1.84E-24
Control-13-vs-Unclassified_Firmicutes	0.853	2.22E-15	4.81E-15
Control-13-vs-[Ruminococcus] gnavus	-0.627	1.14E-11	2.32E-11
Control-13-vs-[Ruminococcus] gnavus	-0.625	1.14E-11	2.32E-11
Control-13-vs-[Ruminococcus] gnavus	-0.629	1.14E-11	2.32E-11
Control-13-vs-Control-129	0.852	1.22E-27	4.40E-27
Oscillibacter sp. ER4-vs-Oscillibacter	0.977	6.13E-05	6.85E-05
Oscillibacter sp. ER4-vs-Control-22	0.846	4.05E-06	4.86E-06
Oscillibacter sp. ER4-vs-Oscillibacter	0.963	6.13E-05	6.85E-05
Oscillibacter sp. ER4-vs-Control-28	0.837	8.88E-08	1.09E-07
Oscillibacter sp. ER4-vs-[Ruminococcus] gnavus	-0.599	5.32E-06	6.26E-06
Oscillibacter sp. ER4-vs-[Ruminococcus] gnavus	-0.6	5.32E-06	6.26E-06
Oscillibacter sp. ER4-vs-[Ruminococcus] gnavus	-0.599	5.32E-06	6.26E-06
Faecalibacterium-vs-HF-43	-0.55	2.70E-05	3.10E-05
Faecalibacterium-vs-[Ruminococcus] gnavus	-0.553	0.001583	0.001692
Faecalibacterium-vs-Control-61	0.987	0.001315	0.001433

Control-16-vs-Control-18	0.86	1.44E-28	5.58E-28
Control-16-vs-[Ruminococcus] gnavus	-0.606	6.55E-10	1.20E-09
Control-16-vs-[Ruminococcus] gnavus	-0.592	6.55E-10	1.20E-09
Control-16-vs-[Ruminococcus] gnavus	-0.584	6.55E-10	1.20E-09
Unclassified_Firmicutes-vs-Control-30	0.816	4.14E-17	1.09E-16
Unclassified_Firmicutes-vs-Control-76	0.831	2.11E-15	4.71E-15
Unclassified_Firmicutes-vs-Oscillibacter	0.807	0.009138	0.009415
Oscillibacter-vs-Control-22	0.806	4.93E-06	5.86E-06
Oscillibacter-vs-[Ruminococcus] gnavus	-0.556	0.003533	0.003652
Oscillibacter-vs-[Ruminococcus] gnavus	-0.558	0.003533	0.003652
Oscillibacter-vs-[Ruminococcus] gnavus	-0.556	0.003533	0.003652
Unclassified_Firmicutes-vs-Control-30	0.859	4.14E-17	1.09E-16
Unclassified_Firmicutes-vs-Control-45	0.863	1.03E-18	2.88E-18
Faecalibacterium-vs-Control-61	0.987	0.001315	0.001433
Control-22-vs-Oscillibacter	0.802	4.93E-06	5.86E-06
Control-22-vs-Control-28	0.943	1.37E-45	1.11E-44
Control-22-vs-HF-43	-0.629	1.12E-11	2.31E-11
Control-22-vs-[Ruminococcus] gnavus	-0.638	3.25E-10	6.15E-10
Control-22-vs-[Ruminococcus] gnavus	-0.622	3.25E-10	6.15E-10
Control-22-vs-HF-78	-0.591	3.50E-10	6.57E-10
Control-22-vs-HF-84	-0.574	1.43E-09	2.41E-09
Control-22-vs-HF-90	-0.571	1.91E-09	3.21E-09
Control-22-vs-[Ruminococcus] gnavus	-0.592	3.25E-10	6.15E-10
Control-22-vs-HF-102	-0.554	6.88E-09	9.39E-09
Unclassified_Firmicutes-vs-Control-129	0.832	2.13E-15	4.71E-15

Oscillibacter-vs-[Ruminococcus] gnavus	-0.55	0.003533	0.003652
Oscillibacter-vs-[Ruminococcus] gnavus	-0.551	0.003533	0.003652
Oscillibacter-vs-[Ruminococcus] gnavus	-0.553	0.003533	0.003652
Control-28-vs-HF-43	-0.616	3.88E-11	7.77E-11
Control-28-vs-[Ruminococcus] gnavus	-0.666	8.34E-12	1.74E-11
Control-28-vs-[Ruminococcus] gnavus	-0.653	8.34E-12	1.74E-11
Control-28-vs-HF-78	-0.587	5.24E-10	9.74E-10
Control-28-vs-HF-84	-0.562	3.75E-09	5.60E-09
Control-28-vs-HF-90	-0.566	2.84E-09	4.50E-09
Control-28-vs-[Ruminococcus] gnavus	-0.632	8.34E-12	1.74E-11
Control-28-vs-HF-102	-0.555	6.51E-09	8.93E-09
Control-28-vs-Erysipelatoclostridium	-0.558	5.14E-09	7.37E-09
Control-30-vs-Control-31	0.82	4.81E-24	1.46E-23
Control-30-vs-Unclassified_Firmicutes	0.826	4.14E-17	1.09E-16
Control-30-vs-Control-45	0.82	4.63E-24	1.42E-23
Control-30-vs-[Ruminococcus] gnavus	-0.566	7.03E-09	9.44E-09
Control-30-vs-Unclassified_Firmicutes	0.839	4.14E-17	1.09E-16
Control-30-vs-Control-76	0.841	3.02E-26	9.97E-26
Control-30-vs-[Ruminococcus] gnavus	-0.554	7.03E-09	9.44E-09
Control-30-vs-Control-112	0.808	7.89E-23	2.27E-22
Control-30-vs-Unclassified_Firmicutes	0.835	4.14E-17	1.09E-16
Control-30-vs-Unclassified_Firmicutes	0.843	4.14E-17	1.09E-16
Faecalibacterium-vs-Control-61	0.958	0.001315	0.001433
Control-33-vs-Control-42	0.958	6.62E-52	7.26E-51
Control-33-vs-Control-46	0.95	3.25E-48	2.91E-47

Control-33-vs-[Ruminococcus] gnavus	-0.563	6.30E-08	7.85E-08
Control-33-vs-[Ruminococcus] gnavus	-0.555	6.30E-08	7.85E-08
Faecalibacterium prausnitzii-vs-Faecalibacterium	0.981	3.41E-05	3.86E-05
Faecalibacterium prausnitzii-vs-Faecalibacterium	0.954	3.41E-05	3.86E-05
Faecalibacterium prausnitzii-vs-Faecalibacterium	0.971	3.41E-05	3.86E-05
Unclassified_Firmicutes-vs-[Ruminococcus] gnavus	-0.559	1.46E-05	1.69E-05
Unclassified_Firmicutes-vs-[Ruminococcus] gnavus	-0.56	1.46E-05	1.69E-05
Unclassified_Firmicutes-vs-Control-129	0.834	2.13E-15	4.71E-15
Unclassified_Firmicutes-vs-Control-76	0.857	2.11E-15	4.71E-15
Unclassified_Firmicutes-vs-Control-129	0.801	2.13E-15	4.71E-15
Control-42-vs-Control-46	0.971	5.46E-59	7.42E-58
Control-42-vs-[Ruminococcus] gnavus	-0.56	7.53E-08	9.30E-08
Control-42-vs-[Ruminococcus] gnavus	-0.55	7.53E-08	9.30E-08
HF-43-vs-Faecalibacterium prausnitzii	-0.553	4.02E-09	5.86E-09
HF-43-vs-Faecalibacterium prausnitzii	-0.561	4.02E-09	5.86E-09
HF-43-vs-HF-78	0.937	6.30E-44	4.65E-43
HF-43-vs-HF-84	0.887	1.07E-32	4.87E-32
HF-43-vs-HF-90	0.923	6.11E-40	3.92E-39
HF-43-vs-Control-94	-0.58	8.77E-10	1.56E-09
HF-43-vs-HF-96	0.873	1.96E-30	8.12E-30
HF-43-vs-HF-102	0.884	4.65E-32	2.05E-31
HF-43-vs-Control-103	-0.637	5.09E-12	1.08E-11
HF-43-vs-Control-130	-0.561	4.06E-09	5.88E-09
HF-43-vs-Control-131	-0.608	7.74E-11	1.52E-10
HF-43-vs-Control-133	-0.552	8.35E-09	1.10E-08

HF-43-vs-Control-141	-0.561	4.05E-09	5.88E-09
HF-43-vs-Control-158	-0.63	1.00E-11	2.08E-11
HF-43-vs-Unclassified_Firmicutes	-0.569	6.49E-05	7.21E-05
HF-43-vs-Control-164	-0.554	6.96E-09	9.43E-09
HF-43-vs-Control-196	-0.567	2.48E-09	3.96E-09
HF-43-vs-Control-197	-0.583	7.13E-10	1.28E-09
HF-43-vs-Control-215	-0.562	3.68E-09	5.58E-09
HF-43-vs-Control-235	-0.572	1.74E-09	2.92E-09
HF-43-vs-Unclassified_Firmicutes	-0.555	6.49E-05	7.21E-05
HF-43-vs-Faecalibacterium	-0.615	2.70E-05	3.10E-05
HF-43-vs-Control-425	-0.6	1.62E-10	3.15E-10
Control-45-vs-Unclassified_Firmicutes	0.824	1.03E-18	2.88E-18
Control-46-vs-[Ruminococcus] gnavus	-0.564	5.02E-08	6.32E-08
Control-46-vs-[Ruminococcus] gnavus	-0.555	5.02E-08	6.32E-08
[Clostridium] clostridioforme-vs-Lachnoclostridium	0.997	8.02E-07	9.70E-07
Faecalibacterium prausnitzii-vs-[Ruminococcus] gnavus	-0.578	1.07E-09	1.82E-09
Faecalibacterium prausnitzii-vs-[Ruminococcus] gnavus	-0.57	1.07E-09	1.82E-09
Eubacterium sp. CAG:115-vs-Eubacterium	0.879	0.01011	0.010354
Eubacterium sp. CAG:115-vs-Eubacterium	0.84	0.01011	0.010354
Eubacterium sp. CAG:115-vs-Veillonella sp. 6_1_27	-0.555	5.13E-08	6.43E-08
Unclassified_Firmicutes-vs-Control-76	0.859	2.11E-15	4.71E-15
[Ruminococcus] gnavus-vs-Faecalibacterium prausnitzii	-0.562	1.07E-09	1.82E-09
[Ruminococcus] gnavus-vs-Faecalibacterium prausnitzii	-0.554	1.07E-09	1.82E-09
[Ruminococcus] gnavus-vs-Faecalibacterium prausnitzii	-0.595	1.07E-09	1.82E-09
[Ruminococcus] gnavus-vs-Control-71	-0.55	7.27E-09	9.65E-09

[Ruminococcus] gnavus-vs-Control-94	-0.6	3.29E-09	5.10E-09
[Ruminococcus] gnavus-vs-Unclassified_Firmicutes	-0.558	1.46E-05	1.69E-05
[Ruminococcus] gnavus-vs-Control-103	-0.553	4.93E-07	6.00E-07
[Ruminococcus] gnavus-vs-Faecalibacterium	-0.611	0.001583	0.001692
[Ruminococcus] gnavus-vs-Control-130	-0.596	2.33E-09	3.75E-09
[Ruminococcus] gnavus-vs-Control-133	-0.578	1.71E-08	2.23E-08
[Ruminococcus] gnavus-vs-Control-141	-0.592	2.01E-09	3.31E-09
[Ruminococcus] gnavus-vs-Control-158	-0.598	3.75E-09	5.60E-09
[Ruminococcus] gnavus-vs-Control-193	-0.578	6.02E-09	8.39E-09
[Ruminococcus] gnavus-vs-Control-197	-0.599	3.37E-09	5.14E-09
[Ruminococcus] gnavus-vs-Control-215	-0.565	3.61E-08	4.58E-08
[Ruminococcus] gnavus-vs-Control-235	-0.553	1.06E-07	1.30E-07
[Ruminococcus] gnavus-vs-Unclassified_Firmicutes	-0.55	1.46E-05	1.69E-05
[Ruminococcus] gnavus-vs-Faecalibacterium	-0.582	0.001583	0.001692
[Ruminococcus] gnavus-vs-Control-431	-0.58	3.50E-08	4.46E-08
Unclassified_Firmicutes-vs-Control-129	0.822	2.13E-15	4.71E-15
Unclassified_Firmicutes-vs-Control-129	0.802	2.13E-15	4.71E-15
Faecalibacterium prausnitzii-vs-[Ruminococcus] gnavus	-0.557	1.07E-09	1.82E-09
Faecalibacterium prausnitzii-vs-HF-78	-0.586	6.02E-09	8.39E-09
Faecalibacterium prausnitzii-vs-HF-84	-0.573	3.39E-07	4.14E-07
Faecalibacterium prausnitzii-vs-HF-90	-0.56	8.64E-07	1.04E-06
Faecalibacterium prausnitzii-vs-[Ruminococcus] gnavus	-0.593	1.07E-09	1.82E-09
Faecalibacterium prausnitzii-vs-HF-78	-0.556	6.02E-09	8.39E-09
Faecalibacterium prausnitzii-vs-[Ruminococcus] gnavus	-0.578	1.07E-09	1.82E-09
Faecalibacterium prausnitzii-vs-Faecalibacterium	0.962	3.41E-05	3.86E-05

Control-71-vs-[Ruminococcus] gnavus	-0.553	7.27E-09	9.65E-09
Control-71-vs-[Ruminococcus] gnavus	-0.553	7.27E-09	9.65E-09
Unclassified_Firmicutes-vs-Control-76	0.81	2.11E-15	4.71E-15
Control-76-vs-Unclassified_Firmicutes	0.866	2.11E-15	4.71E-15
Control-76-vs-Unclassified_Firmicutes	0.828	2.11E-15	4.71E-15
[Ruminococcus] gnavus-vs-Control-94	-0.586	3.29E-09	5.10E-09
[Ruminococcus] gnavus-vs-Unclassified_Firmicutes	-0.555	1.46E-05	1.69E-05
[Ruminococcus] gnavus-vs-Faecalibacterium	-0.61	0.001583	0.001692
[Ruminococcus] gnavus-vs-Control-130	-0.584	2.33E-09	3.75E-09
[Ruminococcus] gnavus-vs-Control-133	-0.562	1.71E-08	2.23E-08
[Ruminococcus] gnavus-vs-Control-141	-0.586	2.01E-09	3.31E-09
[Ruminococcus] gnavus-vs-Control-158	-0.581	3.75E-09	5.60E-09
[Ruminococcus] gnavus-vs-Control-193	-0.586	6.02E-09	8.39E-09
[Ruminococcus] gnavus-vs-Control-197	-0.582	3.37E-09	5.14E-09
[Ruminococcus] gnavus-vs-Faecalibacterium	-0.578	0.001583	0.001692
[Ruminococcus] gnavus-vs-Control-431	-0.571	3.50E-08	4.46E-08
HF-78-vs-HF-84	0.911	4.16E-37	2.44E-36
HF-78-vs-HF-90	0.897	2.57E-34	1.23E-33
HF-78-vs-HF-96	0.886	2.06E-32	9.22E-32
HF-78-vs-HF-102	0.871	3.22E-30	1.32E-29
HF-78-vs-Control-103	-0.626	1.45E-11	2.91E-11
HF-78-vs-Control-108	-0.567	2.65E-09	4.22E-09
HF-78-vs-Control-131	-0.57	2.02E-09	3.31E-09
HF-78-vs-Control-149	-0.555	6.38E-09	8.78E-09
HF-78-vs-Control-157	-0.569	2.18E-09	3.57E-09

HF-78-vs-Control-158	-0.564	3.16E-09	4.98E-09
HF-78-vs-Control-196	-0.583	7.10E-10	1.28E-09
HF-78-vs-Control-230	-0.556	6.13E-09	8.47E-09
HF-78-vs-Faecalibacterium	-0.638	0.000227	0.000251
HF-78-vs-Control-425	-0.608	8.05E-11	1.57E-10
Lachnoclostridium-vs-[Clostridium] clostridioforme	0.989	8.02E-07	9.70E-07
HF-84-vs-HF-90	0.909	1.19E-36	6.86E-36
HF-84-vs-HF-96	0.919	6.09E-39	3.76E-38
HF-84-vs-HF-102	0.859	1.84E-28	7.03E-28
HF-84-vs-Control-103	-0.594	2.86E-10	5.49E-10
HF-84-vs-Control-158	-0.56	4.25E-09	6.13E-09
HF-84-vs-Control-196	-0.568	2.42E-09	3.88E-09
HF-84-vs-Faecalibacterium	-0.583	0.001728	0.001842
HF-90-vs-HF-96	0.889	4.88E-33	2.27E-32
HF-90-vs-HF-102	0.938	3.49E-44	2.64E-43
HF-90-vs-Control-103	-0.597	2.06E-10	3.98E-10
HF-90-vs-Control-158	-0.556	6.02E-09	8.39E-09
HF-90-vs-Control-196	-0.556	6.07E-09	8.42E-09
HF-90-vs-Faecalibacterium	-0.609	3.82E-05	4.32E-05
HF-90-vs-Control-425	-0.583	6.94E-10	1.25E-09
Control-94-vs-[Ruminococcus] gnavus	-0.564	3.29E-09	5.10E-09
Control-94-vs-Control-130	0.959	5.22E-52	5.92E-51
Control-94-vs-Control-133	0.944	2.96E-46	2.45E-45
Control-94-vs-Control-146	0.857	3.12E-28	1.15E-27
Control-94-vs-Control-158	0.956	7.21E-51	7.66E-50

Control-94-vs-Control-197	0.937	7.30E-44	5.28E-43
Control-94-vs-Control-215	0.923	5.87E-40	3.91E-39
Control-94-vs-Control-235	0.923	6.03E-40	3.92E-39
HF-96-vs-HF-102	0.878	3.63E-31	1.54E-30
HF-96-vs-Control-103	-0.575	1.35E-09	2.30E-09
HF-96-vs-Faecalibacterium	-0.568	0.002757	0.002929
HF-96-vs-Control-425	-0.554	6.93E-09	9.43E-09
[Ruminococcus] gnavus-vs-Faecalibacterium	-0.589	0.001583	0.001692
[Ruminococcus] gnavus-vs-Control-130	-0.568	2.33E-09	3.75E-09
[Ruminococcus] gnavus-vs-Control-141	-0.57	2.01E-09	3.31E-09
[Ruminococcus] gnavus-vs-Control-158	-0.562	3.75E-09	5.60E-09
[Ruminococcus] gnavus-vs-Control-193	-0.556	6.02E-09	8.39E-09
[Ruminococcus] gnavus-vs-Control-197	-0.563	3.37E-09	5.14E-09
HF-102-vs-Control-103	-0.591	3.65E-10	6.81E-10
HF-102-vs-Faecalibacterium	-0.615	0.000246	0.000271
Control-103-vs-Veillonella sp. 6_1_27	-0.594	7.58E-11	1.50E-10
Control-103-vs-Bifidobacterium breve	-0.584	6.59E-10	1.20E-09
Control-103-vs-Erysipelatoclostridium	-0.551	8.53E-09	1.12E-08
Control-103-vs-Veillonella sp. 6_1_27	-0.609	7.58E-11	1.50E-10
Control-108-vs-Control-142	0.982	6.77E-69	1.92E-67
Control-108-vs-Control-149	0.986	2.91E-73	9.89E-72
Control-108-vs-Control-153	0.953	1.05E-49	9.88E-49
Control-108-vs-Control-157	0.94	8.95E-45	6.92E-44
Control-108-vs-Control-220	0.912	2.21E-37	1.32E-36
Control-108-vs-Control-230	0.901	4.52E-35	2.33E-34

Alistipes senegalensis-vs-Streptococcus sp. HSISS1	-0.557	5.54E-09	7.91E-09
Unclassified_Firmicutes-vs-Clostridium sp. CAG:81	0.993	0.960501	0.960501
Control-130-vs-Control-133	0.955	1.56E-50	1.61E-49
Control-130-vs-Control-146	0.85	2.13E-27	7.62E-27
Control-130-vs-Control-158	0.945	2.52E-46	2.14E-45
Control-130-vs-Control-197	0.941	4.64E-45	3.67E-44
Control-130-vs-Control-215	0.933	1.67E-42	1.19E-41
Control-130-vs-Control-235	0.925	2.40E-40	1.63E-39
Control-131-vs-Control-154	0.88	1.91E-31	8.33E-31
Control-131-vs-Control-164	0.889	6.58E-33	3.02E-32
Control-133-vs-Control-146	0.849	3.11E-27	1.08E-26
Control-133-vs-Control-158	0.919	6.09E-39	3.76E-38
Control-133-vs-Control-197	0.913	1.23E-37	7.47E-37
Control-133-vs-Control-215	0.908	1.78E-36	1.01E-35
Control-133-vs-Control-235	0.898	1.35E-34	6.73E-34
Unclassified_Firmicutes-vs-Clostridium	0.984	2.34E-08	3.01E-08
Control-142-vs-Control-149	0.979	1.93E-65	5.05E-64
Control-142-vs-Control-153	0.966	5.79E-56	7.29E-55
Control-142-vs-Control-157	0.907	2.93E-36	1.56E-35
Control-142-vs-Control-220	0.874	1.21E-30	5.08E-30
Control-142-vs-Control-230	0.866	2.27E-29	8.99E-29
Clostridium sp. CAG:81-vs-Unclassified_Firmicutes	0.971	0.960501	0.960501
Control-149-vs-Control-153	0.951	8.39E-49	7.71E-48
Control-149-vs-Control-157	0.929	2.29E-41	1.59E-40
Control-149-vs-Control-220	0.907	2.19E-36	1.22E-35

Control-149-vs-Control-230	0.897	2.36E-34	1.15E-33
Control-153-vs-Control-157	0.849	2.79E-27	9.77E-27
Control-153-vs-Control-220	0.822	2.93E-24	9.05E-24
Control-153-vs-Control-230	0.811	3.86E-23	1.12E-22
Control-154-vs-Control-164	0.899	8.03E-35	4.08E-34
Bifidobacterium breve-vs-Control-431	-0.564	3.30E-09	5.11E-09
Control-157-vs-Control-220	0.979	2.25E-65	5.48E-64
Control-157-vs-Control-230	0.979	4.39E-65	9.96E-64
Control-158-vs-Control-197	0.962	1.46E-53	1.71E-52
Control-158-vs-Control-215	0.954	5.53E-50	5.37E-49
Control-158-vs-Control-235	0.955	1.97E-50	1.97E-49
Sutterella wadsworthensis-vs-[Clostridium] spiroforme	-0.574	0.000683	0.000751
Unclassified_Firmicutes-vs-Clostridium	0.98	2.34E-08	3.01E-08
Clostridium-vs-Unclassified_Firmicutes	0.844	2.34E-08	3.01E-08
Control-197-vs-Control-215	0.969	1.02E-57	1.34E-56
Control-197-vs-Control-235	0.965	5.25E-55	6.38E-54
Parasutterella-vs-Parasutterella excrementihominis	0.816	6.03E-74	2.28E-72
Parasutterella excrementihominis-vs-Parasutterella	0.928	6.03E-74	2.28E-72
Control-215-vs-Control-235	0.983	4.75E-70	1.47E-68
Control-220-vs-Control-230	0.994	3.22E-89	3.65E-87
Parasutterella excrementihominis-vs-Parasutterella	0.911	6.03E-74	2.28E-72
Parasutterella excrementihominis-vs-Parasutterella	0.859	6.03E-74	2.28E-72
Parasutterella excrementihominis-vs-Parasutterella	0.803	6.03E-74	2.28E-72
Parasutterella excrementihominis-vs-Parasutterella	0.986	6.03E-74	2.28E-72
Bacteroides ovatus-vs-Bacteroides sp. 3_1_23	0.987	2.26E-64	3.66E-63

Bacteroides ovatus-vs-Bacteroides sp. 3_1_23	0.958	2.26E-64	3.66E-63
Bacteroides ovatus-vs-Bacteroides sp. 1_1_30	0.943	1.58E-63	2.24E-62
Bacteroides sp. 3_1_19-vs-Parabacteroides sp. CAG:2	0.949	8.93E-48	7.79E-47
Bacteroides sp. 3_1_23-vs-Bacteroides ovatus	0.962	2.26E-64	3.66E-63
Bacteroides sp. 3_1_23-vs-Bacteroides ovatus	0.914	2.26E-64	3.66E-63
Bacteroides sp. 3_1_23-vs-Bacteroides sp. 1_1_30	0.902	4.44E-90	7.55E-88
Bacteroides ovatus-vs-Bacteroides sp. 3_1_23	0.948	2.26E-64	3.66E-63
Bacteroides ovatus-vs-Bacteroides sp. 1_1_30	0.932	1.58E-63	2.24E-62
Bacteroides sp. 3_1_23-vs-Bacteroides ovatus	0.978	2.26E-64	3.66E-63
Bacteroides sp. 3_1_23-vs-Bacteroides sp. 1_1_30	0.994	4.44E-90	7.55E-88
Bacteroides ovatus-vs-Bacteroides sp. 1_1_30	0.977	1.58E-63	2.24E-62
Bacteroides sp. 9_1_42FAA-vs-Bacteroides vulgatus	0.961	0.763315	0.767831
Bacteroides sp. 9_1_42FAA-vs-Bacteroides sp. 3_1_40A	0.978	0.42018	0.425182
Bacteroides sp. 9_1_42FAA-vs-Bacteroides sp. 3_1_40A	0.876	0.42018	0.425182
Bacteroides sp. 9_1_42FAA-vs-Bacteroides vulgatus CAG:6	0.844	0.297369	0.30362
Bacteroides vulgatus-vs-Bacteroides sp. 9_1_42FAA	0.941	0.763315	0.767831
Bacteroides sp. 3_1_40A-vs-Bacteroides sp. 9_1_42FAA	0.921	0.42018	0.425182
Eubacterium-vs-Eubacterium sp. CAG:252	0.879	2.11E-31	9.09E-31

Supplementary Table S1. Spearman's correlation coefficients between marker co-abundance groups. Spearman's correlations coefficients between marker CAGs and p values were calculated and p values were corrected for multiple testing as q values with Benjamin & Hochberg method. SCC = Spearman's correlation coefficient.

Faecal Metabolites in ES+	SD of peak area of CHF	SD of peak area of controls	SD of peak area of quality controls	Levels of identification confidence
L-Proline	8.5845288	45.48906187	1.615283729	3
Indole	3.60910842	47.63950543	1.782479467	3
L-(+)-Valine	2.73532051	44.74096819	0.342323998	3
Niacin	28.1220871	39.39535612	10.5372618	3
Thymine	11.5193398	24.72728257	1.82085256	3
Vigabatrin	8.63248598	17.45197355	0.694741303	3
CREATINE	7.02771867	18.60200668	1.355806245	3
L-(+)-Leucine	138.776336	307.4436436	29.64333088	3
2,3,4-Trihydroxybutanoic acid	0.96030018	9.203876076	0.059742176	3
D-threonic acid	9.93080419	22.7897733	1.322118936	2a
4-Aminobenzoic acid	0.18050498	7.526836591	0.024349682	3
4-Nitroaniline	1.80515061	6.203632903	0.096778358	3
2-Naphthylamine	1.28333022	12.30090211	0.50951536	2a
methyl hygrate betaine	5.34401524	6.202195861	1.440574161	3
Xanthine	12.53946	18.66316194	1.929685711	3
Indole-3-acetaldehyde	0.89838167	23.07800539	0.101811706	3
L-Tyrosine	11.3882319	20.55267311	1.654880265	2a
N-[2-(1H-Imidazol-4-yl)ethyl]-3-methylbutanamide	0.99401636	17.4172973	0.301449747	3
pantothenic acid	40.1282393	41.20168166	5.949096282	3
3-(1H-Imidazol-4-yl)-2-oxopropyl dihydrogen phosphate	28.9040274	46.90243385	16.82604716	3

Kappaxan (VAN)	3.171299	15.19171084	0.257664189	3
Malic Acid	3.70181522	12.1302813	0.346614646	3
DErySphinganine	274.226158	73.12773374	116.4672142	3
loganate	4.67341132	7.273369919	0.528197287	3
oleandolide	1.85414504	7.554859747	0.08346722	3

Supplementary Table S2. Standard deviations of peak area of faecal metabolites in ES+ in CHF, controls and quality controls and levels of identification confidence. ES+ = positive ion mode; SD = standard deviations.

Faecal Metabolites in ES-	SD of peak area of CHF	SD of peak area of controls	SD of peak area of quality controls	Levels of identification confidence
1-Amino-1-carboxycyclopropane	0.054983	1.9021	0.018939	3
Malonic acid	0.047575	3.20977	0.003148	3
D(-)-β-hydroxy butyric acid	5.582775	11.78904	1.629368	2a
DL-Glyceric acid	0.423858	0.883891	0.018882	3
4-Methylphenol	1.015929	4.269853	0.0512	3
catechol	0.347431	2.94485	0.065425	3
Uracil	4.007624	9.169282	0.181617	2a
4-hyroxy-5-methyl-3-furanone	2.394327	1.438518	0.236338	3
Indole	0.116758	5.07161	0.030302	3
L-(+)-Valine	0.490837	1.655227	0.11391	3
L-(-)-Threonine	1.689007	2.497372	0.055989	3
Acetophenone	48.93493	105.2004	2.396735	3
Benzoic acid	3.262594	7.393557	0.152258	3
(±)-1-phenylethanol	0.905904	9.476939	0.051328	3
Guaiacol	1.110491	20.49473	0.80934	3
Thymine	3.157628	10.03237	0.179448	3
L-Pyroglutamic acid	2.505574	4.058462	0.150723	3
(Hydroxyethyl)methacrylate	1.332103	5.861122	0.045829	3
L-(+)-Leucine	9.592135	37.40501	1.496902	2a
Glutaric acid	8.666406	12.342	1.475701	3
N-lactoylethanolamine	0.063386	0.831746	0.010117	3
Malic Acid	0.837615	3.064	0.027227	2a

2,3,4-Trihydroxybutanoic acid	0.631962	1.464607	0.023138	3
Hypoxanthin	3.020409	7.991747	0.168233	2a
Phenylacetic acid	0.049098	2.79152	0.004095	3
Tyramine	0.729462	2.658039	0.107347	3
Salicylic acid	2.028339	3.293071	0.069571	3
Phenoxyethanol	1.637778	7.410134	0.05674	3
4-Nitrophenol	3.580187	5.865619	0.02865	3
Naphthalen-2-amine	0.098516	1.910344	0.02897	3
Adipic acid	2.67729	7.231519	0.363047	3
Cinnamic acid	0.102539	2.473145	0.096111	3
L-(-)-methionine	0.686999	1.914445	0.100025	3
S-(-)-Cathinone	0.496784	1.431141	0.056047	3
2,3,4,5-Tetrahydroxypentanal	2.063124	10.67085	0.03195	3
Xanthine	28.93958	31.68557	3.973937	2a
3-Hydroxyphenylacetic acid	0.461973	1.84158	0.044113	2a
protocatechuic acid	1.239481	1.745089	0.134331	3
(5S,6S)-6-Amino-5-hydroxy-1,3-cyclohexadiene-1-carboxylic acid	0.040555	0.834315	0.017082	3
Orotic acid	0.511	2.793358	0.016264	3
N-Acetylvaline	0.595775	0.844244	0.095257	3
3-Indolecarboxylic acid	13.43448	39.80457	0.417049	3
Diethylpyrocarbonate	7.760471	25.89518	0.270046	3
4-Oxo-4-(3-pyridinyl)butanal	0.549713	3.172873	0.083317	3
Eugenol	0.066826	0.820404	0.023569	3

(2S)-2-Amino-4-(methylsulfinyl)butanoic acid	0.306496	0.678824	0.024939	3
L-Phenylalanine	6.252693	17.21207	2.133358	2a
Terephthalic acid	4.22599	7.160103	0.303951	2a
Pentonic acid	1.336049	4.173748	0.034659	3
methylxanthine	8.945392	17.98884	1.891625	3
veratraldehyde	50.96595	97.56139	2.437134	3
2-Methoxy-5-nitrophenol	0.098485	1.242732	0.009017	3
Oxaceprol	1.807959	1.580854	0.075254	3
Suberic acid	1.399791	3.209777	0.060345	3
L-Theanine	1.521915	7.238848	0.094732	3
Formyl-L-methionine	0.780962	1.752869	0.038578	3
Aspirin	0.344574	1.288095	0.124335	3
Hexose	1.121068	4.581704	0.27331	3
DL-TYROSINE	1.063235	3.998017	0.059065	3
4-Hydroxyphthalic acid	0.523986	1.440183	0.087285	3
mephenesin	5.554306	15.64352	0.118447	3
Saccharin	7.104063	25.14509	0.552375	3
6-Acetamido-2-oxohexanoic acid	2.112588	3.106712	0.09821	3
Pivagabine	2.8354	5.074833	0.161563	3
5-Hydroxyindoleacetate	2.538798	11.81455	0.157622	3
Metirosine	1.69983	2.709303	0.124694	3
Gluconic acid	0.575892	1.205875	0.037135	3
2,4,5-Trimethoxybenzaldehyde	0.339653	2.910888	0.05877	3

5-Hydroxy-6-methyl-3,4-pyridinedicarboxylic acid	0.253151	2.667838	0.003745	3
clavulanic acid	0.26298	1.266545	0.082489	3
N-(1-Hydroxycyclopropyl)glutamine	0.910621	1.514118	0.078072	3
DL-Tryptophan	2.087788	6.234209	0.720224	3
3-(2-Formamidophenyl)-3-oxopropanoic acid	12.13405	35.97131	0.291729	3
(2E)-3-(3,4-Dimethoxyphenyl)acrylic acid	0.576954	4.730069	0.07675	3
3-(4-Hydroxy-3-methoxyphenyl)-2-oxopropanoic acid	0.073656	1.64782	0.023692	3
Sinapyl alcohol	0.61419	4.528038	0.045998	3
Isoprenaline	0.15512	1.177017	0.003158	3
Methyl laurate	0.021155	1.406096	0.003331	3
(2R,3S)-2,3,4-Trihydroxy-3-methylbutyl dihydrogen phosphate	6.774981	11.48	0.349238	3
tert-Butyl 3-amino-1-methyl-2,3-dioxopropylcarbamate	0.687966	2.86717	0.196389	3
meprobamate	2.107603	9.812129	0.12566	3
Pantothenic Acid	39.69245	50.95519	7.436999	2a
N-Acetyl-b-D-glucosamine	3.092851	9.10847	0.109944	3
L-3-hydroxykynurenine	0.020967	1.33813	0.003049	3
Methyl Jasmonate	0.044683	1.293207	0.019121	3

(4Z)-3-(Dihydroxymethylene)-7-methyl-2,6-dioxo-4-octenoic acid	1.028282	2.376017	0.045938	3
Traumatic Acid	3.583969	24.40835	0.16086	3
para-Tolyl octanoate	5.855045	0.757432	0.440286	3
1-hexadecanal	0.144588	2.118556	0.042925	3
1-(2-Deoxypentofuranosyl)-5-methyl-2,4(1H,3H)-pyrimidinedione	13.17278	48.04387	1.388647	3
p,p'-sec-Butylidenediphenol	0.053784	2.478483	0.120251	3
Pentadecanoic acid	0.400745	2.770011	0.092037	3
1-Pentofuranosyl-2,4(1H,3H)-pyrimidinedione	0.66815	1.679974	0.101316	3
2'-Deoxyinosine	7.403629	17.63314	0.295387	3
Daidzein	12.60662	36.87932	0.040307	3
Palmitelaidic acid	0.958253	1.415509	0.637692	3
Isoliquiritigenin	3.691503	18.10152	0.050668	3
Palmitic acid	1.080187	3.869032	0.339327	2a
1-(beta-D-ribofuranosyl)thymine	1.010905	2.983586	0.04504	3
Kappaxan (VAN)	7.030158	30.48541	0.29617	3
Methyl (4R)-4-[(2R)-6-methyl-4-oxo-5-hepten-2-yl]-1-cyclohexene-1-carboxylate	0.105796	3.194215	0.003825	3
threonylphenylalanine	0.898682	1.642101	0.008633	3
Methyl (2E,6E)-(10R)-10,11-epoxy-3,7,11-trimethyl-2,6-dodecadienoate	0.347123	8.713882	0.026374	3
Arabinosylhypoxanthine	11.04859	11.77697	0.212321	3

Genistein	2.731843	17.03434	0.112663	3
Arbutin	0.078432	0.60949	0.043583	3
16-hydroxypalmitic acid	0.485569	2.409965	0.067093	3
N~6~-(5-Oxo-D-isoleucyl)-L-lysine	0.682882	2.227459	0.085922	3
Phloretin	0.85398	7.264875	0.012906	3
N-(2,6-Dimethylphenyl)-2-methoxy-N-(2-oxo-1,3-oxazolidin-3-yl)acetamide	0.636801	5.909892	0.038749	3
(9E,12E,15E)-9,12,15-Octadecatrienoic acid	0.684386	6.781446	0.165737	3
9E,12E-octadecadienoic acid	4.762404	18.84906	1.523083	3
Artemisinin	5.373005	15.11253	0.072393	3
Oleic acid	0.482467	3.384283	0.119995	2a
9-Pentofuranosyl-3,9-dihydro-1H-purine-2,6-dione	1.362347	2.239534	0.136289	3
Stearic acid	5.255753	19.45677	0.962663	2a
hexadecandioic acid	0.559538	1.676469	0.102636	3
methyl 3-hydroxypalmitate	7.773478	27.38357	0.553204	3
10,16-Dihydroxyhexadecanoic acid	0.657998	2.306741	0.025205	3
8-{(1S,5S)-4-Oxo-5-[(2Z)-2-penten-1-yl]-2-cyclopenten-1-yl}octanoic acid	0.14024	5.346178	0.011502	3
13-KODE	1.160407	5.737578	0.111107	3
8-(2-Octyl-1-cyclopropen-1-yl)octanoic acid	4.077706	37.72967	0.605767	3

13S-hydroxyoctadecadienoic acid	16.41134	86.68177	2.349664	3
Ricinoleic Acid	3.504957	51.01335	1.94333	3
2-Methoxyestradiol	1.419745	2.885564	0.016768	3
6 beta hydroxy testosterone	0.930456	2.531212	0.012339	3
Arachidonic acid	1.880047	16.00287	0.863613	2a
Dihomo-gamma-linolenic acid	1.099602	4.963355	0.143921	3
Ethyl linoleate (JAN)	0.057566	2.046246	0.030048	3
(9S,10E,12Z,15Z)-9-Hydroperoxy-10,12,15-octadecatrienoic acid	1.085103	10.4863	0.131486	3
Ethyl (9E)-9-octadecenoate	0.18741	4.50543	0.04295	3
(10E,12Z)-9-Hydroperoxy-10,12-octadecadienoic acid	2.919067	6.927318	0.054452	3
6-(1,1-DIMETHYLALLYL)-2-(1-HYDROXY-1-METHYLETHYL)-2,3-DIHYDRO-7H-FURO[3,2-G]CHROMEN-7-ONE	0.025446	3.262111	0.003311	3
Dibutyl sebacate	0.920227	5.024325	0.193275	3
17-(Hydroxymethyl)-12-methyl-8-oxapentacyclo[14.2.1.0~1,13~.0~4,12~.0~5,9~]nonadeca-5(9),6-dien-17-ol	0.026015	1.744707	0.004736	3
9,10-Dihydroxyoctadecanoic acid	22.2128	43.64497	1.310922	3
15-Hydroxyeicosatetraenoic acid	0.150371	4.124629	0.026792	3
Bisoprolol	0.372722	2.18529	0.06832	3

(3Z)-5-{(1S,2R,3R,5S)-3,5-Dihydroxy-2-[(1E,3S)-3-hydroxy-1-octen-1-yl]cyclopentyl}-3-pentenoic acid	0.30054	1.308161	0.031811	3
4-Deoxy-5-C-(3,5-di-sec-butyl-1-cyclopenten-1-yl)pentonic acid	1.657274	3.454864	0.282099	3
Retinyl acetate	3.989558	117.3204	1.279092	3
gibberellin A7	0.14093	1.149674	0.003405	3
(10E)-9,12,13-Trihydroxy-10-octadecenoic acid	2.45499	11.24157	0.198985	3
Ethyl eicosapentaenoic acid	2.138718	60.63279	0.920693	3
Gibberellin A4	0.076627	1.683623	0.003265	3
15,16-Epoxy-6b,9-dihydroxy-8bH-labda-13(16),14-dien-19-oic Acid g-Lactone	0.789406	2.901869	0.056426	3
9,10,18-Trihydroxystearic Acid	15.75634	84.04333	0.650007	3
Ganaxolone	1.866637	12.32644	0.797774	3
Dihexyl phthalate	1.206263	1.362401	0.068592	3
Leukotriene B4	1.807474	7.222422	0.090975	3
(1S,2S,5R,6R,7R,10S,12S,13R)-6,13-Bis(hydroxymethyl)-2,6-dimethyltetracyclo[10.3.1.0~1,10~.0~2,7~]hexadecane-5,13-diol	0.150104	1.607429	0.045967	3
Erucic acid	0.208306	5.708015	0.038242	3

(1R,2S,3S,4R,5S,6R)-2-Amino-3,4,5,6-tetrahydroxycyclohexyl 2-amino-2-deoxy-alpha-D-glucopyranoside	1.420052	1.370223	0.033783	3
behenic acid	0.027869	1.353081	0.006753	3
(+)-Gibberellic acid	0.249972	1.459311	0.003798	3
7,11,12-Trihydroxy-6,20-epoxyabieta-8(14),9(11),12-trien-20-one	0.144037	1.56147	0.019722	3
gibberellin A1	0.488041	1.827383	0.128956	3
(+)-Ingenol	0.139363	1.761334	0.026846	3
Dinoprostone	0.027933	0.722426	0.00274	3
22-Oxodocosanoic acid	0.435385	4.6902	0.038708	3
22-Hydroxydocosanoic acid	3.918501	35.27181	2.318513	3
(1alpha,2beta,4bbeta,10beta)-2,7-Dihydroxy-1-methyl-8-methylene-3-oxogibb-4-ene-1,10-dicarboxylic acid	0.937716	2.153582	0.122769	3
gibberellin A8	5.104804	7.161676	0.281147	3
4beta-Phorbol	8.3032	17.64728	0.066107	3
.beta.-Butoxyethyl phthalate	1.862062	12.55784	0.002674	3
Nervonic acid	18.27977	119.19	17.99766	3
Dehydroepiandrosterone sulfate	47.12133	219.0763	15.92678	3

(11alpha,13E,15S)-11,15-Dihydroxy-6,9-dioxoprost-13-en-1-oic acid	1.704873	9.146622	0.050446	3
Lignoceric acid	0.349784	20.35675	0.168477	3
DEHA	0.301058	1.11153	0.238181	3
Lithocholic acid	82.66116	463.3883	26.10059	3
Deoxycholic acid	117.0864	320.9515	108.0849	2a
Pregnenolone sulfate	12.679	36.64396	0.089101	3
L-Palmitoylcarnitine	2.908473	9.33825	1.22669	3
cholic acid	27.22299	188.766	0.664587	2a
Calcitriol	1.1423	3.968417	0.596314	3
Calcifediol hydrate	1.880805	6.177465	0.372563	3
(2E,4E,8E,10E,12R,13R,14E)-7,13-Dihydroxy-N-[(2S)-1-hydroxy-2-propanyl]-2,10,12,14,16-pentamethyl-2,4,8,10,14-heptadecapentaenamide	1.053122	10.68415	0.206821	3
chitobiose, di-N-acetyl	0.09628	2.047	0.031325	3
3b,4b,7a,12a-Tetrahydroxy-5b-cholanoic acid	0.692571	1.622796	0.104782	3
gitogenin	0.201543	1.671709	0.103623	3
Prunin	2.333661	15.61824	0.073833	3
(1S,3R,5Z,7E,24R)-9,10-Secocholesta-5,7,10-triene-1,3,24-triol hydrate (1:1)	0.592882	4.063045	0.153886	3

5b-Cholestane-3a,7a,12a,26-tetrol	0.26327	3.480273	0.127571	3
Reomol DiDP	8.472091	16.6758	0.677979	3
16-{[(2E)-3-(4-Hydroxy-3-methoxyphenyl)-2-propenoyl]oxy}hexadecanoic acid	0.084703	1.077525	0.051731	3
(25R)-5a-Spirostan-2a,3b,15b-triol	2.019159	13.1765	0.192487	3
(3alpha,5alpha,14xi,22R,23R,24S)-3,22,23-Trihydroxyergostan-6-one	0.106071	2.047549	0.073252	3
(3alpha,5beta,7alpha,12alpha,25R)-3,7,12-Trihydroxycholestan-26-oic acid	2.30294	8.402231	0.51762	3
Ecdysone	0.679485	3.447174	0.055191	3
(3alpha,5beta,7alpha,12alpha)-3,7,12-Trihydroxycholestane-5-carboxylic acid	0.557435	2.445105	0.55451	3
(1S,4R,5'S,6'R,9S,10E,12E,14S,15S,16E,19R,21S)-9,15-Dihydroxy-6'-isopropyl-5',6,10,14,16-pentamethyl-5',6'-dihydro-3H,7H-spiro[2,20-dioxatricyclo[17.3.1.0~4,9~]tricosa-5,10,12,16-tetraene-21,2'-pyran]-3,7-dione	0.362259	27.94493	0.232328	3

Supplementary Table S3. Standard deviations of peak area of faecal metabolites in ES- in CHF, controls and quality controls and levels of identification confidence. ES- = negative ion mode; SD = standard deviations.

Plasma Metabolites in ES+	SD of peak area of CHF	SD of peak area of controls	SD of peak area of quality controls	Levels of identification confidence
Indole	5.091668179	0.31358575	1.231854257	2a
Benzoic acid	0.36137326	3.677962686	0.157552471	3
L-(+)-Leucine	189.7515243	509.8845513	116.7926458	2a
Naphthalen-2-amine	2.747160345	0.516340504	1.311271384	3
DL-Lysine	2.600391578	3.288871693	0.225184947	3
L-Histidine	0.531959156	2.695016285	0.263883875	2a
(-)-L-Carnitine	42.62881105	11.9231736	39.12251382	2a
O-(Carbamoylamino)-D-serine	7.263766761	0	0.91636004	3
L-Phenylalanine	195.129888	196.94284	26.73816848	2a
Uric Acid	10.69926356	4.714414664	0.792969298	3
3,4-dihydroxyphenylacetic acid	7.560670109	0.040616252	0.931328138	3
Indole-3-acetic acid	3.475977582	0.024838989	0.99285494	3
Glufosinate	15.90324099	1.828966751	3.303718008	3
4-amino-2-hydroxyamino-6-nitrotoluene	1.562405311	7.475609775	2.06692036	3
2-Oxo-3-(phosphonooxy)propanoic acid	7.740110692	0	4.406247037	3
ibufenac	0.062034714	1.062851001	0.010761341	3
2,4,5-Trimethoxybenzaldehyde	1.543511708	0.032932721	0.188750586	3
L-Tryptophan	131.206092	0.020190778	155.9456998	2a
5-Methoxy-3-indoleaceate	0.051175018	2.453140546	0.277509621	3
3-Carboxy-3-deoxypent-2-ulosaric acid	0.64688049	1.417371192	0.167386253	3

3-C-Carboxy-2,4-dideoxy-2-methylpentaric acid	0.493532573	1.120562711	0.068405568	3
4-(Phosphonooxy)-L-threonine	12.0109056	0	4.570803643	3
3-(1H-Imidazol-4-yl)-2-oxopropyl dihydrogen phosphate	38.5789304	0	54.49347132	3
2-Hydroxy-2-[3-(methylsulfanyl)propyl]succinic acid	0.046711076	4.488123176	0.007615758	3
Diethyl phthalate	0.043328401	86.02957541	0.006670288	3
Bufexamac	0.537844551	1.386463946	0.301952632	3
5-acetamido-6-formamido-3-methyluracil	7.921805829	0.038593922	2.573227066	3
thr-asp	13.99821102	30.00564969	8.203563944	3
Trolox	0.041905885	28.61084895	0.007843152	3
(±)-(2E)-Abscisic acid	4.096227829	0.03070927	0.365488047	3
Alphalin	26.9699649	0.068122012	22.92226445	3
Methyl alpha-aspartylphenylalaninate	1.627629226	0.020506125	0.176013691	3
8-(2-Octyl-1-cyclopropen-1-yl)octanoic acid	5.145773212	0.04091973	2.430273024	3
Pregnane-3,20-dione	2.174096333	0.030737634	0.737090596	3
Phytosphingosine	0.043659571	5.781579905	0.006957989	3
Allopregnanolone	25.14410136	0.031911598	22.37584946	3
Retinyl acetate	2.355181357	0.053420002	0.248097314	3
Corticosterone	0.04073509	0.768922126	0.007565244	3
Cortisol	4.252243627	0.024040334	0.898398772	3
Sphingosine 1-phosphate	18.59263432	0.033461634	2.095033636	2a

L-Palmitoylcarnitine	14.38690914	0.026313668	1.203033166	2a
Hecogenin	158.4291155	0.093289658	71.55933042	3
(3beta,7beta,22E,24xi)-Ergosta-5,22-diene-3,7,8-triol	12.15355335	0.083738105	3.162408303	3
5-(3-Hydroxy-3,7,11,15-tetramethylhexadecyl)-2,3-dimethyl-1,4-benzoquinone	2.858776162	0.033285204	0.762914918	3
4,6-Diamino-3-({3-amino-6-[1-(methylamino)ethyl]tetrahydro-2H-pyran-2-yl}oxy)-2-hydroxycyclohexyl 3-deoxy-4-C-methyl-3-(methylamino)pentopyranoside	0.065062344	1.344769899	0.028503828	3

Supplementary Table S4. Standard deviations of peak area of plasma metabolites in ES+ in CHF, controls and quality controls and levels of identification confidence. ES+ = positive ion mode; SD = standard deviations.

Plasma Metabolites in ES-	SD of peak area of CHF	SD of peak area of controls	SD of peak area of quality controls	Levels of identification confidence
2-Furoic acid	4.252728	8.576265	2.303222	3
Levulinic acid	15.63143	0.609709	16.96087	3
Ethyl lactate	34.79379	0.156649	12.01488	3
Acetophenone	0.126512	4.628731	0.009909	3
Itaconic acid	6.904364	9.495895	1.907848	3
(Hydroxyethyl)methacrylate	1.327088	125.6866	0.656351	3
Paraldehyde	4.104297	0.123308	0.477366	3
Cinnamyl alcohol	0.115675	4.743259	0.009098	3
alpha-Ketoglutaric acid	49.88289	106.2348	12.02445	3
(+/-)-Camphor	0.102844	4.806871	0.052158	3
Gentisic acid	16.84532	0.051114	1.489846	2a
Ethyl 3-oxohexanoate	7.214117	0.097858	0.647443	3
2-Hydroxyoctanoic acid	0.144539	3.967169	0.010076	3
4-Oxo-4-(3-pyridinyl)butanal	261.3925	0.09852	28.63135	3
L-Phenylalanine	28.46867	7.858324	10.83313	2a
Phenyllactic acid	0.099126	4.412917	0.039106	2a
Uric Acid	41.08673	51.25232	30.75761	3
5-(1,2-DIHYDROXYETHYL)-2,3,4(5H)-FURANTRIONE	4.695947	10.9778	1.780929	3
3-METHYLORSELLINIC ACID	15.12212	0.059182	6.01869	2a
3,4-Dihydroxyphenylglycolic acid	18.3874	0.043957	0.999784	3

1,3,4-Trihydroxy-5-oxocyclohexanecarboxylic acid	5.000965	0.708143	0.692027	3
Citric acid	45.62422	112.4109	17.24363	3
ibufenac	0.112525	18.12861	0.020005	3
Metirosine	0.191984	8.864308	0.161513	3
DL-Tryptophan	31.57286	0.044291	4.943489	3
5-Methoxy-3-indoleaceate	0.105982	9.425475	0.005455	3
(2R,3S)-2,3,4-Trihydroxy-3-methylbutyl dihydrogen phosphate	323.7802	6.080291	276.6683	3
Methyl Jasmonate	0.096132	10.93454	0.008217	3
para-Tolyl octanoate	0.121149	2.765351	0.074488	3
2-Hydroxy-2-phenyl-1,3-propanediyl dicarbamate	8.722828	0.051078	0.460917	3
Palmitic acid	0.446511	21.84774	0.585407	2a
16-hydroxypalmitic acid	17.38348	0.107092	3.070073	3
9E,12E-octadecadienoic acid	0.869691	7.860687	0.26896	3
Oleic acid	0.401201	4.873842	0.305882	2a
Stearic acid	0.12932	7.605594	0.231849	2a
2-Amino-6-[(3-carboxypropanoyl)amino]heptanedioic acid	7.131939	0.05172	0.620208	3
(3S,4R)-3,4,5-Trihydroxy-4'-oxo-3',4,4',5-tetrahydro-2'H,3H-spiro[furan-2,1'-naphthalene]-6'-carboxylic acid	8.318284	0.437776	0.373439	3

2,5-Dihydroxy-3-undecyl-1,4-benzoquinone	73.25762	0.05071	0.523242	3
13S-hydroxyoctadecadienoic acid	0.093383	3.163011	0.02298	3
Ricinoleic Acid	0.156676	5.900793	0.290247	3
8,11-eicosadiynoic acid	0.097127	4.397781	0.00554	2a
(9S,10E,12Z,15Z)-9-Hydroperoxy-10,12,15-octadecatrienoic acid	15.8423	0.048762	3.134864	3
(10E,12Z)-9-Hydroperoxy-10,12-octadecadienoic acid	26.68743	0.055825	1.932357	3
9,10-Dihydroxyoctadecanoic acid	6.51426	0.062288	2.17624	3
Adipostatin A	0.11025	2.666365	0.008899	3
(4Z,7Z,10Z,13Z,16Z,19Z)-4,7,10,13,16,19-Docosahexaenoic acid	9.129792	0.053654	0.419853	2a
Protirelin	11.35051	0.052301	0.072825	3
Dehydroepiandrosterone sulfate	0.120479	7.517062	0.005787	3
(3R,4S,5R,6S,7S,9R,11R,12S,13R,14R)-4,6,12-Trihydroxy-3,5,7,9,11,13,14-heptamethyloxacyclotetradecane-2,10-dione	9.835905	0.058224	7.569579	3
2-arachidonoylglycerol	0.112432	3.401217	0.007598	3
Sphingosine 1-phosphate	43.94685	0.056277	6.010662	3
2-Amino-3-hydroxyoctadecyl dihydrogen phosphate	0.131242	13.64691	0.009186	3
Deoxycholic acid	0.188833	9.19659	0.214312	3

Pregnenolone sulfate	0.121962	25.84439	0.006259	3
Gefarnate	0.126634	6.240233	0.007142	3
cholic acid	0.091247	2.243938	0.027726	3
Hecogenin	16.87939	0.047165	1.58048	3
gitogenin	0.113708	5.655858	0.009348	3
(1S,3R,5Z,7E,24R)-9,10-Secocholesta-5,7,10-triene-1,3,24-triol hydrate (1:1)	0.11414	4.37812	0.006633	3
5b-Cholestane-3a,7a,12a,26-tetrol	0.154617	8.612412	0.012224	3
Reomol DiDP	0.096987	69.79652	0.006977	3
16-{[(2E)-3-(4-Hydroxy-3-methoxyphenyl)-2-propenoyl]oxy}hexadecanoic acid	0.09605	7.152311	0.007252	3
(3beta,5alpha,14xi,22R,23R,24S)-3,22,23-Trihydroxyergostan-6-one	0.133565	12.6916	0.076996	3
(2alpha,3alpha,5alpha,22R,23R,24S)-Ergostane-2,3,22,23-tetrol	0.126466	28.35025	0.007426	3
Ecdysone	0.233562	8.593969	0.048481	3
(3alpha,5alpha)-17-Oxoandrostan-3-yl beta-D-glucopyranosiduronic acid	0.105647	4.549983	0.006085	3
10-(3,4-Dihydroxy-5,6-dimethyl-2-heptanyl)-5,6-dihydroxy-7a,9a-dimethylhexadecahydro-3H-benzo[c]indeno[5,4-e]oxepin-3-one	0.098354	2.319793	0.005588	3
Azelnidipine	0.099933	5.592633	0.004976	3

4E,15Z-Bilirubin IXa	0.096899	40.91702	0.006487	3

Supplementary Table S5. Standard deviations of peak area of plasma metabolites in ES- in CHF, controls and quality controls and levels of identification confidence. ES- = negative ion mode; SD = standard deviations.

Faecal metabolites vs. Microbial genera	SCC index	p value	q value
Hypoxanthin-vs-Prevotella	0.172799	0.162009	0.295062
Hypoxanthin-vs-Alistipes	0.400511	0.000784	0.004863
Hypoxanthin-vs-Faecalibacterium	0.508301	1.13E-05	0.00021
Hypoxanthin-vs-Oscillibacter	0.425613	0.00033	0.002448
Hypoxanthin-vs-Subdoligranulum	0.290167	0.017223	0.053619
Hypoxanthin-vs-Bilophila	0.304414	0.012259	0.041418
Hypoxanthin-vs-Anaerotruncus	0.438183	0.000208	0.001658
Hypoxanthin-vs-Butyricicoccus	0.502674	1.46E-05	0.000248
Hypoxanthin-vs-Paraprevotella	0.259997	0.033603	0.089818
Hypoxanthin-vs-Collinsella	0.298867	0.014021	0.045343
Hypoxanthin-vs-Parasutterella	0.140115	0.258099	0.418121
Hypoxanthin-vs-Pyramidobacter	0.302299	0.012907	0.042829
Hypoxanthin-vs-Intestinimonas	0.349868	0.003705	0.016034
Hypoxanthin-vs-Pseudoflavonifractor	0.358369	0.002904	0.013278
Hypoxanthin-vs-Holdemania	0.40933	0.000583	0.00378
Hypoxanthin-vs-Oribacterium	0.362399	0.002581	0.012099
Hypoxanthin-vs-Bacteroides	-0.14243	0.250239	0.408729
Hypoxanthin-vs-Eubacterium	0.124751	0.314489	0.474814
Hypoxanthin-vs-Veillonella	-0.30852	0.01108	0.038165
Hypoxanthin-vs-Lactobacillus	-0.11737	0.344204	0.50493
Hypoxanthin-vs-Agathobacter	0.033961	0.784983	0.863003
Hypoxanthin-vs-Enterococcus	-0.20648	0.093654	0.199389
Hypoxanthin-vs-Blautia	-0.10907	0.379631	0.538925
Hypoxanthin-vs-Streptococcus	-0.23733	0.053139	0.127144

Hypoxanthin-vs-Erysipelatoclostridium	-0.28382	0.019936	0.060057
Hypoxanthin-vs-Lachnospira	0.032405	0.794616	0.869763
Hypoxanthin-vs-Fusobacterium	-0.05284	0.671091	0.777104
Hypoxanthin-vs-Bacillus	0.025301	0.838951	0.897975
Hypoxanthin-vs-Dorea	-0.04529	0.715884	0.816081
Hypoxanthin-vs-Tyzzerella	0.030449	0.806762	0.8758
Hypoxanthin-vs-Butyrivibrio	0.171482	0.165281	0.299486
Hypoxanthin-vs-Mycoplasma	-0.09414	0.448591	0.600189
Hypoxanthin-vs-Coprobacillus	-0.31694	0.008969	0.032319
Hypoxanthin-vs-Selenomonas	0.092545	0.456356	0.607219
Hypoxanthin-vs-Anaerostipes	-0.12715	0.305217	0.466873
Hypoxanthin-vs-Peptoclostridium	0.074507	0.549024	0.681071
Hypoxanthin-vs-Dysgonomonas	-0.09713	0.434232	0.58682
Hypoxanthin-vs-Coprobacter	0.06465	0.603225	0.726694
Hypoxanthin-vs-Capnocytophaga	-0.12216	0.324736	0.485799
Hypoxanthin-vs-Campylobacter	-0.14327	0.247434	0.405516
Hypoxanthin-vs-Flavobacterium	-0.04673	0.707273	0.808933
Oleic acid-vs-Prevotella	0.110743	0.372307	0.532275
Oleic acid-vs-Alistipes	0.190757	0.12204	0.240794
Oleic acid-vs-Faecalibacterium	0.371418	0.001972	0.009837
Oleic acid-vs-Oscillibacter	0.300383	0.013519	0.044252
Oleic acid-vs-Subdoligranulum	0.120042	0.333246	0.494251
Oleic acid-vs-Bilophila	0.121438	0.327611	0.487925
Oleic acid-vs-Anaerotruncus	0.112818	0.363358	0.523816
Oleic acid-vs-Butyricicoccus	0.381874	0.001429	0.007597

Oleic acid-vs-Paraprevotella	0.139676	0.259608	0.420062
Oleic acid-vs-Collinsella	0.389337	0.001128	0.006348
Oleic acid-vs-Parasutterella	0.378961	0.001565	0.00818
Oleic acid-vs-Pyramidobacter	0.331192	0.006188	0.023875
Oleic acid-vs-Intestinimonas	0.246229	0.044582	0.111064
Oleic acid-vs-Pseudoflavonifractor	0.224359	0.067964	0.155245
Oleic acid-vs-Holdemania	0.205364	0.095483	0.202075
Oleic acid-vs-Oribacterium	0.201054	0.1028	0.213087
Oleic acid-vs-Bacteroides	-0.1888	0.125986	0.245576
Oleic acid-vs-Eubacterium	-0.05196	0.676251	0.78148
Oleic acid-vs-Veillonella	-0.29136	0.016748	0.052479
Oleic acid-vs-Lactobacillus	-0.32513	0.007263	0.027366
Oleic acid-vs-Agathobacter	-0.13309	0.282965	0.442751
Oleic acid-vs-Enterococcus	-0.50259	1.46E-05	0.000248
Oleic acid-vs-Blautia	-0.31355	0.009773	0.034513
Oleic acid-vs-Streptococcus	-0.41408	0.000495	0.003393
Oleic acid-vs-Erysipelatoclostridium	-0.54949	1.47E-06	0.000048
Oleic acid-vs-Lachnospira	-0.03189	0.797834	0.871584
Oleic acid-vs-Fusobacterium	-0.35789	0.002944	0.013413
Oleic acid-vs-Bacillus	-0.33227	0.006013	0.023372
Oleic acid-vs-Dorea	-0.01636	0.895445	0.937012
Oleic acid-vs-Tyzzerella	-0.14283	0.2489	0.407644
Oleic acid-vs-Butyrivibrio	-0.12615	0.309059	0.470544
Oleic acid-vs-Mycoplasma	-0.2756	0.023987	0.069075
Oleic acid-vs-Coprobacillus	-0.53029	3.92E-06	9.78E-05

Oleic acid-vs-Selenomonas	-0.21678	0.078066	0.172773
Oleic acid-vs-Anaerostipes	-0.1825	0.139372	0.263685
Oleic acid-vs-Peptoclostridium	-0.13688	0.269353	0.429792
Oleic acid-vs-Dysgonomonas	-0.29308	0.016087	0.050936
Oleic acid-vs-Coprobacter	-0.09219	0.458113	0.607219
Oleic acid-vs-Capnocytophaga	-0.21398	0.082075	0.179511
Oleic acid-vs-Campylobacter	-0.16893	0.171764	0.307548
Oleic acid-vs-Flavobacterium	-0.21929	0.074592	0.167071
Leukotriene B4-vs-Prevotella	0.162942	0.187684	0.327566
Leukotriene B4-vs-Alistipes	0.296193	0.014945	0.047695
Leukotriene B4-vs-Faecalibacterium	0.461849	8.35E-05	0.000882
Leukotriene B4-vs-Oscillibacter	0.586679	1.82E-07	1.08E-05
Leukotriene B4-vs-Subdoligranulum	0.428207	0.000301	0.002258
Leukotriene B4-vs-Bilophila	0.285019	0.019398	0.058876
Leukotriene B4-vs-Anaerotruncus	0.439899	0.000195	0.001576
Leukotriene B4-vs-Butyricicoccus	0.541903	2.18E-06	6.43E-05
Leukotriene B4-vs-Paraprevotella	0.195626	0.112625	0.226655
Leukotriene B4-vs-Collinsella	0.490901	2.47E-05	0.000369
Leukotriene B4-vs-Parasutterella	0.244034	0.046579	0.113697
Leukotriene B4-vs-Pyramidobacter	0.35342	0.003349	0.014811
Leukotriene B4-vs-Intestinimonas	0.397717	0.00086	0.00516
Leukotriene B4-vs-Pseudoflavonifractor	0.405898	0.000655	0.004135
Leukotriene B4-vs-Holdemania	0.376048	0.001712	0.008732
Leukotriene B4-vs-Oribacterium	0.281267	0.021127	0.062939
Leukotriene B4-vs-Bacteroides	-0.31906	0.008497	0.030772

Leukotriene B4-vs-Eubacterium	0.05591	0.653154	0.764013
Leukotriene B4-vs-Veillonella	-0.58855	1.62E-07	1.06E-05
Leukotriene B4-vs-Lactobacillus	-0.2481	0.042932	0.108266
Leukotriene B4-vs-Agathobacter	0.186847	0.130028	0.250229
Leukotriene B4-vs-Enterococcus	-0.38387	0.001342	0.007296
Leukotriene B4-vs-Blautia	-0.15065	0.22365	0.37466
Leukotriene B4-vs-Streptococcus	-0.30709	0.011481	0.039115
Leukotriene B4-vs-Erysipelatoclostridium	-0.26012	0.033518	0.089691
Leukotriene B4-vs-Lachnospira	0.00862	0.944806	0.966787
Leukotriene B4-vs-Fusobacterium	-0.16139	0.191992	0.332923
Leukotriene B4-vs-Bacillus	-0.05858	0.637704	0.75571
Leukotriene B4-vs-Dorea	0.064411	0.60457	0.727228
Leukotriene B4-vs-Tyzzerella	-0.00136	0.991305	0.99377
Leukotriene B4-vs-Butyrivibrio	0.152167	0.218969	0.369893
Leukotriene B4-vs-Mycoplasma	-0.06796	0.58475	0.7144
Leukotriene B4-vs-Coprobacillus	-0.09686	0.435561	0.587959
Leukotriene B4-vs-Selenomonas	-0.2074	0.092172	0.196791
Leukotriene B4-vs-Anaerostipes	0.023944	0.847481	0.903116
Leukotriene B4-vs-Peptoclostridium	0.114215	0.357409	0.517399
Leukotriene B4-vs-Dysgonomonas	-0.3035	0.012537	0.042003
Leukotriene B4-vs-Coprobacter	-0.04957	0.690396	0.794892
Leukotriene B4-vs-Capnocytophaga	-0.11386	0.358933	0.518983
Leukotriene B4-vs-Campylobacter	-0.11505	0.353869	0.514428
Leukotriene B4-vs-Flavobacterium	-0.18054	0.143734	0.268904
Xanthine-vs-Prevotella	0.163261	0.186809	0.326746

Xanthine-vs-Alistipes	0.335182	0.00556	0.021941
Xanthine-vs-Faecalibacterium	0.448041	0.000144	0.001281
Xanthine-vs-Oscillibacter	0.503472	1.41E-05	0.000245
Xanthine-vs-Subdoligranulum	0.419267	0.000413	0.002881
Xanthine-vs-Bilophila	0.309043	0.010939	0.037856
Xanthine-vs-Anaerotruncus	0.550682	1.38E-06	4.63E-05
Xanthine-vs-Butyricicoccus	0.546213	1.74E-06	5.41E-05
Xanthine-vs-Paraprevotella	0.072232	0.561321	0.692067
Xanthine-vs-Collinsella	0.380637	0.001485	0.007862
Xanthine-vs-Parasutterella	0.057387	0.644603	0.759151
Xanthine-vs-Pyramidobacter	0.310839	0.010461	0.036674
Xanthine-vs-Intestinimonas	0.496329	1.94E-05	0.000313
Xanthine-vs-Pseudoflavonifractor	0.552997	1.22E-06	4.33E-05
Xanthine-vs-Holdemania	0.497007	1.88E-05	0.000306
Xanthine-vs-Oribacterium	0.398436	0.00084	0.00509
Xanthine-vs-Bacteroides	-0.27584	0.023859	0.069017
Xanthine-vs-Eubacterium	0.175792	0.154751	0.284023
Xanthine-vs-Veillonella	-0.38451	0.001316	0.007183
Xanthine-vs-Lactobacillus	0.043499	0.726699	0.823348
Xanthine-vs-Agathobacter	0.002993	0.980822	0.987764
Xanthine-vs-Enterococcus	-0.02111	0.86535	0.915296
Xanthine-vs-Blautia	0.028813	0.816961	0.882639
Xanthine-vs-Streptococcus	-0.1228	0.322194	0.482892
Xanthine-vs-Erysipelatoclostridium	-0.08899	0.473894	0.618645
Xanthine-vs-Lachnospira	-0.09243	0.456941	0.607219

Xanthine-vs-Fusobacterium	-0.00658	0.957824	0.974748
Xanthine-vs-Bacillus	0.226953	0.064759	0.149478
Xanthine-vs-Dorea	0.023266	0.851753	0.905536
Xanthine-vs-Tyzzerella	0.045973	0.711813	0.812589
Xanthine-vs-Butyrivibrio	0.232261	0.058582	0.137051
Xanthine-vs-Mycoplasma	0.181978	0.14052	0.264733
Xanthine-vs-Coprobacillus	-0.25932	0.034085	0.090706
Xanthine-vs-Selenomonas	0.18964	0.124283	0.243631
Xanthine-vs-Anaerostipes	-0.02382	0.848235	0.903116
Xanthine-vs-Peptoclostridium	0.20265	0.10004	0.208799
Xanthine-vs-Dysgonomonas	-0.02969	0.811485	0.878291
Xanthine-vs-Coprobacter	0.032924	0.791401	0.866636
Xanthine-vs-Capnocytophaga	-0.16701	0.176747	0.314376
Xanthine-vs-Campylobacter	-0.02418	0.845975	0.903116
Xanthine-vs-Flavobacterium	-0.00375	0.975965	0.985334
Arabinosylhypoxanthine-vs-Prevotella	0.152167	0.218969	0.369893
Arabinosylhypoxanthine-vs-Alistipes	0.395722	0.000918	0.005428
Arabinosylhypoxanthine-vs-Faecalibacterium	0.4051	0.000673	0.004215
Arabinosylhypoxanthine-vs-Oscillibacter	0.507143	1.19E-05	0.000217
Arabinosylhypoxanthine-vs-Subdoligranulum	0.340769	0.004775	0.019512
Arabinosylhypoxanthine-vs-Bilophila	0.378841	0.001571	0.00818
Arabinosylhypoxanthine-vs-Anaerotruncus	0.550802	1.37E-06	4.63E-05
Arabinosylhypoxanthine-vs-Butyricicoccus	0.520911	6.21E-06	0.000132
Arabinosylhypoxanthine-vs-Paraprevotella	0.199777	0.105049	0.216267
Arabinosylhypoxanthine-vs-Collinsella	0.3966	0.000892	0.0053

Arabinosylhypoxanthine-vs-Parasutterella	-0.03081	0.804528	0.875238
Arabinosylhypoxanthine-vs-Pyramidobacter	0.248942	0.042211	0.107143
Arabinosylhypoxanthine-vs-Intestinimonas	0.408492	0.0006	0.003868
Arabinosylhypoxanthine-vs-Pseudoflavonifractor	0.457858	9.79E-05	0.000991
Arabinosylhypoxanthine-vs-Holdemania	0.522228	5.83E-06	0.00013
Arabinosylhypoxanthine-vs-Oribacterium	0.337697	0.005194	0.02094
Arabinosylhypoxanthine-vs-Bacteroides	-0.1969	0.11025	0.22355
Arabinosylhypoxanthine-vs-Eubacterium	0.078219	0.529243	0.664368
Arabinosylhypoxanthine-vs-Veillonella	-0.45083	0.000129	0.001186
Arabinosylhypoxanthine-vs-Lactobacillus	-0.06397	0.60704	0.729111
Arabinosylhypoxanthine-vs-Agathobacter	0.036795	0.767528	0.851166
Arabinosylhypoxanthine-vs-Enterococcus	-0.07483	0.547308	0.679989
Arabinosylhypoxanthine-vs-Blautia	0.080932	0.515007	0.652174
Arabinosylhypoxanthine-vs-Streptococcus	-0.18238	0.139637	0.263685
Arabinosylhypoxanthine-vs-Erysipelatoclostridium	-0.02666	0.830439	0.892418
Arabinosylhypoxanthine-vs-Lachnospira	-0.06114	0.623087	0.742852
Arabinosylhypoxanthine-vs-Fusobacterium	-0.02466	0.842963	0.901073
Arabinosylhypoxanthine-vs-Bacillus	0.197222	0.109663	0.222545
Arabinosylhypoxanthine-vs-Dorea	0.045215	0.716364	0.816243
Arabinosylhypoxanthine-vs-Tyzzerella	0.100966	0.416236	0.568535
Arabinosylhypoxanthine-vs-Butyrivibrio	0.196823	0.110397	0.223623
Arabinosylhypoxanthine-vs-Mycoplasma	0.187804	0.128036	0.248173
Arabinosylhypoxanthine-vs-Coprobacillus	-0.10891	0.380333	0.539289
Arabinosylhypoxanthine-vs-Selenomonas	0.160148	0.195466	0.337497
Arabinosylhypoxanthine-vs-Anaerostipes	-0.00216	0.986191	0.991108

Arabinosylhypoxanthine-vs-Peptoclostridium	0.189959	0.123639	0.242919
Arabinosylhypoxanthine-vs-Dysgonomonas	-0.14023	0.257688	0.417793
Arabinosylhypoxanthine-vs-Coprobacter	0.053197	0.668985	0.77605
Arabinosylhypoxanthine-vs-Capnocytophaga	-0.20465	0.096673	0.204237
Arabinosylhypoxanthine-vs-Campylobacter	-0.09027	0.467547	0.613671
Arabinosylhypoxanthine-vs-Flavobacterium	-0.01628	0.895952	0.937012
Traumatic Acid-vs-Prevotella	0.084284	0.497694	0.638608
Traumatic Acid-vs-Alistipes	0.39229	0.001026	0.005924
Traumatic Acid-vs-Faecalibacterium	0.529172	4.15E-06	0.000102
Traumatic Acid-vs-Oscillibacter	0.617288	2.64E-08	3.2E-06
Traumatic Acid-vs-Subdoligranulum	0.444289	0.000166	0.001403
Traumatic Acid-vs-Bilophila	0.451433	0.000126	0.001172
Traumatic Acid-vs-Anaerotruncus	0.515724	7.96E-06	0.000163
Traumatic Acid-vs-Butyricicoccus	0.571873	4.3E-07	0.00002
Traumatic Acid-vs-Paraprevotella	0.299505	0.013808	0.044954
Traumatic Acid-vs-Collinsella	0.327839	0.006764	0.025847
Traumatic Acid-vs-Parasutterella	0.165496	0.180766	0.320581
Traumatic Acid-vs-Pyramidobacter	0.463445	7.83E-05	0.000857
Traumatic Acid-vs-Intestinimonas	0.507303	1.18E-05	0.000217
Traumatic Acid-vs-Pseudoflavonifractor	0.500758	1.59E-05	0.000267
Traumatic Acid-vs-Holdemania	0.570077	4.76E-07	0.000021
Traumatic Acid-vs-Oribacterium	0.456261	0.000104	0.001017
Traumatic Acid-vs-Bacteroides	-0.04984	0.688741	0.79412
Traumatic Acid-vs-Eubacterium	0.187844	0.127954	0.248173
Traumatic Acid-vs-Veillonella	-0.46045	8.83E-05	0.000915

Traumatic Acid-vs-Lactobacillus	-0.10751	0.386509	0.544535
Traumatic Acid-vs-Agathobacter	0.124751	0.314489	0.474814
Traumatic Acid-vs-Enterococcus	-0.23358	0.057126	0.134266
Traumatic Acid-vs-Blautia	-0.11829	0.340417	0.502116
Traumatic Acid-vs-Streptococcus	-0.25217	0.039523	0.102144
Traumatic Acid-vs-Erysipelatoclostridium	-0.19678	0.110471	0.223623
Traumatic Acid-vs-Lachnospira	0.103001	0.406854	0.559193
Traumatic Acid-vs-Fusobacterium	-0.09103	0.4638	0.610742
Traumatic Acid-vs-Bacillus	0.105435	0.395795	0.551832
Traumatic Acid-vs-Dorea	0.113776	0.359272	0.519164
Traumatic Acid-vs-Tyzzerella	-0.05535	0.656401	0.766329
Traumatic Acid-vs-Butyrivibrio	0.243316	0.047248	0.114867
Traumatic Acid-vs-Mycoplasma	0.089911	0.469327	0.61534
Traumatic Acid-vs-Coprobacillus	-0.16893	0.171764	0.307548
Traumatic Acid-vs-Selenomonas	0.056469	0.649913	0.762435
Traumatic Acid-vs-Anaerostipes	0.057307	0.645064	0.759324
Traumatic Acid-vs-Peptoclostridium	0.073829	0.552678	0.68455
Traumatic Acid-vs-Dysgonomonas	-0.06872	0.580558	0.711794
Traumatic Acid-vs-Coprobacter	0.216937	0.077841	0.172434
Traumatic Acid-vs-Capnocytophaga	-0.04458	0.720204	0.819846
Traumatic Acid-vs-Campylobacter	0.149294	0.227896	0.37967
Traumatic Acid-vs-Flavobacterium	0.06026	0.628096	0.748086
Pentonic acid-vs-Prevotella	0.187485	0.128698	0.248856
Pentonic acid-vs-Alistipes	-0.03125	0.801799	0.873942
Pentonic acid-vs-Faecalibacterium	0.134608	0.277466	0.437142

Pentonic acid-vs-Oscillibacter	0.056549	0.649451	0.762262
Pentonic acid-vs-Subdoligranulum	-0.10468	0.39922	0.554488
Pentonic acid-vs-Bilophila	-0.08424	0.497898	0.638608
Pentonic acid-vs-Anaerotruncus	-0.11485	0.35471	0.514967
Pentonic acid-vs-Butyricicoccus	0.220728	0.072663	0.163204
Pentonic acid-vs-Paraprevotella	-0.06964	0.575501	0.707746
Pentonic acid-vs-Collinsella	0.234416	0.056215	0.132926
Pentonic acid-vs-Parasutterella	-0.01481	0.905342	0.941136
Pentonic acid-vs-Pyramidobacter	-0.08588	0.489557	0.633283
Pentonic acid-vs-Intestinimonas	0.044976	0.717803	0.817498
Pentonic acid-vs-Pseudoflavonifractor	0.092066	0.4587	0.607258
Pentonic acid-vs-Holdemania	-0.09813	0.429504	0.582382
Pentonic acid-vs-Oribacterium	-0.16107	0.192884	0.333992
Pentonic acid-vs-Bacteroides	-0.27472	0.024458	0.070098
Pentonic acid-vs-Eubacterium	-0.32233	0.00781	0.028876
Pentonic acid-vs-Veillonella	-0.22771	0.063846	0.147652
Pentonic acid-vs-Lactobacillus	-0.37134	0.001976	0.009837
Pentonic acid-vs-Agathobacter	-0.2948	0.015449	0.049043
Pentonic acid-vs-Enterococcus	-0.27915	0.022158	0.065527
Pentonic acid-vs-Blautia	-0.31603	0.009181	0.032707
Pentonic acid-vs-Streptococcus	-0.22284	0.069895	0.15846
Pentonic acid-vs-Erysipelatoclostridium	-0.39285	0.001008	0.005839
Pentonic acid-vs-Lachnospira	-0.28398	0.019864	0.060043
Pentonic acid-vs-Fusobacterium	-0.44624	0.000154	0.001333
Pentonic acid-vs-Bacillus	-0.33223	0.006019	0.023372

Pentonic acid-vs-Dorea	-0.22133	0.071871	0.161726
Pentonic acid-vs-Tyzzerella	-0.2408	0.049653	0.119513
Pentonic acid-vs-Butyrivibrio	-0.37892	0.001567	0.00818
Pentonic acid-vs-Mycoplasma	-0.38307	0.001376	0.007382
Pentonic acid-vs-Coprobacillus	-0.50978	1.05E-05	0.0002
Pentonic acid-vs-Selenomonas	-0.34556	0.004182	0.017533
Pentonic acid-vs-Anaerostipes	-0.33522	0.005554	0.021941
Pentonic acid-vs-Peptoclostridium	-0.17787	0.149862	0.277578
Pentonic acid-vs-Dysgonomonas	-0.24543	0.0453	0.111702
Pentonic acid-vs-Coprobacter	-0.13573	0.273461	0.433087
Pentonic acid-vs-Capnocytophaga	-0.14486	0.24215	0.397936
Pentonic acid-vs-Campylobacter	-0.2762	0.02367	0.068819
Pentonic acid-vs-Flavobacterium	-0.18341	0.13736	0.261221
4-Aminobenzoic acid-vs-Prevotella	0.042222	0.734422	0.82785
4-Aminobenzoic acid-vs-Alistipes	0.322212	0.007835	0.028876
4-Aminobenzoic acid-vs-Faecalibacterium	0.427089	0.000313	0.002336
4-Aminobenzoic acid-vs-Oscillibacter	0.539269	2.5E-06	7.27E-05
4-Aminobenzoic acid-vs-Subdoligranulum	0.408173	0.000606	0.00389
4-Aminobenzoic acid-vs-Bilophila	0.313872	0.009695	0.034287
4-Aminobenzoic acid-vs-Anaerotruncus	0.459095	9.32E-05	0.000951
4-Aminobenzoic acid-vs-Butyricicoccus	0.485953	3.06E-05	0.000433
4-Aminobenzoic acid-vs-Paraprevotella	0.186926	0.129861	0.250229
4-Aminobenzoic acid-vs-Collinsella	0.408333	0.000603	0.003879
4-Aminobenzoic acid-vs-Parasutterella	0.166254	0.178748	0.317468
4-Aminobenzoic acid-vs-Pyramidobacter	0.393128	0.000999	0.005807

4-Aminobenzoic acid-vs-Intestinimonas	0.452071	0.000123	0.001147
4-Aminobenzoic acid-vs-Pseudoflavonifractor	0.51273	9.16E-06	0.00018
4-Aminobenzoic acid-vs-Holdemania	0.423697	0.000353	0.002586
4-Aminobenzoic acid-vs-Oribacterium	0.289728	0.0174	0.054101
4-Aminobenzoic acid-vs-Bacteroides	-0.25365	0.038344	0.09995
4-Aminobenzoic acid-vs-Eubacterium	-0.02039	0.869891	0.918358
4-Aminobenzoic acid-vs-Veillonella	-0.39197	0.001037	0.005945
4-Aminobenzoic acid-vs-Lactobacillus	-0.13457	0.27761	0.437142
4-Aminobenzoic acid-vs-Agathobacter	0.014207	0.909153	0.944691
4-Aminobenzoic acid-vs-Enterococcus	-0.27369	0.025024	0.071385
4-Aminobenzoic acid-vs-Blautia	-0.19044	0.122677	0.241658
4-Aminobenzoic acid-vs-Streptococcus	-0.19427	0.11519	0.231241
4-Aminobenzoic acid-vs-Erysipelatoclostridium	-0.32525	0.00724	0.027352
4-Aminobenzoic acid-vs-Lachnospira	-0.10647	0.391136	0.549066
4-Aminobenzoic acid-vs-Fusobacterium	-0.29887	0.014021	0.045343
4-Aminobenzoic acid-vs-Bacillus	-0.02865	0.817958	0.883321
4-Aminobenzoic acid-vs-Dorea	-0.02542	0.838199	0.897965
4-Aminobenzoic acid-vs-Tyzzerella	-0.13748	0.267244	0.428229
4-Aminobenzoic acid-vs-Butyrivibrio	0.069559	0.57594	0.707926
4-Aminobenzoic acid-vs-Mycoplasma	-0.03069	0.805272	0.875238
4-Aminobenzoic acid-vs-Coprobacillus	-0.30932	0.010863	0.037702
4-Aminobenzoic acid-vs-Selenomonas	-0.1044	0.400487	0.554538
4-Aminobenzoic acid-vs-Anaerostipes	-0.07523	0.545167	0.678292
4-Aminobenzoic acid-vs-Peptoclostridium	0.075545	0.543458	0.677641
4-Aminobenzoic acid-vs-Dysgonomonas	-0.21414	0.081842	0.179163

4-Aminobenzoic acid-vs-Coprobacter	0.073031	0.556991	0.68778
4-Aminobenzoic acid-vs-Capnocytophaga	-0.02351	0.850245	0.904858
4-Aminobenzoic acid-vs-Campylobacter	-0.10563	0.394897	0.551533
4-Aminobenzoic acid-vs-Flavobacterium	0.08205	0.509202	0.648636
Lithocholic acid-vs-Prevotella	0.126028	0.309522	0.470544
Lithocholic acid-vs-Alistipes	0.399792	0.000803	0.004967
Lithocholic acid-vs-Faecalibacterium	0.498962	1.73E-05	0.000286
Lithocholic acid-vs-Oscillibacter	0.6639	9.13E-10	2.76E-07
Lithocholic acid-vs-Subdoligranulum	0.40937	0.000582	0.00378
Lithocholic acid-vs-Bilophila	0.324328	0.007416	0.027769
Lithocholic acid-vs-Anaerotruncus	0.582848	2.28E-07	1.24E-05
Lithocholic acid-vs-Butyricicoccus	0.45686	0.000102	0.00101
Lithocholic acid-vs-Paraprevotella	0.17244	0.162896	0.296275
Lithocholic acid-vs-Collinsella	0.386982	0.001216	0.006733
Lithocholic acid-vs-Parasutterella	0.21119	0.086246	0.18611
Lithocholic acid-vs-Pyramidobacter	0.489664	2.61E-05	0.000384
Lithocholic acid-vs-Intestinimonas	0.468792	0.000063	0.000744
Lithocholic acid-vs-Pseudoflavonifractor	0.412842	0.000517	0.003472
Lithocholic acid-vs-Holdemania	0.547051	1.67E-06	5.24E-05
Lithocholic acid-vs-Oribacterium	0.455822	0.000106	0.001031
Lithocholic acid-vs-Bacteroides	-0.20764	0.091789	0.196145
Lithocholic acid-vs-Eubacterium	0.196185	0.111581	0.224929
Lithocholic acid-vs-Veillonella	-0.41935	0.000412	0.002881
Lithocholic acid-vs-Lactobacillus	-0.04174	0.737324	0.829462
Lithocholic acid-vs-Agathobacter	0.137361	0.267665	0.428229

Lithocholic acid-vs-Enterococcus	-0.11793	0.341896	0.503006
Lithocholic acid-vs-Blautia	-0.03691	0.766793	0.851166
Lithocholic acid-vs-Streptococcus	-0.09969	0.422187	0.574392
Lithocholic acid-vs-Erysipelatoclostridium	-0.12379	0.318248	0.47846
Lithocholic acid-vs-Lachnospira	0.048847	0.694661	0.798283
Lithocholic acid-vs-Fusobacterium	0.138399	0.264033	0.42523
Lithocholic acid-vs-Bacillus	0.160069	0.195692	0.337646
Lithocholic acid-vs-Dorea	0.04298	0.729833	0.823829
Lithocholic acid-vs-Tyzzerella	0.0087	0.944295	0.966787
Lithocholic acid-vs-Butyrivibrio	0.27604	0.023754	0.068898
Lithocholic acid-vs-Mycoplasma	0.203967	0.097808	0.205916
Lithocholic acid-vs-Coprobacillus	-0.11924	0.336494	0.497238
Lithocholic acid-vs-Selenomonas	0.178187	0.14912	0.277265
Lithocholic acid-vs-Anaerostipes	0.027416	0.825691	0.889291
Lithocholic acid-vs-Peptoclostridium	0.150411	0.224395	0.375648
Lithocholic acid-vs-Dysgonomonas	-0.17316	0.161125	0.293937
Lithocholic acid-vs-Coprobacter	0.135845	0.273034	0.433087
Lithocholic acid-vs-Capnocytophaga	-0.03592	0.772925	0.854527
Lithocholic acid-vs-Campylobacter	0.024104	0.846477	0.903116
Lithocholic acid-vs-Flavobacterium	0.039189	0.752867	0.842673
4beta-Phorbol-vs-Prevotella	0.227752	0.063798	0.147652
4beta-Phorbol-vs-Alistipes	0.347833	0.003924	0.016741
4beta-Phorbol-vs-Faecalibacterium	0.517719	7.24E-06	0.00015
4beta-Phorbol-vs-Oscillibacter	0.439061	0.000202	0.001616
4beta-Phorbol-vs-Subdoligranulum	0.278434	0.022518	0.066026

4beta-Phorbol-vs-Bilophila	0.197542	0.109078	0.222478
4beta-Phorbol-vs-Anaerotruncus	0.342007	0.004615	0.018891
4beta-Phorbol-vs-Butyricicoccus	0.596456	1E-07	8.25E-06
4beta-Phorbol-vs-Paraprevotella	0.273326	0.025223	0.071614
4beta-Phorbol-vs-Collinsella	0.393407	0.00099	0.005798
4beta-Phorbol-vs-Parasutterella	0.270333	0.026932	0.074863
4beta-Phorbol-vs-Pyramidobacter	0.454306	0.000113	0.001076
4beta-Phorbol-vs-Intestinimonas	0.47418	5.05E-05	0.000631
4beta-Phorbol-vs-Pseudoflavonifractor	0.413281	0.000509	0.003453
4beta-Phorbol-vs-Holdemania	0.432676	0.000255	0.001968
4beta-Phorbol-vs-Oribacterium	0.420345	0.000398	0.002798
4beta-Phorbol-vs-Bacteroides	-0.13118	0.290013	0.450862
4beta-Phorbol-vs-Eubacterium	0.176471	0.15314	0.281707
4beta-Phorbol-vs-Veillonella	-0.32217	0.007843	0.028876
4beta-Phorbol-vs-Lactobacillus	-0.10424	0.401211	0.555157
4beta-Phorbol-vs-Agathobacter	-0.0433	0.727904	0.823348
4beta-Phorbol-vs-Enterococcus	-0.24236	0.048153	0.116365
4beta-Phorbol-vs-Blautia	-0.25034	0.041032	0.1047
4beta-Phorbol-vs-Streptococcus	-0.28594	0.018994	0.058013
4beta-Phorbol-vs-Erysipelatoclostridium	-0.3377	0.005194	0.02094
4beta-Phorbol-vs-Lachnospira	0.053396	0.667816	0.775438
4beta-Phorbol-vs-Fusobacterium	-0.20125	0.102451	0.213087
4beta-Phorbol-vs-Bacillus	0.015883	0.898489	0.939071
4beta-Phorbol-vs-Dorea	-0.00603	0.9614	0.976593
4beta-Phorbol-vs-Tyzzerella	-0.122	0.325373	0.486451

4beta-Phorbol-vs-Butyrivibrio	0.154641	0.211481	0.359502
4beta-Phorbol-vs-Mycoplasma	-0.0955	0.442049	0.594323
4beta-Phorbol-vs-Coprobacillus	-0.39796	0.000853	0.005133
4beta-Phorbol-vs-Selenomonas	0.06485	0.602105	0.726337
4beta-Phorbol-vs-Anaerostipes	-0.07307	0.556775	0.68778
4beta-Phorbol-vs-Peptoclostridium	0.112818	0.363358	0.523816
4beta-Phorbol-vs-Dysgonomonas	-0.07894	0.525455	0.660643
4beta-Phorbol-vs-Coprobacter	0.129659	0.295674	0.457251
4beta-Phorbol-vs-Capnocytophaga	-0.19375	0.116183	0.232462
4beta-Phorbol-vs-Campylobacter	-0.13389	0.280062	0.43963
4beta-Phorbol-vs-Flavobacterium	0.052398	0.673669	0.779343
Erucic acid-vs-Prevotella	0.124711	0.314645	0.474814
Erucic acid-vs-Alistipes	0.283861	0.019918	0.060057
Erucic acid-vs-Faecalibacterium	0.592984	1.24E-07	9.1E-06
Erucic acid-vs-Oscillibacter	0.492976	2.25E-05	0.000347
Erucic acid-vs-Subdoligranulum	0.343683	0.004406	0.018314
Erucic acid-vs-Bilophila	0.170285	0.168297	0.303587
Erucic acid-vs-Anaerotruncus	0.391851	0.001041	0.005945
Erucic acid-vs-Butyricicoccus	0.476135	4.65E-05	0.000595
Erucic acid-vs-Paraprevotella	0.211709	0.085459	0.184906
Erucic acid-vs-Collinsella	0.294716	0.015478	0.049071
Erucic acid-vs-Parasutterella	0.263988	0.03088	0.083814
Erucic acid-vs-Pyramidobacter	0.376526	0.001687	0.008664
Erucic acid-vs-Intestinimonas	0.391053	0.001068	0.006049
Erucic acid-vs-Pseudoflavonifractor	0.324727	0.007339	0.027553

Erucic acid-vs-Holdemania	0.411046	0.00055	0.003613
Erucic acid-vs-Oribacterium	0.378801	0.001572	0.00818
Erucic acid-vs-Bacteroides	-0.09035	0.467152	0.613485
Erucic acid-vs-Eubacterium	0.218134	0.076175	0.169363
Erucic acid-vs-Veillonella	-0.30729	0.011424	0.039017
Erucic acid-vs-Lactobacillus	-0.07104	0.567845	0.699042
Erucic acid-vs-Agathobacter	0.210671	0.087039	0.187319
Erucic acid-vs-Enterococcus	-0.12878	0.298984	0.460371
Erucic acid-vs-Blautia	-0.25932	0.034085	0.090706
Erucic acid-vs-Streptococcus	-0.04158	0.738293	0.829893
Erucic acid-vs-Erysipelatoclostridium	-0.19838	0.107553	0.220819
Erucic acid-vs-Lachnospira	0.146301	0.237462	0.392363
Erucic acid-vs-Fusobacterium	-0.08851	0.476286	0.620021
Erucic acid-vs-Bacillus	0.088874	0.474491	0.619091
Erucic acid-vs-Dorea	0.056868	0.647602	0.760461
Erucic acid-vs-Tyzzerella	-0.13936	0.26071	0.421562
Erucic acid-vs-Butyrivibrio	0.28027	0.021608	0.064136
Erucic acid-vs-Mycoplasma	0.081251	0.513345	0.651355
Erucic acid-vs-Coprobacillus	-0.2497	0.041567	0.105843
Erucic acid-vs-Selenomonas	0.182457	0.13946	0.263685
Erucic acid-vs-Anaerostipes	0.023426	0.850748	0.904995
Erucic acid-vs-Peptoclostridium	0.087038	0.483702	0.628735
Erucic acid-vs-Dysgonomonas	-0.05998	0.629693	0.748882
Erucic acid-vs-Coprobacter	0.218932	0.07508	0.1677
Erucic acid-vs-Capnocytophaga	-0.01489	0.904835	0.941012

Erucic acid-vs-Campylobacter	-0.00375	0.975965	0.985334
Erucic acid-vs-Flavobacterium	0.131774	0.287798	0.448861
Phenoxyethanol-vs-Prevotella	0.361362	0.002661	0.012354
Phenoxyethanol-vs-Alistipes	0.068122	0.583867	0.714041
Phenoxyethanol-vs-Faecalibacterium	0.537553	2.72E-06	7.57E-05
Phenoxyethanol-vs-Oscillibacter	0.195347	0.113149	0.227521
Phenoxyethanol-vs-Subdoligranulum	0.092386	0.457136	0.607219
Phenoxyethanol-vs-Bilophila	0.15496	0.210528	0.358539
Phenoxyethanol-vs-Anaerotruncus	0.026499	0.83144	0.893096
Phenoxyethanol-vs-Butyricicoccus	0.250419	0.040965	0.1047
Phenoxyethanol-vs-Paraprevotella	0.067723	0.586077	0.715659
Phenoxyethanol-vs-Collinsella	0.19407	0.115571	0.23143
Phenoxyethanol-vs-Parasutterella	0.263988	0.03088	0.083814
Phenoxyethanol-vs-Pyramidobacter	0.094022	0.449171	0.600633
Phenoxyethanol-vs-Intestinimonas	0.103759	0.40339	0.556012
Phenoxyethanol-vs-Pseudoflavonifractor	0.015604	0.900265	0.939071
Phenoxyethanol-vs-Holdemania	0.188882	0.125823	0.245576
Phenoxyethanol-vs-Oribacterium	0.134887	0.276461	0.436527
Phenoxyethanol-vs-Bacteroides	-0.09318	0.453241	0.604072
Phenoxyethanol-vs-Eubacterium	-0.00818	0.947612	0.96884
Phenoxyethanol-vs-Veillonella	-0.24479	0.045881	0.11279
Phenoxyethanol-vs-Lactobacillus	-0.27105	0.026513	0.07406
Phenoxyethanol-vs-Agathobacter	0.108149	0.383679	0.542125
Phenoxyethanol-vs-Enterococcus	-0.37888	0.001569	0.00818
Phenoxyethanol-vs-Blautia	-0.19231	0.118967	0.236817

Phenoxyethanol-vs-Streptococcus	-0.32485	0.007316	0.027524
Phenoxyethanol-vs-Erysipelatoclostridium	-0.43395	0.000244	0.001896
Phenoxyethanol-vs-Lachnospira	0.165137	0.181727	0.32205
Phenoxyethanol-vs-Fusobacterium	-0.1368	0.269635	0.429958
Phenoxyethanol-vs-Bacillus	-0.18066	0.143464	0.268606
Phenoxyethanol-vs-Dorea	0.075585	0.543244	0.677641
Phenoxyethanol-vs-Tyzzerella	-0.00547	0.964976	0.978322
Phenoxyethanol-vs-Butyrivibrio	0.026179	0.833441	0.894055
Phenoxyethanol-vs-Mycoplasma	-0.23673	0.053759	0.128122
Phenoxyethanol-vs-Coprobacillus	-0.25381	0.038218	0.099838
Phenoxyethanol-vs-Selenomonas	-0.019	0.878733	0.923796
Phenoxyethanol-vs-Anaerostipes	-0.12168	0.326651	0.487157
Phenoxyethanol-vs-Peptoclostridium	-0.0176	0.887589	0.930277
Phenoxyethanol-vs-Dysgonomonas	-0.30741	0.011391	0.038973
Phenoxyethanol-vs-Coprobacter	-0.06122	0.622633	0.742677
Phenoxyethanol-vs-Capnocytophaga	-0.22236	0.070514	0.159266
Phenoxyethanol-vs-Campylobacter	-0.11126	0.370057	0.529685
Phenoxyethanol-vs-Flavobacterium	-0.12762	0.303384	0.465368
Calcifediol hydrate-vs-Prevotella	0.150212	0.225018	0.376378
Calcifediol hydrate-vs-Alistipes	0.470668	5.84E-05	0.000699
Calcifediol hydrate-vs-Faecalibacterium	0.365552	0.002351	0.011151
Calcifediol hydrate-vs-Oscillibacter	0.629859	1.13E-08	1.82E-06
Calcifediol hydrate-vs-Subdoligranulum	0.370141	0.002049	0.010034
Calcifediol hydrate-vs-Bilophila	0.371738	0.001953	0.009799
Calcifediol hydrate-vs-Anaerotruncus	0.590111	1.48E-07	0.00001

Calcifediol hydrate-vs-Butyricicoccus	0.535039	3.09E-06	8.31E-05
Calcifediol hydrate-vs-Paraprevotella	0.176471	0.15314	0.281707
Calcifediol hydrate-vs-Collinsella	0.436148	0.000225	0.001765
Calcifediol hydrate-vs-Parasutterella	0.304095	0.012355	0.041568
Calcifediol hydrate-vs-Pyramidobacter	0.461649	8.42E-05	0.000885
Calcifediol hydrate-vs-Intestinimonas	0.561976	7.48E-07	3.02E-05
Calcifediol hydrate-vs-Pseudoflavonifractor	0.54733	1.64E-06	5.23E-05
Calcifediol hydrate-vs-Holdemania	0.450714	0.000129	0.001186
Calcifediol hydrate-vs-Oribacterium	0.2758	0.023881	0.069017
Calcifediol hydrate-vs-Bacteroides	-0.12838	0.300496	0.461524
Calcifediol hydrate-vs-Eubacterium	0.091228	0.462817	0.610038
Calcifediol hydrate-vs-Veillonella	-0.52558	4.95E-06	0.000114
Calcifediol hydrate-vs-Lactobacillus	-0.08808	0.478484	0.622287
Calcifediol hydrate-vs-Agathobacter	0.046652	0.70775	0.809096
Calcifediol hydrate-vs-Enterococcus	-0.21642	0.078572	0.173701
Calcifediol hydrate-vs-Blautia	-0.11781	0.34239	0.50312
Calcifediol hydrate-vs-Streptococcus	-0.29164	0.016639	0.052273
Calcifediol hydrate-vs-Erysipelatoclostridium	-0.14159	0.253066	0.411955
Calcifediol hydrate-vs-Lachnospira	-0.04326	0.728145	0.823348
Calcifediol hydrate-vs-Fusobacterium	-0.1459	0.238758	0.393644
Calcifediol hydrate-vs-Bacillus	0.062375	0.616059	0.736651
Calcifediol hydrate-vs-Dorea	-0.09562	0.441475	0.593953
Calcifediol hydrate-vs-Tyzzerella	-0.06908	0.578577	0.710085
Calcifediol hydrate-vs-Butyrivibrio	0.155599	0.208631	0.35616
Calcifediol hydrate-vs-Mycoplasma	0.122995	0.321403	0.482242

Calcifediol hydrate-vs-Coprobacillus	-0.16841	0.173103	0.309487
Calcifediol hydrate-vs-Selenomonas	0.119563	0.335193	0.495921
Calcifediol hydrate-vs-Anaerostipes	-0.0583	0.639311	0.756601
Calcifediol hydrate-vs-Peptoclostridium	0.112699	0.363871	0.524242
Calcifediol hydrate-vs-Dysgonomonas	-0.04829	0.697985	0.801341
Calcifediol hydrate-vs-Coprobacter	0.051161	0.680954	0.785891
Calcifediol hydrate-vs-Capnocytophaga	-0.17803	0.149491	0.277527
Calcifediol hydrate-vs-Campylobacter	-0.07307	0.556775	0.68778
Calcifediol hydrate-vs-Flavobacterium	-0.03492	0.77907	0.858574
DL-Glyceric acid-vs-Prevotella	0.127384	0.3043	0.46612
DL-Glyceric acid-vs-Alistipes	0.075465	0.543885	0.677742
DL-Glyceric acid-vs-Faecalibacterium	0.266342	0.029362	0.080712
DL-Glyceric acid-vs-Oscillibacter	0.153644	0.214478	0.363576
DL-Glyceric acid-vs-Subdoligranulum	0.11657	0.347518	0.507943
DL-Glyceric acid-vs-Bilophila	0.109705	0.376831	0.536208
DL-Glyceric acid-vs-Anaerotruncus	0.047929	0.700124	0.802547
DL-Glyceric acid-vs-Butyricicoccus	0.36631	0.002299	0.01101
DL-Glyceric acid-vs-Paraprevotella	0.074587	0.548594	0.680888
DL-Glyceric acid-vs-Collinsella	0.226794	0.064953	0.149782
DL-Glyceric acid-vs-Parasutterella	0.071993	0.562623	0.693319
DL-Glyceric acid-vs-Pyramidobacter	0.130457	0.292685	0.45385
DL-Glyceric acid-vs-Intestinimonas	0.23721	0.053262	0.127314
DL-Glyceric acid-vs-Pseudoflavonifractor	0.250579	0.040832	0.104522
DL-Glyceric acid-vs-Holdemania	0.067284	0.588513	0.716825
DL-Glyceric acid-vs-Oribacterium	-0.01113	0.928744	0.95887

DL-Glyceric acid-vs-Bacteroides	-0.24495	0.045735	0.112546
DL-Glyceric acid-vs-Eubacterium	-0.21446	0.081376	0.178791
DL-Glyceric acid-vs-Veillonella	-0.23282	0.057961	0.13586
DL-Glyceric acid-vs-Lactobacillus	-0.22284	0.069895	0.15846
DL-Glyceric acid-vs-Agathobacter	-0.15368	0.214358	0.363576
DL-Glyceric acid-vs-Enterococcus	-0.31064	0.010513	0.036804
DL-Glyceric acid-vs-Blautia	-0.29543	0.015217	0.048416
DL-Glyceric acid-vs-Streptococcus	-0.19758	0.109005	0.222478
DL-Glyceric acid-vs-Erysipelatoclostridium	-0.35889	0.00286	0.013104
DL-Glyceric acid-vs-Lachnospira	-0.2082	0.090899	0.19476
DL-Glyceric acid-vs-Fusobacterium	-0.40538	0.000666	0.004197
DL-Glyceric acid-vs-Bacillus	-0.19978	0.105049	0.216267
DL-Glyceric acid-vs-Dorea	-0.14275	0.249168	0.407805
DL-Glyceric acid-vs-Tyzzerella	-0.24	0.050437	0.12116
DL-Glyceric acid-vs-Butyrivibrio	-0.18717	0.129362	0.249542
DL-Glyceric acid-vs-Mycoplasma	-0.35095	0.003594	0.01572
DL-Glyceric acid-vs-Coprobacillus	-0.53344	3.35E-06	8.72E-05
DL-Glyceric acid-vs-Selenomonas	-0.14027	0.257551	0.417793
DL-Glyceric acid-vs-Anaerostipes	-0.208	0.091216	0.195266
DL-Glyceric acid-vs-Peptoclostridium	-0.0445	0.720684	0.820007
DL-Glyceric acid-vs-Dysgonomonas	-0.16234	0.189332	0.32973
DL-Glyceric acid-vs-Coprobacter	-0.02474	0.842461	0.900935
DL-Glyceric acid-vs-Capnocytophaga	-0.08117	0.51376	0.651355
DL-Glyceric acid-vs-Campylobacter	-0.21917	0.074754	0.167281
DL-Glyceric acid-vs-Flavobacterium	-0.17005	0.168905	0.30423

Sinapyl alcohol-vs-Prevotella	0.131415	0.289126	0.450061
Sinapyl alcohol-vs-Alistipes	0.281467	0.021032	0.062789
Sinapyl alcohol-vs-Faecalibacterium	0.365791	0.002334	0.011137
Sinapyl alcohol-vs-Oscillibacter	0.52147	6.04E-06	0.000131
Sinapyl alcohol-vs-Subdoligranulum	0.365712	0.00234	0.011142
Sinapyl alcohol-vs-Bilophila	0.192394	0.118811	0.236741
Sinapyl alcohol-vs-Anaerotruncus	0.37054	0.002025	0.009995
Sinapyl alcohol-vs-Butyricicoccus	0.478929	4.13E-05	0.000535
Sinapyl alcohol-vs-Paraprevotella	0.119802	0.334219	0.495086
Sinapyl alcohol-vs-Collinsella	0.467356	6.68E-05	0.000769
Sinapyl alcohol-vs-Parasutterella	0.256724	0.035983	0.094921
Sinapyl alcohol-vs-Pyramidobacter	0.342924	0.0045	0.018607
Sinapyl alcohol-vs-Intestinimonas	0.445087	0.000161	0.001383
Sinapyl alcohol-vs-Pseudoflavonifractor	0.458855	9.41E-05	0.000956
Sinapyl alcohol-vs-Holdemania	0.304893	0.012117	0.04105
Sinapyl alcohol-vs-Oribacterium	0.162543	0.188782	0.329008
Sinapyl alcohol-vs-Bacteroides	-0.12399	0.317463	0.477873
Sinapyl alcohol-vs-Eubacterium	0.045774	0.71301	0.813461
Sinapyl alcohol-vs-Veillonella	-0.47733	4.42E-05	0.000569
Sinapyl alcohol-vs-Lactobacillus	-0.18884	0.125904	0.245576
Sinapyl alcohol-vs-Agathobacter	0.040346	0.745812	0.837567
Sinapyl alcohol-vs-Enterococcus	-0.36918	0.002109	0.010245
Sinapyl alcohol-vs-Blautia	-0.21754	0.077005	0.170894
Sinapyl alcohol-vs-Streptococcus	-0.36128	0.002667	0.012359
Sinapyl alcohol-vs-Erysipelatoclostridium	-0.35083	0.003606	0.015742

Sinapyl alcohol-vs-Lachnospira	0.054753	0.659887	0.769286
Sinapyl alcohol-vs-Fusobacterium	-0.11238	0.36524	0.525523
Sinapyl alcohol-vs-Bacillus	-0.03823	0.758722	0.846172
Sinapyl alcohol-vs-Dorea	-0.01361	0.912966	0.946214
Sinapyl alcohol-vs-Tyzzerella	-0.08337	0.502404	0.642684
Sinapyl alcohol-vs-Butyrivibrio	0.121398	0.327771	0.487925
Sinapyl alcohol-vs-Mycoplasma	-0.02043	0.869639	0.918358
Sinapyl alcohol-vs-Coprobacillus	-0.20844	0.090519	0.194291
Sinapyl alcohol-vs-Selenomonas	-0.04921	0.692528	0.796209
Sinapyl alcohol-vs-Anaerostipes	-0.05571	0.654313	0.76463
Sinapyl alcohol-vs-Peptoclostridium	-0.01708	0.890882	0.933324
Sinapyl alcohol-vs-Dysgonomonas	-0.19834	0.107625	0.220819
Sinapyl alcohol-vs-Coprobacter	-0.09147	0.461638	0.609554
Sinapyl alcohol-vs-Capnocytophaga	-0.10927	0.378755	0.538312
Sinapyl alcohol-vs-Campylobacter	-0.06469	0.603001	0.726694
Sinapyl alcohol-vs-Flavobacterium	-0.11777	0.342554	0.50312
Artemisinin-vs-Prevotella	0.323968	0.007486	0.027944
Artemisinin-vs-Alistipes	0.09494	0.444737	0.596682
Artemisinin-vs-Faecalibacterium	0.10787	0.384916	0.543104
Artemisinin-vs-Oscillibacter	0.079456	0.522729	0.659112
Artemisinin-vs-Subdoligranulum	-0.18848	0.126639	0.246452
Artemisinin-vs-Bilophila	0.058105	0.64046	0.757492
Artemisinin-vs-Anaerotruncus	-0.1046	0.399582	0.554488
Artemisinin-vs-Butyricicoccus	0.208317	0.090709	0.194525
Artemisinin-vs-Paraprevotella	0.111422	0.369367	0.52901

Artemisinin-vs-Collinsella	0.30166	0.013108	0.0432
Artemisinin-vs-Parasutterella	0.2327	0.058094	0.136039
Artemisinin-vs-Pyramidobacter	0.151888	0.219826	0.371081
Artemisinin-vs-Intestinimonas	0.059382	0.633121	0.75185
Artemisinin-vs-Pseudoflavonifractor	-0.03065	0.805521	0.875238
Artemisinin-vs-Holdemania	0.079057	0.524826	0.660194
Artemisinin-vs-Oribacterium	0.033203	0.789672	0.866067
Artemisinin-vs-Bacteroides	-0.25688	0.035864	0.094813
Artemisinin-vs-Eubacterium	-0.10188	0.41199	0.56458
Artemisinin-vs-Veillonella	-0.14347	0.246769	0.404701
Artemisinin-vs-Lactobacillus	-0.19411	0.115495	0.23143
Artemisinin-vs-Agathobacter	-0.15157	0.220808	0.371702
Artemisinin-vs-Enterococcus	-0.34835	0.003867	0.016586
Artemisinin-vs-Blautia	-0.28833	0.017973	0.055527
Artemisinin-vs-Streptococcus	-0.18896	0.12566	0.245534
Artemisinin-vs-Erysipelatoclostridium	-0.48811	2.79E-05	0.000406
Artemisinin-vs-Lachnospira	0.026818	0.829439	0.891739
Artemisinin-vs-Fusobacterium	-0.30389	0.012415	0.041655
Artemisinin-vs-Bacillus	-0.35581	0.003126	0.013976
Artemisinin-vs-Dorea	-0.05088	0.682603	0.787418
Artemisinin-vs-Tyzzerella	-0.28242	0.02058	0.061689
Artemisinin-vs-Butyrivibrio	-0.18266	0.139021	0.263511
Artemisinin-vs-Mycoplasma	-0.2999	0.013676	0.044645
Artemisinin-vs-Coprobacillus	-0.50643	1.23E-05	0.000222
Artemisinin-vs-Selenomonas	-0.15596	0.207569	0.354598

Artemisinin-vs-Anaerostipes	-0.22508	0.067064	0.153334
Artemisinin-vs-Peptoclostridium	-0.26694	0.028986	0.079951
Artemisinin-vs-Dysgonomonas	-0.16298	0.187574	0.327566
Artemisinin-vs-Coprobacter	0.148735	0.229661	0.381508
Artemisinin-vs-Capnocytophaga	-0.00942	0.939704	0.964423
Artemisinin-vs-Campylobacter	-0.30453	0.012223	0.041355
Artemisinin-vs-Flavobacterium	0.091388	0.462031	0.60974
Dehydroepiandrosterone sulfate-vs-Prevotella	0.14243	0.250239	0.408729
Dehydroepiandrosterone sulfate-vs-Alistipes	-0.19156	0.120456	0.238839
Dehydroepiandrosterone sulfate-vs-Faecalibacterium	0.130537	0.292388	0.453679
Dehydroepiandrosterone sulfate-vs-Oscillibacter	-0.11174	0.367988	0.52766
Dehydroepiandrosterone sulfate-vs-Subdoligranulum	-0.13381	0.280352	0.439799
Dehydroepiandrosterone sulfate-vs-Bilophila	0.051361	0.679777	0.784907
Dehydroepiandrosterone sulfate-vs-Anaerotruncus	-0.27285	0.02549	0.072182
Dehydroepiandrosterone sulfate-vs-Butyricicoccus	0.004071	0.97392	0.984913
Dehydroepiandrosterone sulfate-vs-Paraprevotella	0.111741	0.367988	0.52766
Dehydroepiandrosterone sulfate-vs-Collinsella	0.053755	0.665714	0.77384
Dehydroepiandrosterone sulfate-vs-Parasutterella	0.023226	0.852005	0.905536
Dehydroepiandrosterone sulfate-vs-Pyramidobacter	-0.26287	0.031623	0.085662
Dehydroepiandrosterone sulfate-vs-Intestinimonas	-0.20045	0.103849	0.214527
Dehydroepiandrosterone sulfate-vs-Pseudoflavonifractor	-0.2849	0.019451	0.058963
Dehydroepiandrosterone sulfate-vs-Holdemania	-0.14726	0.234371	0.388584
Dehydroepiandrosterone sulfate-vs-Oribacterium	-0.14023	0.257688	0.417793
Dehydroepiandrosterone sulfate-vs-Bacteroides	-0.01548	0.901026	0.939071
Dehydroepiandrosterone sulfate-vs-Eubacterium	-0.34743	0.003968	0.01686

Dehydroepiandrosterone sulfate-vs-Veillonella	-0.3195	0.008402	0.030518
Dehydroepiandrosterone sulfate-vs-Lactobacillus	-0.46728	6.71E-05	0.000769
Dehydroepiandrosterone sulfate-vs-Agathobacter	-0.17148	0.165281	0.299486
Dehydroepiandrosterone sulfate-vs-Enterococcus	-0.50822	1.13E-05	0.00021
Dehydroepiandrosterone sulfate-vs-Blautia	-0.14885	0.229282	0.381508
Dehydroepiandrosterone sulfate-vs-Streptococcus	-0.35226	0.003462	0.015197
Dehydroepiandrosterone sulfate-vs-Erysipelatoclostridium	-0.24571	0.045047	0.111534
Dehydroepiandrosterone sulfate-vs-Lachnospira	0.012411	0.920597	0.952898
Dehydroepiandrosterone sulfate-vs-Fusobacterium	-0.28654	0.018734	0.057437
Dehydroepiandrosterone sulfate-vs-Bacillus	-0.51317	8.98E-06	0.000178
Dehydroepiandrosterone sulfate-vs-Dorea	-0.2922	0.016423	0.051775
Dehydroepiandrosterone sulfate-vs-Tyzzerella	-0.28163	0.020956	0.062661
Dehydroepiandrosterone sulfate-vs-Butyrivibrio	-0.3282	0.0067	0.02567
Dehydroepiandrosterone sulfate-vs-Mycoplasma	-0.3442	0.004343	0.018114
Dehydroepiandrosterone sulfate-vs-Coprobacillus	-0.31682	0.008996	0.032337
Dehydroepiandrosterone sulfate-vs-Selenomonas	-0.3428	0.004515	0.018637
Dehydroepiandrosterone sulfate-vs-Anaerostipes	-0.23354	0.05717	0.134266
Dehydroepiandrosterone sulfate-vs-Peptoclostridium	-0.21738	0.077227	0.17123
Dehydroepiandrosterone sulfate-vs-Dysgonomonas	-0.36076	0.002708	0.012524
Dehydroepiandrosterone sulfate-vs-Coprobacter	-0.07582	0.541964	0.676825
Dehydroepiandrosterone sulfate-vs-Capnocytophaga	0.000359	0.997698	0.998524
Dehydroepiandrosterone sulfate-vs-Campylobacter	-0.23462	0.056	0.132547
Dehydroepiandrosterone sulfate-vs-Flavobacterium	-0.2667	0.029136	0.080182
Nervonic acid-vs-Prevotella	0.097414	0.432905	0.586009
Nervonic acid-vs-Alistipes	0.386384	0.00124	0.006847

Nervonic acid-vs-Faecalibacterium	0.555112	1.09E-06	3.93E-05
Nervonic acid-vs-Oscillibacter	0.622476	1.87E-08	2.38E-06
Nervonic acid-vs-Subdoligranulum	0.417871	0.000434	0.003008
Nervonic acid-vs-Bilophila	0.275361	0.024114	0.06934
Nervonic acid-vs-Anaerotruncus	0.471147	5.72E-05	0.000689
Nervonic acid-vs-Butyricicoccus	0.495371	2.03E-05	0.000322
Nervonic acid-vs-Paraprevotella	0.233738	0.056952	0.134144
Nervonic acid-vs-Collinsella	0.343762	0.004396	0.018305
Nervonic acid-vs-Parasutterella	0.266741	0.029111	0.080182
Nervonic acid-vs-Pyramidobacter	0.385226	0.001286	0.00707
Nervonic acid-vs-Intestinimonas	0.454945	0.00011	0.001054
Nervonic acid-vs-Pseudoflavonifractor	0.387541	0.001195	0.006645
Nervonic acid-vs-Holdemania	0.480884	3.81E-05	0.000503
Nervonic acid-vs-Oribacterium	0.416394	0.000457	0.003158
Nervonic acid-vs-Bacteroides	-0.14662	0.236428	0.391191
Nervonic acid-vs-Eubacterium	0.252933	0.038914	0.101001
Nervonic acid-vs-Veillonella	-0.39105	0.001068	0.006049
Nervonic acid-vs-Lactobacillus	-0.08672	0.485313	0.629476
Nervonic acid-vs-Agathobacter	0.311517	0.010285	0.036163
Nervonic acid-vs-Enterococcus	-0.12707	0.305523	0.466873
Nervonic acid-vs-Blautia	-0.19307	0.117491	0.234305
Nervonic acid-vs-Streptococcus	-0.08859	0.475887	0.619909
Nervonic acid-vs-Erysipelatoclostridium	-0.19199	0.119592	0.23771
Nervonic acid-vs-Lachnospira	0.201093	0.10273	0.213087
Nervonic acid-vs-Fusobacterium	-0.0158	0.898996	0.939071

Nervonic acid-vs-Bacillus	0.112419	0.365069	0.525523
Nervonic acid-vs-Dorea	0.092026	0.458895	0.607258
Nervonic acid-vs-Tyzzerella	-0.15767	0.20255	0.347893
Nervonic acid-vs-Butyrivibrio	0.33618	0.005412	0.02164
Nervonic acid-vs-Mycoplasma	0.114295	0.357071	0.517399
Nervonic acid-vs-Coprobacillus	-0.20325	0.099021	0.207349
Nervonic acid-vs-Selenomonas	0.187565	0.128532	0.248856
Nervonic acid-vs-Anaerostipes	0.066286	0.594067	0.720686
Nervonic acid-vs-Peptoclostridium	0.156397	0.206277	0.353339
Nervonic acid-vs-Dysgonomonas	-0.09538	0.442624	0.594508
Nervonic acid-vs-Coprobacter	0.24555	0.045191	0.111549
Nervonic acid-vs-Capnocytophaga	-0.12563	0.311069	0.47118
Nervonic acid-vs-Campylobacter	0.084923	0.494431	0.636524
Nervonic acid-vs-Flavobacterium	0.084803	0.495042	0.636632
9-Pentofuranosyl-3,9-dihydro-1H-purine-2,6-dione-vs-Prevotella	0.174794	0.157143	0.287758
9-Pentofuranosyl-3,9-dihydro-1H-purine-2,6-dione-vs-Alistipes	0.446604	0.000152	0.001325
9-Pentofuranosyl-3,9-dihydro-1H-purine-2,6-dione-vs-Faecalibacterium	0.398675	0.000833	0.005063
9-Pentofuranosyl-3,9-dihydro-1H-purine-2,6-dione-vs-Oscillibacter	0.532884	3.45E-06	8.87E-05
9-Pentofuranosyl-3,9-dihydro-1H-purine-2,6-dione-vs-Subdoligranulum	0.309043	0.010939	0.037856

9-Pentofuranosyl-3,9-dihydro-1H-purine-2,6-dione-vs-Bilophila	0.350706	0.003618	0.015742
9-Pentofuranosyl-3,9-dihydro-1H-purine-2,6-dione-vs-Anaerotruncus	0.543699	1.99E-06	5.93E-05
9-Pentofuranosyl-3,9-dihydro-1H-purine-2,6-dione-vs-Butyricicoccus	0.470189	5.95E-05	0.000709
9-Pentofuranosyl-3,9-dihydro-1H-purine-2,6-dione-vs-Paraprevotella	0.204126	0.09754	0.205709
9-Pentofuranosyl-3,9-dihydro-1H-purine-2,6-dione-vs-Collinsella	0.271849	0.026054	0.073457
9-Pentofuranosyl-3,9-dihydro-1H-purine-2,6-dione-vs-Parasutterella	0.161146	0.192661	0.333844
9-Pentofuranosyl-3,9-dihydro-1H-purine-2,6-dione-vs-Pyramidobacter	0.276918	0.023294	0.067972
9-Pentofuranosyl-3,9-dihydro-1H-purine-2,6-dione-vs-Intestinimonas	0.393248	0.000995	0.005798
9-Pentofuranosyl-3,9-dihydro-1H-purine-2,6-dione-vs-Pseudoflavonifractor	0.438064	0.000209	0.00166
9-Pentofuranosyl-3,9-dihydro-1H-purine-2,6-dione-vs-Holdemania	0.368984	0.002122	0.010286
9-Pentofuranosyl-3,9-dihydro-1H-purine-2,6-dione-vs-Oribacterium	0.331232	0.006182	0.023875
9-Pentofuranosyl-3,9-dihydro-1H-purine-2,6-dione-vs-Bacteroides	-0.19662	0.110766	0.223846

9-Pentofuranosyl-3,9-dihydro-1H-purine-2,6-dione-vs-Eubacterium	0.079416	0.522938	0.659112
9-Pentofuranosyl-3,9-dihydro-1H-purine-2,6-dione-vs-Veillonella	-0.41192	0.000533	0.003554
9-Pentofuranosyl-3,9-dihydro-1H-purine-2,6-dione-vs-Lactobacillus	-0.08181	0.510443	0.649191
9-Pentofuranosyl-3,9-dihydro-1H-purine-2,6-dione-vs-Agathobacter	0.119802	0.334219	0.495086
9-Pentofuranosyl-3,9-dihydro-1H-purine-2,6-dione-vs-Enterococcus	-0.09578	0.440709	0.593253
9-Pentofuranosyl-3,9-dihydro-1H-purine-2,6-dione-vs-Blautia	-0.03009	0.808998	0.87717
9-Pentofuranosyl-3,9-dihydro-1H-purine-2,6-dione-vs-Streptococcus	-0.18808	0.127459	0.247451
9-Pentofuranosyl-3,9-dihydro-1H-purine-2,6-dione-vs-Erysipelatoclostridium	-0.21311	0.083368	0.181519
9-Pentofuranosyl-3,9-dihydro-1H-purine-2,6-dione-vs-Lachnospira	-0.05451	0.661284	0.770542
9-Pentofuranosyl-3,9-dihydro-1H-purine-2,6-dione-vs-Fusobacterium	-0.06816	0.583646	0.714041
9-Pentofuranosyl-3,9-dihydro-1H-purine-2,6-dione-vs-Bacillus	0.045734	0.713249	0.813461
9-Pentofuranosyl-3,9-dihydro-1H-purine-2,6-dione-vs-Dorea	0.057866	0.64184	0.757914
9-Pentofuranosyl-3,9-dihydro-1H-purine-2,6-dione-vs-Tyzzerella	0.067284	0.588513	0.716825

9-Pentofuranosyl-3,9-dihydro-1H-purine-2,6-dione-vs-Butyrivibrio	0.228191	0.063275	0.146611
9-Pentofuranosyl-3,9-dihydro-1H-purine-2,6-dione-vs-Mycoplasma	0.174196	0.158591	0.289752
9-Pentofuranosyl-3,9-dihydro-1H-purine-2,6-dione-vs-Coprobacillus	-0.24723	0.043698	0.109425
9-Pentofuranosyl-3,9-dihydro-1H-purine-2,6-dione-vs-Selenomonas	0.15065	0.22365	0.37466
9-Pentofuranosyl-3,9-dihydro-1H-purine-2,6-dione-vs-Anaerostipes	-0.01026	0.93435	0.961375
9-Pentofuranosyl-3,9-dihydro-1H-purine-2,6-dione-vs-Peptoclostridium	0.108309	0.382973	0.541445
9-Pentofuranosyl-3,9-dihydro-1H-purine-2,6-dione-vs-Dysgonomonas	-0.03775	0.761654	0.848814
9-Pentofuranosyl-3,9-dihydro-1H-purine-2,6-dione-vs-Coprobacter	0.098492	0.427809	0.580409
9-Pentofuranosyl-3,9-dihydro-1H-purine-2,6-dione-vs-Capnocytophaga	-0.08269	0.5059	0.646472
9-Pentofuranosyl-3,9-dihydro-1H-purine-2,6-dione-vs-Campylobacter	0.111102	0.370748	0.530361
9-Pentofuranosyl-3,9-dihydro-1H-purine-2,6-dione-vs-Flavobacterium	0.062415	0.615832	0.736651
Stearic acid-vs-Prevotella	0.108069	0.384032	0.542308
Stearic acid-vs-Alistipes	0.391811	0.001042	0.005945
Stearic acid-vs-Faecalibacterium	0.463644	7.77E-05	0.000854

Stearic acid-vs-Oscillibacter	0.610823	4.04E-08	4.07E-06
Stearic acid-vs-Subdoligranulum	0.36192	0.002617	0.012199
Stearic acid-vs-Bilophila	0.303097	0.012659	0.042296
Stearic acid-vs-Anaerotruncus	0.571195	4.47E-07	2.04E-05
Stearic acid-vs-Butyricicoccus	0.583606	2.18E-07	1.23E-05
Stearic acid-vs-Paraprevotella	0.028414	0.819453	0.884541
Stearic acid-vs-Collinsella	0.309721	0.010756	0.037421
Stearic acid-vs-Parasutterella	0.27568	0.023944	0.069075
Stearic acid-vs-Pyramidobacter	0.44405	0.000167	0.001404
Stearic acid-vs-Intestinimonas	0.586559	1.83E-07	1.08E-05
Stearic acid-vs-Pseudoflavonifractor	0.612579	3.6E-08	3.92E-06
Stearic acid-vs-Holdemania	0.382832	0.001387	0.007422
Stearic acid-vs-Oribacterium	0.320057	0.008283	0.030174
Stearic acid-vs-Bacteroides	-0.2157	0.079593	0.175351
Stearic acid-vs-Eubacterium	0.065289	0.599644	0.724906
Stearic acid-vs-Veillonella	-0.41109	0.000549	0.003613
Stearic acid-vs-Lactobacillus	-0.10376	0.40339	0.556012
Stearic acid-vs-Agathobacter	-0.13856	0.263477	0.424617
Stearic acid-vs-Enterococcus	-0.19726	0.109589	0.222545
Stearic acid-vs-Blautia	-0.14881	0.229409	0.381508
Stearic acid-vs-Streptococcus	-0.26742	0.028688	0.079311
Stearic acid-vs-Erysipelatoclostridium	-0.24571	0.045047	0.111534
Stearic acid-vs-Lachnospira	-0.27117	0.026444	0.07406
Stearic acid-vs-Fusobacterium	-0.16765	0.175074	0.312089
Stearic acid-vs-Bacillus	-0.06166	0.620135	0.740428

Stearic acid-vs-Dorea	-0.19858	0.107193	0.220305
Stearic acid-vs-Tyzzerella	0.007383	0.952717	0.972675
Stearic acid-vs-Butyrivibrio	0.07311	0.556559	0.68778
Stearic acid-vs-Mycoplasma	0.03859	0.756525	0.844111
Stearic acid-vs-Coprobacillus	-0.35382	0.003311	0.014696
Stearic acid-vs-Selenomonas	0.046053	0.711335	0.812426
Stearic acid-vs-Anaerostipes	-0.12858	0.29974	0.460947
Stearic acid-vs-Peptoclostridium	-0.01576	0.89925	0.939071
Stearic acid-vs-Dysgonomonas	-0.15604	0.207334	0.354446
Stearic acid-vs-Coprobacter	-0.02514	0.839954	0.89865
Stearic acid-vs-Capnocytophaga	-0.17675	0.15248	0.280921
Stearic acid-vs-Campylobacter	-0.10188	0.41199	0.56458
Stearic acid-vs-Flavobacterium	-0.12711	0.30537	0.466873
Niacin-vs-Prevotella	0.118206	0.340746	0.502294
Niacin-vs-Alistipes	0.391931	0.001038	0.005945
Niacin-vs-Faecalibacterium	0.555272	1.08E-06	3.93E-05
Niacin-vs-Oscillibacter	0.515285	8.12E-06	0.000165
Niacin-vs-Subdoligranulum	0.472863	5.33E-05	0.000651
Niacin-vs-Bilophila	0.334225	0.005706	0.022369
Niacin-vs-Anaerotruncus	0.463126	7.93E-05	0.000861
Niacin-vs-Butyricicoccus	0.528693	4.25E-06	0.000103
Niacin-vs-Paraprevotella	0.246069	0.044724	0.111305
Niacin-vs-Collinsella	0.295395	0.015231	0.048416
Niacin-vs-Parasutterella	0.166813	0.177272	0.315078
Niacin-vs-Pyramidobacter	0.31455	0.00953	0.033804

Niacin-vs-Intestinimonas	0.424575	0.000342	0.002525
Niacin-vs-Pseudoflavonifractor	0.427209	0.000312	0.002333
Niacin-vs-Holdemania	0.463205	7.91E-05	0.000861
Niacin-vs-Oribacterium	0.4397	0.000197	0.001583
Niacin-vs-Bacteroides	-0.10567	0.394717	0.551533
Niacin-vs-Eubacterium	0.245271	0.045444	0.111945
Niacin-vs-Veillonella	-0.37218	0.001927	0.00971
Niacin-vs-Lactobacillus	-0.11605	0.349683	0.510118
Niacin-vs-Agathobacter	0.278833	0.022318	0.065816
Niacin-vs-Enterococcus	-0.11829	0.340417	0.502116
Niacin-vs-Blautia	-0.0071	0.954504	0.973417
Niacin-vs-Streptococcus	-0.21758	0.076949	0.170894
Niacin-vs-Erysipelatoclostridium	-0.22324	0.069383	0.157742
Niacin-vs-Lachnospira	0.154881	0.210766	0.358539
Niacin-vs-Fusobacterium	0.042222	0.734422	0.82785
Niacin-vs-Bacillus	0.082449	0.507137	0.647291
Niacin-vs-Dorea	0.18513	0.133656	0.255786
Niacin-vs-Tyzzerella	0.101844	0.412174	0.56458
Niacin-vs-Butyrivibrio	0.350227	0.003668	0.0159
Niacin-vs-Mycoplasma	0.085561	0.491179	0.634363
Niacin-vs-Coprobacillus	-0.11034	0.374043	0.533811
Niacin-vs-Selenomonas	-0.01568	0.899757	0.939071
Niacin-vs-Anaerostipes	0.041703	0.737567	0.829462
Niacin-vs-Peptoclostridium	0.125868	0.31014	0.470658
Niacin-vs-Dysgonomonas	-0.0176	0.887589	0.930277

Niacin-vs-Coprobacter	0.116769	0.346688	0.50765
Niacin-vs-Capnocytophaga	-0.09334	0.452464	0.603369
Niacin-vs-Campylobacter	0.235134	0.055443	0.131487
Niacin-vs-Flavobacterium	-0.08117	0.51376	0.651355
D-threonic acid-vs-Prevotella	0.069678	0.575281	0.707746
D-threonic acid-vs-Alistipes	0.279472	0.022	0.065138
D-threonic acid-vs-Faecalibacterium	0.39652	0.000894	0.005301
D-threonic acid-vs-Oscillibacter	0.439939	0.000195	0.001576
D-threonic acid-vs-Subdoligranulum	0.370261	0.002042	0.010018
D-threonic acid-vs-Bilophila	0.335302	0.005542	0.021941
D-threonic acid-vs-Anaerotruncus	0.44413	0.000167	0.001404
D-threonic acid-vs-Butyricicoccus	0.492218	2.33E-05	0.00035
D-threonic acid-vs-Paraprevotella	0.16378	0.185393	0.325683
D-threonic acid-vs-Collinsella	0.328159	0.006707	0.02567
D-threonic acid-vs-Parasutterella	0.198061	0.108132	0.221483
D-threonic acid-vs-Pyramidobacter	0.327321	0.006857	0.026161
D-threonic acid-vs-Intestinimonas	0.445247	0.00016	0.00138
D-threonic acid-vs-Pseudoflavonifractor	0.503233	1.42E-05	0.000246
D-threonic acid-vs-Holdemania	0.383311	0.001366	0.00736
D-threonic acid-vs-Oribacterium	0.265704	0.029767	0.081364
D-threonic acid-vs-Bacteroides	-0.21638	0.078629	0.173701
D-threonic acid-vs-Eubacterium	-0.02015	0.871406	0.918888
D-threonic acid-vs-Veillonella	-0.42174	0.000379	0.002722
D-threonic acid-vs-Lactobacillus	-0.19766	0.108859	0.222407
D-threonic acid-vs-Agathobacter	-0.02382	0.848235	0.903116

D-threonic acid-vs-Enterococcus	-0.25245	0.039298	0.101779
D-threonic acid-vs-Blautia	-0.09207	0.4587	0.607258
D-threonic acid-vs-Streptococcus	-0.31403	0.009656	0.034199
D-threonic acid-vs-Erysipelatoclostridium	-0.25373	0.038281	0.099894
D-threonic acid-vs-Lachnospira	-0.16462	0.183123	0.323339
D-threonic acid-vs-Fusobacterium	-0.1813	0.142031	0.266916
D-threonic acid-vs-Bacillus	-0.03121	0.802047	0.873942
D-threonic acid-vs-Dorea	-0.08979	0.469922	0.615786
D-threonic acid-vs-Tyzzerella	-0.03404	0.784489	0.862974
D-threonic acid-vs-Butyrivibrio	0.057187	0.645756	0.759399
D-threonic acid-vs-Mycoplasma	-0.13924	0.261123	0.421667
D-threonic acid-vs-Coprobacillus	-0.32349	0.007579	0.028164
D-threonic acid-vs-Selenomonas	-0.10687	0.389353	0.548221
D-threonic acid-vs-Anaerostipes	-0.12295	0.321561	0.482242
D-threonic acid-vs-Peptoclostridium	0.109386	0.37823	0.537882
D-threonic acid-vs-Dysgonomonas	-0.14027	0.257551	0.417793
D-threonic acid-vs-Coprobacter	-0.09765	0.43177	0.584799
D-threonic acid-vs-Capnocytophaga	-0.08337	0.502404	0.642684
D-threonic acid-vs-Campylobacter	-0.09127	0.46262	0.610038
D-threonic acid-vs-Flavobacterium	-0.15436	0.212317	0.36067
L-Theanine-vs-Prevotella	0.043778	0.725013	0.822997
L-Theanine-vs-Alistipes	0.193152	0.117337	0.23419
L-Theanine-vs-Faecalibacterium	0.197262	0.109589	0.222545
L-Theanine-vs-Oscillibacter	0.290207	0.017207	0.053619
L-Theanine-vs-Subdoligranulum	0.201093	0.10273	0.213087

L-Theanine-vs-Bilophila	0.228709	0.06266	0.145715
L-Theanine-vs-Anaerotruncus	0.251377	0.040173	0.102944
L-Theanine-vs-Butyricicoccus	0.510655	1.01E-05	0.000194
L-Theanine-vs-Paraprevotella	0.076183	0.540046	0.675477
L-Theanine-vs-Collinsella	0.359526	0.002807	0.012887
L-Theanine-vs-Parasutterella	0.206441	0.093718	0.199389
L-Theanine-vs-Pyramidobacter	0.267619	0.028565	0.079061
L-Theanine-vs-Intestinimonas	0.370381	0.002034	0.010018
L-Theanine-vs-Pseudoflavonifractor	0.452949	0.000119	0.001122
L-Theanine-vs-Holdemania	0.255846	0.036645	0.096352
L-Theanine-vs-Oribacterium	0.097254	0.433663	0.586378
L-Theanine-vs-Bacteroides	-0.12607	0.309367	0.470544
L-Theanine-vs-Eubacterium	-0.16478	0.182693	0.323195
L-Theanine-vs-Veillonella	-0.3695	0.002089	0.010208
L-Theanine-vs-Lactobacillus	-0.2568	0.035923	0.094867
L-Theanine-vs-Agathobacter	-0.28334	0.020155	0.06049
L-Theanine-vs-Enterococcus	-0.22157	0.071556	0.161468
L-Theanine-vs-Blautia	-0.03165	0.79932	0.872543
L-Theanine-vs-Streptococcus	-0.33849	0.005082	0.020593
L-Theanine-vs-Erysipelatoclostridium	-0.17136	0.16558	0.299805
L-Theanine-vs-Lachnospira	-0.25146	0.040108	0.102886
L-Theanine-vs-Fusobacterium	-0.2151	0.080452	0.176921
L-Theanine-vs-Bacillus	-0.11509	0.353701	0.514428
L-Theanine-vs-Dorea	-0.20353	0.098548	0.206753
L-Theanine-vs-Tyzzerella	-0.15293	0.216655	0.366495

L-Theanine-vs-Butyrivibrio	-0.16174	0.190991	0.331426
L-Theanine-vs-Mycoplasma	-0.27113	0.026467	0.07406
L-Theanine-vs-Coprobacillus	-0.44904	0.000138	0.001253
L-Theanine-vs-Selenomonas	-0.06736	0.58807	0.716825
L-Theanine-vs-Anaerostipes	-0.22524	0.066865	0.153025
L-Theanine-vs-Peptoclostridium	0.053556	0.666881	0.774825
L-Theanine-vs-Dysgonomonas	-0.08656	0.48612	0.629847
L-Theanine-vs-Coprobacter	-0.10328	0.405576	0.55807
L-Theanine-vs-Capnocytophaga	-0.13433	0.278474	0.43799
L-Theanine-vs-Campylobacter	-0.2343	0.056344	0.133102
L-Theanine-vs-Flavobacterium	-0.10615	0.392566	0.550184
(3alpha,5alpha,14xi,22R,23R,24S)-3,22,23-Trihydroxyergostan-6-one-vs-Prevotella	0.270293	0.026956	0.074863
(3alpha,5alpha,14xi,22R,23R,24S)-3,22,23-Trihydroxyergostan-6-one-vs-Alistipes	0.289089	0.01766	0.054769
(3alpha,5alpha,14xi,22R,23R,24S)-3,22,23-Trihydroxyergostan-6-one-vs-Faecalibacterium	0.574906	3.62E-07	1.72E-05
(3alpha,5alpha,14xi,22R,23R,24S)-3,22,23-Trihydroxyergostan-6-one-vs-Oscillibacter	0.466518	6.92E-05	0.000789
(3alpha,5alpha,14xi,22R,23R,24S)-3,22,23-Trihydroxyergostan-6-one-vs-Subdoligranulum	0.230824	0.060205	0.14044
(3alpha,5alpha,14xi,22R,23R,24S)-3,22,23-Trihydroxyergostan-6-one-vs-Bilophila	0.270612	0.026769	0.074515
(3alpha,5alpha,14xi,22R,23R,24S)-3,22,23-Trihydroxyergostan-6-one-vs-Anaerotruncus	0.365233	0.002373	0.011227

(3alpha,5alpha,14xi,22R,23R,24S)-3,22,23-Trihydroxyergostan-6-one-vs-Butyricicoccus	0.465081	7.33E-05	0.000817
(3alpha,5alpha,14xi,22R,23R,24S)-3,22,23-Trihydroxyergostan-6-one-vs-Paraprevotella	0.191356	0.120851	0.239229
(3alpha,5alpha,14xi,22R,23R,24S)-3,22,23-Trihydroxyergostan-6-one-vs-Collinsella	0.383351	0.001364	0.00736
(3alpha,5alpha,14xi,22R,23R,24S)-3,22,23-Trihydroxyergostan-6-one-vs-Parasutterella	0.272927	0.025446	0.072161
(3alpha,5alpha,14xi,22R,23R,24S)-3,22,23-Trihydroxyergostan-6-one-vs-Pyramidobacter	0.191915	0.119749	0.237826
(3alpha,5alpha,14xi,22R,23R,24S)-3,22,23-Trihydroxyergostan-6-one-vs-Intestinimonas	0.389856	0.00111	0.006257
(3alpha,5alpha,14xi,22R,23R,24S)-3,22,23-Trihydroxyergostan-6-one-vs-Pseudoflavonifractor	0.349429	0.003751	0.016205
(3alpha,5alpha,14xi,22R,23R,24S)-3,22,23-Trihydroxyergostan-6-one-vs-Holdemania	0.365552	0.002351	0.011151
(3alpha,5alpha,14xi,22R,23R,24S)-3,22,23-Trihydroxyergostan-6-one-vs-Oribacterium	0.187325	0.129029	0.2491
(3alpha,5alpha,14xi,22R,23R,24S)-3,22,23-Trihydroxyergostan-6-one-vs-Bacteroides	-0.24635	0.044475	0.110912
(3alpha,5alpha,14xi,22R,23R,24S)-3,22,23-Trihydroxyergostan-6-one-vs-Eubacterium	-0.10404	0.402118	0.555208
(3alpha,5alpha,14xi,22R,23R,24S)-3,22,23-Trihydroxyergostan-6-one-vs-Veillonella	-0.48464	3.24E-05	0.000453

(3alpha,5alpha,14xi,22R,23R,24S)-3,22,23-Trihydroxyergostan-6-one-vs-Lactobacillus	-0.3462	0.004108	0.017313
(3alpha,5alpha,14xi,22R,23R,24S)-3,22,23-Trihydroxyergostan-6-one-vs-Agathobacter	-0.00555	0.964465	0.978214
(3alpha,5alpha,14xi,22R,23R,24S)-3,22,23-Trihydroxyergostan-6-one-vs-Enterococcus	-0.40781	0.000614	0.003907
(3alpha,5alpha,14xi,22R,23R,24S)-3,22,23-Trihydroxyergostan-6-one-vs-Blautia	-0.34245	0.00456	0.018727
(3alpha,5alpha,14xi,22R,23R,24S)-3,22,23-Trihydroxyergostan-6-one-vs-Streptococcus	-0.4132	0.00051	0.003453
(3alpha,5alpha,14xi,22R,23R,24S)-3,22,23-Trihydroxyergostan-6-one-vs-Erysipelatoclostridium	-0.46205	8.28E-05	0.000882
(3alpha,5alpha,14xi,22R,23R,24S)-3,22,23-Trihydroxyergostan-6-one-vs-Lachnospira	-0.06876	0.580337	0.711794
(3alpha,5alpha,14xi,22R,23R,24S)-3,22,23-Trihydroxyergostan-6-one-vs-Fusobacterium	-0.35306	0.003384	0.014882
(3alpha,5alpha,14xi,22R,23R,24S)-3,22,23-Trihydroxyergostan-6-one-vs-Bacillus	-0.19347	0.11672	0.233152
(3alpha,5alpha,14xi,22R,23R,24S)-3,22,23-Trihydroxyergostan-6-one-vs-Dorea	-0.12224	0.324418	0.485623
(3alpha,5alpha,14xi,22R,23R,24S)-3,22,23-Trihydroxyergostan-6-one-vs-Tyzzerella	-0.1991	0.10626	0.218573
(3alpha,5alpha,14xi,22R,23R,24S)-3,22,23-Trihydroxyergostan-6-one-vs-Butyrivibrio	-0.0184	0.882527	0.926576

(3alpha,5alpha,14xi,22R,23R,24S)-3,22,23-Trihydroxyergostan-6-one-vs-Mycoplasma	-0.14255	0.249837	0.408624
(3alpha,5alpha,14xi,22R,23R,24S)-3,22,23-Trihydroxyergostan-6-one-vs-Coprobacillus	-0.39333	0.000992	0.005798
(3alpha,5alpha,14xi,22R,23R,24S)-3,22,23-Trihydroxyergostan-6-one-vs-Selenomonas	-0.1378	0.266124	0.427744
(3alpha,5alpha,14xi,22R,23R,24S)-3,22,23-Trihydroxyergostan-6-one-vs-Anaerostipes	-0.18996	0.123639	0.242919
(3alpha,5alpha,14xi,22R,23R,24S)-3,22,23-Trihydroxyergostan-6-one-vs-Peptoclostridium	-0.0332	0.789672	0.866067
(3alpha,5alpha,14xi,22R,23R,24S)-3,22,23-Trihydroxyergostan-6-one-vs-Dysgonomonas	-0.30773	0.011301	0.03876
(3alpha,5alpha,14xi,22R,23R,24S)-3,22,23-Trihydroxyergostan-6-one-vs-Coprobacter	-0.04306	0.72935	0.823669
(3alpha,5alpha,14xi,22R,23R,24S)-3,22,23-Trihydroxyergostan-6-one-vs-Capnocytophaga	-0.28685	0.018597	0.057089
(3alpha,5alpha,14xi,22R,23R,24S)-3,22,23-Trihydroxyergostan-6-one-vs-Campylobacter	-0.18074	0.143284	0.268478
(3alpha,5alpha,14xi,22R,23R,24S)-3,22,23-Trihydroxyergostan-6-one-vs-Flavobacterium	-0.19076	0.12204	0.240794
2-Methoxyestradiol-vs-Prevotella	0.08612	0.488343	0.63205
2-Methoxyestradiol-vs-Alistipes	0.34927	0.003768	0.016249
2-Methoxyestradiol-vs-Faecalibacterium	0.447961	0.000144	0.001281
2-Methoxyestradiol-vs-Oscillibacter	0.570197	4.73E-07	0.000021
2-Methoxyestradiol-vs-Subdoligranulum	0.287693	0.018241	0.056209

2-Methoxyestradiol-vs-Bilophila	0.17675	0.15248	0.280921
2-Methoxyestradiol-vs-Anaerotruncus	0.446364	0.000153	0.001332
2-Methoxyestradiol-vs-Butyricicoccus	0.697821	5.29E-11	5.27E-08
2-Methoxyestradiol-vs-Paraprevotella	0.190997	0.121563	0.240246
2-Methoxyestradiol-vs-Collinsella	0.32341	0.007595	0.028179
2-Methoxyestradiol-vs-Parasutterella	0.161745	0.190991	0.331426
2-Methoxyestradiol-vs-Pyramidobacter	0.411406	0.000543	0.003588
2-Methoxyestradiol-vs-Intestinimonas	0.493774	2.18E-05	0.000339
2-Methoxyestradiol-vs-Pseudoflavonifractor	0.487309	2.89E-05	0.000413
2-Methoxyestradiol-vs-Holdemania	0.459973	0.00009	0.000923
2-Methoxyestradiol-vs-Oribacterium	0.413082	0.000513	0.003453
2-Methoxyestradiol-vs-Bacteroides	-0.06297	0.61267	0.734051
2-Methoxyestradiol-vs-Eubacterium	0.200894	0.103079	0.213482
2-Methoxyestradiol-vs-Veillonella	-0.26147	0.032573	0.087939
2-Methoxyestradiol-vs-Lactobacillus	0.005787	0.962932	0.977479
2-Methoxyestradiol-vs-Agathobacter	0.118006	0.341567	0.502892
2-Methoxyestradiol-vs-Enterococcus	-0.09183	0.459874	0.607888
2-Methoxyestradiol-vs-Blautia	-0.09957	0.422747	0.574831
2-Methoxyestradiol-vs-Streptococcus	-0.10663	0.390422	0.548769
2-Methoxyestradiol-vs-Erysipelatoclostridium	-0.25984	0.033716	0.089921
2-Methoxyestradiol-vs-Lachnospira	0.007343	0.952972	0.972675
2-Methoxyestradiol-vs-Fusobacterium	-0.14873	0.229661	0.381508
2-Methoxyestradiol-vs-Bacillus	0.136364	0.271189	0.431867
2-Methoxyestradiol-vs-Dorea	0.104557	0.399763	0.554488
2-Methoxyestradiol-vs-Tyzzerella	-0.04442	0.721165	0.820056

2-Methoxyestradiol-vs-Butyrivibrio	0.218413	0.07579	0.168819
2-Methoxyestradiol-vs-Mycoplasma	0.125708	0.310759	0.47118
2-Methoxyestradiol-vs-Coprobacillus	-0.28035	0.021569	0.064099
2-Methoxyestradiol-vs-Selenomonas	0.067164	0.589178	0.717274
2-Methoxyestradiol-vs-Anaerostipes	-0.02386	0.847984	0.903116
2-Methoxyestradiol-vs-Peptoclostridium	0.100846	0.416792	0.568973
2-Methoxyestradiol-vs-Dysgonomonas	-0.03536	0.776365	0.856764
2-Methoxyestradiol-vs-Coprobacter	0.207878	0.091406	0.195501
2-Methoxyestradiol-vs-Capnocytophaga	-0.17807	0.149398	0.277527
2-Methoxyestradiol-vs-Campylobacter	-0.00255	0.983634	0.98936
2-Methoxyestradiol-vs-Flavobacterium	0.096736	0.436131	0.588072
loganate-vs-Prevotella	0.025541	0.837448	0.897557
loganate-vs-Alistipes	0.174675	0.157432	0.288069
loganate-vs-Faecalibacterium	0.348631	0.003837	0.016486
loganate-vs-Oscillibacter	0.398915	0.000826	0.005049
loganate-vs-Subdoligranulum	0.320417	0.008206	0.029942
loganate-vs-Bilophila	0.285617	0.019134	0.058293
loganate-vs-Anaerotruncus	0.378003	0.001612	0.008331
loganate-vs-Butyricicoccus	0.493415	2.21E-05	0.000343
loganate-vs-Paraprevotella	0.149493	0.227268	0.378885
loganate-vs-Collinsella	0.278554	0.022458	0.066009
loganate-vs-Parasutterella	0.205403	0.095417	0.202075
loganate-vs-Pyramidobacter	0.261314	0.032683	0.08804
loganate-vs-Intestinimonas	0.467954	6.52E-05	0.000759
loganate-vs-Pseudoflavonifractor	0.538471	2.6E-06	7.49E-05

loganate-vs-Holdemania	0.346636	0.004058	0.017162
loganate-vs-Oribacterium	0.220289	0.073248	0.164366
loganate-vs-Bacteroides	-0.25174	0.03988	0.102518
loganate-vs-Eubacterium	0.039947	0.748243	0.839401
loganate-vs-Veillonella	-0.3877	0.001189	0.006642
loganate-vs-Lactobacillus	-0.08089	0.515215	0.652174
loganate-vs-Agathobacter	-0.03061	0.805769	0.875238
loganate-vs-Enterococcus	-0.20417	0.097473	0.205709
loganate-vs-Blautia	-0.05419	0.663148	0.77197
loganate-vs-Streptococcus	-0.23486	0.055742	0.132067
loganate-vs-Erysipelatoclostridium	-0.13652	0.270623	0.43125
loganate-vs-Lachnospira	-0.22548	0.066568	0.152634
loganate-vs-Fusobacterium	-0.17791	0.149769	0.277578
loganate-vs-Bacillus	0.018756	0.88025	0.924587
loganate-vs-Dorea	-0.0862	0.487938	0.631864
loganate-vs-Tyzzerella	-0.08576	0.490165	0.63373
loganate-vs-Butyrivibrio	0.065009	0.601209	0.726337
loganate-vs-Mycoplasma	0.004829	0.969064	0.980823
loganate-vs-Coprobacillus	-0.34815	0.003889	0.016621
loganate-vs-Selenomonas	0.059981	0.629693	0.748882
loganate-vs-Anaerostipes	-0.05691	0.647372	0.760461
loganate-vs-Peptoclostridium	0.059981	0.629693	0.748882
loganate-vs-Dysgonomonas	-0.08568	0.49057	0.633915
loganate-vs-Coprobacter	-0.09701	0.434801	0.587261
loganate-vs-Capnocytophaga	-0.08385	0.499944	0.640553

loganate-vs-Campylobacter	-0.01026	0.93435	0.961375
loganate-vs-Flavobacterium	-0.08995	0.469129	0.61534
1-Pentofuranosyl-2,4(1H,3H)-pyrimidinedione-vs-Prevotella	0.182297	0.139813	0.263812
1-Pentofuranosyl-2,4(1H,3H)-pyrimidinedione-vs-Alistipes	0.31052	0.010545	0.03686
1-Pentofuranosyl-2,4(1H,3H)-pyrimidinedione-vs-Faecalibacterium	0.494652	2.09E-05	0.000329
1-Pentofuranosyl-2,4(1H,3H)-pyrimidinedione-vs-Oscillibacter	0.527736	4.45E-06	0.000106
1-Pentofuranosyl-2,4(1H,3H)-pyrimidinedione-vs-Subdoligranulum	0.449078	0.000138	0.001253
1-Pentofuranosyl-2,4(1H,3H)-pyrimidinedione-vs-Bilophila	0.251776	0.039847	0.102518
1-Pentofuranosyl-2,4(1H,3H)-pyrimidinedione-vs-Anaerotruncus	0.393248	0.000995	0.005798
1-Pentofuranosyl-2,4(1H,3H)-pyrimidinedione-vs-Butyricicoccus	0.594301	1.14E-07	8.93E-06
1-Pentofuranosyl-2,4(1H,3H)-pyrimidinedione-vs-Paraprevotella	0.115931	0.350183	0.510297
1-Pentofuranosyl-2,4(1H,3H)-pyrimidinedione-vs-Collinsella	0.423657	0.000354	0.002586
1-Pentofuranosyl-2,4(1H,3H)-pyrimidinedione-vs-Parasutterella	0.109905	0.375958	0.535845
1-Pentofuranosyl-2,4(1H,3H)-pyrimidinedione-vs-Pyramidobacter	0.309761	0.010746	0.037421
1-Pentofuranosyl-2,4(1H,3H)-pyrimidinedione-vs-Intestinimonas	0.442813	0.000175	0.001446

1-Pentofuranosyl-2,4(1H,3H)-pyrimidinedione-vs-Pseudoflavonifractor	0.515045	8.22E-06	0.000165
1-Pentofuranosyl-2,4(1H,3H)-pyrimidinedione-vs-Holdemania	0.52143	6.05E-06	0.000131
1-Pentofuranosyl-2,4(1H,3H)-pyrimidinedione-vs-Oribacterium	0.369223	0.002107	0.010245
1-Pentofuranosyl-2,4(1H,3H)-pyrimidinedione-vs-Bacteroides	-0.06421	0.605692	0.728216
1-Pentofuranosyl-2,4(1H,3H)-pyrimidinedione-vs-Eubacterium	0.023027	0.853262	0.905678
1-Pentofuranosyl-2,4(1H,3H)-pyrimidinedione-vs-Veillonella	-0.47135	5.67E-05	0.000686
1-Pentofuranosyl-2,4(1H,3H)-pyrimidinedione-vs-Lactobacillus	-0.13565	0.273745	0.433087
1-Pentofuranosyl-2,4(1H,3H)-pyrimidinedione-vs-Agathobacter	-0.00487	0.968809	0.980823
1-Pentofuranosyl-2,4(1H,3H)-pyrimidinedione-vs-Enterococcus	-0.20345	0.098683	0.206857
1-Pentofuranosyl-2,4(1H,3H)-pyrimidinedione-vs-Blautia	-0.06485	0.602105	0.726337
1-Pentofuranosyl-2,4(1H,3H)-pyrimidinedione-vs-Streptococcus	-0.1973	0.109516	0.222545
1-Pentofuranosyl-2,4(1H,3H)-pyrimidinedione-vs-Erysipelatoclostridium	-0.14586	0.238888	0.393644
1-Pentofuranosyl-2,4(1H,3H)-pyrimidinedione-vs-Lachnospira	0.033921	0.785229	0.863003
1-Pentofuranosyl-2,4(1H,3H)-pyrimidinedione-vs-Fusobacterium	0.003831	0.975454	0.985334

1-Pentofuranosyl-2,4(1H,3H)-pyrimidinedione-vs-Bacillus	0.081371	0.512722	0.651063
1-Pentofuranosyl-2,4(1H,3H)-pyrimidinedione-vs-Dorea	0.053037	0.669921	0.776492
1-Pentofuranosyl-2,4(1H,3H)-pyrimidinedione-vs-Tyzzerella	-0.06916	0.578137	0.709905
1-Pentofuranosyl-2,4(1H,3H)-pyrimidinedione-vs-Butyrivibrio	0.1208	0.330179	0.490906
1-Pentofuranosyl-2,4(1H,3H)-pyrimidinedione-vs-Mycoplasma	-0.00016	0.998977	0.999233
1-Pentofuranosyl-2,4(1H,3H)-pyrimidinedione-vs-Coprobacillus	-0.23689	0.053593	0.127978
1-Pentofuranosyl-2,4(1H,3H)-pyrimidinedione-vs-Selenomonas	0.011254	0.92798	0.95849
1-Pentofuranosyl-2,4(1H,3H)-pyrimidinedione-vs-Anaerostipes	-0.08484	0.494838	0.636632
1-Pentofuranosyl-2,4(1H,3H)-pyrimidinedione-vs-Peptoclostridium	0.090869	0.464587	0.611446
1-Pentofuranosyl-2,4(1H,3H)-pyrimidinedione-vs-Dysgonomonas	-0.09195	0.459286	0.607443
1-Pentofuranosyl-2,4(1H,3H)-pyrimidinedione-vs-Coprobacter	0.072751	0.558505	0.689297
1-Pentofuranosyl-2,4(1H,3H)-pyrimidinedione-vs-Capnocytophaga	-0.16358	0.185937	0.326119
1-Pentofuranosyl-2,4(1H,3H)-pyrimidinedione-vs-Campylobacter	0.012411	0.920597	0.952898
1-Pentofuranosyl-2,4(1H,3H)-pyrimidinedione-vs-Flavobacterium	-0.0067	0.957058	0.974748

4-Deoxy-5-C-(3,5-di-sec-butyl-1-cyclopenten-1-yl)pentonic acid-vs-Prevotella	0.088674	0.475488	0.619723
4-Deoxy-5-C-(3,5-di-sec-butyl-1-cyclopenten-1-yl)pentonic acid-vs-Alistipes	0.228669	0.062707	0.145715
4-Deoxy-5-C-(3,5-di-sec-butyl-1-cyclopenten-1-yl)pentonic acid-vs-Faecalibacterium	0.61206	3.73E-08	3.92E-06
4-Deoxy-5-C-(3,5-di-sec-butyl-1-cyclopenten-1-yl)pentonic acid-vs-Oscillibacter	0.37062	0.00202	0.009991
4-Deoxy-5-C-(3,5-di-sec-butyl-1-cyclopenten-1-yl)pentonic acid-vs-Subdoligranulum	0.323011	0.007674	0.028429
4-Deoxy-5-C-(3,5-di-sec-butyl-1-cyclopenten-1-yl)pentonic acid-vs-Bilophila	0.146221	0.237721	0.392523
4-Deoxy-5-C-(3,5-di-sec-butyl-1-cyclopenten-1-yl)pentonic acid-vs-Anaerotruncus	0.362439	0.002578	0.012099
4-Deoxy-5-C-(3,5-di-sec-butyl-1-cyclopenten-1-yl)pentonic acid-vs-Butyricicoccus	0.442613	0.000177	0.001447
4-Deoxy-5-C-(3,5-di-sec-butyl-1-cyclopenten-1-yl)pentonic acid-vs-Paraprevotella	0.156357	0.206394	0.353339
4-Deoxy-5-C-(3,5-di-sec-butyl-1-cyclopenten-1-yl)pentonic acid-vs-Collinsella	0.236731	0.053759	0.128122
4-Deoxy-5-C-(3,5-di-sec-butyl-1-cyclopenten-1-yl)pentonic acid-vs-Parasutterella	0.236172	0.054344	0.12936
4-Deoxy-5-C-(3,5-di-sec-butyl-1-cyclopenten-1-yl)pentonic acid-vs-Pyramidobacter	0.321335	0.008015	0.029419

4-Deoxy-5-C-(3,5-di-sec-butyl-1-cyclopenten-1-yl)pentonic acid-vs-Intestinimonas	0.395482	0.000925	0.005457
4-Deoxy-5-C-(3,5-di-sec-butyl-1-cyclopenten-1-yl)pentonic acid-vs-Pseudoflavonifractor	0.340251	0.004844	0.019726
4-Deoxy-5-C-(3,5-di-sec-butyl-1-cyclopenten-1-yl)pentonic acid-vs-Holdemania	0.484276	3.29E-05	0.000458
4-Deoxy-5-C-(3,5-di-sec-butyl-1-cyclopenten-1-yl)pentonic acid-vs-Oribacterium	0.412443	0.000524	0.003511
4-Deoxy-5-C-(3,5-di-sec-butyl-1-cyclopenten-1-yl)pentonic acid-vs-Bacteroides	-0.18868	0.12623	0.245855
4-Deoxy-5-C-(3,5-di-sec-butyl-1-cyclopenten-1-yl)pentonic acid-vs-Eubacterium	0.278793	0.022338	0.065816
4-Deoxy-5-C-(3,5-di-sec-butyl-1-cyclopenten-1-yl)pentonic acid-vs-Veillonella	-0.21083	0.086794	0.186959
4-Deoxy-5-C-(3,5-di-sec-butyl-1-cyclopenten-1-yl)pentonic acid-vs-Lactobacillus	-0.11234	0.365411	0.525523
4-Deoxy-5-C-(3,5-di-sec-butyl-1-cyclopenten-1-yl)pentonic acid-vs-Agathobacter	0.201173	0.102591	0.213087
4-Deoxy-5-C-(3,5-di-sec-butyl-1-cyclopenten-1-yl)pentonic acid-vs-Enterococcus	-0.10831	0.382973	0.541445
4-Deoxy-5-C-(3,5-di-sec-butyl-1-cyclopenten-1-yl)pentonic acid-vs-Blautia	-0.12503	0.313398	0.474115
4-Deoxy-5-C-(3,5-di-sec-butyl-1-cyclopenten-1-yl)pentonic acid-vs-Streptococcus	-0.03185	0.798081	0.871584

4-Deoxy-5-C-(3,5-di-sec-butyl-1-cyclopenten-1-yl)pentonic acid-vs-Erysipelatoclostridium	-0.13876	0.262783	0.423782
4-Deoxy-5-C-(3,5-di-sec-butyl-1-cyclopenten-1-yl)pentonic acid-vs-Lachnospira	0.115093	0.353701	0.514428
4-Deoxy-5-C-(3,5-di-sec-butyl-1-cyclopenten-1-yl)pentonic acid-vs-Fusobacterium	-0.0271	0.82769	0.890254
4-Deoxy-5-C-(3,5-di-sec-butyl-1-cyclopenten-1-yl)pentonic acid-vs-Bacillus	0.084045	0.49892	0.63958
4-Deoxy-5-C-(3,5-di-sec-butyl-1-cyclopenten-1-yl)pentonic acid-vs-Dorea	0.13345	0.281656	0.44156
4-Deoxy-5-C-(3,5-di-sec-butyl-1-cyclopenten-1-yl)pentonic acid-vs-Tyzzerella	0.101125	0.415496	0.568166
4-Deoxy-5-C-(3,5-di-sec-butyl-1-cyclopenten-1-yl)pentonic acid-vs-Butyrivibrio	0.283502	0.020082	0.060345
4-Deoxy-5-C-(3,5-di-sec-butyl-1-cyclopenten-1-yl)pentonic acid-vs-Mycoplasma	-0.03871	0.755793	0.844073
4-Deoxy-5-C-(3,5-di-sec-butyl-1-cyclopenten-1-yl)pentonic acid-vs-Coprobacillus	-0.27376	0.02498	0.071343
4-Deoxy-5-C-(3,5-di-sec-butyl-1-cyclopenten-1-yl)pentonic acid-vs-Selenomonas	0.18098	0.142746	0.267677
4-Deoxy-5-C-(3,5-di-sec-butyl-1-cyclopenten-1-yl)pentonic acid-vs-Anaerostipes	0.105555	0.395256	0.551649
4-Deoxy-5-C-(3,5-di-sec-butyl-1-cyclopenten-1-yl)pentonic acid-vs-Peptoclostridium	0.213026	0.083487	0.181614

4-Deoxy-5-C-(3,5-di-sec-butyl-1-cyclopenten-1-yl)pentonic acid-vs-Dysgonomonas	-0.07523	0.545167	0.678292
4-Deoxy-5-C-(3,5-di-sec-butyl-1-cyclopenten-1-yl)pentonic acid-vs-Coprobacter	0.171881	0.164284	0.298575
4-Deoxy-5-C-(3,5-di-sec-butyl-1-cyclopenten-1-yl)pentonic acid-vs-Capnocytophaga	-0.12048	0.331469	0.492218
4-Deoxy-5-C-(3,5-di-sec-butyl-1-cyclopenten-1-yl)pentonic acid-vs-Campylobacter	-0.03312	0.790166	0.866067
4-Deoxy-5-C-(3,5-di-sec-butyl-1-cyclopenten-1-yl)pentonic acid-vs-Flavobacterium	0.083526	0.501583	0.642313
Uracil-vs-Prevotella	0.210552	0.087222	0.187548
Uracil-vs-Alistipes	0.309682	0.010767	0.037421
Uracil-vs-Faecalibacterium	0.389057	0.001138	0.006375
Uracil-vs-Oscillibacter	0.442892	0.000175	0.001446
Uracil-vs-Subdoligranulum	0.279831	0.021823	0.064693
Uracil-vs-Bilophila	0.316665	0.009033	0.032372
Uracil-vs-Anaerotruncus	0.407974	0.00061	0.003906
Uracil-vs-Butyricicoccus	0.552159	1.27E-06	0.000044
Uracil-vs-Paraprevotella	0.137361	0.267665	0.428229
Uracil-vs-Collinsella	0.378522	0.001586	0.008234
Uracil-vs-Parasutterella	0.105834	0.393999	0.551234
Uracil-vs-Pyramidobacter	0.223921	0.068518	0.156217
Uracil-vs-Intestinimonas	0.42661	0.000318	0.002369
Uracil-vs-Pseudoflavonifractor	0.4656	7.18E-05	0.000808
Uracil-vs-Holdemania	0.323849	0.007509	0.027988

Uracil-vs-Oribacterium	0.256525	0.036132	0.095212
Uracil-vs-Bacteroides	-0.31918	0.008471	0.030723
Uracil-vs-Eubacterium	-0.00219	0.985936	0.991108
Uracil-vs-Veillonella	-0.39959	0.000808	0.004974
Uracil-vs-Lactobacillus	-0.19227	0.119045	0.236817
Uracil-vs-Agathobacter	-0.09231	0.457527	0.607219
Uracil-vs-Enterococcus	-0.18345	0.137273	0.261221
Uracil-vs-Blautia	-0.11166	0.368332	0.527841
Uracil-vs-Streptococcus	-0.31024	0.010618	0.037064
Uracil-vs-Erysipelatoclostridium	-0.30793	0.011246	0.038641
Uracil-vs-Lachnospira	-0.21885	0.075189	0.167788
Uracil-vs-Fusobacterium	-0.16446	0.183554	0.323627
Uracil-vs-Bacillus	-0.0095	0.939194	0.964423
Uracil-vs-Dorea	-0.11661	0.347352	0.507943
Uracil-vs-Tyzzerella	-0.06449	0.604122	0.72705
Uracil-vs-Butyrivibrio	0.019395	0.876205	0.922341
Uracil-vs-Mycoplasma	-0.10144	0.414018	0.566785
Uracil-vs-Coprobacillus	-0.41472	0.000484	0.003338
Uracil-vs-Selenomonas	-0.02111	0.86535	0.915296
Uracil-vs-Anaerostipes	-0.1487	0.229788	0.381508
Uracil-vs-Peptoclostridium	0.023027	0.853262	0.905678
Uracil-vs-Dysgonomonas	-0.17108	0.166281	0.300624
Uracil-vs-Coprobacter	-0.05719	0.645756	0.759399
Uracil-vs-Capnocytophaga	-0.12327	0.320296	0.480941
Uracil-vs-Campylobacter	-0.15492	0.210647	0.358539

Uracil-vs-Flavobacterium	-0.05336	0.66805	0.775438
6-Acetamido-2-oxohexanoic acid-vs-Prevotella	-0.01381	0.911695	0.946214
6-Acetamido-2-oxohexanoic acid-vs-Alistipes	0.378322	0.001596	0.008267
6-Acetamido-2-oxohexanoic acid-vs-Faecalibacterium	0.316945	0.008969	0.032319
6-Acetamido-2-oxohexanoic acid-vs-Oscillibacter	0.47394	0.000051	0.000631
6-Acetamido-2-oxohexanoic acid-vs-Subdoligranulum	0.407894	0.000612	0.003906
6-Acetamido-2-oxohexanoic acid-vs-Bilophila	0.276598	0.023461	0.068292
6-Acetamido-2-oxohexanoic acid-vs-Anaerotruncus	0.431878	0.000263	0.002013
6-Acetamido-2-oxohexanoic acid-vs-Butyricicoccus	0.586839	1.8E-07	1.08E-05
6-Acetamido-2-oxohexanoic acid-vs-Paraprevotella	0.080693	0.516255	0.653149
6-Acetamido-2-oxohexanoic acid-vs-Collinsella	0.43539	0.000231	0.00181
6-Acetamido-2-oxohexanoic acid-vs-Parasutterella	0.18118	0.142299	0.267045
6-Acetamido-2-oxohexanoic acid-vs-Pyramidobacter	0.420784	0.000392	0.002779
6-Acetamido-2-oxohexanoic acid-vs-Intestinimonas	0.55623	1.02E-06	3.81E-05
6-Acetamido-2-oxohexanoic acid-vs-Pseudoflavonifractor	0.63377	8.58E-09	1.48E-06
6-Acetamido-2-oxohexanoic acid-vs-Holdemania	0.457698	9.85E-05	0.000993
6-Acetamido-2-oxohexanoic acid-vs-Oribacterium	0.333666	0.005792	0.022635
6-Acetamido-2-oxohexanoic acid-vs-Bacteroides	-0.03233	0.795111	0.869911
6-Acetamido-2-oxohexanoic acid-vs-Eubacterium	0.043778	0.725013	0.822997
6-Acetamido-2-oxohexanoic acid-vs-Veillonella	-0.34815	0.003889	0.016621
6-Acetamido-2-oxohexanoic acid-vs-Lactobacillus	-0.03931	0.752137	0.842323
6-Acetamido-2-oxohexanoic acid-vs-Agathobacter	-0.12435	0.316052	0.476046
6-Acetamido-2-oxohexanoic acid-vs-Enterococcus	-0.10851	0.382092	0.541148
6-Acetamido-2-oxohexanoic acid-vs-Blautia	-0.1032	0.405941	0.558255
6-Acetamido-2-oxohexanoic acid-vs-Streptococcus	-0.30872	0.011026	0.038048

6-Acetamido-2-oxohexanoic acid-vs-Erysipelatoclostridium	-0.12455	0.31527	0.47546
6-Acetamido-2-oxohexanoic acid-vs-Lachnospira	-0.01097	0.929763	0.958957
6-Acetamido-2-oxohexanoic acid-vs-Fusobacterium	-0.01093	0.930018	0.958957
6-Acetamido-2-oxohexanoic acid-vs-Bacillus	0.089552	0.471111	0.616677
6-Acetamido-2-oxohexanoic acid-vs-Dorea	0.036874	0.767038	0.851166
6-Acetamido-2-oxohexanoic acid-vs-Tyzzerella	-0.02897	0.815965	0.881956
6-Acetamido-2-oxohexanoic acid-vs-Butyrivibrio	0.109865	0.376133	0.535845
6-Acetamido-2-oxohexanoic acid-vs-Mycoplasma	0.026339	0.832441	0.893774
6-Acetamido-2-oxohexanoic acid-vs-Coprobacillus	-0.16354	0.186045	0.326119
6-Acetamido-2-oxohexanoic acid-vs-Selenomonas	0.00874	0.94404	0.966787
6-Acetamido-2-oxohexanoic acid-vs-Anaerostipes	-0.09929	0.424056	0.575895
6-Acetamido-2-oxohexanoic acid-vs-Peptoclostridium	0.109785	0.376482	0.536027
6-Acetamido-2-oxohexanoic acid-vs-Dysgonomonas	-0.00798	0.948888	0.969734
6-Acetamido-2-oxohexanoic acid-vs-Coprobacter	-0.01548	0.901026	0.939071
6-Acetamido-2-oxohexanoic acid-vs-Capnocytophaga	-0.19407	0.115571	0.23143
6-Acetamido-2-oxohexanoic acid-vs-Campylobacter	-0.16977	0.169616	0.305197
6-Acetamido-2-oxohexanoic acid-vs-Flavobacterium	0.022787	0.854771	0.906882
5-Hydroxyindoleacetate-vs-Prevotella	-0.03975	0.749459	0.840102
5-Hydroxyindoleacetate-vs-Alistipes	0.383351	0.001364	0.00736
5-Hydroxyindoleacetate-vs-Faecalibacterium	0.444609	0.000164	0.001403
5-Hydroxyindoleacetate-vs-Oscillibacter	0.509099	1.09E-05	0.000205
5-Hydroxyindoleacetate-vs-Subdoligranulum	0.308484	0.011091	0.038165
5-Hydroxyindoleacetate-vs-Bilophila	0.316186	0.009144	0.032671
5-Hydroxyindoleacetate-vs-Anaerotruncus	0.480006	3.95E-05	0.000516
5-Hydroxyindoleacetate-vs-Butyricicoccus	0.492457	2.31E-05	0.000349

5-Hydroxyindoleacetate-vs-Paraprevotella	0.0433	0.727904	0.823348
5-Hydroxyindoleacetate-vs-Collinsella	0.174435	0.158011	0.28891
5-Hydroxyindoleacetate-vs-Parasutterella	0.273326	0.025223	0.071614
5-Hydroxyindoleacetate-vs-Pyramidobacter	0.492617	2.29E-05	0.000348
5-Hydroxyindoleacetate-vs-Intestinimonas	0.59067	1.43E-07	0.00001
5-Hydroxyindoleacetate-vs-Pseudoflavonifractor	0.579695	2.74E-07	1.33E-05
5-Hydroxyindoleacetate-vs-Holdemania	0.377404	0.001642	0.008469
5-Hydroxyindoleacetate-vs-Oribacterium	0.290646	0.017032	0.05323
5-Hydroxyindoleacetate-vs-Bacteroides	-0.1621	0.189995	0.330645
5-Hydroxyindoleacetate-vs-Eubacterium	0.125868	0.31014	0.470658
5-Hydroxyindoleacetate-vs-Veillonella	-0.22651	0.065293	0.15028
5-Hydroxyindoleacetate-vs-Lactobacillus	0.017719	0.88683	0.930277
5-Hydroxyindoleacetate-vs-Agathobacter	0.005627	0.963954	0.978106
5-Hydroxyindoleacetate-vs-Enterococcus	-0.16326	0.186809	0.326746
5-Hydroxyindoleacetate-vs-Blautia	-0.08033	0.51813	0.654837
5-Hydroxyindoleacetate-vs-Streptococcus	-0.25409	0.037999	0.09948
5-Hydroxyindoleacetate-vs-Erysipelatoclostridium	-0.1813	0.142031	0.266916
5-Hydroxyindoleacetate-vs-Lachnospira	-0.1046	0.399582	0.554488
5-Hydroxyindoleacetate-vs-Fusobacterium	-0.03703	0.766059	0.851166
5-Hydroxyindoleacetate-vs-Bacillus	0.015205	0.902803	0.939304
5-Hydroxyindoleacetate-vs-Dorea	-0.00395	0.974687	0.985277
5-Hydroxyindoleacetate-vs-Tyzzerella	0.075425	0.544098	0.677742
5-Hydroxyindoleacetate-vs-Butyrivibrio	0.20688	0.093007	0.198399
5-Hydroxyindoleacetate-vs-Mycoplasma	-0.01177	0.92467	0.956296
5-Hydroxyindoleacetate-vs-Coprobacillus	-0.19798	0.108277	0.221592

5-Hydroxyindoleacetate-vs-Selenomonas	0.128901	0.298531	0.459966
5-Hydroxyindoleacetate-vs-Anaerostipes	-0.02626	0.832941	0.893915
5-Hydroxyindoleacetate-vs-Peptoclostridium	0.182377	0.139637	0.263685
5-Hydroxyindoleacetate-vs-Dysgonomonas	-0.03564	0.774644	0.855255
5-Hydroxyindoleacetate-vs-Coprobacter	0.119682	0.334705	0.495503
5-Hydroxyindoleacetate-vs-Capnocytophaga	-0.15855	0.200016	0.34388
5-Hydroxyindoleacetate-vs-Campylobacter	-0.17152	0.165181	0.299486
5-Hydroxyindoleacetate-vs-Flavobacterium	-0.08153	0.511893	0.650351
Dihomo-gamma-linolenic acid-vs-Prevotella	-0.019	0.878733	0.923796
Dihomo-gamma-linolenic acid-vs-Alistipes	-0.09298	0.454213	0.604701
Dihomo-gamma-linolenic acid-vs-Faecalibacterium	0.072631	0.559154	0.689747
Dihomo-gamma-linolenic acid-vs-Oscillibacter	-0.06457	0.603673	0.726872
Dihomo-gamma-linolenic acid-vs-Subdoligranulum	-0.1827	0.138933	0.263511
Dihomo-gamma-linolenic acid-vs-Bilophila	0.027257	0.82669	0.889844
Dihomo-gamma-linolenic acid-vs-Anaerotruncus	-0.16809	0.173931	0.310738
Dihomo-gamma-linolenic acid-vs-Butyricicoccus	0.006585	0.957824	0.974748
Dihomo-gamma-linolenic acid-vs-Paraprevotella	0.033123	0.790166	0.866067
Dihomo-gamma-linolenic acid-vs-Collinsella	0.095459	0.442241	0.594323
Dihomo-gamma-linolenic acid-vs-Parasutterella	0.172959	0.161615	0.29461
Dihomo-gamma-linolenic acid-vs-Pyramidobacter	-0.13149	0.28883	0.449891
Dihomo-gamma-linolenic acid-vs-Intestinimonas	-0.03105	0.803039	0.874629
Dihomo-gamma-linolenic acid-vs-Pseudoflavonifractor	-0.01006	0.935625	0.961868
Dihomo-gamma-linolenic acid-vs-Holdemania	-0.14119	0.254419	0.41388
Dihomo-gamma-linolenic acid-vs-Oribacterium	-0.13764	0.266684	0.428074
Dihomo-gamma-linolenic acid-vs-Bacteroides	0.017958	0.88531	0.929096

Dihomo-gamma-linolenic acid-vs-Eubacterium	-0.35071	0.003618	0.015742
Dihomo-gamma-linolenic acid-vs-Veillonella	-0.16901	0.171558	0.307548
Dihomo-gamma-linolenic acid-vs-Lactobacillus	-0.34636	0.00409	0.017266
Dihomo-gamma-linolenic acid-vs-Agathobacter	-0.38351	0.001358	0.00736
Dihomo-gamma-linolenic acid-vs-Enterococcus	-0.42026	0.000399	0.002798
Dihomo-gamma-linolenic acid-vs-Blautia	-0.03628	0.770716	0.853136
Dihomo-gamma-linolenic acid-vs-Streptococcus	-0.40394	0.000699	0.004371
Dihomo-gamma-linolenic acid-vs-Erysipelatoclostridium	-0.09302	0.454019	0.604701
Dihomo-gamma-linolenic acid-vs-Lachnospira	-0.33742	0.005234	0.02103
Dihomo-gamma-linolenic acid-vs-Fusobacterium	-0.35761	0.002968	0.013472
Dihomo-gamma-linolenic acid-vs-Bacillus	-0.43778	0.000212	0.001672
Dihomo-gamma-linolenic acid-vs-Dorea	-0.32521	0.007248	0.027352
Dihomo-gamma-linolenic acid-vs-Tyzzerella	-0.00946	0.939449	0.964423
Dihomo-gamma-linolenic acid-vs-Butyrivibrio	-0.44429	0.000166	0.001403
Dihomo-gamma-linolenic acid-vs-Mycoplasma	-0.32397	0.007486	0.027944
Dihomo-gamma-linolenic acid-vs-Coprobacillus	-0.35813	0.002924	0.013345
Dihomo-gamma-linolenic acid-vs-Selenomonas	-0.3359	0.005453	0.021743
Dihomo-gamma-linolenic acid-vs-Anaerostipes	-0.17404	0.158979	0.290241
Dihomo-gamma-linolenic acid-vs-Peptoclostridium	-0.1295	0.296274	0.457654
Dihomo-gamma-linolenic acid-vs-Dysgonomonas	-0.33395	0.005749	0.022502
Dihomo-gamma-linolenic acid-vs-Coprobacter	-0.27408	0.024805	0.070926
Dihomo-gamma-linolenic acid-vs-Capnocytophaga	-0.18649	0.130781	0.251279
Dihomo-gamma-linolenic acid-vs-Campylobacter	-0.16793	0.174346	0.31102
Dihomo-gamma-linolenic acid-vs-Flavobacterium	-0.50395	1.38E-05	0.000243
1-(beta-D-ribofuranosyl)thymine-vs-Prevotella	0.085282	0.4926	0.634843

1-(beta-D-ribofuranosyl)thymine-vs-Alistipes	0.366749	0.002269	0.01091
1-(beta-D-ribofuranosyl)thymine-vs-Faecalibacterium	0.472344	5.45E-05	0.000662
1-(beta-D-ribofuranosyl)thymine-vs-Oscillibacter	0.52175	5.96E-06	0.000131
1-(beta-D-ribofuranosyl)thymine-vs-Subdoligranulum	0.344601	0.004295	0.017945
1-(beta-D-ribofuranosyl)thymine-vs-Bilophila	0.356094	0.003101	0.013893
1-(beta-D-ribofuranosyl)thymine-vs-Anaerotruncus	0.339452	0.004951	0.020094
1-(beta-D-ribofuranosyl)thymine-vs-Butyricicoccus	0.457139	0.000101	0.001003
1-(beta-D-ribofuranosyl)thymine-vs-Paraprevotella	0.247945	0.04307	0.108414
1-(beta-D-ribofuranosyl)thymine-vs-Collinsella	0.290925	0.016921	0.052952
1-(beta-D-ribofuranosyl)thymine-vs-Parasutterella	0.092226	0.457918	0.607219
1-(beta-D-ribofuranosyl)thymine-vs-Pyramidobacter	0.299226	0.013901	0.045196
1-(beta-D-ribofuranosyl)thymine-vs-Intestinimonas	0.397518	0.000865	0.005181
1-(beta-D-ribofuranosyl)thymine-vs-Pseudoflavonifractor	0.411805	0.000536	0.003559
1-(beta-D-ribofuranosyl)thymine-vs-Holdemania	0.447801	0.000145	0.001284
1-(beta-D-ribofuranosyl)thymine-vs-Oribacterium	0.399234	0.000818	0.005021
1-(beta-D-ribofuranosyl)thymine-vs-Bacteroides	-0.10595	0.393461	0.5508
1-(beta-D-ribofuranosyl)thymine-vs-Eubacterium	-0.00527	0.966254	0.979207
1-(beta-D-ribofuranosyl)thymine-vs-Veillonella	-0.36623	0.002304	0.011015
1-(beta-D-ribofuranosyl)thymine-vs-Lactobacillus	-0.20034	0.10406	0.21478
1-(beta-D-ribofuranosyl)thymine-vs-Agathobacter	0.117567	0.343379	0.504025
1-(beta-D-ribofuranosyl)thymine-vs-Enterococcus	-0.19806	0.108132	0.221483
1-(beta-D-ribofuranosyl)thymine-vs-Blautia	-0.07966	0.521682	0.658637
1-(beta-D-ribofuranosyl)thymine-vs-Streptococcus	-0.23406	0.056604	0.133455
1-(beta-D-ribofuranosyl)thymine-vs-Erysipelatoclostridium	-0.23909	0.051352	0.123112
1-(beta-D-ribofuranosyl)thymine-vs-Lachnospira	-0.03061	0.805769	0.875238

1-(beta-D-ribofuranosyl)thymine-vs-Fusobacterium	0.028095	0.821448	0.885904
1-(beta-D-ribofuranosyl)thymine-vs-Bacillus	0.07347	0.554617	0.686601
1-(beta-D-ribofuranosyl)thymine-vs-Dorea	0.091548	0.461246	0.609369
1-(beta-D-ribofuranosyl)thymine-vs-Tyzzerella	-0.03903	0.753842	0.842673
1-(beta-D-ribofuranosyl)thymine-vs-Butyrivibrio	0.149493	0.227268	0.378885
1-(beta-D-ribofuranosyl)thymine-vs-Mycoplasma	-0.0587	0.637015	0.755363
1-(beta-D-ribofuranosyl)thymine-vs-Coprobacillus	-0.24371	0.046875	0.114306
1-(beta-D-ribofuranosyl)thymine-vs-Selenomonas	0.012451	0.920342	0.952898
1-(beta-D-ribofuranosyl)thymine-vs-Anaerostipes	-0.05779	0.6423	0.757914
1-(beta-D-ribofuranosyl)thymine-vs-Peptoclostridium	0.138239	0.264589	0.425843
1-(beta-D-ribofuranosyl)thymine-vs-Dysgonomonas	0.005986	0.961655	0.976593
1-(beta-D-ribofuranosyl)thymine-vs-Coprobacter	0.170484	0.167791	0.302901
1-(beta-D-ribofuranosyl)thymine-vs-Capnocytophaga	0.096736	0.436131	0.588072
1-(beta-D-ribofuranosyl)thymine-vs-Campylobacter	0.114055	0.358086	0.518068
1-(beta-D-ribofuranosyl)thymine-vs-Flavobacterium	0.15532	0.209459	0.357322
Oxaceprol-vs-Prevotella	0.034719	0.780301	0.859539
Oxaceprol-vs-Alistipes	0.399074	0.000822	0.005035
Oxaceprol-vs-Faecalibacterium	0.27588	0.023838	0.069017
Oxaceprol-vs-Oscillibacter	0.565767	6.07E-07	2.57E-05
Oxaceprol-vs-Subdoligranulum	0.385705	0.001267	0.00698
Oxaceprol-vs-Bilophila	0.304214	0.012319	0.041545
Oxaceprol-vs-Anaerotruncus	0.535957	2.95E-06	8.12E-05
Oxaceprol-vs-Butyricicoccus	0.562176	7.4E-07	3.02E-05
Oxaceprol-vs-Paraprevotella	0.193471	0.11672	0.233152
Oxaceprol-vs-Collinsella	0.467515	6.64E-05	0.000769

Oxaceprol-vs-Parasutterella	0.149972	0.225766	0.37716
Oxaceprol-vs-Pyramidobacter	0.456461	0.000103	0.001017
Oxaceprol-vs-Intestinimonas	0.642549	4.58E-09	9.24E-07
Oxaceprol-vs-Pseudoflavonifractor	0.656836	1.58E-09	4.25E-07
Oxaceprol-vs-Holdemania	0.443132	0.000173	0.001439
Oxaceprol-vs-Oribacterium	0.257044	0.035745	0.094706
Oxaceprol-vs-Bacteroides	-0.24348	0.047099	0.114735
Oxaceprol-vs-Eubacterium	0.031487	0.800311	0.872837
Oxaceprol-vs-Veillonella	-0.46245	8.15E-05	0.000873
Oxaceprol-vs-Lactobacillus	-0.02969	0.811485	0.878291
Oxaceprol-vs-Agathobacter	-0.14706	0.235013	0.389327
Oxaceprol-vs-Enterococcus	-0.16055	0.194341	0.336227
Oxaceprol-vs-Blautia	-0.17276	0.162107	0.295062
Oxaceprol-vs-Streptococcus	-0.33953	0.00494	0.020084
Oxaceprol-vs-Erysipelatoclostridium	-0.15149	0.221055	0.371857
Oxaceprol-vs-Lachnospira	-0.20568	0.094957	0.201492
Oxaceprol-vs-Fusobacterium	-0.18278	0.138758	0.263511
Oxaceprol-vs-Bacillus	0.08205	0.509202	0.648636
Oxaceprol-vs-Dorea	-0.1121	0.366441	0.526378
Oxaceprol-vs-Tyzzerella	-0.12435	0.316052	0.476046
Oxaceprol-vs-Butyrivibrio	0.080453	0.517505	0.654388
Oxaceprol-vs-Mycoplasma	0.04326	0.728145	0.823348
Oxaceprol-vs-Coprobacillus	-0.25309	0.038787	0.100779
Oxaceprol-vs-Selenomonas	0.047729	0.701314	0.803257
Oxaceprol-vs-Anaerostipes	-0.05579	0.653849	0.764457

Oxaceprol-vs-Peptoclostridium	0.011533	0.926197	0.957466
Oxaceprol-vs-Dysgonomonas	-0.10412	0.401756	0.555208
Oxaceprol-vs-Coprobacter	0.068481	0.58188	0.712963
Oxaceprol-vs-Capnocytophaga	-0.09925	0.424243	0.575895
Oxaceprol-vs-Campylobacter	-0.0421	0.735147	0.828282
Oxaceprol-vs-Flavobacterium	-0.05272	0.671794	0.777545
Cinnamic acid-vs-Prevotella	0.189201	0.125173	0.24478
Cinnamic acid-vs-Alistipes	0.181259	0.14212	0.266916
Cinnamic acid-vs-Faecalibacterium	0.468274	6.44E-05	0.000752
Cinnamic acid-vs-Oscillibacter	0.368345	0.002163	0.010455
Cinnamic acid-vs-Subdoligranulum	0.129819	0.295075	0.456676
Cinnamic acid-vs-Bilophila	0.132533	0.285009	0.445372
Cinnamic acid-vs-Anaerotruncus	0.334464	0.005669	0.022286
Cinnamic acid-vs-Butyricicoccus	0.549685	1.45E-06	0.000048
Cinnamic acid-vs-Paraprevotella	0.122356	0.32394	0.485208
Cinnamic acid-vs-Collinsella	0.420943	0.00039	0.002779
Cinnamic acid-vs-Parasutterella	0.1898	0.12396	0.243196
Cinnamic acid-vs-Pyramidobacter	0.249342	0.041871	0.106505
Cinnamic acid-vs-Intestinimonas	0.384827	0.001302	0.007127
Cinnamic acid-vs-Pseudoflavonifractor	0.376447	0.001691	0.008667
Cinnamic acid-vs-Holdemania	0.324687	0.007347	0.027553
Cinnamic acid-vs-Oribacterium	0.247266	0.043662	0.109425
Cinnamic acid-vs-Bacteroides	-0.22995	0.061215	0.142657
Cinnamic acid-vs-Eubacterium	0.085402	0.491991	0.634657
Cinnamic acid-vs-Veillonella	-0.32225	0.007827	0.028876

Cinnamic acid-vs-Lactobacillus	-0.1307	0.291793	0.453047
Cinnamic acid-vs-Agathobacter	-0.0016	0.989771	0.993312
Cinnamic acid-vs-Enterococcus	-0.24443	0.04621	0.11337
Cinnamic acid-vs-Blautia	-0.19431	0.115114	0.231241
Cinnamic acid-vs-Streptococcus	-0.21813	0.076175	0.169363
Cinnamic acid-vs-Erysipelatoclostridium	-0.3602	0.002752	0.012682
Cinnamic acid-vs-Lachnospira	-0.01233	0.921106	0.953017
Cinnamic acid-vs-Fusobacterium	-0.27233	0.025782	0.072774
Cinnamic acid-vs-Bacillus	-0.10492	0.398137	0.554071
Cinnamic acid-vs-Dorea	-0.00363	0.976732	0.985543
Cinnamic acid-vs-Tyzzerella	-0.05316	0.669219	0.77605
Cinnamic acid-vs-Butyrivibrio	0.036396	0.76998	0.853136
Cinnamic acid-vs-Mycoplasma	-0.15516	0.209934	0.357879
Cinnamic acid-vs-Coprobacillus	-0.449	0.000138	0.001253
Cinnamic acid-vs-Selenomonas	0.020512	0.869134	0.918358
Cinnamic acid-vs-Anaerostipes	-0.0767	0.537282	0.672633
Cinnamic acid-vs-Peptoclostridium	0.033003	0.790907	0.866487
Cinnamic acid-vs-Dysgonomonas	-0.2515	0.040075	0.102886
Cinnamic acid-vs-Coprobacter	0.063373	0.610415	0.731982
Cinnamic acid-vs-Capnocytophaga	-0.27157	0.026214	0.073821
Cinnamic acid-vs-Campylobacter	-0.17068	0.167287	0.302216
Cinnamic acid-vs-Flavobacterium	-0.02099	0.866106	0.915696
2,3,4-Trihydroxybutanoic acid-vs-Prevotella	0.135965	0.272607	0.433087
2,3,4-Trihydroxybutanoic acid-vs-Alistipes	0.387341	0.001203	0.006672
2,3,4-Trihydroxybutanoic acid-vs-Faecalibacterium	0.43096	0.000272	0.002075

2,3,4-Trihydroxybutanoic acid-vs-Oscillibacter	0.52155	6.02E-06	0.000131
2,3,4-Trihydroxybutanoic acid-vs-Subdoligranulum	0.399713	0.000805	0.004967
2,3,4-Trihydroxybutanoic acid-vs-Bilophila	0.39209	0.001033	0.005945
2,3,4-Trihydroxybutanoic acid-vs-Anaerotruncus	0.486591	2.98E-05	0.000424
2,3,4-Trihydroxybutanoic acid-vs-Butyricicoccus	0.544257	1.93E-06	5.84E-05
2,3,4-Trihydroxybutanoic acid-vs-Paraprevotella	0.244114	0.046505	0.113631
2,3,4-Trihydroxybutanoic acid-vs-Collinsella	0.438423	0.000207	0.001649
2,3,4-Trihydroxybutanoic acid-vs-Parasutterella	0.190438	0.122677	0.241658
2,3,4-Trihydroxybutanoic acid-vs-Pyramidobacter	0.410488	0.00056	0.003663
2,3,4-Trihydroxybutanoic acid-vs-Intestinimonas	0.488547	2.74E-05	0.000401
2,3,4-Trihydroxybutanoic acid-vs-Pseudoflavonifractor	0.502594	1.46E-05	0.000248
2,3,4-Trihydroxybutanoic acid-vs-Holdemania	0.487669	2.84E-05	0.00041
2,3,4-Trihydroxybutanoic acid-vs-Oribacterium	0.244114	0.046505	0.113631
2,3,4-Trihydroxybutanoic acid-vs-Bacteroides	-0.26267	0.031757	0.085929
2,3,4-Trihydroxybutanoic acid-vs-Eubacterium	-0.08911	0.473297	0.618645
2,3,4-Trihydroxybutanoic acid-vs-Veillonella	-0.55922	8.7E-07	3.34E-05
2,3,4-Trihydroxybutanoic acid-vs-Lactobacillus	-0.24639	0.044439	0.110912
2,3,4-Trihydroxybutanoic acid-vs-Agathobacter	-0.07495	0.546665	0.679539
2,3,4-Trihydroxybutanoic acid-vs-Enterococcus	-0.3292	0.006525	0.025095
2,3,4-Trihydroxybutanoic acid-vs-Blautia	-0.16438	0.183769	0.32368
2,3,4-Trihydroxybutanoic acid-vs-Streptococcus	-0.3703	0.002039	0.010018
2,3,4-Trihydroxybutanoic acid-vs-Erysipelatoclostridium	-0.23366	0.057039	0.134219
2,3,4-Trihydroxybutanoic acid-vs-Lachnospira	-0.10651	0.390957	0.549066
2,3,4-Trihydroxybutanoic acid-vs-Fusobacterium	-0.20377	0.098144	0.206263
2,3,4-Trihydroxybutanoic acid-vs-Bacillus	-0.05914	0.634494	0.752742

2,3,4-Trihydroxybutanoic acid-vs-Dorea	-0.14043	0.257005	0.417793
2,3,4-Trihydroxybutanoic acid-vs-Tyzzerella	-0.12962	0.295824	0.457251
2,3,4-Trihydroxybutanoic acid-vs-Butyrivibrio	0.041504	0.738777	0.830052
2,3,4-Trihydroxybutanoic acid-vs-Mycoplasma	-0.11813	0.341074	0.502471
2,3,4-Trihydroxybutanoic acid-vs-Coprobacillus	-0.36208	0.002605	0.012166
2,3,4-Trihydroxybutanoic acid-vs-Selenomonas	-0.11948	0.335518	0.496099
2,3,4-Trihydroxybutanoic acid-vs-Anaerostipes	-0.21618	0.078911	0.174166
2,3,4-Trihydroxybutanoic acid-vs-Peptoclostridium	0.04322	0.728386	0.823348
2,3,4-Trihydroxybutanoic acid-vs-Dysgonomonas	-0.18685	0.130028	0.250229
2,3,4-Trihydroxybutanoic acid-vs-Coprobacter	0.05599	0.652691	0.764013
2,3,4-Trihydroxybutanoic acid-vs-Capnocytophaga	-0.11713	0.345196	0.506078
2,3,4-Trihydroxybutanoic acid-vs-Campylobacter	-0.24324	0.047323	0.114934
2,3,4-Trihydroxybutanoic acid-vs-Flavobacterium	-0.08903	0.473695	0.618645
Calcitriol-vs-Prevotella	0.106074	0.392924	0.550367
Calcitriol-vs-Alistipes	0.393966	0.000972	0.00572
Calcitriol-vs-Faecalibacterium	0.408772	0.000594	0.003842
Calcitriol-vs-Oscillibacter	0.530489	3.88E-06	9.78E-05
Calcitriol-vs-Subdoligranulum	0.333267	0.005854	0.022841
Calcitriol-vs-Bilophila	0.397079	0.000878	0.005231
Calcitriol-vs-Anaerotruncus	0.462567	8.11E-05	0.000872
Calcitriol-vs-Butyricicoccus	0.460292	8.89E-05	0.000915
Calcitriol-vs-Paraprevotella	0.145263	0.240842	0.396593
Calcitriol-vs-Collinsella	0.24555	0.045191	0.111549
Calcitriol-vs-Parasutterella	0.298867	0.014021	0.045343
Calcitriol-vs-Pyramidobacter	0.37952	0.001538	0.008105

Calcitriol-vs-Intestinimonas	0.510775	0.00001	0.000194
Calcitriol-vs-Pseudoflavonifractor	0.482361	3.57E-05	0.000481
Calcitriol-vs-Holdemania	0.356094	0.003101	0.013893
Calcitriol-vs-Oribacterium	0.234217	0.056431	0.133176
Calcitriol-vs-Bacteroides	-0.1914	0.120772	0.239229
Calcitriol-vs-Eubacterium	-0.03516	0.777594	0.857729
Calcitriol-vs-Veillonella	-0.50347	1.41E-05	0.000245
Calcitriol-vs-Lactobacillus	-0.30769	0.011312	0.03876
Calcitriol-vs-Agathobacter	-0.16194	0.190437	0.330939
Calcitriol-vs-Enterococcus	-0.29771	0.014415	0.046308
Calcitriol-vs-Blautia	-0.20405	0.097674	0.205812
Calcitriol-vs-Streptococcus	-0.45642	0.000104	0.001017
Calcitriol-vs-Erysipelatoclostridium	-0.34739	0.003973	0.01686
Calcitriol-vs-Lachnospira	-0.21159	0.08564	0.185133
Calcitriol-vs-Fusobacterium	-0.30174	0.013083	0.043176
Calcitriol-vs-Bacillus	-0.13708	0.268649	0.429518
Calcitriol-vs-Dorea	-0.29156	0.01667	0.052302
Calcitriol-vs-Tyzzerella	-0.12738	0.3043	0.46612
Calcitriol-vs-Butyrivibrio	0.00866	0.94455	0.966787
Calcitriol-vs-Mycoplasma	-0.10643	0.391315	0.549066
Calcitriol-vs-Coprobacillus	-0.35693	0.003027	0.013687
Calcitriol-vs-Selenomonas	0.024344	0.844971	0.90282
Calcitriol-vs-Anaerostipes	-0.27863	0.022418	0.065972
Calcitriol-vs-Peptoclostridium	0.023865	0.847984	0.903116
Calcitriol-vs-Dysgonomonas	-0.06261	0.614702	0.735757

Calcitriol-vs-Coprobacter	-0.00156	0.990027	0.993312
Calcitriol-vs-Capnocytophaga	-0.16474	0.1828	0.323195
Calcitriol-vs-Campylobacter	-0.02758	0.824692	0.888611
Calcitriol-vs-Flavobacterium	-0.05539	0.656169	0.766329
(9E,12E,15E)-9,12,15-Octadecatrienoic acid-vs-Prevotella	0.248064	0.042966	0.108266
(9E,12E,15E)-9,12,15-Octadecatrienoic acid-vs-Alistipes	0.073909	0.552247	0.684368
(9E,12E,15E)-9,12,15-Octadecatrienoic acid-vs-Faecalibacterium	0.346077	0.004122	0.017341
(9E,12E,15E)-9,12,15-Octadecatrienoic acid-vs-Oscillibacter	0.223402	0.069179	0.157541
(9E,12E,15E)-9,12,15-Octadecatrienoic acid-vs-Subdoligranulum	0.05619	0.651533	0.763594
(9E,12E,15E)-9,12,15-Octadecatrienoic acid-vs-Bilophila	0.031487	0.800311	0.872837
(9E,12E,15E)-9,12,15-Octadecatrienoic acid-vs-Anaerotruncus	0.058345	0.639081	0.756601
(9E,12E,15E)-9,12,15-Octadecatrienoic acid-vs-Butyricicoccus	0.316905	0.008978	0.032319
(9E,12E,15E)-9,12,15-Octadecatrienoic acid-vs-Paraprevotella	0.12064	0.330824	0.491562
(9E,12E,15E)-9,12,15-Octadecatrienoic acid-vs-Collinsella	0.226155	0.065732	0.151003
(9E,12E,15E)-9,12,15-Octadecatrienoic acid-vs-Parasutterella	0.18541	0.13306	0.25505
(9E,12E,15E)-9,12,15-Octadecatrienoic acid-vs-Pyramidobacter	0.03895	0.75433	0.842828
(9E,12E,15E)-9,12,15-Octadecatrienoic acid-vs-Intestinimonas	0.105635	0.394897	0.551533

(9E,12E,15E)-9,12,15-Octadecatrienoic acid-vs-Pseudoflavonifractor	0.127863	0.30247	0.464261
(9E,12E,15E)-9,12,15-Octadecatrienoic acid-vs-Holdemania	0.089033	0.473695	0.618645
(9E,12E,15E)-9,12,15-Octadecatrienoic acid-vs-Oribacterium	0.067404	0.587848	0.716825
(9E,12E,15E)-9,12,15-Octadecatrienoic acid-vs-Bacteroides	-0.28861	0.017857	0.055239
(9E,12E,15E)-9,12,15-Octadecatrienoic acid-vs-Eubacterium	-0.19766	0.108859	0.222407
(9E,12E,15E)-9,12,15-Octadecatrienoic acid-vs-Veillonella	-0.34245	0.00456	0.018727
(9E,12E,15E)-9,12,15-Octadecatrienoic acid-vs-Lactobacillus	-0.35781	0.002951	0.013419
(9E,12E,15E)-9,12,15-Octadecatrienoic acid-vs-Agathobacter	-0.15987	0.196257	0.338138
(9E,12E,15E)-9,12,15-Octadecatrienoic acid-vs-Enterococcus	-0.42533	0.000333	0.002465
(9E,12E,15E)-9,12,15-Octadecatrienoic acid-vs-Blautia	-0.22244	0.070411	0.159266
(9E,12E,15E)-9,12,15-Octadecatrienoic acid-vs-Streptococcus	-0.27013	0.02705	0.075038
(9E,12E,15E)-9,12,15-Octadecatrienoic acid-vs-Erysipelatoclostridium	-0.4553	0.000108	0.001048
(9E,12E,15E)-9,12,15-Octadecatrienoic acid-vs-Lachnospira	-0.13453	0.277754	0.437142
(9E,12E,15E)-9,12,15-Octadecatrienoic acid-vs-Fusobacterium	-0.34065	0.004791	0.019544
(9E,12E,15E)-9,12,15-Octadecatrienoic acid-vs-Bacillus	-0.25269	0.039106	0.101389
(9E,12E,15E)-9,12,15-Octadecatrienoic acid-vs-Dorea	-0.08221	0.508376	0.648266
(9E,12E,15E)-9,12,15-Octadecatrienoic acid-vs-Tyzzerella	-0.09586	0.440327	0.593068
(9E,12E,15E)-9,12,15-Octadecatrienoic acid-vs-Butyrivibrio	-0.15293	0.216655	0.366495
(9E,12E,15E)-9,12,15-Octadecatrienoic acid-vs-Mycoplasma	-0.18752	0.128615	0.248856
(9E,12E,15E)-9,12,15-Octadecatrienoic acid-vs-Coprobacillus	-0.47538	0.000048	0.000611
(9E,12E,15E)-9,12,15-Octadecatrienoic acid-vs-Selenomonas	-0.20321	0.099088	0.207349
(9E,12E,15E)-9,12,15-Octadecatrienoic acid-vs-Anaerostipes	-0.21111	0.086368	0.186206

(9E,12E,15E)-9,12,15-Octadecatrienoic acid-vs-Peptoclostridium	-0.05292	0.670623	0.776934
(9E,12E,15E)-9,12,15-Octadecatrienoic acid-vs-Dysgonomonas	-0.50459	1.34E-05	0.000238
(9E,12E,15E)-9,12,15-Octadecatrienoic acid-vs-Coprobacter	-0.17017	0.168601	0.303908
(9E,12E,15E)-9,12,15-Octadecatrienoic acid-vs-Capnocytophaga	-0.16969	0.16982	0.305197
(9E,12E,15E)-9,12,15-Octadecatrienoic acid-vs-Campylobacter	-0.21945	0.074375	0.166741
(9E,12E,15E)-9,12,15-Octadecatrienoic acid-vs-Flavobacterium	-0.13333	0.282092	0.441898
15,16-Epoxy-6b,9-dihydroxy-8bH-labda-13(16),14-dien-19-oic Acid g-Lactone-vs-Prevotella	0.158752	0.199443	0.343139
15,16-Epoxy-6b,9-dihydroxy-8bH-labda-13(16),14-dien-19-oic Acid g-Lactone-vs-Alistipes	0.248503	0.042587	0.107534
15,16-Epoxy-6b,9-dihydroxy-8bH-labda-13(16),14-dien-19-oic Acid g-Lactone-vs-Faecalibacterium	0.537553	2.72E-06	7.57E-05
15,16-Epoxy-6b,9-dihydroxy-8bH-labda-13(16),14-dien-19-oic Acid g-Lactone-vs-Oscillibacter	0.455104	0.000109	0.001052
15,16-Epoxy-6b,9-dihydroxy-8bH-labda-13(16),14-dien-19-oic Acid g-Lactone-vs-Subdoligranulum	0.325764	0.007143	0.027209
15,16-Epoxy-6b,9-dihydroxy-8bH-labda-13(16),14-dien-19-oic Acid g-Lactone-vs-Bilophila	0.36224	0.002593	0.012132
15,16-Epoxy-6b,9-dihydroxy-8bH-labda-13(16),14-dien-19-oic Acid g-Lactone-vs-Anaerotruncus	0.306489	0.011651	0.03964

15,16-Epoxy-6b,9-dihydroxy-8bH-labda-13(16),14-dien-19-oic Acid g-Lactone-vs-Butyricicoccus	0.456341	0.000104	0.001017
15,16-Epoxy-6b,9-dihydroxy-8bH-labda-13(16),14-dien-19-oic Acid g-Lactone-vs-Paraprevotella	0.200694	0.103428	0.213841
15,16-Epoxy-6b,9-dihydroxy-8bH-labda-13(16),14-dien-19-oic Acid g-Lactone-vs-Collinsella	0.343084	0.00448	0.018556
15,16-Epoxy-6b,9-dihydroxy-8bH-labda-13(16),14-dien-19-oic Acid g-Lactone-vs-Parasutterella	0.09067	0.465573	0.612409
15,16-Epoxy-6b,9-dihydroxy-8bH-labda-13(16),14-dien-19-oic Acid g-Lactone-vs-Pyramidobacter	0.153364	0.215323	0.364752
15,16-Epoxy-6b,9-dihydroxy-8bH-labda-13(16),14-dien-19-oic Acid g-Lactone-vs-Intestinimonas	0.241121	0.049342	0.118884
15,16-Epoxy-6b,9-dihydroxy-8bH-labda-13(16),14-dien-19-oic Acid g-Lactone-vs-Pseudoflavonifractor	0.248583	0.042518	0.107473
15,16-Epoxy-6b,9-dihydroxy-8bH-labda-13(16),14-dien-19-oic Acid g-Lactone-vs-Holdemania	0.44844	0.000141	0.00127
15,16-Epoxy-6b,9-dihydroxy-8bH-labda-13(16),14-dien-19-oic Acid g-Lactone-vs-Oribacterium	0.281946	0.020805	0.062286
15,16-Epoxy-6b,9-dihydroxy-8bH-labda-13(16),14-dien-19-oic Acid g-Lactone-vs-Bacteroides	-0.03041	0.807011	0.8758
15,16-Epoxy-6b,9-dihydroxy-8bH-labda-13(16),14-dien-19-oic Acid g-Lactone-vs-Eubacterium	-0.02985	0.81049	0.878291
15,16-Epoxy-6b,9-dihydroxy-8bH-labda-13(16),14-dien-19-oic Acid g-Lactone-vs-Veillonella	-0.56521	6.26E-07	2.61E-05

15,16-Epoxy-6b,9-dihydroxy-8bH-labda-13(16),14-dien-19-oic Acid g-Lactone-vs-Lactobacillus	-0.25525	0.037102	0.097236
15,16-Epoxy-6b,9-dihydroxy-8bH-labda-13(16),14-dien-19-oic Acid g-Lactone-vs-Agathobacter	0.066007	0.595626	0.721853
15,16-Epoxy-6b,9-dihydroxy-8bH-labda-13(16),14-dien-19-oic Acid g-Lactone-vs-Enterococcus	-0.32541	0.00721	0.027337
15,16-Epoxy-6b,9-dihydroxy-8bH-labda-13(16),14-dien-19-oic Acid g-Lactone-vs-Blautia	-0.12623	0.30875	0.470544
15,16-Epoxy-6b,9-dihydroxy-8bH-labda-13(16),14-dien-19-oic Acid g-Lactone-vs-Streptococcus	-0.32548	0.007195	0.027323
15,16-Epoxy-6b,9-dihydroxy-8bH-labda-13(16),14-dien-19-oic Acid g-Lactone-vs-Erysipelatoclostridium	-0.24276	0.047774	0.115657
15,16-Epoxy-6b,9-dihydroxy-8bH-labda-13(16),14-dien-19-oic Acid g-Lactone-vs-Lachnospira	0.169686	0.16982	0.305197
15,16-Epoxy-6b,9-dihydroxy-8bH-labda-13(16),14-dien-19-oic Acid g-Lactone-vs-Fusobacterium	-0.03903	0.753842	0.842673
15,16-Epoxy-6b,9-dihydroxy-8bH-labda-13(16),14-dien-19-oic Acid g-Lactone-vs-Bacillus	-0.0083	0.946847	0.968466
15,16-Epoxy-6b,9-dihydroxy-8bH-labda-13(16),14-dien-19-oic Acid g-Lactone-vs-Dorea	0.00431	0.972387	0.983774
15,16-Epoxy-6b,9-dihydroxy-8bH-labda-13(16),14-dien-19-oic Acid g-Lactone-vs-Tyzzerella	-0.13253	0.285009	0.445372
15,16-Epoxy-6b,9-dihydroxy-8bH-labda-13(16),14-dien-19-oic Acid g-Lactone-vs-Butyrivibrio	0.12918	0.297476	0.459218

15,16-Epoxy-6b,9-dihydroxy-8bH-labda-13(16),14-dien-19-oic Acid g-Lactone-vs-Mycoplasma	0.000359	0.997698	0.998524
15,16-Epoxy-6b,9-dihydroxy-8bH-labda-13(16),14-dien-19-oic Acid g-Lactone-vs-Coprobacillus	-0.20105	0.1028	0.213087
15,16-Epoxy-6b,9-dihydroxy-8bH-labda-13(16),14-dien-19-oic Acid g-Lactone-vs-Selenomonas	-0.09466	0.446084	0.597495
15,16-Epoxy-6b,9-dihydroxy-8bH-labda-13(16),14-dien-19-oic Acid g-Lactone-vs-Anaerostipes	-0.17603	0.154181	0.283366
15,16-Epoxy-6b,9-dihydroxy-8bH-labda-13(16),14-dien-19-oic Acid g-Lactone-vs-Peptoclostridium	0.036755	0.767773	0.851166
15,16-Epoxy-6b,9-dihydroxy-8bH-labda-13(16),14-dien-19-oic Acid g-Lactone-vs-Dysgonomonas	-0.21374	0.082426	0.17992
15,16-Epoxy-6b,9-dihydroxy-8bH-labda-13(16),14-dien-19-oic Acid g-Lactone-vs-Coprobacter	0.007822	0.949909	0.970368
15,16-Epoxy-6b,9-dihydroxy-8bH-labda-13(16),14-dien-19-oic Acid g-Lactone-vs-Capnocytophaga	-0.19176	0.120063	0.238254
15,16-Epoxy-6b,9-dihydroxy-8bH-labda-13(16),14-dien-19-oic Acid g-Lactone-vs-Campylobacter	-0.07519	0.545381	0.678292
15,16-Epoxy-6b,9-dihydroxy-8bH-labda-13(16),14-dien-19-oic Acid g-Lactone-vs-Flavobacterium	-0.06804	0.584308	0.71422
N-Acetyl-b-D-glucosamine-vs-Prevotella	0.120121	0.332923	0.494074
N-Acetyl-b-D-glucosamine-vs-Alistipes	0.261154	0.032793	0.088239
N-Acetyl-b-D-glucosamine-vs-Faecalibacterium	0.44389	0.000168	0.001408
N-Acetyl-b-D-glucosamine-vs-Oscillibacter	0.390534	0.001086	0.006137
N-Acetyl-b-D-glucosamine-vs-Subdoligranulum	0.363836	0.002474	0.011642

N-Acetyl-b-D-glucosamine-vs-Bilophila	0.280629	0.021434	0.063775
N-Acetyl-b-D-glucosamine-vs-Anaerotruncus	0.302339	0.012895	0.042829
N-Acetyl-b-D-glucosamine-vs-Butyricicoccus	0.552199	1.27E-06	0.000044
N-Acetyl-b-D-glucosamine-vs-Paraprevotella	0.057507	0.643912	0.758977
N-Acetyl-b-D-glucosamine-vs-Collinsella	0.383989	0.001337	0.007285
N-Acetyl-b-D-glucosamine-vs-Parasutterella	0.196424	0.111136	0.224219
N-Acetyl-b-D-glucosamine-vs-Pyramidobacter	0.356533	0.003062	0.013769
N-Acetyl-b-D-glucosamine-vs-Intestinimonas	0.398116	0.000848	0.005118
N-Acetyl-b-D-glucosamine-vs-Pseudoflavonifractor	0.433035	0.000252	0.001948
N-Acetyl-b-D-glucosamine-vs-Holdemania	0.451313	0.000127	0.001173
N-Acetyl-b-D-glucosamine-vs-Oribacterium	0.286016	0.018959	0.05798
N-Acetyl-b-D-glucosamine-vs-Bacteroides	-0.1827	0.138933	0.263511
N-Acetyl-b-D-glucosamine-vs-Eubacterium	0.011254	0.92798	0.95849
N-Acetyl-b-D-glucosamine-vs-Veillonella	-0.30058	0.013454	0.044099
N-Acetyl-b-D-glucosamine-vs-Lactobacillus	-0.14219	0.251044	0.409492
N-Acetyl-b-D-glucosamine-vs-Agathobacter	0.006665	0.957313	0.974748
N-Acetyl-b-D-glucosamine-vs-Enterococcus	-0.24308	0.047473	0.115067
N-Acetyl-b-D-glucosamine-vs-Blautia	-0.03911	0.753355	0.842673
N-Acetyl-b-D-glucosamine-vs-Streptococcus	-0.2331	0.057652	0.135267
N-Acetyl-b-D-glucosamine-vs-Erysipelatoclostridium	-0.25812	0.03495	0.092703
N-Acetyl-b-D-glucosamine-vs-Lachnospira	-0.01373	0.912203	0.946214
N-Acetyl-b-D-glucosamine-vs-Fusobacterium	-0.13744	0.267384	0.428229
N-Acetyl-b-D-glucosamine-vs-Bacillus	0.006585	0.957824	0.974748
N-Acetyl-b-D-glucosamine-vs-Dorea	0.088475	0.476486	0.620021
N-Acetyl-b-D-glucosamine-vs-Tyzzerella	-0.01078	0.931037	0.959453

N-Acetyl-b-D-glucosamine-vs-Butyrivibrio	0.066326	0.593844	0.720686
N-Acetyl-b-D-glucosamine-vs-Mycoplasma	-0.1647	0.182908	0.323195
N-Acetyl-b-D-glucosamine-vs-Coprobacillus	-0.28825	0.018007	0.055559
N-Acetyl-b-D-glucosamine-vs-Selenomonas	-0.03572	0.774153	0.855255
N-Acetyl-b-D-glucosamine-vs-Anaerostipes	-0.1133	0.361312	0.521487
N-Acetyl-b-D-glucosamine-vs-Peptoclostridium	0.083007	0.504253	0.644709
N-Acetyl-b-D-glucosamine-vs-Dysgonomonas	-0.10444	0.400306	0.554538
N-Acetyl-b-D-glucosamine-vs-Coprobacter	-0.04653	0.708467	0.809533
N-Acetyl-b-D-glucosamine-vs-Capnocytophaga	-0.1362	0.271756	0.432201
N-Acetyl-b-D-glucosamine-vs-Campylobacter	-0.11481	0.354878	0.514967
N-Acetyl-b-D-glucosamine-vs-Flavobacterium	-0.04358	0.726217	0.823348
(1S,3R,5Z,7E,24R)-9,10-Secocholesta-5,7,10-triene-1,3,24-triol hydrate (1:1)-vs-Prevotella	0.188124	0.127377	0.247451
(1S,3R,5Z,7E,24R)-9,10-Secocholesta-5,7,10-triene-1,3,24-triol hydrate (1:1)-vs-Alistipes	0.356533	0.003062	0.013769
(1S,3R,5Z,7E,24R)-9,10-Secocholesta-5,7,10-triene-1,3,24-triol hydrate (1:1)-vs-Faecalibacterium	0.473861	5.11E-05	0.000631
(1S,3R,5Z,7E,24R)-9,10-Secocholesta-5,7,10-triene-1,3,24-triol hydrate (1:1)-vs-Oscillibacter	0.560061	8.31E-07	3.24E-05
(1S,3R,5Z,7E,24R)-9,10-Secocholesta-5,7,10-triene-1,3,24-triol hydrate (1:1)-vs-Subdoligranulum	0.222763	0.069998	0.158545
(1S,3R,5Z,7E,24R)-9,10-Secocholesta-5,7,10-triene-1,3,24-triol hydrate (1:1)-vs-Bilophila	0.434751	0.000237	0.001847
(1S,3R,5Z,7E,24R)-9,10-Secocholesta-5,7,10-triene-1,3,24-triol hydrate (1:1)-vs-Anaerotruncus	0.461968	8.31E-05	0.000882

(1S,3R,5Z,7E,24R)-9,10-Secocholesta-5,7,10-triene-1,3,24-triol hydrate (1:1)-vs-Butyricicoccus	0.528933	4.2E-06	0.000103
(1S,3R,5Z,7E,24R)-9,10-Secocholesta-5,7,10-triene-1,3,24-triol hydrate (1:1)-vs-Paraprevotella	0.126068	0.309367	0.470544
(1S,3R,5Z,7E,24R)-9,10-Secocholesta-5,7,10-triene-1,3,24-triol hydrate (1:1)-vs-Collinsella	0.422699	0.000366	0.002655
(1S,3R,5Z,7E,24R)-9,10-Secocholesta-5,7,10-triene-1,3,24-triol hydrate (1:1)-vs-Parasutterella	0.332349	0.006	0.023372
(1S,3R,5Z,7E,24R)-9,10-Secocholesta-5,7,10-triene-1,3,24-triol hydrate (1:1)-vs-Pyramidobacter	0.285218	0.01931	0.058681
(1S,3R,5Z,7E,24R)-9,10-Secocholesta-5,7,10-triene-1,3,24-triol hydrate (1:1)-vs-Intestinimonas	0.453069	0.000118	0.001121
(1S,3R,5Z,7E,24R)-9,10-Secocholesta-5,7,10-triene-1,3,24-triol hydrate (1:1)-vs-Pseudoflavonifractor	0.37924	0.001551	0.008157
(1S,3R,5Z,7E,24R)-9,10-Secocholesta-5,7,10-triene-1,3,24-triol hydrate (1:1)-vs-Holdemania	0.430561	0.000276	0.002099
(1S,3R,5Z,7E,24R)-9,10-Secocholesta-5,7,10-triene-1,3,24-triol hydrate (1:1)-vs-Oribacterium	0.317184	0.008915	0.032234
(1S,3R,5Z,7E,24R)-9,10-Secocholesta-5,7,10-triene-1,3,24-triol hydrate (1:1)-vs-Bacteroides	-0.25174	0.03988	0.102518
(1S,3R,5Z,7E,24R)-9,10-Secocholesta-5,7,10-triene-1,3,24-triol hydrate (1:1)-vs-Eubacterium	0.035957	0.772679	0.854527
(1S,3R,5Z,7E,24R)-9,10-Secocholesta-5,7,10-triene-1,3,24-triol hydrate (1:1)-vs-Veillonella	-0.47462	4.95E-05	0.000624

(1S,3R,5Z,7E,24R)-9,10-Secocholesta-5,7,10-triene-1,3,24-triol hydrate (1:1)-vs-Lactobacillus	-0.2521	0.039588	0.102202
(1S,3R,5Z,7E,24R)-9,10-Secocholesta-5,7,10-triene-1,3,24-triol hydrate (1:1)-vs-Agathobacter	-0.00136	0.991305	0.99377
(1S,3R,5Z,7E,24R)-9,10-Secocholesta-5,7,10-triene-1,3,24-triol hydrate (1:1)-vs-Enterococcus	-0.33566	0.005489	0.021766
(1S,3R,5Z,7E,24R)-9,10-Secocholesta-5,7,10-triene-1,3,24-triol hydrate (1:1)-vs-Blautia	-0.15161	0.220685	0.371702
(1S,3R,5Z,7E,24R)-9,10-Secocholesta-5,7,10-triene-1,3,24-triol hydrate (1:1)-vs-Streptococcus	-0.39149	0.001053	0.005993
(1S,3R,5Z,7E,24R)-9,10-Secocholesta-5,7,10-triene-1,3,24-triol hydrate (1:1)-vs-Erysipelatoclostridium	-0.4144	0.00049	0.003365
(1S,3R,5Z,7E,24R)-9,10-Secocholesta-5,7,10-triene-1,3,24-triol hydrate (1:1)-vs-Lachnospira	-0.06409	0.606366	0.728663
(1S,3R,5Z,7E,24R)-9,10-Secocholesta-5,7,10-triene-1,3,24-triol hydrate (1:1)-vs-Fusobacterium	-0.1445	0.243332	0.399335
(1S,3R,5Z,7E,24R)-9,10-Secocholesta-5,7,10-triene-1,3,24-triol hydrate (1:1)-vs-Bacillus	-0.06497	0.601433	0.726337
(1S,3R,5Z,7E,24R)-9,10-Secocholesta-5,7,10-triene-1,3,24-triol hydrate (1:1)-vs-Dorea	0.000838	0.99463	0.996277
(1S,3R,5Z,7E,24R)-9,10-Secocholesta-5,7,10-triene-1,3,24-triol hydrate (1:1)-vs-Tyzzerella	-0.02035	0.870143	0.918358
(1S,3R,5Z,7E,24R)-9,10-Secocholesta-5,7,10-triene-1,3,24-triol hydrate (1:1)-vs-Butyrivibrio	0.110504	0.373348	0.533382

(1S,3R,5Z,7E,24R)-9,10-Secocholesta-5,7,10-triene-1,3,24-triol hydrate (1:1)-vs-Mycoplasma	-0.08181	0.510443	0.649191
(1S,3R,5Z,7E,24R)-9,10-Secocholesta-5,7,10-triene-1,3,24-triol hydrate (1:1)-vs-Coprobacillus	-0.22532	0.066766	0.152943
(1S,3R,5Z,7E,24R)-9,10-Secocholesta-5,7,10-triene-1,3,24-triol hydrate (1:1)-vs-Selenomonas	-0.18349	0.137186	0.261221
(1S,3R,5Z,7E,24R)-9,10-Secocholesta-5,7,10-triene-1,3,24-triol hydrate (1:1)-vs-Anaerostipes	-0.07606	0.540685	0.675577
(1S,3R,5Z,7E,24R)-9,10-Secocholesta-5,7,10-triene-1,3,24-triol hydrate (1:1)-vs-Peptoclostridium	0.06481	0.602329	0.726337
(1S,3R,5Z,7E,24R)-9,10-Secocholesta-5,7,10-triene-1,3,24-triol hydrate (1:1)-vs-Dysgonomonas	-0.33215	0.006032	0.023384
(1S,3R,5Z,7E,24R)-9,10-Secocholesta-5,7,10-triene-1,3,24-triol hydrate (1:1)-vs-Coprobacter	-0.06333	0.610641	0.731982
(1S,3R,5Z,7E,24R)-9,10-Secocholesta-5,7,10-triene-1,3,24-triol hydrate (1:1)-vs-Capnocytophaga	-0.22336	0.06923	0.157541
(1S,3R,5Z,7E,24R)-9,10-Secocholesta-5,7,10-triene-1,3,24-triol hydrate (1:1)-vs-Campylobacter	0.047889	0.700362	0.802547
(1S,3R,5Z,7E,24R)-9,10-Secocholesta-5,7,10-triene-1,3,24-triol hydrate (1:1)-vs-Flavobacterium	-0.18828	0.127049	0.247051
mephenesin-vs-Prevotella	0.144624	0.242938	0.398959
mephenesin-vs-Alistipes	0.527416	4.52E-06	0.000106
mephenesin-vs-Faecalibacterium	0.627903	1.29E-08	1.95E-06
mephenesin-vs-Oscillibacter	0.695467	6.53E-11	5.27E-08
mephenesin-vs-Subdoligranulum	0.535717	2.99E-06	8.12E-05

mephenesin-vs-Bilophila	0.457459	9.94E-05	0.000994
mephenesin-vs-Anaerotruncus	0.615293	3.02E-08	3.48E-06
mephenesin-vs-Butyricicoccus	0.596137	1.02E-07	8.25E-06
mephenesin-vs-Paraprevotella	0.244353	0.046284	0.11341
mephenesin-vs-Collinsella	0.444529	0.000164	0.001403
mephenesin-vs-Parasutterella	0.213904	0.082192	0.179605
mephenesin-vs-Pyramidobacter	0.597534	9.38E-08	8.11E-06
mephenesin-vs-Intestinimonas	0.66805	6.58E-10	2.65E-07
mephenesin-vs-Pseudoflavonifractor	0.623474	1.75E-08	2.35E-06
mephenesin-vs-Holdemania	0.710751	1.61E-11	3.89E-08
mephenesin-vs-Oribacterium	0.605874	5.55E-08	5.37E-06
mephenesin-vs-Bacteroides	-0.12998	0.294476	0.456042
mephenesin-vs-Eubacterium	0.335821	0.005465	0.021743
mephenesin-vs-Veillonella	-0.35322	0.003368	0.014868
mephenesin-vs-Lactobacillus	0.074188	0.550742	0.682852
mephenesin-vs-Agathobacter	0.292162	0.016438	0.051775
mephenesin-vs-Enterococcus	-0.04685	0.706557	0.808879
mephenesin-vs-Blautia	-0.05783	0.64207	0.757914
mephenesin-vs-Streptococcus	-0.16909	0.171353	0.307548
mephenesin-vs-Erysipelatoclostridium	-0.10779	0.385269	0.543104
mephenesin-vs-Lachnospira	0.253452	0.038502	0.100145
mephenesin-vs-Fusobacterium	0.126467	0.307826	0.469799
mephenesin-vs-Bacillus	0.265304	0.030023	0.08197
mephenesin-vs-Dorea	0.26726	0.028787	0.079494
mephenesin-vs-Tyzzerella	0.116849	0.346356	0.507471

mephenesin-vs-Butyrivibrio	0.512012	9.48E-06	0.000185
mephenesin-vs-Mycoplasma	0.241759	0.048725	0.117631
mephenesin-vs-Coprobacillus	-0.00359	0.976988	0.985543
mephenesin-vs-Selenomonas	0.264586	0.030488	0.083145
mephenesin-vs-Anaerostipes	0.171283	0.16578	0.299942
mephenesin-vs-Peptoclostridium	0.321335	0.008015	0.029419
mephenesin-vs-Dysgonomonas	0.00012	0.999233	0.999233
mephenesin-vs-Coprobacter	0.187365	0.128946	0.2491
mephenesin-vs-Capnocytophaga	-0.12607	0.309367	0.470544
mephenesin-vs-Campylobacter	0.066925	0.59051	0.717811
mephenesin-vs-Flavobacterium	0.177668	0.150327	0.278013
Aspirin-vs-Prevotella	0.195786	0.112326	0.226241
Aspirin-vs-Alistipes	0.109107	0.379456	0.538925
Aspirin-vs-Faecalibacterium	0.587637	1.71E-07	1.08E-05
Aspirin-vs-Oscillibacter	0.413122	0.000512	0.003453
Aspirin-vs-Subdoligranulum	0.420784	0.000392	0.002779
Aspirin-vs-Bilophila	0.110464	0.373522	0.533382
Aspirin-vs-Anaerotruncus	0.21542	0.079993	0.176072
Aspirin-vs-Butyricicoccus	0.371658	0.001957	0.009803
Aspirin-vs-Paraprevotella	0.033323	0.788931	0.866067
Aspirin-vs-Collinsella	0.299026	0.013967	0.045343
Aspirin-vs-Parasutterella	0.144984	0.241757	0.39756
Aspirin-vs-Pyramidobacter	0.179025	0.147186	0.273879
Aspirin-vs-Intestinimonas	0.24583	0.044939	0.111496
Aspirin-vs-Pseudoflavonifractor	0.316107	0.009162	0.032689

Aspirin-vs-Holdemania	0.421662	0.00038	0.002722
Aspirin-vs-Oribacterium	0.304174	0.012331	0.041545
Aspirin-vs-Bacteroides	-0.12116	0.328733	0.489056
Aspirin-vs-Eubacterium	-0.00331	0.978777	0.986783
Aspirin-vs-Veillonella	-0.41168	0.000538	0.003564
Aspirin-vs-Lactobacillus	-0.15109	0.222288	0.37362
Aspirin-vs-Agathobacter	0.092266	0.457722	0.607219
Aspirin-vs-Enterococcus	-0.24735	0.043592	0.109388
Aspirin-vs-Blautia	-0.16051	0.194453	0.336227
Aspirin-vs-Streptococcus	-0.12898	0.29823	0.459794
Aspirin-vs-Erysipelatoclostridium	-0.18956	0.124444	0.243749
Aspirin-vs-Lachnospira	0.133291	0.282237	0.441898
Aspirin-vs-Fusobacterium	-0.03771	0.761898	0.848814
Aspirin-vs-Bacillus	0.032165	0.7961	0.8706
Aspirin-vs-Dorea	0.108748	0.381036	0.539969
Aspirin-vs-Tyzzerella	-0.06589	0.596295	0.72194
Aspirin-vs-Butyrivibrio	0.177508	0.1507	0.27849
Aspirin-vs-Mycoplasma	-0.06736	0.58807	0.716825
Aspirin-vs-Coprobacillus	-0.12846	0.300194	0.461352
Aspirin-vs-Selenomonas	0.037074	0.765814	0.851166
Aspirin-vs-Anaerostipes	-0.06529	0.599644	0.724906
Aspirin-vs-Peptoclostridium	0.066047	0.595403	0.721853
Aspirin-vs-Dysgonomonas	-0.21175	0.085399	0.184906
Aspirin-vs-Coprobacter	-0.04027	0.746298	0.837724
Aspirin-vs-Capnocytophaga	-0.10332	0.405393	0.55807

Aspirin-vs-Campylobacter	-0.02722	0.82694	0.889844
Aspirin-vs-Flavobacterium	0.022109	0.85905	0.910092
4-Oxo-4-(3-pyridinyl)butanal-vs-Prevotella	0.137641	0.266684	0.428074
4-Oxo-4-(3-pyridinyl)butanal-vs-Alistipes	0.356972	0.003024	0.013687
4-Oxo-4-(3-pyridinyl)butanal-vs-Faecalibacterium	0.520832	6.23E-06	0.000132
4-Oxo-4-(3-pyridinyl)butanal-vs-Oscillibacter	0.387541	0.001195	0.006645
4-Oxo-4-(3-pyridinyl)butanal-vs-Subdoligranulum	0.30182	0.013058	0.043151
4-Oxo-4-(3-pyridinyl)butanal-vs-Bilophila	0.213704	0.082485	0.17992
4-Oxo-4-(3-pyridinyl)butanal-vs-Anaerotruncus	0.312315	0.010082	0.035534
4-Oxo-4-(3-pyridinyl)butanal-vs-Butyricicoccus	0.392809	0.001009	0.005839
4-Oxo-4-(3-pyridinyl)butanal-vs-Paraprevotella	0.149812	0.226266	0.377735
4-Oxo-4-(3-pyridinyl)butanal-vs-Collinsella	0.256884	0.035864	0.094813
4-Oxo-4-(3-pyridinyl)butanal-vs-Parasutterella	-0.01959	0.874941	0.921813
4-Oxo-4-(3-pyridinyl)butanal-vs-Pyramidobacter	0.156198	0.206864	0.353892
4-Oxo-4-(3-pyridinyl)butanal-vs-Intestinimonas	0.355735	0.003134	0.013976
4-Oxo-4-(3-pyridinyl)butanal-vs-Pseudoflavonifractor	0.337577	0.005211	0.020973
4-Oxo-4-(3-pyridinyl)butanal-vs-Holdemania	0.398795	0.00083	0.005056
4-Oxo-4-(3-pyridinyl)butanal-vs-Oribacterium	0.24986	0.041433	0.105612
4-Oxo-4-(3-pyridinyl)butanal-vs-Bacteroides	-0.11601	0.34985	0.510118
4-Oxo-4-(3-pyridinyl)butanal-vs-Eubacterium	0.015324	0.902042	0.939304
4-Oxo-4-(3-pyridinyl)butanal-vs-Veillonella	-0.22296	0.069741	0.158408
4-Oxo-4-(3-pyridinyl)butanal-vs-Lactobacillus	-0.14514	0.241234	0.396969
4-Oxo-4-(3-pyridinyl)butanal-vs-Agathobacter	0.009019	0.942255	0.96622
4-Oxo-4-(3-pyridinyl)butanal-vs-Enterococcus	-0.17767	0.150327	0.278013
4-Oxo-4-(3-pyridinyl)butanal-vs-Blautia	-0.15105	0.222411	0.37362

4-Oxo-4-(3-pyridinyl)butanal-vs-Streptococcus	-0.13473	0.277035	0.437142
4-Oxo-4-(3-pyridinyl)butanal-vs-Erysipelatoclostridium	-0.22915	0.062144	0.144684
4-Oxo-4-(3-pyridinyl)butanal-vs-Lachnospira	-0.06214	0.617416	0.73791
4-Oxo-4-(3-pyridinyl)butanal-vs-Fusobacterium	-0.07606	0.540685	0.675577
4-Oxo-4-(3-pyridinyl)butanal-vs-Bacillus	-0.01908	0.878227	0.923796
4-Oxo-4-(3-pyridinyl)butanal-vs-Dorea	-0.05747	0.644142	0.758977
4-Oxo-4-(3-pyridinyl)butanal-vs-Tyzzerella	-0.04853	0.69656	0.800084
4-Oxo-4-(3-pyridinyl)butanal-vs-Butyrivibrio	0.135645	0.273745	0.433087
4-Oxo-4-(3-pyridinyl)butanal-vs-Mycoplasma	-0.00635	0.959356	0.975487
4-Oxo-4-(3-pyridinyl)butanal-vs-Coprobacillus	-0.2847	0.01954	0.059159
4-Oxo-4-(3-pyridinyl)butanal-vs-Selenomonas	0.046732	0.707273	0.808933
4-Oxo-4-(3-pyridinyl)butanal-vs-Anaerostipes	-0.03991	0.748486	0.839401
4-Oxo-4-(3-pyridinyl)butanal-vs-Peptoclostridium	-0.0018	0.988493	0.992853
4-Oxo-4-(3-pyridinyl)butanal-vs-Dysgonomonas	-0.06637	0.593622	0.720686
4-Oxo-4-(3-pyridinyl)butanal-vs-Coprobacter	0.302059	0.012982	0.04296
4-Oxo-4-(3-pyridinyl)butanal-vs-Capnocytophaga	-0.15181	0.220071	0.371236
4-Oxo-4-(3-pyridinyl)butanal-vs-Campylobacter	-0.13573	0.273461	0.433087
4-Oxo-4-(3-pyridinyl)butanal-vs-Flavobacterium	0.049286	0.692054	0.796043
7,11,12-Trihydroxy-6,20-epoxyabieta-8(14),9(11),12-trien-20-one-vs-Prevotella	0.283542	0.020064	0.060345
7,11,12-Trihydroxy-6,20-epoxyabieta-8(14),9(11),12-trien-20-one-vs-Alistipes	0.248823	0.042313	0.107291
7,11,12-Trihydroxy-6,20-epoxyabieta-8(14),9(11),12-trien-20-one-vs-Faecalibacterium	0.584005	2.13E-07	1.23E-05

7,11,12-Trihydroxy-6,20-epoxyabieta-8(14),9(11),12-trien-20-one-vs-Oscillibacter	0.4484	0.000142	0.00127
7,11,12-Trihydroxy-6,20-epoxyabieta-8(14),9(11),12-trien-20-one-vs-Subdoligranulum	0.298029	0.014305	0.046078
7,11,12-Trihydroxy-6,20-epoxyabieta-8(14),9(11),12-trien-20-one-vs-Bilophila	0.18533	0.13323	0.255174
7,11,12-Trihydroxy-6,20-epoxyabieta-8(14),9(11),12-trien-20-one-vs-Anaerotruncus	0.345878	0.004145	0.017408
7,11,12-Trihydroxy-6,20-epoxyabieta-8(14),9(11),12-trien-20-one-vs-Butyricicoccus	0.482321	3.58E-05	0.000481
7,11,12-Trihydroxy-6,20-epoxyabieta-8(14),9(11),12-trien-20-one-vs-Paraprevotella	0.119044	0.337309	0.498138
7,11,12-Trihydroxy-6,20-epoxyabieta-8(14),9(11),12-trien-20-one-vs-Collinsella	0.342725	0.004525	0.018646
7,11,12-Trihydroxy-6,20-epoxyabieta-8(14),9(11),12-trien-20-one-vs-Parasutterella	0.177947	0.149676	0.277578
7,11,12-Trihydroxy-6,20-epoxyabieta-8(14),9(11),12-trien-20-one-vs-Pyramidobacter	0.299545	0.013794	0.044954
7,11,12-Trihydroxy-6,20-epoxyabieta-8(14),9(11),12-trien-20-one-vs-Intestinimonas	0.40518	0.000671	0.004215
7,11,12-Trihydroxy-6,20-epoxyabieta-8(14),9(11),12-trien-20-one-vs-Pseudoflavonifractor	0.307247	0.011436	0.039017
7,11,12-Trihydroxy-6,20-epoxyabieta-8(14),9(11),12-trien-20-one-vs-Holdemania	0.460532	0.000088	0.000915

7,11,12-Trihydroxy-6,20-epoxyabieta-8(14),9(11),12-trien-20-one-vs-Oribacterium	0.325245	0.00724	0.027352
7,11,12-Trihydroxy-6,20-epoxyabieta-8(14),9(11),12-trien-20-one-vs-Bacteroides	-0.14219	0.251044	0.409492
7,11,12-Trihydroxy-6,20-epoxyabieta-8(14),9(11),12-trien-20-one-vs-Eubacterium	0.136882	0.269353	0.429792
7,11,12-Trihydroxy-6,20-epoxyabieta-8(14),9(11),12-trien-20-one-vs-Veillonella	-0.278	0.022741	0.066437
7,11,12-Trihydroxy-6,20-epoxyabieta-8(14),9(11),12-trien-20-one-vs-Lactobacillus	-0.1362	0.271756	0.432201
7,11,12-Trihydroxy-6,20-epoxyabieta-8(14),9(11),12-trien-20-one-vs-Agathobacter	0.067044	0.589844	0.717362
7,11,12-Trihydroxy-6,20-epoxyabieta-8(14),9(11),12-trien-20-one-vs-Enterococcus	-0.1542	0.212796	0.36123
7,11,12-Trihydroxy-6,20-epoxyabieta-8(14),9(11),12-trien-20-one-vs-Blautia	-0.2661	0.029513	0.080922
7,11,12-Trihydroxy-6,20-epoxyabieta-8(14),9(11),12-trien-20-one-vs-Streptococcus	-0.12567	0.310914	0.47118
7,11,12-Trihydroxy-6,20-epoxyabieta-8(14),9(11),12-trien-20-one-vs-Erysipelatoclostridium	-0.33047	0.006308	0.024297
7,11,12-Trihydroxy-6,20-epoxyabieta-8(14),9(11),12-trien-20-one-vs-Lachnospira	0.101006	0.416051	0.568535
7,11,12-Trihydroxy-6,20-epoxyabieta-8(14),9(11),12-trien-20-one-vs-Fusobacterium	-0.11214	0.366269	0.526378

7,11,12-Trihydroxy-6,20-epoxyabieta-8(14),9(11),12-trien-20-one-vs-Bacillus	0.034959	0.778824	0.858574
7,11,12-Trihydroxy-6,20-epoxyabieta-8(14),9(11),12-trien-20-one-vs-Dorea	-0.02933	0.813724	0.879928
7,11,12-Trihydroxy-6,20-epoxyabieta-8(14),9(11),12-trien-20-one-vs-Tyzzerella	-0.13221	0.286181	0.446916
7,11,12-Trihydroxy-6,20-epoxyabieta-8(14),9(11),12-trien-20-one-vs-Butyrivibrio	0.200694	0.103428	0.213841
7,11,12-Trihydroxy-6,20-epoxyabieta-8(14),9(11),12-trien-20-one-vs-Mycoplasma	-0.05914	0.634494	0.752742
7,11,12-Trihydroxy-6,20-epoxyabieta-8(14),9(11),12-trien-20-one-vs-Coprobacillus	-0.36024	0.002749	0.012682
7,11,12-Trihydroxy-6,20-epoxyabieta-8(14),9(11),12-trien-20-one-vs-Selenomonas	0.135326	0.274887	0.434325
7,11,12-Trihydroxy-6,20-epoxyabieta-8(14),9(11),12-trien-20-one-vs-Anaerostipes	-0.08656	0.48612	0.629847
7,11,12-Trihydroxy-6,20-epoxyabieta-8(14),9(11),12-trien-20-one-vs-Peptoclostridium	-0.01692	0.891896	0.933981
7,11,12-Trihydroxy-6,20-epoxyabieta-8(14),9(11),12-trien-20-one-vs-Dysgonomonas	-0.11641	0.348183	0.508608
7,11,12-Trihydroxy-6,20-epoxyabieta-8(14),9(11),12-trien-20-one-vs-Coprobacter	0.260196	0.033462	0.08964
7,11,12-Trihydroxy-6,20-epoxyabieta-8(14),9(11),12-trien-20-one-vs-Capnocytophaga	-0.06138	0.621724	0.741959

7,11,12-Trihydroxy-6,20-epoxyabieta-8(14),9(11),12-trien-20-one-vs-Campylobacter	-0.01632	0.895699	0.937012
7,11,12-Trihydroxy-6,20-epoxyabieta-8(14),9(11),12-trien-20-one-vs-Flavobacterium	0.16366	0.185719	0.326019
22-Oxodocosanoic acid-vs-Prevotella	0.250339	0.041032	0.1047
22-Oxodocosanoic acid-vs-Alistipes	0.364355	0.002436	0.011487
22-Oxodocosanoic acid-vs-Faecalibacterium	0.420704	0.000393	0.002779
22-Oxodocosanoic acid-vs-Oscillibacter	0.447442	0.000147	0.001297
22-Oxodocosanoic acid-vs-Subdoligranulum	0.211868	0.085218	0.184715
22-Oxodocosanoic acid-vs-Bilophila	0.27137	0.026329	0.073972
22-Oxodocosanoic acid-vs-Anaerotruncus	0.371378	0.001974	0.009837
22-Oxodocosanoic acid-vs-Butyricicoccus	0.443451	0.000171	0.001426
22-Oxodocosanoic acid-vs-Paraprevotella	0.190039	0.123478	0.242919
22-Oxodocosanoic acid-vs-Collinsella	0.446923	0.00015	0.001313
22-Oxodocosanoic acid-vs-Parasutterella	0.28877	0.017791	0.055105
22-Oxodocosanoic acid-vs-Pyramidobacter	0.297151	0.014608	0.046805
22-Oxodocosanoic acid-vs-Intestinimonas	0.375928	0.001718	0.008732
22-Oxodocosanoic acid-vs-Pseudoflavonifractor	0.338096	0.005138	0.020783
22-Oxodocosanoic acid-vs-Holdemania	0.245949	0.044832	0.111432
22-Oxodocosanoic acid-vs-Oribacterium	0.13788	0.265845	0.427579
22-Oxodocosanoic acid-vs-Bacteroides	-0.2756	0.023987	0.069075
22-Oxodocosanoic acid-vs-Eubacterium	-0.09039	0.466954	0.613485
22-Oxodocosanoic acid-vs-Veillonella	-0.53819	2.64E-06	0.000075
22-Oxodocosanoic acid-vs-Lactobacillus	-0.28693	0.018563	0.057056
22-Oxodocosanoic acid-vs-Agathobacter	-0.19104	0.121484	0.240246

22-Oxodocosanoic acid-vs-Enterococcus	-0.33566	0.005489	0.021766
22-Oxodocosanoic acid-vs-Blautia	-0.27532	0.024136	0.06934
22-Oxodocosanoic acid-vs-Streptococcus	-0.42904	0.000292	0.002204
22-Oxodocosanoic acid-vs-Erysipelatoclostridium	-0.42282	0.000365	0.002655
22-Oxodocosanoic acid-vs-Lachnospira	-0.21127	0.086125	0.186014
22-Oxodocosanoic acid-vs-Fusobacterium	-0.29264	0.016254	0.05133
22-Oxodocosanoic acid-vs-Bacillus	-0.25393	0.038124	0.0997
22-Oxodocosanoic acid-vs-Dorea	-0.25198	0.039685	0.102343
22-Oxodocosanoic acid-vs-Tyzzerella	-0.13169	0.288093	0.449031
22-Oxodocosanoic acid-vs-Butyrivibrio	-0.05942	0.632892	0.75185
22-Oxodocosanoic acid-vs-Mycoplasma	-0.07754	0.532832	0.668181
22-Oxodocosanoic acid-vs-Coprobacillus	-0.52817	4.36E-06	0.000104
22-Oxodocosanoic acid-vs-Selenomonas	-0.03859	0.756525	0.844111
22-Oxodocosanoic acid-vs-Anaerostipes	-0.19994	0.104766	0.216052
22-Oxodocosanoic acid-vs-Peptoclostridium	-0.06178	0.619455	0.739981
22-Oxodocosanoic acid-vs-Dysgonomonas	-0.30266	0.012795	0.042691
22-Oxodocosanoic acid-vs-Coprobacter	0.001078	0.993095	0.995152
22-Oxodocosanoic acid-vs-Capnocytophaga	-0.19646	0.111062	0.224219
22-Oxodocosanoic acid-vs-Campylobacter	-0.29699	0.014664	0.046921
22-Oxodocosanoic acid-vs-Flavobacterium	-0.12555	0.311379	0.471355
Orotic acid-vs-Prevotella	0.114734	0.355215	0.515147
Orotic acid-vs-Alistipes	0.255727	0.036736	0.096486
Orotic acid-vs-Faecalibacterium	0.402107	0.000743	0.004635
Orotic acid-vs-Oscillibacter	0.439979	0.000195	0.001576
Orotic acid-vs-Subdoligranulum	0.27141	0.026306	0.073972

Orotic acid-vs-Bilophila	0.054075	0.663847	0.772413
Orotic acid-vs-Anaerotruncus	0.356533	0.003062	0.013769
Orotic acid-vs-Butyricicoccus	0.58201	2.39E-07	1.26E-05
Orotic acid-vs-Paraprevotella	0.099689	0.422187	0.574392
Orotic acid-vs-Collinsella	0.48276	3.51E-05	0.000481
Orotic acid-vs-Parasutterella	0.121758	0.326331	0.487157
Orotic acid-vs-Pyramidobacter	0.289528	0.017481	0.054283
Orotic acid-vs-Intestinimonas	0.413441	0.000506	0.003453
Orotic acid-vs-Pseudoflavonifractor	0.485314	3.15E-05	0.000443
Orotic acid-vs-Holdemania	0.380717	0.001482	0.00786
Orotic acid-vs-Oribacterium	0.185809	0.132213	0.253626
Orotic acid-vs-Bacteroides	-0.27803	0.02272	0.066437
Orotic acid-vs-Eubacterium	0.042302	0.733938	0.82785
Orotic acid-vs-Veillonella	-0.34492	0.004257	0.017817
Orotic acid-vs-Lactobacillus	0.034041	0.784489	0.862974
Orotic acid-vs-Agathobacter	0.05591	0.653154	0.764013
Orotic acid-vs-Enterococcus	-0.18665	0.130446	0.250834
Orotic acid-vs-Blautia	-0.11605	0.349683	0.510118
Orotic acid-vs-Streptococcus	-0.03628	0.770716	0.853136
Orotic acid-vs-Erysipelatoclostridium	-0.25557	0.036857	0.096701
Orotic acid-vs-Lachnospira	-0.08875	0.475089	0.619537
Orotic acid-vs-Fusobacterium	-0.24874	0.042381	0.107351
Orotic acid-vs-Bacillus	0.037673	0.762143	0.848814
Orotic acid-vs-Dorea	0.059462	0.632663	0.75185
Orotic acid-vs-Tyzzerella	-0.10292	0.407219	0.559378

Orotic acid-vs-Butyrivibrio	0.042023	0.735631	0.828441
Orotic acid-vs-Mycoplasma	-0.0443	0.721886	0.820217
Orotic acid-vs-Coprobacillus	-0.3721	0.001931	0.009713
Orotic acid-vs-Selenomonas	0.039588	0.750432	0.840804
Orotic acid-vs-Anaerostipes	0.036795	0.767528	0.851166
Orotic acid-vs-Peptoclostridium	0.009259	0.940724	0.96506
Orotic acid-vs-Dysgonomonas	-0.23613	0.054386	0.12936
Orotic acid-vs-Coprobacter	0.100168	0.41995	0.572637
Orotic acid-vs-Capnocytophaga	-0.17978	0.145452	0.271489
Orotic acid-vs-Campylobacter	-0.17176	0.164582	0.298893
Orotic acid-vs-Flavobacterium	0.049405	0.691343	0.795604
Isoprenaline-vs-Prevotella	0.125988	0.309676	0.470544
Isoprenaline-vs-Alistipes	0.245909	0.044868	0.111432
Isoprenaline-vs-Faecalibacterium	0.506186	1.24E-05	0.000223
Isoprenaline-vs-Oscillibacter	0.420345	0.000398	0.002798
Isoprenaline-vs-Subdoligranulum	0.320896	0.008106	0.029664
Isoprenaline-vs-Bilophila	0.085482	0.491585	0.634548
Isoprenaline-vs-Anaerotruncus	0.383111	0.001375	0.007382
Isoprenaline-vs-Butyricicoccus	0.479049	4.11E-05	0.000535
Isoprenaline-vs-Paraprevotella	0.131295	0.289569	0.450462
Isoprenaline-vs-Collinsella	0.300623	0.013441	0.044099
Isoprenaline-vs-Parasutterella	0.106273	0.392029	0.54975
Isoprenaline-vs-Pyramidobacter	0.300702	0.013415	0.044092
Isoprenaline-vs-Intestinimonas	0.413241	0.00051	0.003453
Isoprenaline-vs-Pseudoflavonifractor	0.450794	0.000129	0.001186

Isoprenaline-vs-Holdemania	0.441496	0.000184	0.001505
Isoprenaline-vs-Oribacterium	0.302219	0.012932	0.042853
Isoprenaline-vs-Bacteroides	-0.26064	0.033154	0.088988
Isoprenaline-vs-Eubacterium	0.058544	0.637933	0.75571
Isoprenaline-vs-Veillonella	-0.25345	0.038502	0.100145
Isoprenaline-vs-Lactobacillus	-0.02315	0.852507	0.905672
Isoprenaline-vs-Agathobacter	0.036236	0.770961	0.853136
Isoprenaline-vs-Enterococcus	-0.10488	0.398317	0.554071
Isoprenaline-vs-Blautia	-0.14195	0.251851	0.410255
Isoprenaline-vs-Streptococcus	-0.02179	0.861065	0.911561
Isoprenaline-vs-Erysipelatoclostridium	-0.20552	0.09522	0.201872
Isoprenaline-vs-Lachnospira	-0.12866	0.299437	0.460775
Isoprenaline-vs-Fusobacterium	-0.11178	0.367816	0.52766
Isoprenaline-vs-Bacillus	0.114814	0.354878	0.514967
Isoprenaline-vs-Dorea	0.068242	0.583204	0.713953
Isoprenaline-vs-Tyzzerella	-0.05228	0.674373	0.779784
Isoprenaline-vs-Butyrivibrio	0.1291	0.297777	0.45939
Isoprenaline-vs-Mycoplasma	-0.02239	0.857288	0.909153
Isoprenaline-vs-Coprobacillus	-0.29284	0.016178	0.051157
Isoprenaline-vs-Selenomonas	0.097893	0.430636	0.58359
Isoprenaline-vs-Anaerostipes	0.043659	0.725735	0.823348
Isoprenaline-vs-Peptoclostridium	0.093623	0.451107	0.602555
Isoprenaline-vs-Dysgonomonas	-0.12363	0.318877	0.479108
Isoprenaline-vs-Coprobacter	0.182217	0.139989	0.263939
Isoprenaline-vs-Capnocytophaga	-0.1042	0.401393	0.555157

Isoprenaline-vs-Campylobacter	-0.16422	0.184201	0.323825
Isoprenaline-vs-Flavobacterium	0.134169	0.279051	0.438327
(3alpha,5beta,7alpha,12alpha,25R)-3,7,12-Trihydroxycholestan-26-oic acid-vs-Prevotella	-0.07814	0.529664	0.664553
(3alpha,5beta,7alpha,12alpha,25R)-3,7,12-Trihydroxycholestan-26-oic acid-vs-Alistipes	0.163181	0.187027	0.326892
(3alpha,5beta,7alpha,12alpha,25R)-3,7,12-Trihydroxycholestan-26-oic acid-vs-Faecalibacterium	0.557347	9.64E-07	3.64E-05
(3alpha,5beta,7alpha,12alpha,25R)-3,7,12-Trihydroxycholestan-26-oic acid-vs-Oscillibacter	0.40965	0.000577	0.003759
(3alpha,5beta,7alpha,12alpha,25R)-3,7,12-Trihydroxycholestan-26-oic acid-vs-Subdoligranulum	0.261394	0.032628	0.08799
(3alpha,5beta,7alpha,12alpha,25R)-3,7,12-Trihydroxycholestan-26-oic acid-vs-Bilophila	0.201532	0.101966	0.21245
(3alpha,5beta,7alpha,12alpha,25R)-3,7,12-Trihydroxycholestan-26-oic acid-vs-Anaerotruncus	0.422659	0.000367	0.002655
(3alpha,5beta,7alpha,12alpha,25R)-3,7,12-Trihydroxycholestan-26-oic acid-vs-Butyricicoccus	0.524543	5.21E-06	0.000119
(3alpha,5beta,7alpha,12alpha,25R)-3,7,12-Trihydroxycholestan-26-oic acid-vs-Paraprevotella	-0.01536	0.901788	0.939304
(3alpha,5beta,7alpha,12alpha,25R)-3,7,12-Trihydroxycholestan-26-oic acid-vs-Collinsella	0.095219	0.443392	0.595208
(3alpha,5beta,7alpha,12alpha,25R)-3,7,12-Trihydroxycholestan-26-oic acid-vs-Parasutterella	0.139037	0.261814	0.422501

(3alpha,5beta,7alpha,12alpha,25R)-3,7,12-Trihydroxycholestan-26-oic acid-vs-Pyramidobacter	0.22428	0.068064	0.155328
(3alpha,5beta,7alpha,12alpha,25R)-3,7,12-Trihydroxycholestan-26-oic acid-vs-Intestinimonas	0.468952	6.26E-05	0.000742
(3alpha,5beta,7alpha,12alpha,25R)-3,7,12-Trihydroxycholestan-26-oic acid-vs-Pseudoflavonifractor	0.466119	7.03E-05	0.000798
(3alpha,5beta,7alpha,12alpha,25R)-3,7,12-Trihydroxycholestan-26-oic acid-vs-Holdemania	0.397238	0.000873	0.005216
(3alpha,5beta,7alpha,12alpha,25R)-3,7,12-Trihydroxycholestan-26-oic acid-vs-Oribacterium	0.440698	0.00019	0.001545
(3alpha,5beta,7alpha,12alpha,25R)-3,7,12-Trihydroxycholestan-26-oic acid-vs-Bacteroides	0.047969	0.699886	0.802547
(3alpha,5beta,7alpha,12alpha,25R)-3,7,12-Trihydroxycholestan-26-oic acid-vs-Eubacterium	0.337218	0.005262	0.021109
(3alpha,5beta,7alpha,12alpha,25R)-3,7,12-Trihydroxycholestan-26-oic acid-vs-Veillonella	-0.07475	0.547737	0.680172
(3alpha,5beta,7alpha,12alpha,25R)-3,7,12-Trihydroxycholestan-26-oic acid-vs-Lactobacillus	0.065807	0.596741	0.722119
(3alpha,5beta,7alpha,12alpha,25R)-3,7,12-Trihydroxycholestan-26-oic acid-vs-Agathobacter	0.12495	0.31371	0.47429
(3alpha,5beta,7alpha,12alpha,25R)-3,7,12-Trihydroxycholestan-26-oic acid-vs-Enterococcus	-0.09929	0.424056	0.575895
(3alpha,5beta,7alpha,12alpha,25R)-3,7,12-Trihydroxycholestan-26-oic acid-vs-Blautia	0.076662	0.537495	0.672633

(3alpha,5beta,7alpha,12alpha,25R)-3,7,12-Trihydroxycholestan-26-oic acid-vs-Streptococcus	-0.04438	0.721405	0.820056
(3alpha,5beta,7alpha,12alpha,25R)-3,7,12-Trihydroxycholestan-26-oic acid-vs-Erysipelatoclostridium	-0.0433	0.727904	0.823348
(3alpha,5beta,7alpha,12alpha,25R)-3,7,12-Trihydroxycholestan-26-oic acid-vs-Lachnospira	0.08181	0.510443	0.649191
(3alpha,5beta,7alpha,12alpha,25R)-3,7,12-Trihydroxycholestan-26-oic acid-vs-Fusobacterium	0.132133	0.286475	0.447085
(3alpha,5beta,7alpha,12alpha,25R)-3,7,12-Trihydroxycholestan-26-oic acid-vs-Bacillus	0.10336	0.405211	0.55807
(3alpha,5beta,7alpha,12alpha,25R)-3,7,12-Trihydroxycholestan-26-oic acid-vs-Dorea	0.135326	0.274887	0.434325
(3alpha,5beta,7alpha,12alpha,25R)-3,7,12-Trihydroxycholestan-26-oic acid-vs-Tyzzerella	0.264307	0.03067	0.083549
(3alpha,5beta,7alpha,12alpha,25R)-3,7,12-Trihydroxycholestan-26-oic acid-vs-Butyrivibrio	0.278913	0.022278	0.0658
(3alpha,5beta,7alpha,12alpha,25R)-3,7,12-Trihydroxycholestan-26-oic acid-vs-Mycoplasma	-0.00327	0.979033	0.986783
(3alpha,5beta,7alpha,12alpha,25R)-3,7,12-Trihydroxycholestan-26-oic acid-vs-Coprobacillus	-0.11425	0.35724	0.517399
(3alpha,5beta,7alpha,12alpha,25R)-3,7,12-Trihydroxycholestan-26-oic acid-vs-Selenomonas	0.076662	0.537495	0.672633
(3alpha,5beta,7alpha,12alpha,25R)-3,7,12-Trihydroxycholestan-26-oic acid-vs-Anaerostipes	0.109905	0.375958	0.535845

(3alpha,5beta,7alpha,12alpha,25R)-3,7,12-Trihydroxycholestan-26-oic acid-vs-Peptoclostridium	0.248623	0.042484	0.107473
(3alpha,5beta,7alpha,12alpha,25R)-3,7,12-Trihydroxycholestan-26-oic acid-vs-Dysgonomonas	-0.10276	0.407951	0.559747
(3alpha,5beta,7alpha,12alpha,25R)-3,7,12-Trihydroxycholestan-26-oic acid-vs-Coprobacter	-0.0261	0.833942	0.894196
(3alpha,5beta,7alpha,12alpha,25R)-3,7,12-Trihydroxycholestan-26-oic acid-vs-Capnocytophaga	-0.27281	0.025513	0.072182
(3alpha,5beta,7alpha,12alpha,25R)-3,7,12-Trihydroxycholestan-26-oic acid-vs-Campylobacter	0.097294	0.433473	0.586378
(3alpha,5beta,7alpha,12alpha,25R)-3,7,12-Trihydroxycholestan-26-oic acid-vs-Flavobacterium	-0.17926	0.146637	0.273278
gibberellin A1-vs-Prevotella	0.240243	0.050201	0.120712
gibberellin A1-vs-Alistipes	0.243116	0.047435	0.115067
gibberellin A1-vs-Faecalibacterium	0.302578	0.01282	0.042715
gibberellin A1-vs-Oscillibacter	0.433275	0.00025	0.001937
gibberellin A1-vs-Subdoligranulum	0.298787	0.014048	0.04537
gibberellin A1-vs-Bilophila	0.215939	0.079252	0.174758
gibberellin A1-vs-Anaerotruncus	0.259837	0.033716	0.089921
gibberellin A1-vs-Butyricicoccus	0.372695	0.001897	0.00959
gibberellin A1-vs-Paraprevotella	0.049645	0.689923	0.794892
gibberellin A1-vs-Collinsella	0.465959	7.07E-05	0.0008
gibberellin A1-vs-Parasutterella	0.305611	0.011905	0.040448
gibberellin A1-vs-Pyramidobacter	0.287134	0.018478	0.056867
gibberellin A1-vs-Intestinimonas	0.281427	0.021051	0.062789

gibberellin A1-vs-Pseudoflavonifractor	0.278115	0.02268	0.066419
gibberellin A1-vs-Holdemania	0.271131	0.026467	0.07406
gibberellin A1-vs-Oribacterium	0.104039	0.402118	0.555208
gibberellin A1-vs-Bacteroides	-0.17527	0.155991	0.285866
gibberellin A1-vs-Eubacterium	-0.09482	0.445314	0.597125
gibberellin A1-vs-Veillonella	-0.54434	1.92E-06	5.84E-05
gibberellin A1-vs-Lactobacillus	-0.29815	0.014264	0.046007
gibberellin A1-vs-Agathobacter	0.002554	0.983634	0.98936
gibberellin A1-vs-Enterococcus	-0.56748	5.51E-07	2.38E-05
gibberellin A1-vs-Blautia	-0.26766	0.02854	0.079061
gibberellin A1-vs-Streptococcus	-0.50706	1.19E-05	0.000217
gibberellin A1-vs-Erysipelatoclostridium	-0.48244	3.56E-05	0.000481
gibberellin A1-vs-Lachnospira	0.054793	0.659655	0.769286
gibberellin A1-vs-Fusobacterium	-0.34903	0.003794	0.01633
gibberellin A1-vs-Bacillus	-0.2857	0.019099	0.058259
gibberellin A1-vs-Dorea	-0.08967	0.470516	0.616231
gibberellin A1-vs-Tyzzerella	-0.21327	0.083132	0.181168
gibberellin A1-vs-Butyrivibrio	-0.04789	0.700362	0.802547
gibberellin A1-vs-Mycoplasma	-0.09973	0.422	0.574392
gibberellin A1-vs-Coprobacillus	-0.26606	0.029539	0.080922
gibberellin A1-vs-Selenomonas	-0.31591	0.009209	0.032759
gibberellin A1-vs-Anaerostipes	-0.27265	0.025602	0.07235
gibberellin A1-vs-Peptoclostridium	-0.10891	0.380333	0.539289
gibberellin A1-vs-Dysgonomonas	-0.29555	0.015174	0.04836
gibberellin A1-vs-Coprobacter	-0.1973	0.109516	0.222545

gibberellin A1-vs-Capnocytophaga	-0.25229	0.039427	0.102003
gibberellin A1-vs-Campylobacter	-0.18046	0.143914	0.269033
gibberellin A1-vs-Flavobacterium	-0.27444	0.024609	0.070449
Tyramine-vs-Prevotella	0.066366	0.593622	0.720686
Tyramine-vs-Alistipes	0.593343	1.21E-07	9.1E-06
Tyramine-vs-Faecalibacterium	0.665496	8.05E-10	2.76E-07
Tyramine-vs-Oscillibacter	0.677428	3.07E-10	1.48E-07
Tyramine-vs-Subdoligranulum	0.385067	0.001293	0.00709
Tyramine-vs-Bilophila	0.487629	2.85E-05	0.00041
Tyramine-vs-Anaerotruncus	0.600327	7.88E-08	7.06E-06
Tyramine-vs-Butyricicoccus	0.589911	1.5E-07	0.00001
Tyramine-vs-Paraprevotella	0.316466	0.009079	0.032488
Tyramine-vs-Collinsella	0.429962	0.000282	0.002139
Tyramine-vs-Parasutterella	0.259079	0.034257	0.090963
Tyramine-vs-Pyramidobacter	0.468513	6.38E-05	0.000749
Tyramine-vs-Intestinimonas	0.580294	2.65E-07	1.31E-05
Tyramine-vs-Pseudoflavonifractor	0.533841	3.29E-06	8.64E-05
Tyramine-vs-Holdemania	0.580573	2.61E-07	1.31E-05
Tyramine-vs-Oribacterium	0.500399	1.62E-05	0.00027
Tyramine-vs-Bacteroides	-0.27843	0.022518	0.066026
Tyramine-vs-Eubacterium	0.206521	0.093589	0.199389
Tyramine-vs-Veillonella	-0.42825	0.0003	0.002258
Tyramine-vs-Lactobacillus	-0.10504	0.397596	0.553704
Tyramine-vs-Agathobacter	0.221327	0.071871	0.161726
Tyramine-vs-Enterococcus	-0.30513	0.012046	0.040868

Tyramine-vs-Blautia	-0.15017	0.225142	0.376378
Tyramine-vs-Streptococcus	-0.32046	0.008198	0.029942
Tyramine-vs-Erysipelatoclostridium	-0.33442	0.005675	0.022286
Tyramine-vs-Lachnospira	0.140075	0.258236	0.418121
Tyramine-vs-Fusobacterium	-0.07327	0.555695	0.687584
Tyramine-vs-Bacillus	0.007223	0.953738	0.973046
Tyramine-vs-Dorea	0.121797	0.326171	0.487157
Tyramine-vs-Tyzzerella	0.103879	0.402845	0.555894
Tyramine-vs-Butyrivibrio	0.300184	0.013584	0.044405
Tyramine-vs-Mycoplasma	0.05192	0.676486	0.78148
Tyramine-vs-Coprobacillus	-0.21215	0.084798	0.183969
Tyramine-vs-Selenomonas	0.080932	0.515007	0.652174
Tyramine-vs-Anaerostipes	0.035677	0.774399	0.855255
Tyramine-vs-Peptoclostridium	0.130378	0.292983	0.454021
Tyramine-vs-Dysgonomonas	-0.29208	0.016469	0.051805
Tyramine-vs-Coprobacter	0.093423	0.452076	0.603184
Tyramine-vs-Capnocytophaga	-0.15991	0.196144	0.338138
Tyramine-vs-Campylobacter	0.079256	0.523777	0.65956
Tyramine-vs-Flavobacterium	-0.06369	0.608614	0.730277
4-Nitroaniline-vs-Prevotella	0.019634	0.874689	0.921813
4-Nitroaniline-vs-Alistipes	0.300742	0.013402	0.044092
4-Nitroaniline-vs-Faecalibacterium	0.353101	0.00338	0.014882
4-Nitroaniline-vs-Oscillibacter	0.480685	3.84E-05	0.000505
4-Nitroaniline-vs-Subdoligranulum	0.303895	0.012415	0.041655
4-Nitroaniline-vs-Bilophila	0.398156	0.000847	0.005118

4-Nitroaniline-vs-Anaerotruncus	0.432157	0.00026	0.001999
4-Nitroaniline-vs-Butyricicoccus	0.497366	1.85E-05	0.000303
4-Nitroaniline-vs-Paraprevotella	0.082409	0.507343	0.647291
4-Nitroaniline-vs-Collinsella	0.285458	0.019204	0.058433
4-Nitroaniline-vs-Parasutterella	0.247665	0.043313	0.108888
4-Nitroaniline-vs-Pyramidobacter	0.389177	0.001134	0.006365
4-Nitroaniline-vs-Intestinimonas	0.517639	7.26E-06	0.00015
4-Nitroaniline-vs-Pseudoflavonifractor	0.548128	1.58E-06	5.08E-05
4-Nitroaniline-vs-Holdemania	0.44836	0.000142	0.00127
4-Nitroaniline-vs-Oribacterium	0.24563	0.045119	0.111549
4-Nitroaniline-vs-Bacteroides	-0.21438	0.081492	0.178849
4-Nitroaniline-vs-Eubacterium	-0.12655	0.307518	0.469625
4-Nitroaniline-vs-Veillonella	-0.42242	0.00037	0.00267
4-Nitroaniline-vs-Lactobacillus	-0.22236	0.070514	0.159266
4-Nitroaniline-vs-Agathobacter	-0.20608	0.094304	0.200281
4-Nitroaniline-vs-Enterococcus	-0.22755	0.064038	0.147953
4-Nitroaniline-vs-Blautia	0.053875	0.665013	0.773398
4-Nitroaniline-vs-Streptococcus	-0.36719	0.002239	0.01079
4-Nitroaniline-vs-Erysipelatoclostridium	-0.17902	0.147186	0.273879
4-Nitroaniline-vs-Lachnospira	-0.275	0.024307	0.069749
4-Nitroaniline-vs-Fusobacterium	-0.21291	0.083665	0.181837
4-Nitroaniline-vs-Bacillus	-0.04957	0.690396	0.794892
4-Nitroaniline-vs-Dorea	-0.13425	0.278762	0.438158
4-Nitroaniline-vs-Tyzzerella	0.010176	0.93486	0.961491
4-Nitroaniline-vs-Butyrivibrio	-0.02015	0.871406	0.918888

4-Nitroaniline-vs-Mycoplasma	-0.14702	0.235141	0.389327
4-Nitroaniline-vs-Coprobacillus	-0.34727	0.003986	0.016887
4-Nitroaniline-vs-Selenomonas	-0.10843	0.382444	0.54133
4-Nitroaniline-vs-Anaerostipes	-0.18182	0.140874	0.265194
4-Nitroaniline-vs-Peptoclostridium	0.095937	0.439944	0.592883
4-Nitroaniline-vs-Dysgonomonas	-0.16905	0.171456	0.307548
4-Nitroaniline-vs-Coprobacter	-0.0947	0.445891	0.597495
4-Nitroaniline-vs-Capnocytophaga	-0.16498	0.182156	0.322573
4-Nitroaniline-vs-Campylobacter	-0.06589	0.596295	0.72194
4-Nitroaniline-vs-Flavobacterium	-0.10783	0.385093	0.543104
Suberic acid-vs-Prevotella	0.077021	0.535585	0.671285
Suberic acid-vs-Alistipes	0.407295	0.000625	0.003965
Suberic acid-vs-Faecalibacterium	0.634528	8.13E-09	1.48E-06
Suberic acid-vs-Oscillibacter	0.442693	0.000176	0.001447
Suberic acid-vs-Subdoligranulum	0.406417	0.000643	0.004074
Suberic acid-vs-Bilophila	0.296592	0.014804	0.047307
Suberic acid-vs-Anaerotruncus	0.45279	0.000119	0.001124
Suberic acid-vs-Butyricicoccus	0.481403	3.72E-05	0.000495
Suberic acid-vs-Paraprevotella	0.189361	0.124848	0.244343
Suberic acid-vs-Collinsella	0.31479	0.009473	0.033649
Suberic acid-vs-Parasutterella	0.157594	0.202782	0.347893
Suberic acid-vs-Pyramidobacter	0.376846	0.00167	0.008597
Suberic acid-vs-Intestinimonas	0.474898	0.000049	0.00062
Suberic acid-vs-Pseudoflavonifractor	0.48292	3.49E-05	0.000481
Suberic acid-vs-Holdemania	0.423817	0.000352	0.002586

Suberic acid-vs-Oribacterium	0.347594	0.003951	0.016824
Suberic acid-vs-Bacteroides	-0.09239	0.457136	0.607219
Suberic acid-vs-Eubacterium	0.218453	0.075736	0.168819
Suberic acid-vs-Veillonella	-0.30884	0.010993	0.037989
Suberic acid-vs-Lactobacillus	-0.00878	0.943785	0.966787
Suberic acid-vs-Agathobacter	0.134568	0.27761	0.437142
Suberic acid-vs-Enterococcus	-0.24431	0.046321	0.11341
Suberic acid-vs-Blautia	-0.16338	0.186481	0.326646
Suberic acid-vs-Streptococcus	-0.21434	0.081551	0.178849
Suberic acid-vs-Erysipelatoclostridium	-0.31104	0.010409	0.036545
Suberic acid-vs-Lachnospira	0.081611	0.511479	0.650166
Suberic acid-vs-Fusobacterium	-0.10132	0.414572	0.567222
Suberic acid-vs-Bacillus	-0.04314	0.728868	0.823509
Suberic acid-vs-Dorea	0.067084	0.589622	0.717362
Suberic acid-vs-Tyzzerella	0.011014	0.929509	0.958957
Suberic acid-vs-Butyrivibrio	0.249062	0.042109	0.106996
Suberic acid-vs-Mycoplasma	-0.03185	0.798081	0.871584
Suberic acid-vs-Coprobacillus	-0.22624	0.065634	0.150922
Suberic acid-vs-Selenomonas	0.086878	0.484507	0.629105
Suberic acid-vs-Anaerostipes	0.085043	0.49382	0.636076
Suberic acid-vs-Peptoclostridium	0.022189	0.858546	0.910089
Suberic acid-vs-Dysgonomonas	-0.07938	0.523148	0.659112
Suberic acid-vs-Coprobacter	0.078817	0.526086	0.661092
Suberic acid-vs-Capnocytophaga	-0.11657	0.347518	0.507943
Suberic acid-vs-Campylobacter	0.010735	0.931292	0.959453

Suberic acid-vs-Flavobacterium	-0.0279	0.822696	0.886854
Vigabatrin-vs-Prevotella	0.056349	0.650607	0.762879
Vigabatrin-vs-Alistipes	0.518118	7.1E-06	0.000149
Vigabatrin-vs-Faecalibacterium	0.410767	0.000555	0.003638
Vigabatrin-vs-Oscillibacter	0.651169	2.43E-09	5.34E-07
Vigabatrin-vs-Subdoligranulum	0.490422	2.52E-05	0.000374
Vigabatrin-vs-Bilophila	0.460292	8.89E-05	0.000915
Vigabatrin-vs-Anaerotruncus	0.581611	2.45E-07	1.26E-05
Vigabatrin-vs-Butyricicoccus	0.533921	3.27E-06	8.64E-05
Vigabatrin-vs-Paraprevotella	0.17675	0.15248	0.280921
Vigabatrin-vs-Collinsella	0.401149	0.000768	0.004773
Vigabatrin-vs-Parasutterella	0.127345	0.304452	0.46612
Vigabatrin-vs-Pyramidobacter	0.457539	9.91E-05	0.000994
Vigabatrin-vs-Intestinimonas	0.651688	2.33E-09	5.34E-07
Vigabatrin-vs-Pseudoflavonifractor	0.678825	2.73E-10	1.48E-07
Vigabatrin-vs-Holdemania	0.515005	8.23E-06	0.000165
Vigabatrin-vs-Oribacterium	0.37086	0.002005	0.00994
Vigabatrin-vs-Bacteroides	-0.05591	0.653154	0.764013
Vigabatrin-vs-Eubacterium	-0.0099	0.936644	0.962508
Vigabatrin-vs-Veillonella	-0.46273	8.06E-05	0.000871
Vigabatrin-vs-Lactobacillus	-0.08676	0.485112	0.629476
Vigabatrin-vs-Agathobacter	-0.10663	0.390422	0.548769
Vigabatrin-vs-Enterococcus	-0.25908	0.034257	0.090963
Vigabatrin-vs-Blautia	-0.07906	0.524826	0.660194
Vigabatrin-vs-Streptococcus	-0.37118	0.001986	0.009864

Vigabatrin-vs-Erysipelatoclostridium	-0.10001	0.420694	0.57333
Vigabatrin-vs-Lachnospira	-0.10029	0.419391	0.572198
Vigabatrin-vs-Fusobacterium	0.029771	0.810987	0.878291
Vigabatrin-vs-Bacillus	0.018916	0.879238	0.923926
Vigabatrin-vs-Dorea	-0.10516	0.397055	0.55327
Vigabatrin-vs-Tyzzerella	-0.0253	0.838951	0.897975
Vigabatrin-vs-Butyrivibrio	0.137002	0.26893	0.429684
Vigabatrin-vs-Mycoplasma	0.184731	0.134511	0.257218
Vigabatrin-vs-Coprobacillus	-0.16586	0.179808	0.319116
Vigabatrin-vs-Selenomonas	-0.07147	0.565449	0.696446
Vigabatrin-vs-Anaerostipes	-0.14606	0.238239	0.39311
Vigabatrin-vs-Peptoclostridium	0.006226	0.960123	0.975856
Vigabatrin-vs-Dysgonomonas	0.015564	0.900519	0.939071
Vigabatrin-vs-Coprobacter	-0.00176	0.988748	0.992853
Vigabatrin-vs-Capnocytophaga	-0.1645	0.183446	0.323627
Vigabatrin-vs-Campylobacter	-0.03025	0.808004	0.876485
Vigabatrin-vs-Flavobacterium	-0.1643	0.183985	0.32368
Gluconic acid-vs-Prevotella	0.212507	0.084259	0.182965
Gluconic acid-vs-Alistipes	0.041823	0.73684	0.829417
Gluconic acid-vs-Faecalibacterium	0.273406	0.025179	0.071614
Gluconic acid-vs-Oscillibacter	0.179623	0.145816	0.271958
Gluconic acid-vs-Subdoligranulum	0.102762	0.407951	0.559747
Gluconic acid-vs-Bilophila	0.104557	0.399763	0.554488
Gluconic acid-vs-Anaerotruncus	0.091188	0.463013	0.610038
Gluconic acid-vs-Butyricicoccus	0.381874	0.001429	0.007597

Gluconic acid-vs-Paraprevotella	0.010416	0.933331	0.961144
Gluconic acid-vs-Collinsella	0.276079	0.023733	0.068898
Gluconic acid-vs-Parasutterella	0.106673	0.390244	0.548769
Gluconic acid-vs-Pyramidobacter	0.182616	0.139109	0.263511
Gluconic acid-vs-Intestinimonas	0.167132	0.176432	0.314047
Gluconic acid-vs-Pseudoflavonifractor	0.164339	0.183877	0.32368
Gluconic acid-vs-Holdemania	0.093463	0.451882	0.603184
Gluconic acid-vs-Oribacterium	-0.03073	0.805024	0.875238
Gluconic acid-vs-Bacteroides	-0.32908	0.006546	0.025135
Gluconic acid-vs-Eubacterium	-0.25585	0.036645	0.096352
Gluconic acid-vs-Veillonella	-0.37613	0.001708	0.008732
Gluconic acid-vs-Lactobacillus	-0.31675	0.009015	0.032354
Gluconic acid-vs-Agathobacter	-0.20528	0.095614	0.202177
Gluconic acid-vs-Enterococcus	-0.32564	0.007165	0.027252
Gluconic acid-vs-Blautia	-0.24272	0.047812	0.115657
Gluconic acid-vs-Streptococcus	-0.20385	0.098009	0.20616
Gluconic acid-vs-Erysipelatoclostridium	-0.36643	0.00229	0.010993
Gluconic acid-vs-Lachnospira	-0.22819	0.063275	0.146611
Gluconic acid-vs-Fusobacterium	-0.45379	0.000115	0.001094
Gluconic acid-vs-Bacillus	-0.31228	0.010092	0.035534
Gluconic acid-vs-Dorea	-0.19738	0.10937	0.222545
Gluconic acid-vs-Tyzzerella	-0.26403	0.030854	0.083814
Gluconic acid-vs-Butyrivibrio	-0.27081	0.026652	0.074277
Gluconic acid-vs-Mycoplasma	-0.36934	0.002099	0.010237
Gluconic acid-vs-Coprobacillus	-0.56014	8.28E-07	3.24E-05

Gluconic acid-vs-Selenomonas	-0.23873	0.051713	0.123855
Gluconic acid-vs-Anaerostipes	-0.26626	0.029412	0.080758
Gluconic acid-vs-Peptoclostridium	-0.13736	0.267665	0.428229
Gluconic acid-vs-Dysgonomonas	-0.28394	0.019882	0.060043
Gluconic acid-vs-Coprobacter	0.013728	0.912203	0.946214
Gluconic acid-vs-Capnocytophaga	-0.10552	0.395436	0.551649
Gluconic acid-vs-Campylobacter	-0.30314	0.012647	0.042296
Gluconic acid-vs-Flavobacterium	-0.14734	0.234115	0.388425
Adipic acid-vs-Prevotella	0.003113	0.980055	0.987403
Adipic acid-vs-Alistipes	0.464722	7.44E-05	0.000822
Adipic acid-vs-Faecalibacterium	0.492657	2.29E-05	0.000348
Adipic acid-vs-Oscillibacter	0.626546	1.42E-08	2.02E-06
Adipic acid-vs-Subdoligranulum	0.49581	1.99E-05	0.000318
Adipic acid-vs-Bilophila	0.436348	0.000223	0.001758
Adipic acid-vs-Anaerotruncus	0.602841	6.73E-08	6.26E-06
Adipic acid-vs-Butyricicoccus	0.465081	7.33E-05	0.000817
Adipic acid-vs-Paraprevotella	0.265743	0.029742	0.081364
Adipic acid-vs-Collinsella	0.321215	0.008039	0.029465
Adipic acid-vs-Parasutterella	0.058065	0.64069	0.757492
Adipic acid-vs-Pyramidobacter	0.473741	5.14E-05	0.000631
Adipic acid-vs-Intestinimonas	0.482521	3.55E-05	0.000481
Adipic acid-vs-Pseudoflavonifractor	0.464722	7.44E-05	0.000822
Adipic acid-vs-Holdemania	0.498723	1.74E-05	0.000287
Adipic acid-vs-Oribacterium	0.461529	8.46E-05	0.000886
Adipic acid-vs-Bacteroides	-0.18461	0.134768	0.257507

Adipic acid-vs-Eubacterium	0.243395	0.047173	0.114801
Adipic acid-vs-Veillonella	-0.4741	5.06E-05	0.000631
Adipic acid-vs-Lactobacillus	-0.13928	0.260985	0.421667
Adipic acid-vs-Agathobacter	0.162024	0.190216	0.330792
Adipic acid-vs-Enterococcus	-0.15069	0.223526	0.37466
Adipic acid-vs-Blautia	-0.15763	0.202666	0.347893
Adipic acid-vs-Streptococcus	-0.2768	0.023357	0.068072
Adipic acid-vs-Erysipelatoclostridium	-0.27105	0.026513	0.07406
Adipic acid-vs-Lachnospira	0.123873	0.317934	0.478285
Adipic acid-vs-Fusobacterium	-0.03759	0.762632	0.848849
Adipic acid-vs-Bacillus	-0.00263	0.983123	0.98936
Adipic acid-vs-Dorea	0.023984	0.84723	0.903116
Adipic acid-vs-Tyzzerella	-0.01401	0.910424	0.945605
Adipic acid-vs-Butyrivibrio	0.331671	0.00611	0.023648
Adipic acid-vs-Mycoplasma	0.15085	0.22303	0.374399
Adipic acid-vs-Coprobacillus	-0.17727	0.15126	0.279312
Adipic acid-vs-Selenomonas	0.084484	0.496673	0.638391
Adipic acid-vs-Anaerostipes	0.015205	0.902803	0.939304
Adipic acid-vs-Peptoclostridium	0.121678	0.326651	0.487157
Adipic acid-vs-Dysgonomonas	-0.05511	0.657795	0.767586
Adipic acid-vs-Coprobacter	0.214183	0.081783	0.179163
Adipic acid-vs-Capnocytophaga	0.006385	0.959101	0.975487
Adipic acid-vs-Campylobacter	0.086918	0.484306	0.629105
Adipic acid-vs-Flavobacterium	0.03448	0.781779	0.860775
DEHA-vs-Prevotella	0.184133	0.1358	0.259274

DEHA-vs-Alistipes	0.226554	0.065244	0.15028
DEHA-vs-Faecalibacterium	0.355695	0.003137	0.013976
DEHA-vs-Oscillibacter	0.355455	0.003159	0.014047
DEHA-vs-Subdoligranulum	0.160148	0.195466	0.337497
DEHA-vs-Bilophila	0.121398	0.327771	0.487925
DEHA-vs-Anaerotruncus	0.167372	0.175805	0.31316
DEHA-vs-Butyricicoccus	0.421622	0.00038	0.002722
DEHA-vs-Paraprevotella	0.159071	0.198529	0.341809
DEHA-vs-Collinsella	0.372655	0.001899	0.00959
DEHA-vs-Parasutterella	0.202889	0.099632	0.208125
DEHA-vs-Pyramidobacter	0.16801	0.174139	0.310879
DEHA-vs-Intestinimonas	0.286376	0.018803	0.057576
DEHA-vs-Pseudoflavonifractor	0.262232	0.032054	0.086636
DEHA-vs-Holdemania	0.141991	0.251717	0.410255
DEHA-vs-Oribacterium	0.168768	0.172175	0.308056
DEHA-vs-Bacteroides	-0.30234	0.012895	0.042829
DEHA-vs-Eubacterium	-0.02821	0.8207	0.885492
DEHA-vs-Veillonella	-0.3432	0.004465	0.018527
DEHA-vs-Lactobacillus	-0.35989	0.002778	0.012777
DEHA-vs-Agathobacter	-0.11581	0.350685	0.51072
DEHA-vs-Enterococcus	-0.45251	0.000121	0.001132
DEHA-vs-Blautia	-0.32361	0.007556	0.028119
DEHA-vs-Streptococcus	-0.37593	0.001718	0.008732
DEHA-vs-Erysipelatoclostridium	-0.58269	2.3E-07	1.24E-05
DEHA-vs-Lachnospira	-0.08436	0.497285	0.638608

DEHA-vs-Fusobacterium	-0.27337	0.025201	0.071614
DEHA-vs-Bacillus	-0.29041	0.017127	0.053459
DEHA-vs-Dorea	-0.06106	0.623542	0.743028
DEHA-vs-Tyzzerella	-0.20249	0.100314	0.209189
DEHA-vs-Butyrivibrio	-0.06844	0.582101	0.712963
DEHA-vs-Mycoplasma	-0.20293	0.099564	0.208125
DEHA-vs-Coprobacillus	-0.44425	0.000166	0.001403
DEHA-vs-Selenomonas	-0.35166	0.003521	0.01543
DEHA-vs-Anaerostipes	-0.23589	0.054638	0.129705
DEHA-vs-Peptoclostridium	-0.08001	0.5198	0.656604
DEHA-vs-Dysgonomonas	-0.18992	0.123719	0.242919
DEHA-vs-Coprobacter	-0.18369	0.136752	0.26068
DEHA-vs-Capnocytophaga	-0.14638	0.237203	0.392204
DEHA-vs-Campylobacter	-0.0937	0.450719	0.60237
DEHA-vs-Flavobacterium	-0.15676	0.205224	0.351834
behenic acid-vs-Prevotella	0.209474	0.08889	0.190963
behenic acid-vs-Alistipes	0.175313	0.155896	0.285866
behenic acid-vs-Faecalibacterium	0.481762	3.67E-05	0.00049
behenic acid-vs-Oscillibacter	0.365193	0.002376	0.011227
behenic acid-vs-Subdoligranulum	0.206162	0.094173	0.20018
behenic acid-vs-Bilophila	0.037433	0.763611	0.849275
behenic acid-vs-Anaerotruncus	0.236012	0.054512	0.129532
behenic acid-vs-Butyricicoccus	0.526938	4.63E-06	0.000108
behenic acid-vs-Paraprevotella	0.135645	0.273745	0.433087
behenic acid-vs-Collinsella	0.447202	0.000148	0.001304

behenic acid-vs-Parasutterella	0.218932	0.07508	0.1677
behenic acid-vs-Pyramidobacter	0.247147	0.043768	0.109487
behenic acid-vs-Intestinimonas	0.336779	0.005325	0.021327
behenic acid-vs-Pseudoflavonifractor	0.3535	0.003341	0.014804
behenic acid-vs-Holdemania	0.29747	0.014498	0.046512
behenic acid-vs-Oribacterium	0.183853	0.136405	0.260224
behenic acid-vs-Bacteroides	-0.26091	0.032959	0.088588
behenic acid-vs-Eubacterium	-0.02961	0.811982	0.878437
behenic acid-vs-Veillonella	-0.38203	0.001422	0.007593
behenic acid-vs-Lactobacillus	-0.20369	0.098278	0.206367
behenic acid-vs-Agathobacter	-0.01365	0.912711	0.946214
behenic acid-vs-Enterococcus	-0.41799	0.000432	0.003004
behenic acid-vs-Blautia	-0.24136	0.04911	0.118442
behenic acid-vs-Streptococcus	-0.23921	0.051232	0.122946
behenic acid-vs-Erysipelatoclostridium	-0.41192	0.000533	0.003554
behenic acid-vs-Lachnospira	-0.10448	0.400125	0.554538
behenic acid-vs-Fusobacterium	-0.35039	0.003651	0.015857
behenic acid-vs-Bacillus	-0.22839	0.063038	0.146342
behenic acid-vs-Dorea	-0.08536	0.492194	0.634657
behenic acid-vs-Tyzzerella	-0.16254	0.188782	0.329008
behenic acid-vs-Butyrivibrio	-0.03336	0.788684	0.866067
behenic acid-vs-Mycoplasma	-0.22105	0.072239	0.162404
behenic acid-vs-Coprobacillus	-0.49509	2.05E-05	0.000324
behenic acid-vs-Selenomonas	-0.08927	0.472502	0.618162
behenic acid-vs-Anaerostipes	-0.06936	0.577038	0.708915

behenic acid-vs-Peptoclostridium	-0.07954	0.52231	0.659086
behenic acid-vs-Dysgonomonas	-0.27093	0.026583	0.074168
behenic acid-vs-Coprobacter	0.037553	0.762877	0.848849
behenic acid-vs-Capnocytophaga	-0.11789	0.342061	0.503006
behenic acid-vs-Campylobacter	-0.1967	0.110619	0.223735
behenic acid-vs-Flavobacterium	-0.09426	0.448012	0.599746
para-Tolyl octanoate-vs-Prevotella	0.022069	0.859302	0.910092
para-Tolyl octanoate-vs-Alistipes	0.004909	0.968553	0.980823
para-Tolyl octanoate-vs-Faecalibacterium	-0.26395	0.030906	0.083814
para-Tolyl octanoate-vs-Oscillibacter	-0.17599	0.154276	0.283366
para-Tolyl octanoate-vs-Subdoligranulum	-0.06269	0.61425	0.73558
para-Tolyl octanoate-vs-Bilophila	-0.17998	0.144999	0.270851
para-Tolyl octanoate-vs-Anaerotruncus	0.063692	0.608614	0.730277
para-Tolyl octanoate-vs-Butyricicoccus	-0.18617	0.131453	0.25237
para-Tolyl octanoate-vs-Paraprevotella	-0.22548	0.066568	0.152634
para-Tolyl octanoate-vs-Collinsella	-0.13573	0.273461	0.433087
para-Tolyl octanoate-vs-Parasutterella	-0.24695	0.043944	0.109814
para-Tolyl octanoate-vs-Pyramidobacter	-0.11362	0.359951	0.519834
para-Tolyl octanoate-vs-Intestinimonas	-0.05747	0.644142	0.758977
para-Tolyl octanoate-vs-Pseudoflavonifractor	-0.05423	0.662915	0.77197
para-Tolyl octanoate-vs-Holdemania	-0.07566	0.542817	0.677541
para-Tolyl octanoate-vs-Oribacterium	-0.07838	0.5284	0.663655
para-Tolyl octanoate-vs-Bacteroides	0.09035	0.467152	0.613485
para-Tolyl octanoate-vs-Eubacterium	0.221366	0.071818	0.161726
para-Tolyl octanoate-vs-Veillonella	0.334584	0.005651	0.022262

para-Tolyl octanoate-vs-Lactobacillus	0.361761	0.00263	0.012233
para-Tolyl octanoate-vs-Agathobacter	0.082449	0.507137	0.647291
para-Tolyl octanoate-vs-Enterococcus	0.531607	3.67E-06	9.36E-05
para-Tolyl octanoate-vs-Blautia	0.247626	0.043348	0.108888
para-Tolyl octanoate-vs-Streptococcus	0.297789	0.014387	0.046281
para-Tolyl octanoate-vs-Erysipelatoclostridium	0.335821	0.005465	0.021743
para-Tolyl octanoate-vs-Lachnospira	0.056908	0.647372	0.760461
para-Tolyl octanoate-vs-Fusobacterium	0.522548	5.74E-06	0.00013
para-Tolyl octanoate-vs-Bacillus	0.368306	0.002165	0.010455
para-Tolyl octanoate-vs-Dorea	0.084245	0.497898	0.638608
para-Tolyl octanoate-vs-Tyzzerella	0.244433	0.04621	0.11337
para-Tolyl octanoate-vs-Butyrivibrio	0.231064	0.059932	0.139938
para-Tolyl octanoate-vs-Mycoplasma	0.380358	0.001498	0.007913
para-Tolyl octanoate-vs-Coprobacillus	0.319978	0.0083	0.030191
para-Tolyl octanoate-vs-Selenomonas	0.342046	0.00461	0.018891
para-Tolyl octanoate-vs-Anaerostipes	0.231982	0.058895	0.137649
para-Tolyl octanoate-vs-Peptoclostridium	0.130856	0.291199	0.452415
para-Tolyl octanoate-vs-Dysgonomonas	0.374771	0.00178	0.009028
para-Tolyl octanoate-vs-Coprobacter	0.019475	0.875699	0.92221
para-Tolyl octanoate-vs-Capnocytophaga	0.151608	0.220685	0.371702
para-Tolyl octanoate-vs-Campylobacter	-0.05791	0.64161	0.757914
para-Tolyl octanoate-vs-Flavobacterium	0.260595	0.033182	0.088988

Supplementary Table S6. Spearman’s correlation coefficients between faecal metabolites and microbial genera. Spearman’s correlations coefficients between marker CAGs and p values were calculated and p values

were corrected for multiple testing as q values with Benjamin & Hochberg method. SCC = Spearman's correlation coefficient.

Plasma metabolites vs. Microbial genera	SCC index	p value	q value
Acetophenone-vs-Alistipes	0.125378	0.385633	0.457858
Acetophenone-vs-Faecalibacterium	0.445762	0.001178	0.004878
Acetophenone-vs-Oscillibacter	0.249172	0.080987	0.14365
Acetophenone-vs-Subdoligranulum	0.114046	0.430343	0.498323
Acetophenone-vs-Bilophila	0.242257	0.090068	0.155106
Acetophenone-vs-Butyricicoccus	0.346267	0.013767	0.035125
Acetophenone-vs-Paraprevotella	-0.00898	0.95065	0.957198
Acetophenone-vs-Collinsella	0.339544	0.01585	0.039214
Acetophenone-vs-Parasutterella	0.192317	0.18089	0.258192
Acetophenone-vs-Pyramidobacter	-0.01282	0.929583	0.939412
Acetophenone-vs-Intestinimonas	0.057767	0.690279	0.727457
Acetophenone-vs-Pseudoflavonifractor	0.117887	0.41487	0.483335
Acetophenone-vs-Holdemania	0.064202	0.657802	0.69976
Acetophenone-vs-Oribacterium	0.134886	0.350334	0.426755
Acetophenone-vs-Eubacterium	-0.25397	0.075117	0.135461
Acetophenone-vs-Veillonella	-0.60778	2.86E-06	0.000078
Acetophenone-vs-Lactobacillus	-0.5976	4.6E-06	9.46E-05
Acetophenone-vs-Agathobacter	-0.23678	0.097804	0.166012
Acetophenone-vs-Enterococcus	-0.57897	1.06E-05	0.000159
Acetophenone-vs-Blautia	-0.37018	0.008143	0.022942
Acetophenone-vs-Streptococcus	-0.61152	2.39E-06	7.24E-05
Acetophenone-vs-Erysipelatoclostridium	-0.51088	0.00015	0.000992
Acetophenone-vs-Lachnospira	-0.09628	0.505973	0.566371
Acetophenone-vs-Fusobacterium	-0.43808	0.001463	0.005819

Acetophenone-vs-Bacillus	-0.36461	0.009234	0.025553
Acetophenone-vs-Dorea	-0.28163	0.047548	0.094451
Acetophenone-vs-Tyzzerella	-0.26002	0.068205	0.125269
Acetophenone-vs-Butyrivibrio	-0.29719	0.036089	0.076456
Acetophenone-vs-Coprobacillus	-0.61316	2.2E-06	6.93E-05
Acetophenone-vs-Selenomonas	-0.36884	0.008395	0.02352
Acetophenone-vs-Anaerostipes	-0.32034	0.023332	0.053487
Acetophenone-vs-Peptoclostridium	-0.1927	0.180004	0.25737
Acetophenone-vs-Dysgonomonas	-0.49215	0.000283	0.001578
Acetophenone-vs-Capnocytophaga	-0.137	0.342769	0.418977
Acetophenone-vs-Flavobacterium	-0.17724	0.218193	0.297591
2-Hydroxy-2-[3-(methylsulfanyl)propyl]succinic acid-vs-Alistipes	0.282977	0.046454	0.09302
2-Hydroxy-2-[3-(methylsulfanyl)propyl]succinic acid-vs-Faecalibacterium	0.428379	0.001912	0.00722
2-Hydroxy-2-[3-(methylsulfanyl)propyl]succinic acid-vs-Oscillibacter	0.397263	0.004282	0.013747
2-Hydroxy-2-[3-(methylsulfanyl)propyl]succinic acid-vs-Subdoligranulum	0.218247	0.127851	0.199693
2-Hydroxy-2-[3-(methylsulfanyl)propyl]succinic acid-vs-Bilophila	0.318511	0.024176	0.054916
2-Hydroxy-2-[3-(methylsulfanyl)propyl]succinic acid-vs-Butyricicoccus	0.502233	0.000202	0.001219
2-Hydroxy-2-[3-(methylsulfanyl)propyl]succinic acid-vs-Paraprevotella	0.10473	0.469179	0.533087

2-Hydroxy-2-[3-(methylsulfanyl)propyl]succinic acid-vs-Collinsella	0.407923	0.003276	0.011137
2-Hydroxy-2-[3-(methylsulfanyl)propyl]succinic acid-vs-Parasutterella	0.29066	0.040586	0.084046
2-Hydroxy-2-[3-(methylsulfanyl)propyl]succinic acid-vs-Pyramidobacter	0.218247	0.127851	0.199693
2-Hydroxy-2-[3-(methylsulfanyl)propyl]succinic acid-vs-Intestinimonas	0.184826	0.198805	0.277545
2-Hydroxy-2-[3-(methylsulfanyl)propyl]succinic acid-vs-Pseudoflavonifractor	0.233133	0.10324	0.172182
2-Hydroxy-2-[3-(methylsulfanyl)propyl]succinic acid-vs-Holdemania	0.105786	0.464683	0.529453
2-Hydroxy-2-[3-(methylsulfanyl)propyl]succinic acid-vs-Oribacterium	0.157167	0.275704	0.356094
2-Hydroxy-2-[3-(methylsulfanyl)propyl]succinic acid-vs-Eubacterium	-0.24831	0.082081	0.144661
2-Hydroxy-2-[3-(methylsulfanyl)propyl]succinic acid-vs-Veillonella	-0.60307	3.57E-06	8.88E-05
2-Hydroxy-2-[3-(methylsulfanyl)propyl]succinic acid-vs-Lactobacillus	-0.49628	0.000247	0.001424
2-Hydroxy-2-[3-(methylsulfanyl)propyl]succinic acid-vs-Agathobacter	-0.29709	0.036152	0.076524
2-Hydroxy-2-[3-(methylsulfanyl)propyl]succinic acid-vs-Enterococcus	-0.58406	8.48E-06	0.000138

2-Hydroxy-2-[3-(methylsulfanyl)propyl]succinic acid-vs-Blautia	-0.36125	0.009952	0.027177
2-Hydroxy-2-[3-(methylsulfanyl)propyl]succinic acid-vs-Streptococcus	-0.5807	9.83E-06	0.000151
2-Hydroxy-2-[3-(methylsulfanyl)propyl]succinic acid-vs-Erysipelatoclostridium	-0.47169	0.000544	0.002642
2-Hydroxy-2-[3-(methylsulfanyl)propyl]succinic acid-vs-Lachnospira	-0.10992	0.447331	0.514621
2-Hydroxy-2-[3-(methylsulfanyl)propyl]succinic acid-vs-Fusobacterium	-0.4886	0.000318	0.001715
2-Hydroxy-2-[3-(methylsulfanyl)propyl]succinic acid-vs-Bacillus	-0.30727	0.02996	0.065653
2-Hydroxy-2-[3-(methylsulfanyl)propyl]succinic acid-vs-Dorea	-0.31669	0.025046	0.05653
2-Hydroxy-2-[3-(methylsulfanyl)propyl]succinic acid-vs-Tyzzerella	-0.33282	0.018194	0.043642
2-Hydroxy-2-[3-(methylsulfanyl)propyl]succinic acid-vs-Butyrivibrio	-0.25176	0.077774	0.139544
2-Hydroxy-2-[3-(methylsulfanyl)propyl]succinic acid-vs-Coprobacillus	-0.59068	6.31E-06	0.000116
2-Hydroxy-2-[3-(methylsulfanyl)propyl]succinic acid-vs-Selenomonas	-0.35635	0.011084	0.029459
2-Hydroxy-2-[3-(methylsulfanyl)propyl]succinic acid-vs-Anaerostipes	-0.41724	0.002574	0.009125

2-Hydroxy-2-[3-(methylsulfanyl)propyl]succinic acid-vs-Peptoclostridium	-0.22142	0.122273	0.194401
2-Hydroxy-2-[3-(methylsulfanyl)propyl]succinic acid-vs-Dysgonomonas	-0.35232	0.012098	0.031611
2-Hydroxy-2-[3-(methylsulfanyl)propyl]succinic acid-vs-Capnocytophaga	-0.16303	0.257974	0.337758
2-Hydroxy-2-[3-(methylsulfanyl)propyl]succinic acid-vs-Flavobacterium	-0.17273	0.230334	0.309394
L-(+)-Leucine-vs-Alistipes	0.048451	0.738279	0.767946
L-(+)-Leucine-vs-Faecalibacterium	0.385834	0.005652	0.017046
L-(+)-Leucine-vs-Oscillibacter	0.113661	0.431907	0.499902
L-(+)-Leucine-vs-Subdoligranulum	0.070156	0.628295	0.675308
L-(+)-Leucine-vs-Bilophila	0.151597	0.293292	0.372804
L-(+)-Leucine-vs-Butyricicoccus	0.337047	0.016689	0.040858
L-(+)-Leucine-vs-Paraprevotella	0.159472	0.268635	0.347867
L-(+)-Leucine-vs-Collinsella	0.158223	0.272449	0.352623
L-(+)-Leucine-vs-Parasutterella	0.129124	0.371484	0.444885
L-(+)-Leucine-vs-Pyramidobacter	-0.00879	0.951704	0.957872
L-(+)-Leucine-vs-Intestinimonas	0.035774	0.805184	0.829553
L-(+)-Leucine-vs-Pseudoflavonifractor	-0.06651	0.646315	0.689903
L-(+)-Leucine-vs-Holdemania	-0.06199	0.668884	0.70912
L-(+)-Leucine-vs-Oribacterium	0.027035	0.852159	0.870372
L-(+)-Leucine-vs-Eubacterium	-0.23285	0.103679	0.172682
L-(+)-Leucine-vs-Veillonella	-0.53364	6.59E-05	0.000557
L-(+)-Leucine-vs-Lactobacillus	-0.58953	6.65E-06	0.000119

L-(+)-Leucine-vs-Agathobacter	-0.31966	0.02364	0.053994
L-(+)-Leucine-vs-Enterococcus	-0.55928	2.42E-05	0.000276
L-(+)-Leucine-vs-Blautia	-0.45517	0.000896	0.0039
L-(+)-Leucine-vs-Streptococcus	-0.54046	5.09E-05	0.000458
L-(+)-Leucine-vs-Erysipelatoclostridium	-0.6001	4.1E-06	9.26E-05
L-(+)-Leucine-vs-Lachnospira	-0.18761	0.192003	0.270634
L-(+)-Leucine-vs-Fusobacterium	-0.44595	0.001171	0.004859
L-(+)-Leucine-vs-Bacillus	-0.36355	0.009455	0.02599
L-(+)-Leucine-vs-Dorea	-0.34531	0.01405	0.035732
L-(+)-Leucine-vs-Tyzzerella	-0.3577	0.010763	0.028973
L-(+)-Leucine-vs-Butyrivibrio	-0.34982	0.012765	0.033007
L-(+)-Leucine-vs-Coprobacillus	-0.61354	2.16E-06	6.93E-05
L-(+)-Leucine-vs-Selenomonas	-0.32821	0.019967	0.047209
L-(+)-Leucine-vs-Anaerostipes	-0.39611	0.004405	0.013981
L-(+)-Leucine-vs-Peptoclostridium	-0.31899	0.023952	0.054605
L-(+)-Leucine-vs-Dysgonomonas	-0.38583	0.005652	0.017046
L-(+)-Leucine-vs-Capnocytophaga	0.006675	0.963307	0.968373
L-(+)-Leucine-vs-Flavobacterium	-0.15073	0.296086	0.375012
16-{[(2E)-3-(4-Hydroxy-3-methoxyphenyl)-2-propenoyl]oxy}hexadecanoic acid-vs-Alistipes	0.196831	0.170673	0.246582
16-{[(2E)-3-(4-Hydroxy-3-methoxyphenyl)-2-propenoyl]oxy}hexadecanoic acid-vs-Faecalibacterium	0.415414	0.0027	0.009463
16-{[(2E)-3-(4-Hydroxy-3-methoxyphenyl)-2-propenoyl]oxy}hexadecanoic acid-vs-Oscillibacter	0.220072	0.124617	0.196244

16-{[(2E)-3-(4-Hydroxy-3-methoxyphenyl)-2-propenoyl]oxy}hexadecanoic acid-vs-Subdoligranulum	0.17042	0.236709	0.315909
16-{[(2E)-3-(4-Hydroxy-3-methoxyphenyl)-2-propenoyl]oxy}hexadecanoic acid-vs-Bilophila	0.172821	0.230071	0.309375
16-{[(2E)-3-(4-Hydroxy-3-methoxyphenyl)-2-propenoyl]oxy}hexadecanoic acid-vs-Butyricicoccus	0.27078	0.057174	0.108871
16-{[(2E)-3-(4-Hydroxy-3-methoxyphenyl)-2-propenoyl]oxy}hexadecanoic acid-vs-Paraprevotella	0.179832	0.211424	0.290752
16-{[(2E)-3-(4-Hydroxy-3-methoxyphenyl)-2-propenoyl]oxy}hexadecanoic acid-vs-Collinsella	0.380648	0.006391	0.01893
16-{[(2E)-3-(4-Hydroxy-3-methoxyphenyl)-2-propenoyl]oxy}hexadecanoic acid-vs-Parasutterella	0.233709	0.102367	0.1713
16-{[(2E)-3-(4-Hydroxy-3-methoxyphenyl)-2-propenoyl]oxy}hexadecanoic acid-vs-Pyramidobacter	0.074286	0.608159	0.657648
16-{[(2E)-3-(4-Hydroxy-3-methoxyphenyl)-2-propenoyl]oxy}hexadecanoic acid-vs-Intestinimonas	0.044898	0.756863	0.785305
16-{[(2E)-3-(4-Hydroxy-3-methoxyphenyl)-2-propenoyl]oxy}hexadecanoic acid-vs-Pseudoflavonifractor	0.084274	0.560655	0.615111
16-{[(2E)-3-(4-Hydroxy-3-methoxyphenyl)-2-propenoyl]oxy}hexadecanoic acid-vs-Holdemania	0.049316	0.73378	0.764746
16-{[(2E)-3-(4-Hydroxy-3-methoxyphenyl)-2-propenoyl]oxy}hexadecanoic acid-vs-Oribacterium	0.033469	0.817514	0.841211
16-{[(2E)-3-(4-Hydroxy-3-methoxyphenyl)-2-propenoyl]oxy}hexadecanoic acid-vs-Eubacterium	-0.26098	0.067156	0.123524

16-{[(2E)-3-(4-Hydroxy-3-methoxyphenyl)-2-propenoyl]oxy}hexadecanoic acid-vs-Veillonella	-0.65637	2.27E-07	0.000035
16-{[(2E)-3-(4-Hydroxy-3-methoxyphenyl)-2-propenoyl]oxy}hexadecanoic acid-vs-Lactobacillus	-0.58118	9.62E-06	0.00015
16-{[(2E)-3-(4-Hydroxy-3-methoxyphenyl)-2-propenoyl]oxy}hexadecanoic acid-vs-Agathobacter	-0.2363	0.098506	0.166749
16-{[(2E)-3-(4-Hydroxy-3-methoxyphenyl)-2-propenoyl]oxy}hexadecanoic acid-vs-Enterococcus	-0.62228	1.4E-06	5.93E-05
16-{[(2E)-3-(4-Hydroxy-3-methoxyphenyl)-2-propenoyl]oxy}hexadecanoic acid-vs-Blautia	-0.40725	0.003333	0.011283
16-{[(2E)-3-(4-Hydroxy-3-methoxyphenyl)-2-propenoyl]oxy}hexadecanoic acid-vs-Streptococcus	-0.55822	2.53E-05	0.000279
16-{[(2E)-3-(4-Hydroxy-3-methoxyphenyl)-2-propenoyl]oxy}hexadecanoic acid-vs-Erysipelatoclostridium	-0.61671	1.85E-06	0.000063
16-{[(2E)-3-(4-Hydroxy-3-methoxyphenyl)-2-propenoyl]oxy}hexadecanoic acid-vs-Lachnospira	-0.20154	0.160473	0.235962
16-{[(2E)-3-(4-Hydroxy-3-methoxyphenyl)-2-propenoyl]oxy}hexadecanoic acid-vs-Fusobacterium	-0.55246	3.19E-05	0.000334
16-{[(2E)-3-(4-Hydroxy-3-methoxyphenyl)-2-propenoyl]oxy}hexadecanoic acid-vs-Bacillus	-0.52365	9.53E-05	0.000717
16-{[(2E)-3-(4-Hydroxy-3-methoxyphenyl)-2-propenoyl]oxy}hexadecanoic acid-vs-Dorea	-0.30151	0.033346	0.07156
16-{[(2E)-3-(4-Hydroxy-3-methoxyphenyl)-2-propenoyl]oxy}hexadecanoic acid-vs-Tyzzerella	-0.32245	0.022385	0.052037

16-{[(2E)-3-(4-Hydroxy-3-methoxyphenyl)-2-propenoyl]oxy}hexadecanoic acid-vs-Butyrivibrio	-0.35059	0.012556	0.03257
16-{[(2E)-3-(4-Hydroxy-3-methoxyphenyl)-2-propenoyl]oxy}hexadecanoic acid-vs-Coprobacillus	-0.65637	2.27E-07	0.000035
16-{[(2E)-3-(4-Hydroxy-3-methoxyphenyl)-2-propenoyl]oxy}hexadecanoic acid-vs-Selenomonas	-0.50944	0.000158	0.001008
16-{[(2E)-3-(4-Hydroxy-3-methoxyphenyl)-2-propenoyl]oxy}hexadecanoic acid-vs-Anaerostipes	-0.35971	0.010296	0.027993
16-{[(2E)-3-(4-Hydroxy-3-methoxyphenyl)-2-propenoyl]oxy}hexadecanoic acid-vs-Peptoclostridium	-0.27827	0.050377	0.098495
16-{[(2E)-3-(4-Hydroxy-3-methoxyphenyl)-2-propenoyl]oxy}hexadecanoic acid-vs-Dysgonomonas	-0.50415	0.000189	0.001159
16-{[(2E)-3-(4-Hydroxy-3-methoxyphenyl)-2-propenoyl]oxy}hexadecanoic acid-vs-Capnocytophaga	-0.09993	0.489907	0.551867
16-{[(2E)-3-(4-Hydroxy-3-methoxyphenyl)-2-propenoyl]oxy}hexadecanoic acid-vs-Flavobacterium	-0.26646	0.061419	0.11519
5b-Cholestane-3a,7a,12a,26-tetrol-vs-Alistipes	0.241873	0.090595	0.155475
5b-Cholestane-3a,7a,12a,26-tetrol-vs-Faecalibacterium	0.462953	0.000711	0.003212
5b-Cholestane-3a,7a,12a,26-tetrol-vs-Oscillibacter	0.253301	0.075918	0.136608
5b-Cholestane-3a,7a,12a,26-tetrol-vs-Subdoligranulum	0.205282	0.152679	0.22719
5b-Cholestane-3a,7a,12a,26-tetrol-vs-Bilophila	0.197887	0.168343	0.24421
5b-Cholestane-3a,7a,12a,26-tetrol-vs-Butyricicoccus	0.386603	0.005549	0.016879
5b-Cholestane-3a,7a,12a,26-tetrol-vs-Paraprevotella	0.203073	0.157241	0.231896
5b-Cholestane-3a,7a,12a,26-tetrol-vs-Collinsella	0.422041	0.002267	0.008334
5b-Cholestane-3a,7a,12a,26-tetrol-vs-Parasutterella	0.284898	0.044926	0.090691

5b-Cholestane-3a,7a,12a,26-tetrol-vs-Pyramidobacter	0.129412	0.370408	0.44424
5b-Cholestane-3a,7a,12a,26-tetrol-vs-Intestinimonas	0.093397	0.518844	0.577103
5b-Cholestane-3a,7a,12a,26-tetrol-vs-Pseudoflavonifractor	0.176471	0.220228	0.29938
5b-Cholestane-3a,7a,12a,26-tetrol-vs-Holdemania	0.072941	0.614684	0.662686
5b-Cholestane-3a,7a,12a,26-tetrol-vs-Oribacterium	0.142185	0.324631	0.401747
5b-Cholestane-3a,7a,12a,26-tetrol-vs-Eubacterium	-0.19481	0.175185	0.251784
5b-Cholestane-3a,7a,12a,26-tetrol-vs-Veillonella	-0.56812	1.68E-05	0.000214
5b-Cholestane-3a,7a,12a,26-tetrol-vs-Lactobacillus	-0.47659	0.000467	0.002336
5b-Cholestane-3a,7a,12a,26-tetrol-vs-Agathobacter	-0.24523	0.086065	0.149876
5b-Cholestane-3a,7a,12a,26-tetrol-vs-Enterococcus	-0.58569	7.89E-06	0.00013
5b-Cholestane-3a,7a,12a,26-tetrol-vs-Blautia	-0.3164	0.025186	0.056794
5b-Cholestane-3a,7a,12a,26-tetrol-vs-Streptococcus	-0.50022	0.000216	0.001284
5b-Cholestane-3a,7a,12a,26-tetrol-vs-Erysipelatoclostridium	-0.56139	2.22E-05	0.000259
5b-Cholestane-3a,7a,12a,26-tetrol-vs-Lachnospira	-0.0837	0.563347	0.61752
5b-Cholestane-3a,7a,12a,26-tetrol-vs-Fusobacterium	-0.51155	0.000147	0.000979
5b-Cholestane-3a,7a,12a,26-tetrol-vs-Bacillus	-0.43318	0.001677	0.006459
5b-Cholestane-3a,7a,12a,26-tetrol-vs-Dorea	-0.21892	0.126652	0.198694
5b-Cholestane-3a,7a,12a,26-tetrol-vs-Tyzzerella	-0.23188	0.105152	0.17397
5b-Cholestane-3a,7a,12a,26-tetrol-vs-Butyrivibrio	-0.28192	0.047312	0.094434
5b-Cholestane-3a,7a,12a,26-tetrol-vs-Coprobacillus	-0.62017	1.56E-06	5.97E-05
5b-Cholestane-3a,7a,12a,26-tetrol-vs-Selenomonas	-0.49657	0.000245	0.001417
5b-Cholestane-3a,7a,12a,26-tetrol-vs-Anaerostipes	-0.34521	0.014078	0.035735
5b-Cholestane-3a,7a,12a,26-tetrol-vs-Peptoclostridium	-0.20423	0.154849	0.229457
5b-Cholestane-3a,7a,12a,26-tetrol-vs-Dysgonomonas	-0.44701	0.001136	0.004738

5b-Cholestane-3a,7a,12a,26-tetrol-vs-Capnocytophaga	-0.17762	0.217181	0.297025
5b-Cholestane-3a,7a,12a,26-tetrol-vs-Flavobacterium	-0.17868	0.214414	0.294213
Gefarnate-vs-Alistipes	0.238031	0.095996	0.163726
Gefarnate-vs-Faecalibacterium	0.456999	0.000849	0.003722
Gefarnate-vs-Oscillibacter	0.355006	0.011414	0.030141
Gefarnate-vs-Subdoligranulum	0.209508	0.144225	0.218005
Gefarnate-vs-Bilophila	0.20557	0.152092	0.226451
Gefarnate-vs-Butyricicoccus	0.42012	0.002386	0.00868
Gefarnate-vs-Paraprevotella	0.231981	0.105004	0.173957
Gefarnate-vs-Collinsella	0.417047	0.002587	0.009158
Gefarnate-vs-Parasutterella	0.242545	0.089674	0.154536
Gefarnate-vs-Pyramidobacter	0.182041	0.205775	0.284875
Gefarnate-vs-Intestinimonas	0.081008	0.575992	0.628884
Gefarnate-vs-Pseudoflavonifractor	0.138631	0.336992	0.413544
Gefarnate-vs-Holdemania	0.199136	0.16562	0.24167
Gefarnate-vs-Oribacterium	0.143433	0.320357	0.397646
Gefarnate-vs-Eubacterium	-0.24936	0.080746	0.143426
Gefarnate-vs-Veillonella	-0.65032	3.19E-07	3.82E-05
Gefarnate-vs-Lactobacillus	-0.55112	3.36E-05	0.000345
Gefarnate-vs-Agathobacter	-0.20461	0.154057	0.22883
Gefarnate-vs-Enterococcus	-0.58828	7.03E-06	0.000121
Gefarnate-vs-Blautia	-0.34924	0.012923	0.033347
Gefarnate-vs-Streptococcus	-0.53508	6.24E-05	0.000539
Gefarnate-vs-Erysipelatoclostridium	-0.56293	2.09E-05	0.000248
Gefarnate-vs-Lachnospira	-0.05623	0.69812	0.734163

Gefarnate-vs-Fusobacterium	-0.532	0.00007	0.00058
Gefarnate-vs-Bacillus	-0.43366	0.001654	0.006404
Gefarnate-vs-Dorea	-0.21345	0.136665	0.209638
Gefarnate-vs-Tyzzerella	-0.29306	0.038882	0.081127
Gefarnate-vs-Butyrivibrio	-0.23745	0.096827	0.164918
Gefarnate-vs-Coprobacillus	-0.5831	8.85E-06	0.000143
Gefarnate-vs-Selenomonas	-0.49186	0.000286	0.001586
Gefarnate-vs-Anaerostipes	-0.39621	0.004395	0.013981
Gefarnate-vs-Peptoclostridium	-0.21758	0.129059	0.201199
Gefarnate-vs-Dysgonomonas	-0.52163	0.000102	0.000749
Gefarnate-vs-Capnocytophaga	-0.10492	0.46836	0.532665
Gefarnate-vs-Flavobacterium	-0.21364	0.136304	0.209213
cholic acid-vs-Alistipes	0.373637	0.007523	0.021489
cholic acid-vs-Faecalibacterium	0.461128	0.000751	0.003362
cholic acid-vs-Oscillibacter	0.428283	0.001917	0.007228
cholic acid-vs-Subdoligranulum	0.331381	0.018733	0.044676
cholic acid-vs-Bilophila	0.289412	0.041496	0.085574
cholic acid-vs-Butyricicoccus	0.334838	0.017462	0.042293
cholic acid-vs-Paraprevotella	0.290468	0.040725	0.084237
cholic acid-vs-Collinsella	0.410132	0.003096	0.010611
cholic acid-vs-Parasutterella	0.270588	0.057357	0.10897
cholic acid-vs-Pyramidobacter	0.269244	0.058655	0.110843
cholic acid-vs-Intestinimonas	0.228139	0.111053	0.180134
cholic acid-vs-Pseudoflavonifractor	0.248019	0.082449	0.145
cholic acid-vs-Holdemania	0.111068	0.442553	0.510086

cholic acid-vs-Oribacterium	0.064586	0.655882	0.698614
cholic acid-vs-Eubacterium	-0.20643	0.150338	0.224379
cholic acid-vs-Veillonella	-0.60288	3.6E-06	8.88E-05
cholic acid-vs-Lactobacillus	-0.46891	0.000593	0.002807
cholic acid-vs-Agathobacter	-0.20279	0.157843	0.232646
cholic acid-vs-Enterococcus	-0.53613	0.00006	0.000525
cholic acid-vs-Blautia	-0.40773	0.003292	0.011161
cholic acid-vs-Streptococcus	-0.51491	0.00013	0.000892
cholic acid-vs-Erysipelatoclostridium	-0.53047	7.42E-05	0.000596
cholic acid-vs-Lachnospira	-0.28163	0.047548	0.094451
cholic acid-vs-Fusobacterium	-0.49388	0.000268	0.001521
cholic acid-vs-Bacillus	-0.37431	0.007408	0.021183
cholic acid-vs-Dorea	-0.25128	0.078361	0.140092
cholic acid-vs-Tyzzerella	-0.3286	0.019814	0.046937
cholic acid-vs-Butyrivibrio	-0.21498	0.133797	0.206129
cholic acid-vs-Coprobacillus	-0.60058	4.01E-06	9.14E-05
cholic acid-vs-Selenomonas	-0.43731	0.001495	0.005917
cholic acid-vs-Anaerostipes	-0.32648	0.020668	0.048821
cholic acid-vs-Peptoclostridium	-0.20874	0.145736	0.219354
cholic acid-vs-Dysgonomonas	-0.35424	0.011606	0.030486
cholic acid-vs-Capnocytophaga	-0.12106	0.402349	0.471399
cholic acid-vs-Flavobacterium	-0.16187	0.2614	0.341883
L-Histidine-vs-Alistipes	0.18377	0.201429	0.27995
L-Histidine-vs-Faecalibacterium	0.371909	0.007828	0.022205
L-Histidine-vs-Oscillibacter	0.279808	0.049068	0.096619

L-Histidine-vs-Subdoligranulum	0.009268	0.949069	0.955993
L-Histidine-vs-Bilophila	0.341465	0.015229	0.038111
L-Histidine-vs-Butyricicoccus	0.373349	0.007573	0.021557
L-Histidine-vs-Paraprevotella	0.0109	0.940111	0.948124
L-Histidine-vs-Collinsella	0.244178	0.087469	0.151577
L-Histidine-vs-Parasutterella	0.095414	0.509818	0.569392
L-Histidine-vs-Pyramidobacter	0.108379	0.453746	0.519372
L-Histidine-vs-Intestinimonas	0.146218	0.310951	0.389276
L-Histidine-vs-Pseudoflavonifractor	0.136999	0.342769	0.418977
L-Histidine-vs-Holdemania	-0.10463	0.469589	0.533087
L-Histidine-vs-Oribacterium	0.010132	0.944326	0.951602
L-Histidine-vs-Eubacterium	-0.33551	0.017223	0.04192
L-Histidine-vs-Veillonella	-0.48504	0.000357	0.001888
L-Histidine-vs-Lactobacillus	-0.52173	0.000102	0.000749
L-Histidine-vs-Agathobacter	-0.32571	0.020986	0.049432
L-Histidine-vs-Enterococcus	-0.58108	9.66E-06	0.00015
L-Histidine-vs-Blautia	-0.36442	0.009274	0.025606
L-Histidine-vs-Streptococcus	-0.5636	2.03E-05	0.000242
L-Histidine-vs-Erysipelatoclostridium	-0.5249	0.000091	0.00069
L-Histidine-vs-Lachnospira	-0.24562	0.085559	0.149203
L-Histidine-vs-Fusobacterium	-0.50732	0.00017	0.001063
L-Histidine-vs-Bacillus	-0.35222	0.012123	0.031611
L-Histidine-vs-Dorea	-0.3625	0.00968	0.026492
L-Histidine-vs-Tyzzerella	-0.22507	0.11608	0.186705
L-Histidine-vs-Butyrivibrio	-0.34022	0.01563	0.038841

L-Histidine-vs-Coprobacillus	-0.61854	1.69E-06	6.18E-05
L-Histidine-vs-Selenomonas	-0.38036	0.006435	0.019036
L-Histidine-vs-Anaerostipes	-0.42761	0.001952	0.007316
L-Histidine-vs-Peptoclostridium	-0.27126	0.056717	0.108167
L-Histidine-vs-Dysgonomonas	-0.41926	0.002441	0.008829
L-Histidine-vs-Capnocytophaga	-0.05104	0.724809	0.757422
L-Histidine-vs-Flavobacterium	-0.17436	0.22589	0.304908
3-Carboxy-3-deoxypent-2-ulosaric acid-vs-Alistipes	0.293445	0.038615	0.080773
3-Carboxy-3-deoxypent-2-ulosaric acid-vs-Faecalibacterium	0.359232	0.010405	0.028198
3-Carboxy-3-deoxypent-2-ulosaric acid-vs-Oscillibacter	0.345498	0.013993	0.035627
3-Carboxy-3-deoxypent-2-ulosaric acid-vs-Subdoligranulum	0.19904	0.165828	0.241832
3-Carboxy-3-deoxypent-2-ulosaric acid-vs-Bilophila	0.316783	0.025	0.056476
3-Carboxy-3-deoxypent-2-ulosaric acid-vs-Butyricicoccus	0.388331	0.005324	0.016313
3-Carboxy-3-deoxypent-2-ulosaric acid-vs-Paraprevotella	0.143049	0.321668	0.399074
3-Carboxy-3-deoxypent-2-ulosaric acid-vs-Collinsella	0.37892	0.006656	0.019497
3-Carboxy-3-deoxypent-2-ulosaric acid-vs-Parasutterella	0.234094	0.101787	0.170791
3-Carboxy-3-deoxypent-2-ulosaric acid-vs-Pyramidobacter	0.257719	0.070775	0.128942
3-Carboxy-3-deoxypent-2-ulosaric acid-vs-Intestinimonas	0.274238	0.053951	0.104251
3-Carboxy-3-deoxypent-2-ulosaric acid-vs-Pseudoflavonifractor	0.241297	0.091389	0.156612
3-Carboxy-3-deoxypent-2-ulosaric acid-vs-Holdemania	0.011092	0.939058	0.947831
3-Carboxy-3-deoxypent-2-ulosaric acid-vs-Oribacterium	-0.01589	0.912763	0.925047

3-Carboxy-3-deoxypent-2-ulosaric acid-vs-Eubacterium	-0.26185	0.066222	0.122725
3-Carboxy-3-deoxypent-2-ulosaric acid-vs-Veillonella	-0.52922	7.77E-05	0.000619
3-Carboxy-3-deoxypent-2-ulosaric acid-vs-Lactobacillus	-0.46487	0.000671	0.003081
3-Carboxy-3-deoxypent-2-ulosaric acid-vs-Agathobacter	-0.33791	0.016394	0.040296
3-Carboxy-3-deoxypent-2-ulosaric acid-vs-Enterococcus	-0.59433	5.35E-06	0.000103
3-Carboxy-3-deoxypent-2-ulosaric acid-vs-Blautia	-0.40389	0.003629	0.012137
3-Carboxy-3-deoxypent-2-ulosaric acid-vs-Streptococcus	-0.61921	1.64E-06	6.16E-05
3-Carboxy-3-deoxypent-2-ulosaric acid-vs-Erysipelatoclostridium	-0.59453	5.3E-06	0.000103
3-Carboxy-3-deoxypent-2-ulosaric acid-vs-Lachnospira	-0.38574	0.005665	0.017064
3-Carboxy-3-deoxypent-2-ulosaric acid-vs-Fusobacterium	-0.50944	0.000158	0.001008
3-Carboxy-3-deoxypent-2-ulosaric acid-vs-Bacillus	-0.39611	0.004405	0.013981
3-Carboxy-3-deoxypent-2-ulosaric acid-vs-Dorea	-0.33916	0.015976	0.039425
3-Carboxy-3-deoxypent-2-ulosaric acid-vs-Tyzzerella	-0.31419	0.026279	0.058725
3-Carboxy-3-deoxypent-2-ulosaric acid-vs-Butyrivibrio	-0.31745	0.024677	0.056001
3-Carboxy-3-deoxypent-2-ulosaric acid-vs-Coprobacillus	-0.65858	2.01E-07	0.000035
3-Carboxy-3-deoxypent-2-ulosaric acid-vs-Selenomonas	-0.37095	0.008001	0.022595
3-Carboxy-3-deoxypent-2-ulosaric acid-vs-Anaerostipes	-0.38996	0.005118	0.015839
3-Carboxy-3-deoxypent-2-ulosaric acid-vs-Peptoclostridium	-0.27798	0.050626	0.098904
3-Carboxy-3-deoxypent-2-ulosaric acid-vs-Dysgonomonas	-0.2896	0.041355	0.085354
3-Carboxy-3-deoxypent-2-ulosaric acid-vs-Capnocytophaga	-0.10463	0.469589	0.533087
3-Carboxy-3-deoxypent-2-ulosaric acid-vs-Flavobacterium	-0.19049	0.185143	0.262603

5-Methoxy-3-indoleaceate-vs-Alistipes	0.171285	0.234304	0.313541
5-Methoxy-3-indoleaceate-vs-Faecalibacterium	0.428475	0.001907	0.007212
5-Methoxy-3-indoleaceate-vs-Oscillibacter	0.287587	0.042855	0.088013
5-Methoxy-3-indoleaceate-vs-Subdoligranulum	0.176375	0.220483	0.299563
5-Methoxy-3-indoleaceate-vs-Bilophila	0.254454	0.074549	0.134828
5-Methoxy-3-indoleaceate-vs-Butyricicoccus	0.395438	0.004479	0.014142
5-Methoxy-3-indoleaceate-vs-Paraprevotella	0.147083	0.308068	0.386836
5-Methoxy-3-indoleaceate-vs-Collinsella	0.308331	0.029371	0.064591
5-Methoxy-3-indoleaceate-vs-Parasutterella	0.160048	0.266886	0.346868
5-Methoxy-3-indoleaceate-vs-Pyramidobacter	0.043265	0.765448	0.793219
5-Methoxy-3-indoleaceate-vs-Intestinimonas	0.101273	0.484055	0.546266
5-Methoxy-3-indoleaceate-vs-Pseudoflavonifractor	0.11539	0.424891	0.493159
5-Methoxy-3-indoleaceate-vs-Holdemania	0.10982	0.447731	0.514621
5-Methoxy-3-indoleaceate-vs-Oribacterium	0.15515	0.28199	0.361023
5-Methoxy-3-indoleaceate-vs-Eubacterium	-0.25061	0.07919	0.141471
5-Methoxy-3-indoleaceate-vs-Veillonella	-0.61095	2.45E-06	7.26E-05
5-Methoxy-3-indoleaceate-vs-Lactobacillus	-0.52711	0.000084	0.000662
5-Methoxy-3-indoleaceate-vs-Agathobacter	-0.25052	0.079308	0.141581
5-Methoxy-3-indoleaceate-vs-Enterococcus	-0.54411	4.42E-05	0.000418
5-Methoxy-3-indoleaceate-vs-Blautia	-0.33378	0.017842	0.043047
5-Methoxy-3-indoleaceate-vs-Streptococcus	-0.52509	9.04E-05	0.000687
5-Methoxy-3-indoleaceate-vs-Erysipelatoclostridium	-0.52115	0.000104	0.00076
5-Methoxy-3-indoleaceate-vs-Lachnospira	-0.01522	0.916439	0.928016
5-Methoxy-3-indoleaceate-vs-Fusobacterium	-0.42022	0.00238	0.008671
5-Methoxy-3-indoleaceate-vs-Bacillus	-0.37565	0.007181	0.02075

5-Methoxy-3-indoleaceate-vs-Dorea	-0.28173	0.04747	0.094445
5-Methoxy-3-indoleaceate-vs-Tyzzerella	-0.25964	0.068628	0.12586
5-Methoxy-3-indoleaceate-vs-Butyrivibrio	-0.24399	0.087727	0.151917
5-Methoxy-3-indoleaceate-vs-Coprobacillus	-0.57666	1.17E-05	0.000168
5-Methoxy-3-indoleaceate-vs-Selenomonas	-0.45393	0.00093	0.00399
5-Methoxy-3-indoleaceate-vs-Anaerostipes	-0.37758	0.006868	0.020007
5-Methoxy-3-indoleaceate-vs-Peptoclostridium	-0.18694	0.193629	0.272001
5-Methoxy-3-indoleaceate-vs-Dysgonomonas	-0.48543	0.000353	0.001873
5-Methoxy-3-indoleaceate-vs-Capnocytophaga	-0.05345	0.71241	0.746349
5-Methoxy-3-indoleaceate-vs-Flavobacterium	-0.22353	0.118658	0.189624
gitogenin-vs-Alistipes	0.225354	0.115602	0.186056
gitogenin-vs-Faecalibacterium	0.412533	0.00291	0.010114
gitogenin-vs-Oscillibacter	0.267323	0.060551	0.114077
gitogenin-vs-Subdoligranulum	0.181657	0.20675	0.286065
gitogenin-vs-Bilophila	0.174166	0.22641	0.305444
gitogenin-vs-Butyricicoccus	0.342233	0.014987	0.03758
gitogenin-vs-Paraprevotella	0.147179	0.307749	0.386631
gitogenin-vs-Collinsella	0.392941	0.004761	0.014939
gitogenin-vs-Parasutterella	0.294982	0.037562	0.078902
gitogenin-vs-Pyramidobacter	0.145834	0.312238	0.390494
gitogenin-vs-Intestinimonas	0.088595	0.540654	0.596592
gitogenin-vs-Pseudoflavonifractor	0.075438	0.60259	0.653047
gitogenin-vs-Holdemania	0.002065	0.988645	0.98984
gitogenin-vs-Oribacterium	0.02377	0.869851	0.887712
gitogenin-vs-Eubacterium	-0.22939	0.109058	0.178296

gitogenin-vs-Veillonella	-0.57369	1.33E-05	0.000185
gitogenin-vs-Lactobacillus	-0.57167	1.45E-05	0.000199
gitogenin-vs-Agathobacter	-0.2291	0.109516	0.178575
gitogenin-vs-Enterococcus	-0.59481	5.23E-06	0.000103
gitogenin-vs-Blautia	-0.42396	0.002154	0.007976
gitogenin-vs-Streptococcus	-0.55496	2.88E-05	0.000306
gitogenin-vs-Erysipelatoclostridium	-0.56091	2.27E-05	0.000261
gitogenin-vs-Lachnospira	-0.11885	0.411053	0.479111
gitogenin-vs-Fusobacterium	-0.58118	9.62E-06	0.00015
gitogenin-vs-Bacillus	-0.39198	0.004874	0.015216
gitogenin-vs-Dorea	-0.28442	0.045304	0.091158
gitogenin-vs-Tyzzerella	-0.33503	0.017393	0.042168
gitogenin-vs-Butyrivibrio	-0.30372	0.032013	0.069296
gitogenin-vs-Coprobacillus	-0.62142	1.47E-06	5.93E-05
gitogenin-vs-Selenomonas	-0.40216	0.00379	0.012508
gitogenin-vs-Anaerostipes	-0.40418	0.003603	0.012082
gitogenin-vs-Peptoclostridium	-0.26175	0.066326	0.122725
gitogenin-vs-Dysgonomonas	-0.44077	0.001357	0.005492
gitogenin-vs-Capnocytophaga	-0.07717	0.594278	0.645729
gitogenin-vs-Flavobacterium	-0.17782	0.216676	0.296662
Reomol DiDP-vs-Alistipes	0.319664	0.02364	0.053994
Reomol DiDP-vs-Faecalibacterium	0.507419	0.000169	0.001062
Reomol DiDP-vs-Oscillibacter	0.416471	0.002626	0.009271
Reomol DiDP-vs-Subdoligranulum	0.347707	0.013353	0.034173
Reomol DiDP-vs-Bilophila	0.248115	0.082326	0.144887

Reomol DiDP-vs-Butyricicoccus	0.441056	0.001346	0.005457
Reomol DiDP-vs-Paraprevotella	0.296423	0.036596	0.077331
Reomol DiDP-vs-Collinsella	0.395438	0.004479	0.014142
Reomol DiDP-vs-Parasutterella	0.266651	0.061225	0.115174
Reomol DiDP-vs-Pyramidobacter	0.248115	0.082326	0.144887
Reomol DiDP-vs-Intestinimonas	0.185978	0.19597	0.274203
Reomol DiDP-vs-Pseudoflavonifractor	0.198175	0.167712	0.243579
Reomol DiDP-vs-Holdemania	0.129988	0.368263	0.442307
Reomol DiDP-vs-Oribacterium	0.192221	0.181112	0.25836
Reomol DiDP-vs-Eubacterium	-0.19357	0.178021	0.254975
Reomol DiDP-vs-Veillonella	-0.60269	3.63E-06	8.88E-05
Reomol DiDP-vs-Lactobacillus	-0.52547	8.91E-05	0.000686
Reomol DiDP-vs-Agathobacter	-0.15265	0.289902	0.369629
Reomol DiDP-vs-Enterococcus	-0.48091	0.000408	0.002084
Reomol DiDP-vs-Blautia	-0.38151	0.006263	0.018593
Reomol DiDP-vs-Streptococcus	-0.4399	0.001391	0.0056
Reomol DiDP-vs-Erysipelatoclostridium	-0.51645	0.000123	0.000852
Reomol DiDP-vs-Lachnospira	-0.10934	0.449731	0.516425
Reomol DiDP-vs-Fusobacterium	-0.43577	0.001561	0.006088
Reomol DiDP-vs-Bacillus	-0.36989	0.008196	0.023067
Reomol DiDP-vs-Dorea	-0.19846	0.167082	0.242948
Reomol DiDP-vs-Tyzzerella	-0.28038	0.048584	0.096046
Reomol DiDP-vs-Butyrivibrio	-0.13431	0.352415	0.42845
Reomol DiDP-vs-Coprobacillus	-0.5613	2.23E-05	0.000259
Reomol DiDP-vs-Selenomonas	-0.48293	0.000382	0.001991

Reomol DiDP-vs-Anaerostipes	-0.3406	0.015506	0.038609
Reomol DiDP-vs-Peptoclostridium	-0.15304	0.288675	0.368254
Reomol DiDP-vs-Dysgonomonas	-0.41215	0.002939	0.0102
Reomol DiDP-vs-Capnocytophaga	-0.05076	0.726302	0.758663
Reomol DiDP-vs-Flavobacterium	-0.18425	0.200233	0.278912
Adipostatin A-vs-Alistipes	0.258872	0.069481	0.127049
Adipostatin A-vs-Faecalibacterium	0.478319	0.000442	0.002226
Adipostatin A-vs-Oscillibacter	0.305546	0.030944	0.067335
Adipostatin A-vs-Subdoligranulum	0.292581	0.039218	0.081694
Adipostatin A-vs-Bilophila	0.147275	0.30743	0.386425
Adipostatin A-vs-Butyricicoccus	0.323313	0.022008	0.051448
Adipostatin A-vs-Paraprevotella	0.259832	0.068416	0.125564
Adipostatin A-vs-Collinsella	0.401873	0.003818	0.012566
Adipostatin A-vs-Parasutterella	0.271741	0.056263	0.107632
Adipostatin A-vs-Pyramidobacter	0.187323	0.192699	0.271
Adipostatin A-vs-Intestinimonas	0.093301	0.519276	0.577103
Adipostatin A-vs-Pseudoflavonifractor	0.132197	0.36011	0.435038
Adipostatin A-vs-Holdemania	0.109244	0.450131	0.516425
Adipostatin A-vs-Oribacterium	0.085714	0.553949	0.60883
Adipostatin A-vs-Eubacterium	-0.21825	0.127851	0.199693
Adipostatin A-vs-Veillonella	-0.65445	2.54E-07	0.000035
Adipostatin A-vs-Lactobacillus	-0.54833	3.75E-05	0.000373
Adipostatin A-vs-Agathobacter	-0.20327	0.15684	0.231513
Adipostatin A-vs-Enterococcus	-0.57013	1.55E-05	0.000205
Adipostatin A-vs-Blautia	-0.4011	0.003892	0.012742

Adipostatin A-vs-Streptococcus	-0.4885	0.000319	0.001717
Adipostatin A-vs-Erysipelatoclostridium	-0.5564	2.72E-05	0.000295
Adipostatin A-vs-Lachnospira	-0.14747	0.306792	0.38621
Adipostatin A-vs-Fusobacterium	-0.54094	0.00005	0.000452
Adipostatin A-vs-Bacillus	-0.40139	0.003864	0.012684
Adipostatin A-vs-Dorea	-0.23381	0.102222	0.171173
Adipostatin A-vs-Tyzzerella	-0.33695	0.016722	0.040899
Adipostatin A-vs-Butyrivibrio	-0.2411	0.091655	0.156862
Adipostatin A-vs-Coprobacillus	-0.60567	3.16E-06	8.35E-05
Adipostatin A-vs-Selenomonas	-0.45345	0.000943	0.004026
Adipostatin A-vs-Anaerostipes	-0.35731	0.010854	0.029002
Adipostatin A-vs-Peptoclostridium	-0.21556	0.132733	0.20487
Adipostatin A-vs-Dysgonomonas	-0.43712	0.001503	0.005939
Adipostatin A-vs-Capnocytophaga	-0.09397	0.516257	0.575291
Adipostatin A-vs-Flavobacterium	-0.19712	0.170035	0.246233
Ecdysone-vs-Alistipes	0.169364	0.239671	0.319005
Ecdysone-vs-Faecalibacterium	0.517887	0.000117	0.000818
Ecdysone-vs-Oscillibacter	0.365282	0.009096	0.025199
Ecdysone-vs-Subdoligranulum	0.249652	0.080385	0.143091
Ecdysone-vs-Bilophila	0.237167	0.097245	0.165516
Ecdysone-vs-Butyricicoccus	0.46958	0.000581	0.002771
Ecdysone-vs-Paraprevotella	0.130276	0.367193	0.441236
Ecdysone-vs-Collinsella	0.339448	0.015881	0.03923
Ecdysone-vs-Parasutterella	0.241777	0.090727	0.155594
Ecdysone-vs-Pyramidobacter	0.130372	0.366837	0.441021

Ecdysone-vs-Intestinimonas	0.150732	0.296086	0.375012
Ecdysone-vs-Pseudoflavonifractor	0.184538	0.199518	0.278228
Ecdysone-vs-Holdemania	0.151693	0.292983	0.372684
Ecdysone-vs-Oribacterium	0.215846	0.132203	0.204307
Ecdysone-vs-Eubacterium	-0.14257	0.323312	0.400514
Ecdysone-vs-Veillonella	-0.62257	1.38E-06	5.93E-05
Ecdysone-vs-Lactobacillus	-0.53172	7.08E-05	0.000581
Ecdysone-vs-Agathobacter	-0.17253	0.23086	0.309767
Ecdysone-vs-Enterococcus	-0.56802	1.69E-05	0.000214
Ecdysone-vs-Blautia	-0.32351	0.021924	0.05135
Ecdysone-vs-Streptococcus	-0.47582	0.000479	0.002388
Ecdysone-vs-Erysipelatoclostridium	-0.49234	0.000281	0.001575
Ecdysone-vs-Lachnospira	-0.10838	0.453746	0.519372
Ecdysone-vs-Fusobacterium	-0.41609	0.002653	0.009351
Ecdysone-vs-Bacillus	-0.43625	0.00154	0.006036
Ecdysone-vs-Dorea	-0.25695	0.071649	0.130153
Ecdysone-vs-Tyzzerella	-0.22468	0.116721	0.18725
Ecdysone-vs-Butyrivibrio	-0.23112	0.106343	0.175007
Ecdysone-vs-Coprobacillus	-0.56812	1.68E-05	0.000214
Ecdysone-vs-Selenomonas	-0.45191	0.000986	0.004175
Ecdysone-vs-Anaerostipes	-0.31534	0.025704	0.057752
Ecdysone-vs-Peptoclostridium	-0.15697	0.276299	0.356307
Ecdysone-vs-Dysgonomonas	-0.52	0.000109	0.00078
Ecdysone-vs-Capnocytophaga	-0.17522	0.223562	0.302423
Ecdysone-vs-Flavobacterium	-0.22468	0.116721	0.18725

3-C-Carboxy-2,4-dideoxy-2-methylpentaric acid-vs-Alistipes	0.211909	0.13958	0.212664
3-C-Carboxy-2,4-dideoxy-2-methylpentaric acid-vs-Faecalibacterium	0.400048	0.003996	0.012963
3-C-Carboxy-2,4-dideoxy-2-methylpentaric acid-vs-Oscillibacter	0.285186	0.0447	0.090529
3-C-Carboxy-2,4-dideoxy-2-methylpentaric acid-vs-Subdoligranulum	0.151116	0.294842	0.374391
3-C-Carboxy-2,4-dideoxy-2-methylpentaric acid-vs-Bilophila	0.373541	0.00754	0.021511
3-C-Carboxy-2,4-dideoxy-2-methylpentaric acid-vs-Butyricicoccus	0.357503	0.010808	0.028973
3-C-Carboxy-2,4-dideoxy-2-methylpentaric acid-vs-Paraprevotella	0.114046	0.430343	0.498323
3-C-Carboxy-2,4-dideoxy-2-methylpentaric acid-vs-Collinsella	0.335606	0.01719	0.041919
3-C-Carboxy-2,4-dideoxy-2-methylpentaric acid-vs-Parasutterella	0.190396	0.185369	0.262773
3-C-Carboxy-2,4-dideoxy-2-methylpentaric acid-vs-Pyramidobacter	0.106363	0.46224	0.527153
3-C-Carboxy-2,4-dideoxy-2-methylpentaric acid-vs-Intestinimonas	0.137191	0.342086	0.41876
3-C-Carboxy-2,4-dideoxy-2-methylpentaric acid-vs-Pseudoflavonifractor	0.055942	0.699593	0.735402

3-C-Carboxy-2,4-dideoxy-2-methylpentaric acid-vs-Holdemania	-0.03261	0.82215	0.844581
3-C-Carboxy-2,4-dideoxy-2-methylpentaric acid-vs-Oribacterium	-0.02319	0.87298	0.890174
3-C-Carboxy-2,4-dideoxy-2-methylpentaric acid-vs-Eubacterium	-0.24418	0.087469	0.151577
3-C-Carboxy-2,4-dideoxy-2-methylpentaric acid-vs-Veillonella	-0.65887	1.97E-07	0.000035
3-C-Carboxy-2,4-dideoxy-2-methylpentaric acid-vs-Lactobacillus	-0.47323	0.000519	0.002549
3-C-Carboxy-2,4-dideoxy-2-methylpentaric acid-vs-Agathobacter	-0.20663	0.149951	0.223935
3-C-Carboxy-2,4-dideoxy-2-methylpentaric acid-vs-Enterococcus	-0.56447	1.96E-05	0.00024
3-C-Carboxy-2,4-dideoxy-2-methylpentaric acid-vs-Blautia	-0.36288	0.009598	0.026295
3-C-Carboxy-2,4-dideoxy-2-methylpentaric acid-vs-Streptococcus	-0.56831	1.67E-05	0.000214
3-C-Carboxy-2,4-dideoxy-2-methylpentaric acid-vs-Erysipelatoclostridium	-0.60999	2.57E-06	7.34E-05
3-C-Carboxy-2,4-dideoxy-2-methylpentaric acid-vs-Lachnospira	-0.18521	0.197856	0.276376
3-C-Carboxy-2,4-dideoxy-2-methylpentaric acid-vs-Fusobacterium	-0.4522	0.000978	0.004147

3-C-Carboxy-2,4-dideoxy-2-methylpentaric acid-vs-Bacillus	-0.46824	0.000605	0.002855
3-C-Carboxy-2,4-dideoxy-2-methylpentaric acid-vs-Dorea	-0.28586	0.044177	0.089763
3-C-Carboxy-2,4-dideoxy-2-methylpentaric acid-vs-Tyzzerella	-0.30487	0.031334	0.068065
3-C-Carboxy-2,4-dideoxy-2-methylpentaric acid-vs-Butyrivibrio	-0.30122	0.033524	0.071878
3-C-Carboxy-2,4-dideoxy-2-methylpentaric acid-vs-Coprobacillus	-0.64178	5.1E-07	4.31E-05
3-C-Carboxy-2,4-dideoxy-2-methylpentaric acid-vs-Selenomonas	-0.41954	0.002422	0.008775
3-C-Carboxy-2,4-dideoxy-2-methylpentaric acid-vs-Anaerostipes	-0.35462	0.011509	0.030329
3-C-Carboxy-2,4-dideoxy-2-methylpentaric acid-vs-Peptoclostridium	-0.23938	0.094078	0.160786
3-C-Carboxy-2,4-dideoxy-2-methylpentaric acid-vs-Dysgonomonas	-0.45969	0.000784	0.003485
3-C-Carboxy-2,4-dideoxy-2-methylpentaric acid-vs-Capnocytophaga	-0.03942	0.785758	0.812571
3-C-Carboxy-2,4-dideoxy-2-methylpentaric acid-vs-Flavobacterium	-0.20144	0.160676	0.236121
(3beta,5alpha,14xi,22R,23R,24S)-3,22,23-Trihydroxyergostan-6-one-vs-Alistipes	0.277311	0.05121	0.09981
(3beta,5alpha,14xi,22R,23R,24S)-3,22,23-Trihydroxyergostan-6-one-vs-Faecalibacterium	0.474382	0.000501	0.002479

(3beta,5alpha,14xi,22R,23R,24S)-3,22,23-Trihydroxyergostan-6-one-vs-Oscillibacter	0.369604	0.00825	0.023166
(3beta,5alpha,14xi,22R,23R,24S)-3,22,23-Trihydroxyergostan-6-one-vs-Subdoligranulum	0.23419	0.101643	0.170664
(3beta,5alpha,14xi,22R,23R,24S)-3,22,23-Trihydroxyergostan-6-one-vs-Bilophila	0.246291	0.084679	0.148189
(3beta,5alpha,14xi,22R,23R,24S)-3,22,23-Trihydroxyergostan-6-one-vs-Butyricicoccus	0.418968	0.00246	0.008871
(3beta,5alpha,14xi,22R,23R,24S)-3,22,23-Trihydroxyergostan-6-one-vs-Paraprevotella	0.208836	0.145547	0.219202
(3beta,5alpha,14xi,22R,23R,24S)-3,22,23-Trihydroxyergostan-6-one-vs-Collinsella	0.38401	0.005903	0.01759
(3beta,5alpha,14xi,22R,23R,24S)-3,22,23-Trihydroxyergostan-6-one-vs-Parasutterella	0.259064	0.069267	0.126751
(3beta,5alpha,14xi,22R,23R,24S)-3,22,23-Trihydroxyergostan-6-one-vs-Pyramidobacter	0.182617	0.204319	0.283175
(3beta,5alpha,14xi,22R,23R,24S)-3,22,23-Trihydroxyergostan-6-one-vs-Intestinimonas	0.107611	0.456971	0.521863
(3beta,5alpha,14xi,22R,23R,24S)-3,22,23-Trihydroxyergostan-6-one-vs-Pseudoflavonifractor	0.151693	0.292983	0.372684
(3beta,5alpha,14xi,22R,23R,24S)-3,22,23-Trihydroxyergostan-6-one-vs-Holdemania	0.041825	0.773047	0.80076
(3beta,5alpha,14xi,22R,23R,24S)-3,22,23-Trihydroxyergostan-6-one-vs-Oribacterium	0.140648	0.329941	0.406496

(3beta,5alpha,14xi,22R,23R,24S)-3,22,23-Trihydroxyergostan-6-one-vs-Eubacterium	-0.30247	0.032761	0.070486
(3beta,5alpha,14xi,22R,23R,24S)-3,22,23-Trihydroxyergostan-6-one-vs-Veillonella	-0.65196	2.92E-07	3.82E-05
(3beta,5alpha,14xi,22R,23R,24S)-3,22,23-Trihydroxyergostan-6-one-vs-Lactobacillus	-0.58848	6.97E-06	0.000121
(3beta,5alpha,14xi,22R,23R,24S)-3,22,23-Trihydroxyergostan-6-one-vs-Agathobacter	-0.28538	0.04455	0.090299
(3beta,5alpha,14xi,22R,23R,24S)-3,22,23-Trihydroxyergostan-6-one-vs-Enterococcus	-0.60192	3.77E-06	0.00009
(3beta,5alpha,14xi,22R,23R,24S)-3,22,23-Trihydroxyergostan-6-one-vs-Blautia	-0.48773	0.000327	0.001753
(3beta,5alpha,14xi,22R,23R,24S)-3,22,23-Trihydroxyergostan-6-one-vs-Streptococcus	-0.59779	4.56E-06	9.45E-05
(3beta,5alpha,14xi,22R,23R,24S)-3,22,23-Trihydroxyergostan-6-one-vs-Erysipelatoclostridium	-0.59914	4.29E-06	9.31E-05
(3beta,5alpha,14xi,22R,23R,24S)-3,22,23-Trihydroxyergostan-6-one-vs-Lachnospira	-0.18079	0.208955	0.288154
(3beta,5alpha,14xi,22R,23R,24S)-3,22,23-Trihydroxyergostan-6-one-vs-Fusobacterium	-0.55073	3.41E-05	0.000347
(3beta,5alpha,14xi,22R,23R,24S)-3,22,23-Trihydroxyergostan-6-one-vs-Bacillus	-0.32187	0.02264	0.052384
(3beta,5alpha,14xi,22R,23R,24S)-3,22,23-Trihydroxyergostan-6-one-vs-Dorea	-0.34291	0.014777	0.037243

(3beta,5alpha,14xi,22R,23R,24S)-3,22,23-Trihydroxyergostan-6-one-vs-Tyzzerella	-0.41253	0.00291	0.010114
(3beta,5alpha,14xi,22R,23R,24S)-3,22,23-Trihydroxyergostan-6-one-vs-Butyrivibrio	-0.29527	0.037367	0.078559
(3beta,5alpha,14xi,22R,23R,24S)-3,22,23-Trihydroxyergostan-6-one-vs-Coprobacillus	-0.66127	1.72E-07	0.000035
(3beta,5alpha,14xi,22R,23R,24S)-3,22,23-Trihydroxyergostan-6-one-vs-Selenomonas	-0.41551	0.002693	0.009453
(3beta,5alpha,14xi,22R,23R,24S)-3,22,23-Trihydroxyergostan-6-one-vs-Anaerostipes	-0.47073	0.000561	0.00269
(3beta,5alpha,14xi,22R,23R,24S)-3,22,23-Trihydroxyergostan-6-one-vs-Peptoclostridium	-0.30324	0.032299	0.069672
(3beta,5alpha,14xi,22R,23R,24S)-3,22,23-Trihydroxyergostan-6-one-vs-Dysgonomonas	-0.40975	0.003127	0.010672
(3beta,5alpha,14xi,22R,23R,24S)-3,22,23-Trihydroxyergostan-6-one-vs-Capnocytophaga	-0.06593	0.64918	0.692365
(3beta,5alpha,14xi,22R,23R,24S)-3,22,23-Trihydroxyergostan-6-one-vs-Flavobacterium	-0.1322	0.36011	0.435038
thr-asp-vs-Alistipes	0.292485	0.039285	0.081694
thr-asp-vs-Faecalibacterium	0.378535	0.006716	0.01961
thr-asp-vs-Oscillibacter	0.465738	0.000653	0.003018
thr-asp-vs-Subdoligranulum	0.221609	0.121941	0.193997
thr-asp-vs-Bilophila	0.271357	0.056626	0.108077
thr-asp-vs-Butyricicoccus	0.503866	0.000191	0.001167
thr-asp-vs-Paraprevotella	0.206819	0.149564	0.223761

thr-asp-vs-Collinsella	0.376807	0.006992	0.020273
thr-asp-vs-Parasutterella	0.255606	0.073199	0.132484
thr-asp-vs-Pyramidobacter	0.281921	0.047312	0.094434
thr-asp-vs-Intestinimonas	0.216999	0.130101	0.202189
thr-asp-vs-Pseudoflavonifractor	0.195198	0.174319	0.250684
thr-asp-vs-Holdemania	0.139592	0.333623	0.410219
thr-asp-vs-Oribacterium	0.125282	0.386	0.458075
thr-asp-vs-Eubacterium	-0.08773	0.544626	0.599378
thr-asp-vs-Veillonella	-0.64581	4.1E-07	4.07E-05
thr-asp-vs-Lactobacillus	-0.45834	0.000816	0.003608
thr-asp-vs-Agathobacter	-0.15486	0.282896	0.361809
thr-asp-vs-Enterococcus	-0.428	0.001932	0.007274
thr-asp-vs-Blautia	-0.35741	0.010831	0.029002
thr-asp-vs-Streptococcus	-0.48149	0.0004	0.002059
thr-asp-vs-Erysipelatoclostridium	-0.457	0.000849	0.003722
thr-asp-vs-Lachnospira	-0.15601	0.279285	0.358298
thr-asp-vs-Fusobacterium	-0.41388	0.00281	0.009808
thr-asp-vs-Bacillus	-0.27904	0.049719	0.097515
thr-asp-vs-Dorea	-0.22708	0.112762	0.182075
thr-asp-vs-Tyzzerella	-0.28499	0.04485	0.090686
thr-asp-vs-Butyrivibrio	-0.19606	0.172381	0.248617
thr-asp-vs-Coprobacillus	-0.51962	0.00011	0.000789
thr-asp-vs-Selenomonas	-0.31726	0.024769	0.056056
thr-asp-vs-Anaerostipes	-0.34838	0.013163	0.033792
thr-asp-vs-Peptoclostridium	-0.19539	0.173887	0.250243

thr-asp-vs-Dysgonomonas	-0.35933	0.010383	0.028169
thr-asp-vs-Capnocytophaga	-0.0449	0.756863	0.785305
thr-asp-vs-Flavobacterium	-0.00639	0.96489	0.969572
Corticosterone-vs-Alistipes	0.18617	0.1955	0.273854
Corticosterone-vs-Faecalibacterium	0.456807	0.000854	0.003736
Corticosterone-vs-Oscillibacter	0.246002	0.085056	0.148742
Corticosterone-vs-Subdoligranulum	0.155534	0.280786	0.359852
Corticosterone-vs-Bilophila	0.335318	0.017291	0.042003
Corticosterone-vs-Butyricicoccus	0.312749	0.027013	0.060095
Corticosterone-vs-Paraprevotella	0.146987	0.308388	0.387042
Corticosterone-vs-Collinsella	0.318896	0.023996	0.054607
Corticosterone-vs-Parasutterella	0.13431	0.352415	0.42845
Corticosterone-vs-Pyramidobacter	0.039808	0.783721	0.811139
Corticosterone-vs-Intestinimonas	0.081873	0.571914	0.625255
Corticosterone-vs-Pseudoflavonifractor	0.06036	0.677121	0.716324
Corticosterone-vs-Holdemania	0.080528	0.578264	0.630534
Corticosterone-vs-Oribacterium	0.028187	0.845932	0.865079
Corticosterone-vs-Eubacterium	-0.29325	0.038748	0.080984
Corticosterone-vs-Veillonella	-0.61037	2.52E-06	7.29E-05
Corticosterone-vs-Lactobacillus	-0.54507	4.26E-05	0.000409
Corticosterone-vs-Agathobacter	-0.3187	0.024086	0.054761
Corticosterone-vs-Enterococcus	-0.65916	1.94E-07	0.000035
Corticosterone-vs-Blautia	-0.39765	0.004241	0.013635
Corticosterone-vs-Streptococcus	-0.59049	6.37E-06	0.000116
Corticosterone-vs-Erysipelatoclostridium	-0.56956	1.58E-05	0.000207

Corticosterone-vs-Lachnospira	-0.08917	0.538014	0.594206
Corticosterone-vs-Fusobacterium	-0.4836	0.000374	0.001957
Corticosterone-vs-Bacillus	-0.42569	0.002056	0.00766
Corticosterone-vs-Dorea	-0.28624	0.043881	0.08938
Corticosterone-vs-Tyzzerella	-0.34492	0.014164	0.035916
Corticosterone-vs-Butyrivibrio	-0.35616	0.011131	0.029551
Corticosterone-vs-Coprobacillus	-0.66646	1.27E-07	0.000035
Corticosterone-vs-Selenomonas	-0.43088	0.001786	0.006828
Corticosterone-vs-Anaerostipes	-0.41148	0.00299	0.010336
Corticosterone-vs-Peptoclostridium	-0.2534	0.075803	0.1365
Corticosterone-vs-Dysgonomonas	-0.42233	0.00225	0.008282
Corticosterone-vs-Capnocytophaga	-0.15102	0.295153	0.374594
Corticosterone-vs-Flavobacterium	-0.19145	0.182896	0.260158
Bufexamac-vs-Alistipes	0.148427	0.303618	0.382796
Bufexamac-vs-Faecalibacterium	0.43078	0.001791	0.006836
Bufexamac-vs-Oscillibacter	0.29527	0.037367	0.078559
Bufexamac-vs-Subdoligranulum	0.171957	0.232445	0.311221
Bufexamac-vs-Bilophila	0.251573	0.078009	0.139763
Bufexamac-vs-Butyricicoccus	0.415798	0.002673	0.009395
Bufexamac-vs-Paraprevotella	0.131717	0.361873	0.436531
Bufexamac-vs-Collinsella	0.224202	0.117525	0.188297
Bufexamac-vs-Parasutterella	0.127587	0.37725	0.450056
Bufexamac-vs-Pyramidobacter	0.113469	0.432691	0.500576
Bufexamac-vs-Intestinimonas	0.178583	0.214665	0.294394
Bufexamac-vs-Pseudoflavonifractor	0.188764	0.189238	0.267039

Bufexamac-vs-Holdemania	-0.01532	0.915914	0.927862
Bufexamac-vs-Oribacterium	0.051717	0.72133	0.754421
Bufexamac-vs-Eubacterium	-0.30737	0.029906	0.065593
Bufexamac-vs-Veillonella	-0.49964	0.000221	0.001299
Bufexamac-vs-Lactobacillus	-0.5176	0.000118	0.000822
Bufexamac-vs-Agathobacter	-0.33215	0.018444	0.044113
Bufexamac-vs-Enterococcus	-0.57676	1.17E-05	0.000168
Bufexamac-vs-Blautia	-0.43606	0.001548	0.006049
Bufexamac-vs-Streptococcus	-0.59789	4.54E-06	9.45E-05
Bufexamac-vs-Erysipelatoclostridium	-0.58511	8.09E-06	0.000132
Bufexamac-vs-Lachnospira	-0.38401	0.005903	0.01759
Bufexamac-vs-Fusobacterium	-0.60077	3.98E-06	9.14E-05
Bufexamac-vs-Bacillus	-0.47986	0.000421	0.002137
Bufexamac-vs-Dorea	-0.40303	0.003709	0.012338
Bufexamac-vs-Tyzzerella	-0.29565	0.037109	0.078281
Bufexamac-vs-Butyrivibrio	-0.38747	0.005436	0.016614
Bufexamac-vs-Coprobacillus	-0.66953	1.06E-07	0.000035
Bufexamac-vs-Selenomonas	-0.43779	0.001475	0.005856
Bufexamac-vs-Anaerostipes	-0.42761	0.001952	0.007316
Bufexamac-vs-Peptoclostridium	-0.3212	0.022941	0.052883
Bufexamac-vs-Dysgonomonas	-0.39016	0.005095	0.015786
Bufexamac-vs-Capnocytophaga	-0.21863	0.127165	0.199247
Bufexamac-vs-Flavobacterium	-0.20615	0.150921	0.225113
2-Amino-3-hydroxyoctadecyl dihydrogen phosphate-vs-Alistipes	0.152557	0.290209	0.369831

2-Amino-3-hydroxyoctadecyl dihydrogen phosphate-vs-Faecalibacterium	0.556206	2.74E-05	0.000295
2-Amino-3-hydroxyoctadecyl dihydrogen phosphate-vs-Oscillibacter	0.351741	0.012249	0.031907
2-Amino-3-hydroxyoctadecyl dihydrogen phosphate-vs-Subdoligranulum	0.269244	0.058655	0.110843
2-Amino-3-hydroxyoctadecyl dihydrogen phosphate-vs-Bilophila	0.221705	0.121775	0.193982
2-Amino-3-hydroxyoctadecyl dihydrogen phosphate-vs-Butyricicoccus	0.356351	0.011084	0.029459
2-Amino-3-hydroxyoctadecyl dihydrogen phosphate-vs-Paraprevotella	0.097911	0.498753	0.560055
2-Amino-3-hydroxyoctadecyl dihydrogen phosphate-vs-Collinsella	0.378535	0.006716	0.01961
2-Amino-3-hydroxyoctadecyl dihydrogen phosphate-vs-Parasutterella	0.286915	0.043365	0.088767
2-Amino-3-hydroxyoctadecyl dihydrogen phosphate-vs-Pyramidobacter	0.127971	0.375804	0.448977
2-Amino-3-hydroxyoctadecyl dihydrogen phosphate-vs-Intestinimonas	0.097527	0.500447	0.56145
2-Amino-3-hydroxyoctadecyl dihydrogen phosphate-vs-Pseudoflavonifractor	0.185978	0.19597	0.274203
2-Amino-3-hydroxyoctadecyl dihydrogen phosphate-vs-Holdemania	0.266555	0.061322	0.115182

2-Amino-3-hydroxyoctadecyl dihydrogen phosphate-vs-Oribacterium	0.234958	0.100494	0.169306
2-Amino-3-hydroxyoctadecyl dihydrogen phosphate-vs-Eubacterium	-0.1297	0.369335	0.44338
2-Amino-3-hydroxyoctadecyl dihydrogen phosphate-vs-Veillonella	-0.64072	5.39E-07	4.32E-05
2-Amino-3-hydroxyoctadecyl dihydrogen phosphate-vs-Lactobacillus	-0.45854	0.000811	0.003594
2-Amino-3-hydroxyoctadecyl dihydrogen phosphate-vs-Agathobacter	-0.12394	0.391159	0.461772
2-Amino-3-hydroxyoctadecyl dihydrogen phosphate-vs-Enterococcus	-0.4909	0.000295	0.001633
2-Amino-3-hydroxyoctadecyl dihydrogen phosphate-vs-Blautia	-0.25695	0.071649	0.130153
2-Amino-3-hydroxyoctadecyl dihydrogen phosphate-vs-Streptococcus	-0.42703	0.001983	0.00742
2-Amino-3-hydroxyoctadecyl dihydrogen phosphate-vs-Erysipelatoclostridium	-0.44816	0.001099	0.004622
2-Amino-3-hydroxyoctadecyl dihydrogen phosphate-vs-Lachnospira	-0.01042	0.942745	0.950394
2-Amino-3-hydroxyoctadecyl dihydrogen phosphate-vs-Fusobacterium	-0.40812	0.00326	0.011097
2-Amino-3-hydroxyoctadecyl dihydrogen phosphate-vs-Bacillus	-0.35654	0.011038	0.029398

2-Amino-3-hydroxyoctadecyl dihydrogen phosphate-vs-Dorea	-0.10463	0.469589	0.533087
2-Amino-3-hydroxyoctadecyl dihydrogen phosphate-vs-Tyzzerella	-0.20605	0.151116	0.225133
2-Amino-3-hydroxyoctadecyl dihydrogen phosphate-vs-Butyrivibrio	-0.1492	0.301094	0.380192
2-Amino-3-hydroxyoctadecyl dihydrogen phosphate-vs-Coprobacillus	-0.51827	0.000116	0.000809
2-Amino-3-hydroxyoctadecyl dihydrogen phosphate-vs-Selenomonas	-0.3745	0.007375	0.021113
2-Amino-3-hydroxyoctadecyl dihydrogen phosphate-vs-Anaerostipes	-0.20788	0.14745	0.221397
2-Amino-3-hydroxyoctadecyl dihydrogen phosphate-vs-Peptoclostridium	-0.05911	0.683444	0.721785
2-Amino-3-hydroxyoctadecyl dihydrogen phosphate-vs-Dysgonomonas	-0.49618	0.000248	0.001425
2-Amino-3-hydroxyoctadecyl dihydrogen phosphate-vs-Capnocytophaga	-0.11549	0.424503	0.493159
2-Amino-3-hydroxyoctadecyl dihydrogen phosphate-vs-Flavobacterium	-0.13181	0.36152	0.436317
ibufenac-vs-Alistipes	0.214022	0.135584	0.208495
ibufenac-vs-Faecalibacterium	0.433373	0.001668	0.006445
ibufenac-vs-Oscillibacter	0.356831	0.010968	0.029245
ibufenac-vs-Subdoligranulum	0.265018	0.062889	0.117592
ibufenac-vs-Bilophila	0.340408	0.015568	0.038725

ibufenac-vs-Butyricicoccus	0.425114	0.002088	0.007768
ibufenac-vs-Paraprevotella	-0.00399	0.978085	0.981244
ibufenac-vs-Collinsella	0.355582	0.011271	0.029861
ibufenac-vs-Parasutterella	0.235822	0.099212	0.167602
ibufenac-vs-Pyramidobacter	0.232653	0.103972	0.172939
ibufenac-vs-Intestinimonas	0.245234	0.086065	0.149876
ibufenac-vs-Pseudoflavonifractor	0.280288	0.048664	0.096129
ibufenac-vs-Holdemania	0.023097	0.873502	0.890341
ibufenac-vs-Oribacterium	0.031645	0.827307	0.848827
ibufenac-vs-Eubacterium	-0.24485	0.086574	0.150656
ibufenac-vs-Veillonella	-0.53978	5.22E-05	0.000467
ibufenac-vs-Lactobacillus	-0.34271	0.014837	0.037317
ibufenac-vs-Agathobacter	-0.23697	0.097524	0.165764
ibufenac-vs-Enterococcus	-0.54852	3.72E-05	0.000371
ibufenac-vs-Blautia	-0.33974	0.015787	0.039112
ibufenac-vs-Streptococcus	-0.5611	2.25E-05	0.00026
ibufenac-vs-Erysipelatoclostridium	-0.46996	0.000574	0.002744
ibufenac-vs-Lachnospira	-0.17714	0.218447	0.29761
ibufenac-vs-Fusobacterium	-0.54286	4.64E-05	0.000432
ibufenac-vs-Bacillus	-0.32965	0.019398	0.046128
ibufenac-vs-Dorea	-0.28605	0.044029	0.089608
ibufenac-vs-Tyzzerella	-0.31304	0.026865	0.059927
ibufenac-vs-Butyrivibrio	-0.25138	0.078244	0.139982
ibufenac-vs-Coprobacillus	-0.62017	1.56E-06	5.97E-05
ibufenac-vs-Selenomonas	-0.36327	0.009516	0.0261

ibufenac-vs-Anaerostipes	-0.32149	0.022811	0.052732
ibufenac-vs-Peptoclostridium	-0.15208	0.291748	0.371601
ibufenac-vs-Dysgonomonas	-0.36836	0.008487	0.023724
ibufenac-vs-Capnocytophaga	-0.19683	0.170673	0.246582
ibufenac-vs-Flavobacterium	-0.17724	0.218193	0.297591
2-Hydroxyoctanoic acid-vs-Alistipes	0.111741	0.43978	0.508067
2-Hydroxyoctanoic acid-vs-Faecalibacterium	0.464682	0.000675	0.003085
2-Hydroxyoctanoic acid-vs-Oscillibacter	0.19952	0.164788	0.240598
2-Hydroxyoctanoic acid-vs-Subdoligranulum	0.156303	0.278387	0.35733
2-Hydroxyoctanoic acid-vs-Bilophila	0.176567	0.219973	0.299361
2-Hydroxyoctanoic acid-vs-Butyricicoccus	0.363938	0.009374	0.025825
2-Hydroxyoctanoic acid-vs-Paraprevotella	0.111261	0.44176	0.509408
2-Hydroxyoctanoic acid-vs-Collinsella	0.324082	0.021676	0.050816
2-Hydroxyoctanoic acid-vs-Parasutterella	0.220744	0.123441	0.195382
2-Hydroxyoctanoic acid-vs-Pyramidobacter	-0.03145	0.82834	0.849535
2-Hydroxyoctanoic acid-vs-Intestinimonas	0.02012	0.889698	0.904994
2-Hydroxyoctanoic acid-vs-Pseudoflavonifractor	0.085522	0.554841	0.609271
2-Hydroxyoctanoic acid-vs-Holdemania	0.090324	0.532752	0.589443
2-Hydroxyoctanoic acid-vs-Oribacterium	0.211909	0.13958	0.212664
2-Hydroxyoctanoic acid-vs-Eubacterium	-0.18531	0.19762	0.276201
2-Hydroxyoctanoic acid-vs-Veillonella	-0.64235	4.94E-07	4.31E-05
2-Hydroxyoctanoic acid-vs-Lactobacillus	-0.57954	1.03E-05	0.000156
2-Hydroxyoctanoic acid-vs-Agathobacter	-0.16389	0.255424	0.335303
2-Hydroxyoctanoic acid-vs-Enterococcus	-0.55381	3.02E-05	0.000319
2-Hydroxyoctanoic acid-vs-Blautia	-0.32495	0.021309	0.050049

2-Hydroxyoctanoic acid-vs-Streptococcus	-0.47419	0.000504	0.002488
2-Hydroxyoctanoic acid-vs-Erysipelatoclostridium	-0.51049	0.000152	0.000997
2-Hydroxyoctanoic acid-vs-Lachnospira	-0.02761	0.849045	0.867904
2-Hydroxyoctanoic acid-vs-Fusobacterium	-0.41176	0.002968	0.010288
2-Hydroxyoctanoic acid-vs-Bacillus	-0.46267	0.000717	0.003234
2-Hydroxyoctanoic acid-vs-Dorea	-0.23669	0.097944	0.166024
2-Hydroxyoctanoic acid-vs-Tyzzerella	-0.22651	0.113702	0.183474
2-Hydroxyoctanoic acid-vs-Butyrivibrio	-0.25926	0.069053	0.126453
2-Hydroxyoctanoic acid-vs-Coprobacillus	-0.57119	1.48E-05	0.000201
2-Hydroxyoctanoic acid-vs-Selenomonas	-0.48216	0.000392	0.002025
2-Hydroxyoctanoic acid-vs-Anaerostipes	-0.27251	0.055543	0.106583
2-Hydroxyoctanoic acid-vs-Peptoclostridium	-0.16908	0.240483	0.319572
2-Hydroxyoctanoic acid-vs-Dysgonomonas	-0.53268	6.83E-05	0.00057
2-Hydroxyoctanoic acid-vs-Capnocytophaga	-0.07371	0.610952	0.65952
2-Hydroxyoctanoic acid-vs-Flavobacterium	-0.21239	0.138664	0.211659
para-Tolyl octanoate-vs-Alistipes	0.203842	0.155643	0.230359
para-Tolyl octanoate-vs-Faecalibacterium	0.338007	0.016362	0.040296
para-Tolyl octanoate-vs-Oscillibacter	0.292389	0.039353	0.081766
para-Tolyl octanoate-vs-Subdoligranulum	0.161297	0.263124	0.343777
para-Tolyl octanoate-vs-Bilophila	0.262041	0.066017	0.122701
para-Tolyl octanoate-vs-Butyricicoccus	0.402545	0.003754	0.012438
para-Tolyl octanoate-vs-Paraprevotella	0.108667	0.452539	0.518948
para-Tolyl octanoate-vs-Collinsella	0.385642	0.005678	0.017083
para-Tolyl octanoate-vs-Parasutterella	0.222665	0.120127	0.191725
para-Tolyl octanoate-vs-Pyramidobacter	0.136327	0.345165	0.421284

para-Tolyl octanoate-vs-Intestinimonas	0.146411	0.310309	0.388864
para-Tolyl octanoate-vs-Pseudoflavonifractor	0.211429	0.1405	0.213673
para-Tolyl octanoate-vs-Holdemania	0.109244	0.450131	0.516425
para-Tolyl octanoate-vs-Oribacterium	0.081393	0.574178	0.627179
para-Tolyl octanoate-vs-Eubacterium	-0.30881	0.029107	0.064123
para-Tolyl octanoate-vs-Veillonella	-0.5514	3.32E-05	0.000344
para-Tolyl octanoate-vs-Lactobacillus	-0.53489	6.29E-05	0.000539
para-Tolyl octanoate-vs-Agathobacter	-0.32437	0.021553	0.050575
para-Tolyl octanoate-vs-Enterococcus	-0.61873	1.68E-06	6.18E-05
para-Tolyl octanoate-vs-Blautia	-0.36615	0.008921	0.02477
para-Tolyl octanoate-vs-Streptococcus	-0.58175	9.38E-06	0.00015
para-Tolyl octanoate-vs-Erysipelatoclostridium	-0.53767	5.66E-05	0.000499
para-Tolyl octanoate-vs-Lachnospira	-0.13297	0.3573	0.432695
para-Tolyl octanoate-vs-Fusobacterium	-0.46094	0.000755	0.003376
para-Tolyl octanoate-vs-Bacillus	-0.32187	0.02264	0.052384
para-Tolyl octanoate-vs-Dorea	-0.27539	0.052909	0.102478
para-Tolyl octanoate-vs-Tyzzerella	-0.29825	0.035402	0.075385
para-Tolyl octanoate-vs-Butyrivibrio	-0.3114	0.027713	0.061434
para-Tolyl octanoate-vs-Coprobacillus	-0.61681	1.84E-06	0.000063
para-Tolyl octanoate-vs-Selenomonas	-0.41868	0.002478	0.0089
para-Tolyl octanoate-vs-Anaerostipes	-0.4036	0.003655	0.012193
para-Tolyl octanoate-vs-Peptoclostridium	-0.26636	0.061516	0.115285
para-Tolyl octanoate-vs-Dysgonomonas	-0.40975	0.003127	0.010672
para-Tolyl octanoate-vs-Capnocytophaga	-0.17522	0.223562	0.302423
para-Tolyl octanoate-vs-Flavobacterium	-0.16984	0.238321	0.317719

DL-Lysine-vs-Alistipes	0.08581	0.553504	0.608609
DL-Lysine-vs-Faecalibacterium	0.532773	6.81E-05	0.000569
DL-Lysine-vs-Oscillibacter	0.230828	0.106792	0.175398
DL-Lysine-vs-Subdoligranulum	0.149484	0.30015	0.379386
DL-Lysine-vs-Bilophila	0.186843	0.193863	0.272021
DL-Lysine-vs-Butyricicoccus	0.485042	0.000357	0.001888
DL-Lysine-vs-Paraprevotella	0.070828	0.624998	0.672346
DL-Lysine-vs-Collinsella	0.298151	0.035464	0.075453
DL-Lysine-vs-Parasutterella	0.180696	0.209201	0.288333
DL-Lysine-vs-Pyramidobacter	0.065738	0.650135	0.693087
DL-Lysine-vs-Intestinimonas	0.064202	0.657802	0.69976
DL-Lysine-vs-Pseudoflavonifractor	0.082545	0.568751	0.622345
DL-Lysine-vs-Holdemania	-0.00293	0.983893	0.985479
DL-Lysine-vs-Oribacterium	0.171188	0.234571	0.313729
DL-Lysine-vs-Eubacterium	-0.12067	0.403855	0.47294
DL-Lysine-vs-Veillonella	-0.58675	7.53E-06	0.000126
DL-Lysine-vs-Lactobacillus	-0.47553	0.000483	0.0024
DL-Lysine-vs-Agathobacter	-0.20173	0.160066	0.235643
DL-Lysine-vs-Enterococcus	-0.50636	0.000176	0.001093
DL-Lysine-vs-Blautia	-0.35827	0.010628	0.028675
DL-Lysine-vs-Streptococcus	-0.53152	7.13E-05	0.000583
DL-Lysine-vs-Erysipelatoclostridium	-0.54612	4.09E-05	0.000397
DL-Lysine-vs-Lachnospira	-0.11539	0.424891	0.493159
DL-Lysine-vs-Fusobacterium	-0.49273	0.000278	0.00157
DL-Lysine-vs-Bacillus	-0.39179	0.004897	0.015268

DL-Lysine-vs-Dorea	-0.26118	0.066947	0.123415
DL-Lysine-vs-Tyzzerella	-0.19645	0.171525	0.24767
DL-Lysine-vs-Butyrivibrio	-0.27866	0.050047	0.098004
DL-Lysine-vs-Coprobacillus	-0.60355	3.49E-06	8.88E-05
DL-Lysine-vs-Selenomonas	-0.35539	0.011319	0.029954
DL-Lysine-vs-Anaerostipes	-0.34223	0.014987	0.03758
DL-Lysine-vs-Peptoclostridium	-0.2195	0.125632	0.197466
DL-Lysine-vs-Dysgonomonas	-0.48091	0.000408	0.002084
DL-Lysine-vs-Capnocytophaga	-0.15121	0.294532	0.374188
DL-Lysine-vs-Flavobacterium	-0.12797	0.375804	0.448977
(2alpha,3alpha,5alpha,22R,23R,24S)-Ergostane-2,3,22,23-tetrol-vs-Alistipes	0.196831	0.170673	0.246582
(2alpha,3alpha,5alpha,22R,23R,24S)-Ergostane-2,3,22,23-tetrol-vs-Faecalibacterium	0.512509	0.000142	0.000952
(2alpha,3alpha,5alpha,22R,23R,24S)-Ergostane-2,3,22,23-tetrol-vs-Oscillibacter	0.294118	0.038151	0.080073
(2alpha,3alpha,5alpha,22R,23R,24S)-Ergostane-2,3,22,23-tetrol-vs-Subdoligranulum	0.228715	0.110129	0.179221
(2alpha,3alpha,5alpha,22R,23R,24S)-Ergostane-2,3,22,23-tetrol-vs-Bilophila	0.230732	0.106942	0.175528
(2alpha,3alpha,5alpha,22R,23R,24S)-Ergostane-2,3,22,23-tetrol-vs-Butyricicoccus	0.381705	0.006234	0.018531
(2alpha,3alpha,5alpha,22R,23R,24S)-Ergostane-2,3,22,23-tetrol-vs-Paraprevotella	0.147275	0.30743	0.386425

(2alpha,3alpha,5alpha,22R,23R,24S)-Ergostane-2,3,22,23-tetrol-vs-Collinsella	0.322257	0.02247	0.052136
(2alpha,3alpha,5alpha,22R,23R,24S)-Ergostane-2,3,22,23-tetrol-vs-Parasutterella	0.213733	0.136124	0.209195
(2alpha,3alpha,5alpha,22R,23R,24S)-Ergostane-2,3,22,23-tetrol-vs-Pyramidobacter	0.079184	0.584646	0.636376
(2alpha,3alpha,5alpha,22R,23R,24S)-Ergostane-2,3,22,23-tetrol-vs-Intestinimonas	0.107899	0.45576	0.521198
(2alpha,3alpha,5alpha,22R,23R,24S)-Ergostane-2,3,22,23-tetrol-vs-Pseudoflavonifractor	0.146507	0.309988	0.388854
(2alpha,3alpha,5alpha,22R,23R,24S)-Ergostane-2,3,22,23-tetrol-vs-Holdemania	0.10108	0.484889	0.546958
(2alpha,3alpha,5alpha,22R,23R,24S)-Ergostane-2,3,22,23-tetrol-vs-Oribacterium	0.119616	0.408013	0.476687
(2alpha,3alpha,5alpha,22R,23R,24S)-Ergostane-2,3,22,23-tetrol-vs-Eubacterium	-0.25417	0.074889	0.135345
(2alpha,3alpha,5alpha,22R,23R,24S)-Ergostane-2,3,22,23-tetrol-vs-Veillonella	-0.58809	7.09E-06	0.000121
(2alpha,3alpha,5alpha,22R,23R,24S)-Ergostane-2,3,22,23-tetrol-vs-Lactobacillus	-0.57772	1.12E-05	0.000163
(2alpha,3alpha,5alpha,22R,23R,24S)-Ergostane-2,3,22,23-tetrol-vs-Agathobacter	-0.28845	0.042207	0.086896
(2alpha,3alpha,5alpha,22R,23R,24S)-Ergostane-2,3,22,23-tetrol-vs-Enterococcus	-0.617	1.83E-06	0.000063

(2alpha,3alpha,5alpha,22R,23R,24S)-Ergostane-2,3,22,23-tetrol-vs-Blautia	-0.37969	0.006537	0.019247
(2alpha,3alpha,5alpha,22R,23R,24S)-Ergostane-2,3,22,23-tetrol-vs-Streptococcus	-0.54103	4.98E-05	0.000452
(2alpha,3alpha,5alpha,22R,23R,24S)-Ergostane-2,3,22,23-tetrol-vs-Erysipelatoclostridium	-0.63102	8.99E-07	5.32E-05
(2alpha,3alpha,5alpha,22R,23R,24S)-Ergostane-2,3,22,23-tetrol-vs-Lachnospira	-0.19059	0.184917	0.262583
(2alpha,3alpha,5alpha,22R,23R,24S)-Ergostane-2,3,22,23-tetrol-vs-Fusobacterium	-0.508	0.000166	0.001046
(2alpha,3alpha,5alpha,22R,23R,24S)-Ergostane-2,3,22,23-tetrol-vs-Bacillus	-0.38833	0.005324	0.016313
(2alpha,3alpha,5alpha,22R,23R,24S)-Ergostane-2,3,22,23-tetrol-vs-Dorea	-0.2849	0.044926	0.090691
(2alpha,3alpha,5alpha,22R,23R,24S)-Ergostane-2,3,22,23-tetrol-vs-Tyzzerella	-0.30363	0.03207	0.069359
(2alpha,3alpha,5alpha,22R,23R,24S)-Ergostane-2,3,22,23-tetrol-vs-Butyrivibrio	-0.31726	0.024769	0.056056
(2alpha,3alpha,5alpha,22R,23R,24S)-Ergostane-2,3,22,23-tetrol-vs-Coprobacillus	-0.62987	9.54E-07	5.32E-05
(2alpha,3alpha,5alpha,22R,23R,24S)-Ergostane-2,3,22,23-tetrol-vs-Selenomonas	-0.44864	0.001084	0.004566
(2alpha,3alpha,5alpha,22R,23R,24S)-Ergostane-2,3,22,23-tetrol-vs-Anaerostipes	-0.40053	0.003948	0.012842

(2alpha,3alpha,5alpha,22R,23R,24S)-Ergostane-2,3,22,23-tetrol-vs-Peptoclostridium	-0.26185	0.066222	0.122725
(2alpha,3alpha,5alpha,22R,23R,24S)-Ergostane-2,3,22,23-tetrol-vs-Dysgonomonas	-0.45306	0.000954	0.004064
(2alpha,3alpha,5alpha,22R,23R,24S)-Ergostane-2,3,22,23-tetrol-vs-Capnocytophaga	-0.13969	0.333287	0.410009
(2alpha,3alpha,5alpha,22R,23R,24S)-Ergostane-2,3,22,23-tetrol-vs-Flavobacterium	-0.23285	0.103679	0.172682
Deoxycholic acid-vs-Alistipes	0.221321	0.122439	0.194541
Deoxycholic acid-vs-Faecalibacterium	0.446435	0.001155	0.0048
Deoxycholic acid-vs-Oscillibacter	0.299592	0.034543	0.07381
Deoxycholic acid-vs-Subdoligranulum	0.296615	0.036469	0.077128
Deoxycholic acid-vs-Bilophila	0.173782	0.227451	0.306517
Deoxycholic acid-vs-Butyricicoccus	0.346267	0.013767	0.035125
Deoxycholic acid-vs-Paraprevotella	0.248788	0.081472	0.144201
Deoxycholic acid-vs-Collinsella	0.420984	0.002332	0.008533
Deoxycholic acid-vs-Parasutterella	0.291044	0.040309	0.083543
Deoxycholic acid-vs-Pyramidobacter	0.246483	0.084429	0.147855
Deoxycholic acid-vs-Intestinimonas	0.16024	0.266305	0.346294
Deoxycholic acid-vs-Pseudoflavonifractor	0.168788	0.241297	0.320312
Deoxycholic acid-vs-Holdemania	0.088595	0.540654	0.596592
Deoxycholic acid-vs-Oribacterium	0.048163	0.73978	0.769185
Deoxycholic acid-vs-Eubacterium	-0.27942	0.049392	0.097105
Deoxycholic acid-vs-Veillonella	-0.62026	1.55E-06	5.97E-05
Deoxycholic acid-vs-Lactobacillus	-0.49282	0.000277	0.001568

Deoxycholic acid-vs-Agathobacter	-0.2607	0.067469	0.124009
Deoxycholic acid-vs-Enterococcus	-0.60653	3.03E-06	0.000081
Deoxycholic acid-vs-Blautia	-0.37911	0.006626	0.019439
Deoxycholic acid-vs-Streptococcus	-0.49628	0.000247	0.001424
Deoxycholic acid-vs-Erysipelatoclostridium	-0.5563	2.73E-05	0.000295
Deoxycholic acid-vs-Lachnospira	-0.17906	0.213414	0.293003
Deoxycholic acid-vs-Fusobacterium	-0.50492	0.000184	0.00114
Deoxycholic acid-vs-Bacillus	-0.38872	0.005275	0.01624
Deoxycholic acid-vs-Dorea	-0.24888	0.081351	0.144089
Deoxycholic acid-vs-Tyzzerella	-0.37709	0.006945	0.020208
Deoxycholic acid-vs-Butyrivibrio	-0.28	0.048906	0.096453
Deoxycholic acid-vs-Coprobacillus	-0.59894	4.33E-06	9.31E-05
Deoxycholic acid-vs-Selenomonas	-0.48946	0.000309	0.001686
Deoxycholic acid-vs-Anaerostipes	-0.38622	0.005601	0.016973
Deoxycholic acid-vs-Peptoclostridium	-0.22372	0.118333	0.189227
Deoxycholic acid-vs-Dysgonomonas	-0.38103	0.006334	0.018782
Deoxycholic acid-vs-Capnocytophaga	-0.07621	0.59889	0.649887
Deoxycholic acid-vs-Flavobacterium	-0.26214	0.065914	0.122602
Dehydroepiandrosterone sulfate-vs-Alistipes	0.218343	0.12768	0.199693
Dehydroepiandrosterone sulfate-vs-Faecalibacterium	0.438079	0.001463	0.005819
Dehydroepiandrosterone sulfate-vs-Oscillibacter	0.35443	0.011557	0.030424
Dehydroepiandrosterone sulfate-vs-Subdoligranulum	0.143721	0.319376	0.396824
Dehydroepiandrosterone sulfate-vs-Bilophila	0.241873	0.090595	0.155475
Dehydroepiandrosterone sulfate-vs-Butyricicoccus	0.413878	0.00281	0.009808
Dehydroepiandrosterone sulfate-vs-Paraprevotella	0.128259	0.374721	0.448114

Dehydroepiandrosterone sulfate-vs-Collinsella	0.386315	0.005588	0.016954
Dehydroepiandrosterone sulfate-vs-Parasutterella	0.249652	0.080385	0.143091
Dehydroepiandrosterone sulfate-vs-Pyramidobacter	0.069676	0.630654	0.676966
Dehydroepiandrosterone sulfate-vs-Intestinimonas	0.100792	0.486141	0.548122
Dehydroepiandrosterone sulfate-vs-Pseudoflavonifractor	0.123361	0.393382	0.463515
Dehydroepiandrosterone sulfate-vs-Holdemania	0.063721	0.660205	0.701115
Dehydroepiandrosterone sulfate-vs-Oribacterium	0.229004	0.109669	0.178707
Dehydroepiandrosterone sulfate-vs-Eubacterium	-0.20375	0.155842	0.230379
Dehydroepiandrosterone sulfate-vs-Veillonella	-0.60259	3.65E-06	8.88E-05
Dehydroepiandrosterone sulfate-vs-Lactobacillus	-0.59241	5.84E-06	0.000111
Dehydroepiandrosterone sulfate-vs-Agathobacter	-0.15342	0.287452	0.366882
Dehydroepiandrosterone sulfate-vs-Enterococcus	-0.52643	8.61E-05	0.000675
Dehydroepiandrosterone sulfate-vs-Blautia	-0.47102	0.000556	0.002671
Dehydroepiandrosterone sulfate-vs-Streptococcus	-0.56533	1.89E-05	0.000232
Dehydroepiandrosterone sulfate-vs-Erysipelatoclostridium	-0.49791	0.000234	0.001364
Dehydroepiandrosterone sulfate-vs-Lachnospira	-0.01224	0.93274	0.941836
Dehydroepiandrosterone sulfate-vs-Fusobacterium	-0.48706	0.000335	0.001788
Dehydroepiandrosterone sulfate-vs-Bacillus	-0.33321	0.018053	0.043428
Dehydroepiandrosterone sulfate-vs-Dorea	-0.30737	0.029906	0.065593
Dehydroepiandrosterone sulfate-vs-Tyzzerella	-0.34752	0.013408	0.034278
Dehydroepiandrosterone sulfate-vs-Butyrivibrio	-0.19971	0.164374	0.240417
Dehydroepiandrosterone sulfate-vs-Coprobacillus	-0.57138	1.47E-05	0.0002
Dehydroepiandrosterone sulfate-vs-Selenomonas	-0.37066	0.008054	0.022718
Dehydroepiandrosterone sulfate-vs-Anaerostipes	-0.36615	0.008921	0.02477
Dehydroepiandrosterone sulfate-vs-Peptoclostridium	-0.2243	0.117364	0.188161

Dehydroepiandrosterone sulfate-vs-Dysgonomonas	-0.50588	0.000178	0.001109
Dehydroepiandrosterone sulfate-vs-Capnocytophaga	0.000624	0.996567	0.996968
Dehydroepiandrosterone sulfate-vs-Flavobacterium	-0.04394	0.76191	0.789881
Ricinoleic Acid-vs-Alistipes	0.126723	0.380517	0.453518
Ricinoleic Acid-vs-Faecalibacterium	0.442593	0.001289	0.005277
Ricinoleic Acid-vs-Oscillibacter	0.231885	0.105152	0.17397
Ricinoleic Acid-vs-Subdoligranulum	0.123938	0.391159	0.461772
Ricinoleic Acid-vs-Bilophila	0.280192	0.048745	0.096211
Ricinoleic Acid-vs-Butyricicoccus	0.455078	0.000899	0.003902
Ricinoleic Acid-vs-Paraprevotella	0.141705	0.326285	0.403191
Ricinoleic Acid-vs-Collinsella	0.336567	0.016854	0.041182
Ricinoleic Acid-vs-Parasutterella	0.161777	0.261687	0.342079
Ricinoleic Acid-vs-Pyramidobacter	0.065642	0.650614	0.6933
Ricinoleic Acid-vs-Intestinimonas	0.125762	0.384167	0.456554
Ricinoleic Acid-vs-Pseudoflavonifractor	0.11904	0.410291	0.478449
Ricinoleic Acid-vs-Holdemania	0.063721	0.660205	0.701115
Ricinoleic Acid-vs-Oribacterium	0.146411	0.310309	0.388864
Ricinoleic Acid-vs-Eubacterium	-0.34242	0.014927	0.037505
Ricinoleic Acid-vs-Veillonella	-0.58098	9.71E-06	0.00015
Ricinoleic Acid-vs-Lactobacillus	-0.63784	6.29E-07	0.000046
Ricinoleic Acid-vs-Agathobacter	-0.43654	0.001528	0.006006
Ricinoleic Acid-vs-Enterococcus	-0.64139	5.2E-07	4.31E-05
Ricinoleic Acid-vs-Blautia	-0.35683	0.010968	0.029245
Ricinoleic Acid-vs-Streptococcus	-0.58867	6.91E-06	0.000121
Ricinoleic Acid-vs-Erysipelatoclostridium	-0.54785	3.82E-05	0.000378

Ricinoleic Acid-vs-Lachnospira	-0.23678	0.097804	0.166012
Ricinoleic Acid-vs-Fusobacterium	-0.47448	0.000499	0.002476
Ricinoleic Acid-vs-Bacillus	-0.41954	0.002422	0.008775
Ricinoleic Acid-vs-Dorea	-0.35577	0.011224	0.029768
Ricinoleic Acid-vs-Tyzzerella	-0.26723	0.060647	0.114172
Ricinoleic Acid-vs-Butyrivibrio	-0.43923	0.001417	0.005679
Ricinoleic Acid-vs-Coprobacillus	-0.62305	1.35E-06	5.93E-05
Ricinoleic Acid-vs-Selenomonas	-0.51587	0.000126	0.000867
Ricinoleic Acid-vs-Anaerostipes	-0.44154	0.001328	0.005401
Ricinoleic Acid-vs-Peptoclostridium	-0.28106	0.048024	0.095091
Ricinoleic Acid-vs-Dysgonomonas	-0.42118	0.00232	0.008502
Ricinoleic Acid-vs-Capnocytophaga	-0.20106	0.161493	0.23704
Ricinoleic Acid-vs-Flavobacterium	-0.2896	0.041355	0.085354
Benzoic acid-vs-Alistipes	0.096375	0.505547	0.566149
Benzoic acid-vs-Faecalibacterium	0.364034	0.009354	0.025799
Benzoic acid-vs-Oscillibacter	0.231309	0.106044	0.174748
Benzoic acid-vs-Subdoligranulum	0.069484	0.631599	0.677687
Benzoic acid-vs-Bilophila	0.271645	0.056354	0.107723
Benzoic acid-vs-Butyricicoccus	0.391501	0.004931	0.015337
Benzoic acid-vs-Paraprevotella	0.017911	0.901745	0.916123
Benzoic acid-vs-Collinsella	0.215846	0.132203	0.204307
Benzoic acid-vs-Parasutterella	0.261753	0.066326	0.122725
Benzoic acid-vs-Pyramidobacter	0.149292	0.300779	0.379988
Benzoic acid-vs-Intestinimonas	0.055846	0.700085	0.735607
Benzoic acid-vs-Pseudoflavonifractor	0.068715	0.635383	0.680866

Benzoic acid-vs-Holdemania	-0.05863	0.685882	0.723437
Benzoic acid-vs-Oribacterium	-0.00351	0.980725	0.983099
Benzoic acid-vs-Eubacterium	-0.21738	0.129406	0.201487
Benzoic acid-vs-Veillonella	-0.67424	7.97E-08	0.000035
Benzoic acid-vs-Lactobacillus	-0.43414	0.001633	0.00633
Benzoic acid-vs-Agathobacter	-0.22948	0.108906	0.178164
Benzoic acid-vs-Enterococcus	-0.52576	8.82E-05	0.000683
Benzoic acid-vs-Blautia	-0.25378	0.075345	0.135774
Benzoic acid-vs-Streptococcus	-0.52413	9.36E-05	0.000707
Benzoic acid-vs-Erysipelatoclostridium	-0.50752	0.000169	0.001061
Benzoic acid-vs-Lachnospira	-0.11462	0.428001	0.496306
Benzoic acid-vs-Fusobacterium	-0.41023	0.003088	0.0106
Benzoic acid-vs-Bacillus	-0.39496	0.004532	0.014256
Benzoic acid-vs-Dorea	-0.25666	0.071979	0.130656
Benzoic acid-vs-Tyzzerella	-0.17685	0.219209	0.298485
Benzoic acid-vs-Butyrivibrio	-0.3552	0.011366	0.030047
Benzoic acid-vs-Coprobacillus	-0.59433	5.35E-06	0.000103
Benzoic acid-vs-Selenomonas	-0.44067	0.001361	0.005498
Benzoic acid-vs-Anaerostipes	-0.3189	0.023996	0.054607
Benzoic acid-vs-Peptoclostridium	-0.20912	0.144979	0.218745
Benzoic acid-vs-Dysgonomonas	-0.48888	0.000315	0.001707
Benzoic acid-vs-Capnocytophaga	-0.11625	0.421407	0.490031
Benzoic acid-vs-Flavobacterium	-0.27212	0.055902	0.107189
(1S,3R,5Z,7E,24R)-9,10-Secocholesta-5,7,10-triene-1,3,24-triol hydrate (1:1)-vs-Alistipes	0.252341	0.077074	0.138488

(1S,3R,5Z,7E,24R)-9,10-Secocholesta-5,7,10-triene-1,3,24-triol hydrate (1:1)-vs-Faecalibacterium	0.462185	0.000727	0.003263
(1S,3R,5Z,7E,24R)-9,10-Secocholesta-5,7,10-triene-1,3,24-triol hydrate (1:1)-vs-Oscillibacter	0.293637	0.038482	0.080631
(1S,3R,5Z,7E,24R)-9,10-Secocholesta-5,7,10-triene-1,3,24-triol hydrate (1:1)-vs-Subdoligranulum	0.281152	0.047944	0.09501
(1S,3R,5Z,7E,24R)-9,10-Secocholesta-5,7,10-triene-1,3,24-triol hydrate (1:1)-vs-Bilophila	0.16072	0.264856	0.344951
(1S,3R,5Z,7E,24R)-9,10-Secocholesta-5,7,10-triene-1,3,24-triol hydrate (1:1)-vs-Butyricicoccus	0.333205	0.018053	0.043428
(1S,3R,5Z,7E,24R)-9,10-Secocholesta-5,7,10-triene-1,3,24-triol hydrate (1:1)-vs-Paraprevotella	0.281825	0.047391	0.09444
(1S,3R,5Z,7E,24R)-9,10-Secocholesta-5,7,10-triene-1,3,24-triol hydrate (1:1)-vs-Collinsella	0.418872	0.002466	0.00888
(1S,3R,5Z,7E,24R)-9,10-Secocholesta-5,7,10-triene-1,3,24-triol hydrate (1:1)-vs-Parasutterella	0.227851	0.111517	0.180651
(1S,3R,5Z,7E,24R)-9,10-Secocholesta-5,7,10-triene-1,3,24-triol hydrate (1:1)-vs-Pyramidobacter	0.143818	0.319049	0.396815
(1S,3R,5Z,7E,24R)-9,10-Secocholesta-5,7,10-triene-1,3,24-triol hydrate (1:1)-vs-Intestinimonas	0.121056	0.402349	0.471399
(1S,3R,5Z,7E,24R)-9,10-Secocholesta-5,7,10-triene-1,3,24-triol hydrate (1:1)-vs-Pseudoflavonifractor	0.144682	0.316118	0.394553
(1S,3R,5Z,7E,24R)-9,10-Secocholesta-5,7,10-triene-1,3,24-triol hydrate (1:1)-vs-Holdemania	0.133541	0.355202	0.430784

(1S,3R,5Z,7E,24R)-9,10-Secocholesta-5,7,10-triene-1,3,24-triol hydrate (1:1)-vs-Oribacterium	0.099832	0.490326	0.552089
(1S,3R,5Z,7E,24R)-9,10-Secocholesta-5,7,10-triene-1,3,24-triol hydrate (1:1)-vs-Eubacterium	-0.20394	0.155444	0.230202
(1S,3R,5Z,7E,24R)-9,10-Secocholesta-5,7,10-triene-1,3,24-triol hydrate (1:1)-vs-Veillonella	-0.59827	4.46E-06	9.45E-05
(1S,3R,5Z,7E,24R)-9,10-Secocholesta-5,7,10-triene-1,3,24-triol hydrate (1:1)-vs-Lactobacillus	-0.54631	4.06E-05	0.000396
(1S,3R,5Z,7E,24R)-9,10-Secocholesta-5,7,10-triene-1,3,24-triol hydrate (1:1)-vs-Agathobacter	-0.18367	0.201668	0.280126
(1S,3R,5Z,7E,24R)-9,10-Secocholesta-5,7,10-triene-1,3,24-triol hydrate (1:1)-vs-Enterococcus	-0.53076	7.34E-05	0.000594
(1S,3R,5Z,7E,24R)-9,10-Secocholesta-5,7,10-triene-1,3,24-triol hydrate (1:1)-vs-Blautia	-0.3914	0.004943	0.015353
(1S,3R,5Z,7E,24R)-9,10-Secocholesta-5,7,10-triene-1,3,24-triol hydrate (1:1)-vs-Streptococcus	-0.47371	0.000511	0.002516
(1S,3R,5Z,7E,24R)-9,10-Secocholesta-5,7,10-triene-1,3,24-triol hydrate (1:1)-vs-Erysipelatoclostridium	-0.60067	3.99E-06	9.14E-05
(1S,3R,5Z,7E,24R)-9,10-Secocholesta-5,7,10-triene-1,3,24-triol hydrate (1:1)-vs-Lachnospira	-0.17714	0.218447	0.29761
(1S,3R,5Z,7E,24R)-9,10-Secocholesta-5,7,10-triene-1,3,24-triol hydrate (1:1)-vs-Fusobacterium	-0.49263	0.000279	0.001571
(1S,3R,5Z,7E,24R)-9,10-Secocholesta-5,7,10-triene-1,3,24-triol hydrate (1:1)-vs-Bacillus	-0.41052	0.003065	0.010536

(1S,3R,5Z,7E,24R)-9,10-Secocholesta-5,7,10-triene-1,3,24-triol hydrate (1:1)-vs-Dorea	-0.21748	0.129232	0.201343
(1S,3R,5Z,7E,24R)-9,10-Secocholesta-5,7,10-triene-1,3,24-triol hydrate (1:1)-vs-Tyzzerella	-0.27155	0.056445	0.107813
(1S,3R,5Z,7E,24R)-9,10-Secocholesta-5,7,10-triene-1,3,24-triol hydrate (1:1)-vs-Butyrivibrio	-0.21882	0.126823	0.198836
(1S,3R,5Z,7E,24R)-9,10-Secocholesta-5,7,10-triene-1,3,24-triol hydrate (1:1)-vs-Coprobacillus	-0.57407	1.31E-05	0.000183
(1S,3R,5Z,7E,24R)-9,10-Secocholesta-5,7,10-triene-1,3,24-triol hydrate (1:1)-vs-Selenomonas	-0.5102	0.000154	0.000997
(1S,3R,5Z,7E,24R)-9,10-Secocholesta-5,7,10-triene-1,3,24-triol hydrate (1:1)-vs-Anaerostipes	-0.3576	0.010785	0.028973
(1S,3R,5Z,7E,24R)-9,10-Secocholesta-5,7,10-triene-1,3,24-triol hydrate (1:1)-vs-Peptoclostridium	-0.20663	0.149951	0.223935
(1S,3R,5Z,7E,24R)-9,10-Secocholesta-5,7,10-triene-1,3,24-triol hydrate (1:1)-vs-Dysgonomonas	-0.44788	0.001108	0.004645
(1S,3R,5Z,7E,24R)-9,10-Secocholesta-5,7,10-triene-1,3,24-triol hydrate (1:1)-vs-Capnocytophaga	-0.08139	0.574178	0.627179
(1S,3R,5Z,7E,24R)-9,10-Secocholesta-5,7,10-triene-1,3,24-triol hydrate (1:1)-vs-Flavobacterium	-0.18665	0.194329	0.272522
13S-hydroxyoctadecadienoic acid-vs-Alistipes	0.238223	0.09572	0.163368
13S-hydroxyoctadecadienoic acid-vs-Faecalibacterium	0.480528	0.000413	0.002105
13S-hydroxyoctadecadienoic acid-vs-Oscillibacter	0.367107	0.00873	0.024321
13S-hydroxyoctadecadienoic acid-vs-Subdoligranulum	0.262425	0.065606	0.122121
13S-hydroxyoctadecadienoic acid-vs-Bilophila	0.283842	0.045761	0.092003

13S-hydroxyoctadecadienoic acid-vs-Butyricicoccus	0.40389	0.003629	0.012137
13S-hydroxyoctadecadienoic acid-vs-Paraprevotella	0.257335	0.071211	0.129546
13S-hydroxyoctadecadienoic acid-vs-Collinsella	0.361921	0.009805	0.026804
13S-hydroxyoctadecadienoic acid-vs-Parasutterella	0.095894	0.50768	0.56777
13S-hydroxyoctadecadienoic acid-vs-Pyramidobacter	0.080912	0.576446	0.629104
13S-hydroxyoctadecadienoic acid-vs-Intestinimonas	0.106074	0.46346	0.528302
13S-hydroxyoctadecadienoic acid-vs-Pseudoflavonifractor	0.156879	0.276597	0.356321
13S-hydroxyoctadecadienoic acid-vs-Holdemania	0.173974	0.22693	0.30598
13S-hydroxyoctadecadienoic acid-vs-Oribacterium	0.214694	0.134331	0.206824
13S-hydroxyoctadecadienoic acid-vs-Eubacterium	-0.27462	0.053602	0.103658
13S-hydroxyoctadecadienoic acid-vs-Veillonella	-0.60077	3.98E-06	9.14E-05
13S-hydroxyoctadecadienoic acid-vs-Lactobacillus	-0.5903	6.42E-06	0.000116
13S-hydroxyoctadecadienoic acid-vs-Agathobacter	-0.27357	0.054566	0.105031
13S-hydroxyoctadecadienoic acid-vs-Enterococcus	-0.57474	1.27E-05	0.000179
13S-hydroxyoctadecadienoic acid-vs-Blautia	-0.37911	0.006626	0.019439
13S-hydroxyoctadecadienoic acid-vs-Streptococcus	-0.53892	0.000054	0.000481
13S-hydroxyoctadecadienoic acid-vs-Erysipelatoclostridium	-0.54228	4.75E-05	0.000437
13S-hydroxyoctadecadienoic acid-vs-Lachnospira	-0.05412	0.708952	0.743039
13S-hydroxyoctadecadienoic acid-vs-Fusobacterium	-0.46852	0.0006	0.002835
13S-hydroxyoctadecadienoic acid-vs-Bacillus	-0.3745	0.007375	0.021113
13S-hydroxyoctadecadienoic acid-vs-Dorea	-0.24581	0.085307	0.148973
13S-hydroxyoctadecadienoic acid-vs-Tyzzerella	-0.31976	0.023596	0.053994
13S-hydroxyoctadecadienoic acid-vs-Butyrivibrio	-0.26934	0.058562	0.110843
13S-hydroxyoctadecadienoic acid-vs-Coprobacillus	-0.62439	1.26E-06	5.93E-05

13S-hydroxyoctadecadienoic acid-vs-Selenomonas	-0.48965	0.000307	0.001686
13S-hydroxyoctadecadienoic acid-vs-Anaerostipes	-0.39765	0.004241	0.013635
13S-hydroxyoctadecadienoic acid-vs-Peptoclostridium	-0.23256	0.104119	0.172952
13S-hydroxyoctadecadienoic acid-vs-Dysgonomonas	-0.47553	0.000483	0.0024
13S-hydroxyoctadecadienoic acid-vs-Capnocytophaga	-0.16082	0.264567	0.344936
13S-hydroxyoctadecadienoic acid-vs-Flavobacterium	-0.12173	0.399721	0.468984
(10E,12Z)-9-Hydroperoxy-10,12-octadecadienoic acid-vs-Alistipes	-0.12221	0.397851	0.467009
(10E,12Z)-9-Hydroperoxy-10,12-octadecadienoic acid-vs-Faecalibacterium	-0.51433	0.000133	0.0009
(10E,12Z)-9-Hydroperoxy-10,12-octadecadienoic acid-vs-Oscillibacter	-0.18118	0.207973	0.287278
(10E,12Z)-9-Hydroperoxy-10,12-octadecadienoic acid-vs-Subdoligranulum	-0.09186	0.525775	0.583803
(10E,12Z)-9-Hydroperoxy-10,12-octadecadienoic acid-vs-Bilophila	-0.19846	0.167082	0.242948
(10E,12Z)-9-Hydroperoxy-10,12-octadecadienoic acid-vs-Butyricicoccus	-0.36816	0.008524	0.023801
(10E,12Z)-9-Hydroperoxy-10,12-octadecadienoic acid-vs-Paraprevotella	-0.12922	0.371125	0.44467
(10E,12Z)-9-Hydroperoxy-10,12-octadecadienoic acid-vs-Collinsella	-0.34896	0.013003	0.033518
(10E,12Z)-9-Hydroperoxy-10,12-octadecadienoic acid-vs-Parasutterella	-0.24715	0.083558	0.146847

(10E,12Z)-9-Hydroperoxy-10,12-octadecadienoic acid-vs-Pyramidobacter	0.0109	0.940111	0.948124
(10E,12Z)-9-Hydroperoxy-10,12-octadecadienoic acid-vs-Intestinimonas	0.018776	0.897028	0.911704
(10E,12Z)-9-Hydroperoxy-10,12-octadecadienoic acid-vs-Pseudoflavonifractor	-0.01676	0.908039	0.921387
(10E,12Z)-9-Hydroperoxy-10,12-octadecadienoic acid-vs-Holdemania	-0.05921	0.682957	0.721577
(10E,12Z)-9-Hydroperoxy-10,12-octadecadienoic acid-vs-Oribacterium	-0.16917	0.240212	0.319383
(10E,12Z)-9-Hydroperoxy-10,12-octadecadienoic acid-vs-Eubacterium	0.223818	0.118171	0.189089
(10E,12Z)-9-Hydroperoxy-10,12-octadecadienoic acid-vs-Veillonella	0.622665	1.38E-06	5.93E-05
(10E,12Z)-9-Hydroperoxy-10,12-octadecadienoic acid-vs-Lactobacillus	0.549292	3.61E-05	0.000365
(10E,12Z)-9-Hydroperoxy-10,12-octadecadienoic acid-vs-Agathobacter	0.172053	0.232181	0.311035
(10E,12Z)-9-Hydroperoxy-10,12-octadecadienoic acid-vs-Enterococcus	0.566675	1.79E-05	0.000222
(10E,12Z)-9-Hydroperoxy-10,12-octadecadienoic acid-vs-Blautia	0.322065	0.022555	0.052285
(10E,12Z)-9-Hydroperoxy-10,12-octadecadienoic acid-vs-Streptococcus	0.518271	0.000116	0.000809

(10E,12Z)-9-Hydroperoxy-10,12-octadecadienoic acid-vs-Erysipelatoclostridium	0.558703	2.48E-05	0.000277
(10E,12Z)-9-Hydroperoxy-10,12-octadecadienoic acid-vs-Lachnospira	0.060744	0.675179	0.714879
(10E,12Z)-9-Hydroperoxy-10,12-octadecadienoic acid-vs-Fusobacterium	0.518271	0.000116	0.000809
(10E,12Z)-9-Hydroperoxy-10,12-octadecadienoic acid-vs-Bacillus	0.454982	0.000901	0.003902
(10E,12Z)-9-Hydroperoxy-10,12-octadecadienoic acid-vs-Dorea	0.221128	0.122773	0.194697
(10E,12Z)-9-Hydroperoxy-10,12-octadecadienoic acid-vs-Tyzzerella	0.223241	0.119146	0.190282
(10E,12Z)-9-Hydroperoxy-10,12-octadecadienoic acid-vs-Butyrivibrio	0.295846	0.03698	0.078076
(10E,12Z)-9-Hydroperoxy-10,12-octadecadienoic acid-vs-Coprobacillus	0.590108	6.48E-06	0.000117
(10E,12Z)-9-Hydroperoxy-10,12-octadecadienoic acid-vs-Selenomonas	0.409268	0.003165	0.01079
(10E,12Z)-9-Hydroperoxy-10,12-octadecadienoic acid-vs-Anaerostipes	0.286339	0.043807	0.08938
(10E,12Z)-9-Hydroperoxy-10,12-octadecadienoic acid-vs-Peptoclostridium	0.216327	0.131324	0.203581
(10E,12Z)-9-Hydroperoxy-10,12-octadecadienoic acid-vs-Dysgonomonas	0.531717	7.08E-05	0.000581

(10E,12Z)-9-Hydroperoxy-10,12-octadecadienoic acid-vs-Capnocytophaga	0.048643	0.737278	0.767226
(10E,12Z)-9-Hydroperoxy-10,12-octadecadienoic acid-vs-Flavobacterium	0.157839	0.27363	0.353598
DL-Tryptophan-vs-Alistipes	-0.18752	0.192235	0.270807
DL-Tryptophan-vs-Faecalibacterium	-0.51433	0.000133	0.0009
DL-Tryptophan-vs-Oscillibacter	-0.30478	0.03139	0.068127
DL-Tryptophan-vs-Subdoligranulum	-0.2486	0.081715	0.144323
DL-Tryptophan-vs-Bilophila	-0.16677	0.247048	0.326898
DL-Tryptophan-vs-Butyricicoccus	-0.43645	0.001532	0.006013
DL-Tryptophan-vs-Paraprevotella	-0.03866	0.789838	0.816111
DL-Tryptophan-vs-Collinsella	-0.39525	0.0045	0.014191
DL-Tryptophan-vs-Parasutterella	-0.33993	0.015724	0.039035
DL-Tryptophan-vs-Pyramidobacter	-0.10233	0.479484	0.542338
DL-Tryptophan-vs-Intestinimonas	-0.0958	0.508107	0.567992
DL-Tryptophan-vs-Pseudoflavonifractor	-0.19088	0.184242	0.261773
DL-Tryptophan-vs-Holdemania	-0.15505	0.282292	0.361223
DL-Tryptophan-vs-Oribacterium	-0.26646	0.061419	0.11519
DL-Tryptophan-vs-Eubacterium	0.05018	0.72929	0.761464
DL-Tryptophan-vs-Veillonella	0.633229	8.02E-07	5.11E-05
DL-Tryptophan-vs-Lactobacillus	0.464778	0.000673	0.003084
DL-Tryptophan-vs-Agathobacter	0.0303	0.83454	0.854483
DL-Tryptophan-vs-Enterococcus	0.4497	0.001051	0.004444
DL-Tryptophan-vs-Blautia	0.234382	0.101355	0.170329
DL-Tryptophan-vs-Streptococcus	0.435294	0.001581	0.00616

DL-Tryptophan-vs-Erysipelatoclostridium	0.417815	0.002535	0.009013
DL-Tryptophan-vs-Lachnospira	-0.07842	0.588307	0.6398
DL-Tryptophan-vs-Fusobacterium	0.38497	0.00577	0.017317
DL-Tryptophan-vs-Bacillus	0.347995	0.013271	0.034035
DL-Tryptophan-vs-Dorea	0.098487	0.496217	0.557711
DL-Tryptophan-vs-Tyzzerella	0.156687	0.277192	0.356718
DL-Tryptophan-vs-Butyrivibrio	0.135942	0.346539	0.422753
DL-Tryptophan-vs-Coprobacillus	0.497527	0.000237	0.001379
DL-Tryptophan-vs-Selenomonas	0.402641	0.003745	0.012424
DL-Tryptophan-vs-Anaerostipes	0.138247	0.338346	0.415
DL-Tryptophan-vs-Peptoclostridium	0.017335	0.904891	0.918943
DL-Tryptophan-vs-Dysgonomonas	0.505306	0.000182	0.001128
DL-Tryptophan-vs-Capnocytophaga	0.091477	0.527515	0.584951
DL-Tryptophan-vs-Flavobacterium	0.130948	0.364705	0.438882
9,10-Dihydroxyoctadecanoic acid-vs-Alistipes	-0.14555	0.313205	0.391309
9,10-Dihydroxyoctadecanoic acid-vs-Faecalibacterium	-0.51136	0.000148	0.000983
9,10-Dihydroxyoctadecanoic acid-vs-Oscillibacter	-0.25167	0.077891	0.139653
9,10-Dihydroxyoctadecanoic acid-vs-Subdoligranulum	-0.23438	0.101355	0.170329
9,10-Dihydroxyoctadecanoic acid-vs-Bilophila	-0.18319	0.20287	0.281481
9,10-Dihydroxyoctadecanoic acid-vs-Butyricicoccus	-0.38507	0.005757	0.017298
9,10-Dihydroxyoctadecanoic acid-vs-Paraprevotella	-0.09474	0.512818	0.572485
9,10-Dihydroxyoctadecanoic acid-vs-Collinsella	-0.38862	0.005287	0.01624
9,10-Dihydroxyoctadecanoic acid-vs-Parasutterella	-0.32101	0.023027	0.052984
9,10-Dihydroxyoctadecanoic acid-vs-Pyramidobacter	-0.05575	0.700576	0.735812
9,10-Dihydroxyoctadecanoic acid-vs-Intestinimonas	-0.06267	0.665504	0.706139

9,10-Dihydroxyoctadecanoic acid-vs-Pseudoflavonifractor	-0.13335	0.3559	0.431421
9,10-Dihydroxyoctadecanoic acid-vs-Holdemania	-0.17849	0.214916	0.294413
9,10-Dihydroxyoctadecanoic acid-vs-Oribacterium	-0.2727	0.055365	0.106404
9,10-Dihydroxyoctadecanoic acid-vs-Eubacterium	0.101561	0.482806	0.545104
9,10-Dihydroxyoctadecanoic acid-vs-Veillonella	0.661657	1.68E-07	0.000035
9,10-Dihydroxyoctadecanoic acid-vs-Lactobacillus	0.489364	0.00031	0.001688
9,10-Dihydroxyoctadecanoic acid-vs-Agathobacter	0.055078	0.704021	0.738806
9,10-Dihydroxyoctadecanoic acid-vs-Enterococcus	0.504634	0.000186	0.001147
9,10-Dihydroxyoctadecanoic acid-vs-Blautia	0.258391	0.070018	0.127937
9,10-Dihydroxyoctadecanoic acid-vs-Streptococcus	0.466122	0.000646	0.002994
9,10-Dihydroxyoctadecanoic acid-vs-Erysipelatoclostridium	0.443169	0.001268	0.0052
9,10-Dihydroxyoctadecanoic acid-vs-Lachnospira	-0.10204	0.480728	0.543251
9,10-Dihydroxyoctadecanoic acid-vs-Fusobacterium	0.437407	0.001491	0.00591
9,10-Dihydroxyoctadecanoic acid-vs-Bacillus	0.34886	0.013029	0.033552
9,10-Dihydroxyoctadecanoic acid-vs-Dorea	0.119328	0.409151	0.477567
9,10-Dihydroxyoctadecanoic acid-vs-Tyzzerella	0.200672	0.162312	0.237822
9,10-Dihydroxyoctadecanoic acid-vs-Butyrivibrio	0.160816	0.264567	0.344936
9,10-Dihydroxyoctadecanoic acid-vs-Coprobacillus	0.490228	0.000302	0.001662
9,10-Dihydroxyoctadecanoic acid-vs-Selenomonas	0.443265	0.001265	0.005194
9,10-Dihydroxyoctadecanoic acid-vs-Anaerostipes	0.183289	0.202629	0.281304
9,10-Dihydroxyoctadecanoic acid-vs-Peptoclostridium	0.066411	0.646792	0.690116
9,10-Dihydroxyoctadecanoic acid-vs-Dysgonomonas	0.563601	2.03E-05	0.000242
9,10-Dihydroxyoctadecanoic acid-vs-Capnocytophaga	0.056519	0.696647	0.732925
9,10-Dihydroxyoctadecanoic acid-vs-Flavobacterium	0.119904	0.406877	0.475806

3-METHYLORSELLINIC ACID-vs-Alistipes	-0.13815	0.338685	0.415006
3-METHYLORSELLINIC ACID-vs-Faecalibacterium	-0.4739	0.000508	0.002506
3-METHYLORSELLINIC ACID-vs-Oscillibacter	-0.23169	0.105449	0.174113
3-METHYLORSELLINIC ACID-vs-Subdoligranulum	-0.12653	0.381246	0.453951
3-METHYLORSELLINIC ACID-vs-Bilophila	-0.15054	0.296709	0.375609
3-METHYLORSELLINIC ACID-vs-Butyricicoccus	-0.36346	0.009475	0.026017
3-METHYLORSELLINIC ACID-vs-Paraprevotella	-0.12423	0.39005	0.461559
3-METHYLORSELLINIC ACID-vs-Collinsella	-0.38401	0.005903	0.01759
3-METHYLORSELLINIC ACID-vs-Parasutterella	-0.29556	0.037173	0.07835
3-METHYLORSELLINIC ACID-vs-Pyramidobacter	-0.04394	0.76191	0.789881
3-METHYLORSELLINIC ACID-vs-Intestinimonas	-0.03203	0.825243	0.847409
3-METHYLORSELLINIC ACID-vs-Pseudoflavonifractor	-0.06872	0.635383	0.680866
3-METHYLORSELLINIC ACID-vs-Holdemania	-0.06852	0.636331	0.681294
3-METHYLORSELLINIC ACID-vs-Oribacterium	-0.17849	0.214916	0.294413
3-METHYLORSELLINIC ACID-vs-Eubacterium	0.207971	0.147258	0.221244
3-METHYLORSELLINIC ACID-vs-Veillonella	0.663001	1.55E-07	0.000035
3-METHYLORSELLINIC ACID-vs-Lactobacillus	0.548715	3.69E-05	0.00037
3-METHYLORSELLINIC ACID-vs-Agathobacter	0.159568	0.268343	0.347798
3-METHYLORSELLINIC ACID-vs-Enterococcus	0.581657	9.42E-06	0.00015
3-METHYLORSELLINIC ACID-vs-Blautia	0.376903	0.006976	0.020252
3-METHYLORSELLINIC ACID-vs-Streptococcus	0.520864	0.000105	0.000763
3-METHYLORSELLINIC ACID-vs-Erysipelatoclostridium	0.541417	4.91E-05	0.000448
3-METHYLORSELLINIC ACID-vs-Lachnospira	0.022809	0.875067	0.891205
3-METHYLORSELLINIC ACID-vs-Fusobacterium	0.501176	0.00021	0.001252

3-METHYLORSELLINIC ACID-vs-Bacillus	0.466218	0.000644	0.002991
3-METHYLORSELLINIC ACID-vs-Dorea	0.231693	0.105449	0.174113
3-METHYLORSELLINIC ACID-vs-Tyzzerella	0.274046	0.054126	0.104427
3-METHYLORSELLINIC ACID-vs-Butyrivibrio	0.277407	0.051126	0.099803
3-METHYLORSELLINIC ACID-vs-Coprobacillus	0.606531	3.03E-06	0.000081
3-METHYLORSELLINIC ACID-vs-Selenomonas	0.410516	0.003065	0.010536
3-METHYLORSELLINIC ACID-vs-Anaerostipes	0.283361	0.046145	0.0927
3-METHYLORSELLINIC ACID-vs-Peptoclostridium	0.208163	0.146877	0.220804
3-METHYLORSELLINIC ACID-vs-Dysgonomonas	0.482929	0.000382	0.001991
3-METHYLORSELLINIC ACID-vs-Capnocytophaga	-0.03962	0.784739	0.811855
3-METHYLORSELLINIC ACID-vs-Flavobacterium	0.130468	0.366481	0.440806
Ethyl 3-oxohexanoate-vs-Alistipes	-0.20048	0.162723	0.238283
Ethyl 3-oxohexanoate-vs-Faecalibacterium	-0.52845	7.99E-05	0.000634
Ethyl 3-oxohexanoate-vs-Oscillibacter	-0.26982	0.058096	0.110206
Ethyl 3-oxohexanoate-vs-Subdoligranulum	-0.18905	0.188551	0.266675
Ethyl 3-oxohexanoate-vs-Bilophila	-0.21152	0.140315	0.213523
Ethyl 3-oxohexanoate-vs-Butyricicoccus	-0.38478	0.005796	0.017375
Ethyl 3-oxohexanoate-vs-Paraprevotella	-0.09647	0.505121	0.565927
Ethyl 3-oxohexanoate-vs-Collinsella	-0.38679	0.005524	0.016822
Ethyl 3-oxohexanoate-vs-Parasutterella	-0.32264	0.022301	0.051987
Ethyl 3-oxohexanoate-vs-Pyramidobacter	-0.06142	0.671787	0.71159
Ethyl 3-oxohexanoate-vs-Intestinimonas	-0.06679	0.644885	0.688968
Ethyl 3-oxohexanoate-vs-Pseudoflavonifractor	-0.12461	0.388574	0.460471
Ethyl 3-oxohexanoate-vs-Holdemania	-0.16024	0.266305	0.346294
Ethyl 3-oxohexanoate-vs-Oribacterium	-0.24648	0.084429	0.147855

Ethyl 3-oxohexanoate-vs-Eubacterium	0.093301	0.519276	0.577103
Ethyl 3-oxohexanoate-vs-Veillonella	0.621224	1.48E-06	5.93E-05
Ethyl 3-oxohexanoate-vs-Lactobacillus	0.455366	0.000891	0.003885
Ethyl 3-oxohexanoate-vs-Agathobacter	0.033181	0.819059	0.842451
Ethyl 3-oxohexanoate-vs-Enterococcus	0.480048	0.000419	0.002129
Ethyl 3-oxohexanoate-vs-Blautia	0.215942	0.132027	0.204289
Ethyl 3-oxohexanoate-vs-Streptococcus	0.447011	0.001136	0.004738
Ethyl 3-oxohexanoate-vs-Erysipelatoclostridium	0.402161	0.00379	0.012508
Ethyl 3-oxohexanoate-vs-Lachnospira	-0.08552	0.554841	0.609271
Ethyl 3-oxohexanoate-vs-Fusobacterium	0.418391	0.002497	0.008926
Ethyl 3-oxohexanoate-vs-Bacillus	0.361152	0.009973	0.027205
Ethyl 3-oxohexanoate-vs-Dorea	0.122497	0.396731	0.466356
Ethyl 3-oxohexanoate-vs-Tyzzerella	0.134598	0.351374	0.427602
Ethyl 3-oxohexanoate-vs-Butyrivibrio	0.161969	0.261113	0.341688
Ethyl 3-oxohexanoate-vs-Coprobacillus	0.458247	0.000818	0.003612
Ethyl 3-oxohexanoate-vs-Selenomonas	0.387659	0.005411	0.016558
Ethyl 3-oxohexanoate-vs-Anaerostipes	0.166771	0.247048	0.326898
Ethyl 3-oxohexanoate-vs-Peptoclostridium	0.051044	0.724809	0.757422
Ethyl 3-oxohexanoate-vs-Dysgonomonas	0.534022	0.000065	0.000553
Ethyl 3-oxohexanoate-vs-Capnocytophaga	0.0909	0.53013	0.587065
Ethyl 3-oxohexanoate-vs-Flavobacterium	0.131236	0.363641	0.438027
Glufosinate-vs-Alistipes	-0.06411	0.658283	0.699971
Glufosinate-vs-Faecalibacterium	-0.31621	0.025279	0.056953
Glufosinate-vs-Oscillibacter	-0.21076	0.141796	0.214986
Glufosinate-vs-Subdoligranulum	-0.09215	0.524472	0.582617

Glufosinate-vs-Bilophila	-0.16965	0.238861	0.318267
Glufosinate-vs-Butyricicoccus	-0.40082	0.00392	0.012801
Glufosinate-vs-Paraprevotella	-0.05748	0.691747	0.728386
Glufosinate-vs-Collinsella	-0.35952	0.01034	0.028081
Glufosinate-vs-Parasutterella	-0.07227	0.617958	0.665926
Glufosinate-vs-Pyramidobacter	-0.10012	0.489069	0.551173
Glufosinate-vs-Intestinimonas	-0.03616	0.803134	0.827784
Glufosinate-vs-Pseudoflavonifractor	-0.09906	0.493688	0.555371
Glufosinate-vs-Holdemania	0.016375	0.910138	0.92314
Glufosinate-vs-Oribacterium	-0.00619	0.965945	0.97024
Glufosinate-vs-Eubacterium	0.357503	0.010808	0.028973
Glufosinate-vs-Veillonella	0.616807	1.84E-06	0.000063
Glufosinate-vs-Lactobacillus	0.493301	0.000273	0.001547
Glufosinate-vs-Agathobacter	0.418968	0.00246	0.008871
Glufosinate-vs-Enterococcus	0.57042	1.53E-05	0.000205
Glufosinate-vs-Blautia	0.379784	0.006522	0.019247
Glufosinate-vs-Streptococcus	0.531236	7.21E-05	0.000587
Glufosinate-vs-Erysipelatoclostridium	0.568211	1.68E-05	0.000214
Glufosinate-vs-Lachnospira	0.184442	0.199756	0.278404
Glufosinate-vs-Fusobacterium	0.410036	0.003103	0.010623
Glufosinate-vs-Bacillus	0.504538	0.000187	0.001147
Glufosinate-vs-Dorea	0.392269	0.00484	0.015148
Glufosinate-vs-Tyzzerella	0.261465	0.066636	0.123024
Glufosinate-vs-Butyrivibrio	0.440096	0.001383	0.005579
Glufosinate-vs-Coprobacillus	0.67587	7.22E-08	0.000035

Glufosinate-vs-Selenomonas	0.418487	0.002491	0.008919
Glufosinate-vs-Anaerostipes	0.55102	3.37E-05	0.000345
Glufosinate-vs-Peptoclostridium	0.376038	0.007117	0.02059
Glufosinate-vs-Dysgonomonas	0.387275	0.005461	0.016671
Glufosinate-vs-Capnocytophaga	0.175414	0.223046	0.302381
Glufosinate-vs-Flavobacterium	0.169844	0.238321	0.317719
Gentisic acid-vs-Alistipes	-0.15525	0.281689	0.360823
Gentisic acid-vs-Faecalibacterium	-0.50329	0.000195	0.001185
Gentisic acid-vs-Oscillibacter	-0.22122	0.122606	0.194557
Gentisic acid-vs-Subdoligranulum	-0.16341	0.256839	0.336982
Gentisic acid-vs-Bilophila	-0.16485	0.252611	0.332313
Gentisic acid-vs-Butyricicoccus	-0.35299	0.011924	0.031222
Gentisic acid-vs-Paraprevotella	-0.07381	0.610486	0.659304
Gentisic acid-vs-Collinsella	-0.3697	0.008232	0.023142
Gentisic acid-vs-Parasutterella	-0.27731	0.05121	0.09981
Gentisic acid-vs-Pyramidobacter	-0.00408	0.977557	0.981111
Gentisic acid-vs-Intestinimonas	-0.0134	0.926427	0.936603
Gentisic acid-vs-Pseudoflavonifractor	-0.09148	0.527515	0.584951
Gentisic acid-vs-Holdemania	-0.13124	0.363641	0.438027
Gentisic acid-vs-Oribacterium	-0.22094	0.123106	0.195102
Gentisic acid-vs-Eubacterium	0.16072	0.264856	0.344951
Gentisic acid-vs-Veillonella	0.61066	2.49E-06	7.27E-05
Gentisic acid-vs-Lactobacillus	0.512221	0.000143	0.000959
Gentisic acid-vs-Agathobacter	0.087347	0.546396	0.60106
Gentisic acid-vs-Enterococcus	0.501849	0.000205	0.001229

Gentisic acid-vs-Blautia	0.251861	0.077657	0.139435
Gentisic acid-vs-Streptococcus	0.442209	0.001303	0.005308
Gentisic acid-vs-Erysipelatoclostridium	0.471309	0.000551	0.002663
Gentisic acid-vs-Lachnospira	-0.07486	0.605372	0.65549
Gentisic acid-vs-Fusobacterium	0.453637	0.000938	0.004017
Gentisic acid-vs-Bacillus	0.400528	0.003948	0.012842
Gentisic acid-vs-Dorea	0.165426	0.250934	0.330982
Gentisic acid-vs-Tyzzerella	0.193854	0.177364	0.254327
Gentisic acid-vs-Butyrivibrio	0.208836	0.145547	0.219202
Gentisic acid-vs-Coprobacillus	0.52509	9.04E-05	0.000687
Gentisic acid-vs-Selenomonas	0.443553	0.001254	0.00516
Gentisic acid-vs-Anaerostipes	0.224874	0.1164	0.186977
Gentisic acid-vs-Peptoclostridium	0.090996	0.529694	0.587065
Gentisic acid-vs-Dysgonomonas	0.539784	5.22E-05	0.000467
Gentisic acid-vs-Capnocytophaga	0.075534	0.602127	0.65283
Gentisic acid-vs-Flavobacterium	0.179064	0.213414	0.293003
L-Phenylalanine-vs-Alistipes	-0.31726	0.024769	0.056056
L-Phenylalanine-vs-Faecalibacterium	-0.34838	0.013163	0.033792
L-Phenylalanine-vs-Oscillibacter	-0.40514	0.003516	0.011808
L-Phenylalanine-vs-Subdoligranulum	-0.21921	0.126141	0.198142
L-Phenylalanine-vs-Bilophila	-0.33983	0.015755	0.039074
L-Phenylalanine-vs-Butyricicoccus	-0.46382	0.000693	0.003146
L-Phenylalanine-vs-Paraprevotella	-0.14622	0.310951	0.389276
L-Phenylalanine-vs-Collinsella	-0.36634	0.008883	0.024718
L-Phenylalanine-vs-Parasutterella	-0.21095	0.141424	0.214816

L-Phenylalanine-vs-Pyramidobacter	-0.20154	0.160473	0.235962
L-Phenylalanine-vs-Intestinimonas	-0.17647	0.220228	0.29938
L-Phenylalanine-vs-Pseudoflavonifractor	-0.3212	0.022941	0.052883
L-Phenylalanine-vs-Holdemania	-0.11078	0.443745	0.510986
L-Phenylalanine-vs-Oribacterium	-0.10415	0.471641	0.534906
L-Phenylalanine-vs-Eubacterium	0.293926	0.038283	0.080282
L-Phenylalanine-vs-Veillonella	0.589436	6.68E-06	0.000119
L-Phenylalanine-vs-Lactobacillus	0.452773	0.000962	0.004085
L-Phenylalanine-vs-Agathobacter	0.26886	0.059031	0.111467
L-Phenylalanine-vs-Enterococcus	0.557935	2.56E-05	0.000281
L-Phenylalanine-vs-Blautia	0.35395	0.011678	0.030613
L-Phenylalanine-vs-Streptococcus	0.543529	4.52E-05	0.000424
L-Phenylalanine-vs-Erysipelatoclostridium	0.559664	2.38E-05	0.000273
L-Phenylalanine-vs-Lachnospira	0.166291	0.248431	0.328553
L-Phenylalanine-vs-Fusobacterium	0.38449	0.005836	0.017474
L-Phenylalanine-vs-Bacillus	0.269244	0.058655	0.110843
L-Phenylalanine-vs-Dorea	0.293445	0.038615	0.080773
L-Phenylalanine-vs-Tyzzerella	0.248307	0.082081	0.144661
L-Phenylalanine-vs-Butyrivibrio	0.251477	0.078126	0.139873
L-Phenylalanine-vs-Coprobacillus	0.608067	2.82E-06	7.78E-05
L-Phenylalanine-vs-Selenomonas	0.400432	0.003958	0.012856
L-Phenylalanine-vs-Anaerostipes	0.439712	0.001398	0.005612
L-Phenylalanine-vs-Peptoclostridium	0.276831	0.051631	0.100393
L-Phenylalanine-vs-Dysgonomonas	0.396591	0.004354	0.013906
L-Phenylalanine-vs-Capnocytophaga	0.17551	0.222789	0.3022

L-Phenylalanine-vs-Flavobacterium	0.133637	0.354853	0.430571
L-Tryptophan-vs-Alistipes	-0.16802	0.243477	0.323033
L-Tryptophan-vs-Faecalibacterium	-0.43136	0.001763	0.006749
L-Tryptophan-vs-Oscillibacter	-0.27328	0.054831	0.10546
L-Tryptophan-vs-Subdoligranulum	-0.20739	0.148408	0.222299
L-Tryptophan-vs-Bilophila	-0.32034	0.023332	0.053487
L-Tryptophan-vs-Butyricicoccus	-0.45402	0.000927	0.003986
L-Tryptophan-vs-Paraprevotella	-0.03808	0.792901	0.818935
L-Tryptophan-vs-Collinsella	-0.38372	0.005944	0.017689
L-Tryptophan-vs-Parasutterella	-0.26175	0.066326	0.122725
L-Tryptophan-vs-Pyramidobacter	-0.1225	0.396731	0.466356
L-Tryptophan-vs-Intestinimonas	-0.12663	0.380881	0.453735
L-Tryptophan-vs-Pseudoflavonifractor	-0.16898	0.240754	0.319762
L-Tryptophan-vs-Holdemania	-0.18684	0.193863	0.272021
L-Tryptophan-vs-Oribacterium	-0.15995	0.267177	0.347065
L-Tryptophan-vs-Eubacterium	0.187419	0.192467	0.27098
L-Tryptophan-vs-Veillonella	0.634766	7.4E-07	4.96E-05
L-Tryptophan-vs-Lactobacillus	0.548908	3.67E-05	0.000369
L-Tryptophan-vs-Agathobacter	0.264634	0.063286	0.118245
L-Tryptophan-vs-Enterococcus	0.517503	0.000119	0.000822
L-Tryptophan-vs-Blautia	0.233229	0.103094	0.17217
L-Tryptophan-vs-Streptococcus	0.463721	0.000695	0.00315
L-Tryptophan-vs-Erysipelatoclostridium	0.446627	0.001149	0.004782
L-Tryptophan-vs-Lachnospira	-0.03097	0.830922	0.851832
L-Tryptophan-vs-Fusobacterium	0.270588	0.057357	0.10897

L-Tryptophan-vs-Bacillus	0.276255	0.052139	0.101223
L-Tryptophan-vs-Dorea	0.177431	0.217687	0.29739
L-Tryptophan-vs-Tyzzerella	0.200864	0.161902	0.237501
L-Tryptophan-vs-Butyrivibrio	0.236687	0.097944	0.166024
L-Tryptophan-vs-Coprobacillus	0.529796	0.000076	0.000608
L-Tryptophan-vs-Selenomonas	0.374694	0.007342	0.021093
L-Tryptophan-vs-Anaerostipes	0.272605	0.055454	0.106494
L-Tryptophan-vs-Peptoclostridium	0.105594	0.465498	0.530139
L-Tryptophan-vs-Dysgonomonas	0.444802	0.00121	0.004988
L-Tryptophan-vs-Capnocytophaga	0.14401	0.318396	0.396599
L-Tryptophan-vs-Flavobacterium	0.192125	0.181334	0.258529
(9S,10E,12Z,15Z)-9-Hydroperoxy-10,12,15-octadecatrienoic acid-vs-Alistipes	-0.1611	0.263701	0.344168
(9S,10E,12Z,15Z)-9-Hydroperoxy-10,12,15-octadecatrienoic acid-vs-Faecalibacterium	-0.48264	0.000386	0.002005
(9S,10E,12Z,15Z)-9-Hydroperoxy-10,12,15-octadecatrienoic acid-vs-Oscillibacter	-0.21325	0.137027	0.209804
(9S,10E,12Z,15Z)-9-Hydroperoxy-10,12,15-octadecatrienoic acid-vs-Subdoligranulum	-0.09782	0.499176	0.560277
(9S,10E,12Z,15Z)-9-Hydroperoxy-10,12,15-octadecatrienoic acid-vs-Bilophila	-0.1879	0.191309	0.269809
(9S,10E,12Z,15Z)-9-Hydroperoxy-10,12,15-octadecatrienoic acid-vs-Butyricicoccus	-0.43481	0.001603	0.006232
(9S,10E,12Z,15Z)-9-Hydroperoxy-10,12,15-octadecatrienoic acid-vs-Paraprevotella	-0.07928	0.584189	0.636157

(9S,10E,12Z,15Z)-9-Hydroperoxy-10,12,15-octadecatrienoic acid-vs-Collinsella	-0.40216	0.00379	0.012508
(9S,10E,12Z,15Z)-9-Hydroperoxy-10,12,15-octadecatrienoic acid-vs-Parasutterella	-0.21902	0.126482	0.198552
(9S,10E,12Z,15Z)-9-Hydroperoxy-10,12,15-octadecatrienoic acid-vs-Pyramidobacter	-0.03049	0.833506	0.853776
(9S,10E,12Z,15Z)-9-Hydroperoxy-10,12,15-octadecatrienoic acid-vs-Intestinimonas	-0.01628	0.910663	0.923296
(9S,10E,12Z,15Z)-9-Hydroperoxy-10,12,15-octadecatrienoic acid-vs-Pseudoflavonifractor	-0.06449	0.656362	0.698826
(9S,10E,12Z,15Z)-9-Hydroperoxy-10,12,15-octadecatrienoic acid-vs-Holdemania	-0.07563	0.601664	0.652612
(9S,10E,12Z,15Z)-9-Hydroperoxy-10,12,15-octadecatrienoic acid-vs-Oribacterium	-0.1758	0.222019	0.30132
(9S,10E,12Z,15Z)-9-Hydroperoxy-10,12,15-octadecatrienoic acid-vs-Eubacterium	0.164946	0.252331	0.33212
(9S,10E,12Z,15Z)-9-Hydroperoxy-10,12,15-octadecatrienoic acid-vs-Veillonella	0.611236	2.42E-06	7.24E-05
(9S,10E,12Z,15Z)-9-Hydroperoxy-10,12,15-octadecatrienoic acid-vs-Lactobacillus	0.535174	6.22E-05	0.000539
(9S,10E,12Z,15Z)-9-Hydroperoxy-10,12,15-octadecatrienoic acid-vs-Agathobacter	0.165138	0.251771	0.331735
(9S,10E,12Z,15Z)-9-Hydroperoxy-10,12,15-octadecatrienoic acid-vs-Enterococcus	0.542857	4.64E-05	0.000432

(9S,10E,12Z,15Z)-9-Hydroperoxy-10,12,15-octadecatrienoic acid-vs-Blautia	0.333109	0.018088	0.043471
(9S,10E,12Z,15Z)-9-Hydroperoxy-10,12,15-octadecatrienoic acid-vs-Streptococcus	0.495894	0.00025	0.001436
(9S,10E,12Z,15Z)-9-Hydroperoxy-10,12,15-octadecatrienoic acid-vs-Erysipelatoclostridium	0.555726	0.000028	0.000298
(9S,10E,12Z,15Z)-9-Hydroperoxy-10,12,15-octadecatrienoic acid-vs-Lachnospira	0.015126	0.916965	0.92817
(9S,10E,12Z,15Z)-9-Hydroperoxy-10,12,15-octadecatrienoic acid-vs-Fusobacterium	0.484466	0.000364	0.001919
(9S,10E,12Z,15Z)-9-Hydroperoxy-10,12,15-octadecatrienoic acid-vs-Bacillus	0.464586	0.000677	0.003085
(9S,10E,12Z,15Z)-9-Hydroperoxy-10,12,15-octadecatrienoic acid-vs-Dorea	0.207683	0.147832	0.22157
(9S,10E,12Z,15Z)-9-Hydroperoxy-10,12,15-octadecatrienoic acid-vs-Tyzzerella	0.232653	0.103972	0.172939
(9S,10E,12Z,15Z)-9-Hydroperoxy-10,12,15-octadecatrienoic acid-vs-Butyrivibrio	0.284994	0.04485	0.090686
(9S,10E,12Z,15Z)-9-Hydroperoxy-10,12,15-octadecatrienoic acid-vs-Coprobacillus	0.613253	2.19E-06	6.93E-05
(9S,10E,12Z,15Z)-9-Hydroperoxy-10,12,15-octadecatrienoic acid-vs-Selenomonas	0.424058	0.002148	0.007976
(9S,10E,12Z,15Z)-9-Hydroperoxy-10,12,15-octadecatrienoic acid-vs-Anaerostipes	0.275774	0.052566	0.101972

(9S,10E,12Z,15Z)-9-Hydroperoxy-10,12,15-octadecatrienoic acid-vs-Peptoclostridium	0.217095	0.129927	0.202045
(9S,10E,12Z,15Z)-9-Hydroperoxy-10,12,15-octadecatrienoic acid-vs-Dysgonomonas	0.469004	0.000591	0.002804
(9S,10E,12Z,15Z)-9-Hydroperoxy-10,12,15-octadecatrienoic acid-vs-Capnocytophaga	0.04922	0.734279	0.764746
(9S,10E,12Z,15Z)-9-Hydroperoxy-10,12,15-octadecatrienoic acid-vs-Flavobacterium	0.116543	0.420249	0.488913
Indole-vs-Alistipes	-0.24091	0.091922	0.15721
Indole-vs-Faecalibacterium	-0.63256	8.3E-07	5.16E-05
Indole-vs-Oscillibacter	-0.47313	0.000521	0.002551
Indole-vs-Subdoligranulum	-0.44547	0.001187	0.00491
Indole-vs-Bilophila	-0.40552	0.003482	0.011741
Indole-vs-Butyricicoccus	-0.54459	4.34E-05	0.000413
Indole-vs-Paraprevotella	-0.07649	0.597505	0.648951
Indole-vs-Collinsella	-0.49945	0.000222	0.001305
Indole-vs-Parasutterella	-0.13114	0.363996	0.438241
Indole-vs-Pyramidobacter	-0.23381	0.102222	0.171173
Indole-vs-Intestinimonas	-0.36912	0.008341	0.023394
Indole-vs-Pseudoflavonifractor	-0.42924	0.001867	0.007085
Indole-vs-Holdemania	-0.34156	0.015199	0.038073
Indole-vs-Oribacterium	-0.28682	0.043439	0.088844
Indole-vs-Eubacterium	0.226315	0.114017	0.183863
Indole-vs-Veillonella	0.581561	9.46E-06	0.00015
Indole-vs-Lactobacillus	0.447491	0.001121	0.004688

Indole-vs-Agathobacter	0.263289	0.06469	0.120687
Indole-vs-Enterococcus	0.525762	8.82E-05	0.000683
Indole-vs-Blautia	0.217287	0.129579	0.201631
Indole-vs-Streptococcus	0.520288	0.000108	0.000777
Indole-vs-Erysipelatoclostridium	0.454406	0.000917	0.003948
Indole-vs-Lachnospira	0.167731	0.244298	0.323949
Indole-vs-Fusobacterium	0.386026	0.005626	0.01703
Indole-vs-Bacillus	0.261176	0.066947	0.123415
Indole-vs-Dorea	0.192893	0.179562	0.257034
Indole-vs-Tyzzerella	0.141993	0.325292	0.402164
Indole-vs-Butyrivibrio	0.177239	0.218193	0.297591
Indole-vs-Coprobacillus	0.492341	0.000281	0.001575
Indole-vs-Selenomonas	0.332821	0.018194	0.043642
Indole-vs-Anaerostipes	0.289316	0.041566	0.085649
Indole-vs-Peptoclostridium	0.036158	0.803134	0.827784
Indole-vs-Dysgonomonas	0.436927	0.001511	0.005961
Indole-vs-Capnocytophaga	0.284802	0.045001	0.090695
Indole-vs-Flavobacterium	0.198559	0.166872	0.242948
Allopregnanolone-vs-Alistipes	-0.12269	0.395985	0.466141
Allopregnanolone-vs-Faecalibacterium	-0.53834	5.52E-05	0.000488
Allopregnanolone-vs-Oscillibacter	-0.28327	0.046222	0.09278
Allopregnanolone-vs-Subdoligranulum	-0.26194	0.066119	0.122725
Allopregnanolone-vs-Bilophila	-0.3479	0.013299	0.034069
Allopregnanolone-vs-Butyricicoccus	-0.42339	0.002187	0.008076
Allopregnanolone-vs-Paraprevotella	-0.13268	0.358352	0.433758

Allopregnanolone-vs-Collinsella	-0.38593	0.005639	0.017046
Allopregnanolone-vs-Parasutterella	-0.29825	0.035402	0.075385
Allopregnanolone-vs-Pyramidobacter	-0.12346	0.393011	0.463515
Allopregnanolone-vs-Intestinimonas	-0.14814	0.304568	0.383799
Allopregnanolone-vs-Pseudoflavonifractor	-0.17753	0.217434	0.297208
Allopregnanolone-vs-Holdemania	-0.18089	0.208709	0.287975
Allopregnanolone-vs-Oribacterium	-0.20912	0.144979	0.218745
Allopregnanolone-vs-Eubacterium	0.285954	0.044103	0.089685
Allopregnanolone-vs-Veillonella	0.634286	7.59E-07	4.96E-05
Allopregnanolone-vs-Lactobacillus	0.551501	3.31E-05	0.000344
Allopregnanolone-vs-Agathobacter	0.322449	0.022385	0.052037
Allopregnanolone-vs-Enterococcus	0.586843	7.5E-06	0.000126
Allopregnanolone-vs-Blautia	0.279904	0.048987	0.096536
Allopregnanolone-vs-Streptococcus	0.541897	4.82E-05	0.000442
Allopregnanolone-vs-Erysipelatoclostridium	0.489748	0.000306	0.001685
Allopregnanolone-vs-Lachnospira	0.067947	0.639177	0.683752
Allopregnanolone-vs-Fusobacterium	0.326194	0.020787	0.049009
Allopregnanolone-vs-Bacillus	0.307179	0.030014	0.065713
Allopregnanolone-vs-Dorea	0.20605	0.151116	0.225133
Allopregnanolone-vs-Tyzzerella	0.213253	0.137027	0.209804
Allopregnanolone-vs-Butyrivibrio	0.243697	0.088113	0.152374
Allopregnanolone-vs-Coprobacillus	0.556014	2.76E-05	0.000296
Allopregnanolone-vs-Selenomonas	0.314478	0.026134	0.058454
Allopregnanolone-vs-Anaerostipes	0.311116	0.027865	0.061716
Allopregnanolone-vs-Peptoclostridium	0.141128	0.328276	0.405047

Allopregnanolone-vs-Dysgonomonas	0.509628	0.000157	0.001007
Allopregnanolone-vs-Capnocytophaga	0.104058	0.472052	0.534906
Allopregnanolone-vs-Flavobacterium	0.229292	0.109211	0.178428
O-(Carbamoylamino)-D-serine-vs-Alistipes	-0.20427	0.154752	0.229457
O-(Carbamoylamino)-D-serine-vs-Faecalibacterium	-0.49536	0.000255	0.001458
O-(Carbamoylamino)-D-serine-vs-Oscillibacter	-0.33442	0.017613	0.042534
O-(Carbamoylamino)-D-serine-vs-Subdoligranulum	-0.23605	0.098878	0.167151
O-(Carbamoylamino)-D-serine-vs-Bilophila	-0.35063	0.012545	0.03257
O-(Carbamoylamino)-D-serine-vs-Butyricicoccus	-0.48213	0.000392	0.002025
O-(Carbamoylamino)-D-serine-vs-Paraprevotella	-0.13307	0.356908	0.432431
O-(Carbamoylamino)-D-serine-vs-Collinsella	-0.41966	0.002415	0.008773
O-(Carbamoylamino)-D-serine-vs-Parasutterella	-0.23204	0.104918	0.17393
O-(Carbamoylamino)-D-serine-vs-Pyramidobacter	-0.15167	0.293048	0.372684
O-(Carbamoylamino)-D-serine-vs-Intestinimonas	-0.15954	0.268441	0.347798
O-(Carbamoylamino)-D-serine-vs-Pseudoflavonifractor	-0.20324	0.156888	0.231513
O-(Carbamoylamino)-D-serine-vs-Holdemania	-0.11144	0.441035	0.509044
O-(Carbamoylamino)-D-serine-vs-Oribacterium	-0.13237	0.359484	0.434705
O-(Carbamoylamino)-D-serine-vs-Eubacterium	0.28442	0.045302	0.091158
O-(Carbamoylamino)-D-serine-vs-Veillonella	0.6267	1.12E-06	5.58E-05
O-(Carbamoylamino)-D-serine-vs-Lactobacillus	0.591018	6.22E-06	0.000116
O-(Carbamoylamino)-D-serine-vs-Agathobacter	0.26159	0.0665	0.122865
O-(Carbamoylamino)-D-serine-vs-Enterococcus	0.614824	2.03E-06	6.64E-05
O-(Carbamoylamino)-D-serine-vs-Blautia	0.331163	0.018816	0.044829
O-(Carbamoylamino)-D-serine-vs-Streptococcus	0.602948	3.59E-06	8.88E-05
O-(Carbamoylamino)-D-serine-vs-Erysipelatoclostridium	0.616017	1.92E-06	6.43E-05

O-(Carbamoylamino)-D-serine-vs-Lachnospira	0.181497	0.207155	0.286467
O-(Carbamoylamino)-D-serine-vs-Fusobacterium	0.521012	0.000105	0.000761
O-(Carbamoylamino)-D-serine-vs-Bacillus	0.391247	0.004962	0.015393
O-(Carbamoylamino)-D-serine-vs-Dorea	0.227211	0.112554	0.181858
O-(Carbamoylamino)-D-serine-vs-Tyzzerella	0.280678	0.048338	0.095637
O-(Carbamoylamino)-D-serine-vs-Butyrivibrio	0.290439	0.040746	0.084237
O-(Carbamoylamino)-D-serine-vs-Coprobacillus	0.627893	1.06E-06	5.56E-05
O-(Carbamoylamino)-D-serine-vs-Selenomonas	0.423837	0.002161	0.007991
O-(Carbamoylamino)-D-serine-vs-Anaerostipes	0.339514	0.015859	0.039214
O-(Carbamoylamino)-D-serine-vs-Peptoclostridium	0.172116	0.232007	0.310969
O-(Carbamoylamino)-D-serine-vs-Dysgonomonas	0.50187	0.000205	0.001229
O-(Carbamoylamino)-D-serine-vs-Capnocytophaga	0.17537	0.223166	0.302381
O-(Carbamoylamino)-D-serine-vs-Flavobacterium	0.208285	0.146635	0.220573
Indole-3-acetic acid-vs-Alistipes	-0.09743	0.500871	0.561672
Indole-3-acetic acid-vs-Faecalibacterium	-0.47726	0.000457	0.002297
Indole-3-acetic acid-vs-Oscillibacter	-0.21239	0.138664	0.211659
Indole-3-acetic acid-vs-Subdoligranulum	-0.15092	0.295463	0.374797
Indole-3-acetic acid-vs-Bilophila	-0.30295	0.032471	0.069984
Indole-3-acetic acid-vs-Butyricicoccus	-0.41868	0.002478	0.0089
Indole-3-acetic acid-vs-Paraprevotella	-0.08831	0.541977	0.597786
Indole-3-acetic acid-vs-Collinsella	-0.3673	0.008692	0.024243
Indole-3-acetic acid-vs-Parasutterella	-0.17609	0.22125	0.30044
Indole-3-acetic acid-vs-Pyramidobacter	-0.00802	0.955923	0.961728
Indole-3-acetic acid-vs-Intestinimonas	-0.057	0.694195	0.730654
Indole-3-acetic acid-vs-Pseudoflavonifractor	-0.08283	0.567398	0.621138

Indole-3-acetic acid-vs-Holdemania	-0.0837	0.563347	0.61752
Indole-3-acetic acid-vs-Oribacterium	-0.0836	0.563797	0.61774
Indole-3-acetic acid-vs-Eubacterium	0.332533	0.018301	0.043813
Indole-3-acetic acid-vs-Veillonella	0.590492	6.37E-06	0.000116
Indole-3-acetic acid-vs-Lactobacillus	0.588956	6.82E-06	0.00012
Indole-3-acetic acid-vs-Agathobacter	0.307083	0.030068	0.065774
Indole-3-acetic acid-vs-Enterococcus	0.616711	1.85E-06	0.000063
Indole-3-acetic acid-vs-Blautia	0.275294	0.052995	0.102565
Indole-3-acetic acid-vs-Streptococcus	0.517791	0.000118	0.000819
Indole-3-acetic acid-vs-Erysipelatoclostridium	0.544106	4.42E-05	0.000418
Indole-3-acetic acid-vs-Lachnospira	0.030492	0.833506	0.853776
Indole-3-acetic acid-vs-Fusobacterium	0.344058	0.014424	0.036459
Indole-3-acetic acid-vs-Bacillus	0.392173	0.004851	0.015164
Indole-3-acetic acid-vs-Dorea	0.229676	0.108602	0.177784
Indole-3-acetic acid-vs-Tyzzerella	0.299112	0.034848	0.074333
Indole-3-acetic acid-vs-Butyrivibrio	0.338391	0.016232	0.040017
Indole-3-acetic acid-vs-Coprobacillus	0.578679	1.07E-05	0.00016
Indole-3-acetic acid-vs-Selenomonas	0.412533	0.00291	0.010114
Indole-3-acetic acid-vs-Anaerostipes	0.330516	0.019063	0.045375
Indole-3-acetic acid-vs-Peptoclostridium	0.213637	0.136304	0.209213
Indole-3-acetic acid-vs-Dysgonomonas	0.454502	0.000914	0.003944
Indole-3-acetic acid-vs-Capnocytophaga	0.169364	0.239671	0.319005
Indole-3-acetic acid-vs-Flavobacterium	0.260984	0.067156	0.123524
Cortisol-vs-Alistipes	-0.1564	0.278088	0.357316
Cortisol-vs-Faecalibacterium	-0.46459	0.000677	0.003085

Cortisol-vs-Oscillibacter	-0.29181	0.03976	0.082475
Cortisol-vs-Subdoligranulum	-0.19251	0.180446	0.257855
Cortisol-vs-Bilophila	-0.35251	0.012048	0.031515
Cortisol-vs-Butyricicoccus	-0.38968	0.005154	0.015911
Cortisol-vs-Paraprevotella	-0.09349	0.518413	0.577103
Cortisol-vs-Collinsella	-0.36048	0.010123	0.027582
Cortisol-vs-Parasutterella	-0.19846	0.167082	0.242948
Cortisol-vs-Pyramidobacter	-0.09397	0.516257	0.575291
Cortisol-vs-Intestinimonas	-0.1054	0.466315	0.530826
Cortisol-vs-Pseudoflavonifractor	-0.12778	0.376526	0.449624
Cortisol-vs-Holdemania	-0.10732	0.458184	0.523008
Cortisol-vs-Oribacterium	-0.11328	0.433476	0.50125
Cortisol-vs-Eubacterium	0.322353	0.022428	0.052087
Cortisol-vs-Veillonella	0.638127	6.19E-07	0.000046
Cortisol-vs-Lactobacillus	0.623241	1.34E-06	5.93E-05
Cortisol-vs-Agathobacter	0.306122	0.030613	0.06679
Cortisol-vs-Enterococcus	0.593373	5.59E-06	0.000107
Cortisol-vs-Blautia	0.357503	0.010808	0.028973
Cortisol-vs-Streptococcus	0.542665	4.68E-05	0.000434
Cortisol-vs-Erysipelatoclostridium	0.527299	8.34E-05	0.00066
Cortisol-vs-Lachnospira	0.070828	0.624998	0.672346
Cortisol-vs-Fusobacterium	0.351164	0.012402	0.032237
Cortisol-vs-Bacillus	0.402929	0.003718	0.012351
Cortisol-vs-Dorea	0.248307	0.082081	0.144661
Cortisol-vs-Tyzzerella	0.271068	0.056899	0.108432

Cortisol-vs-Butyrivibrio	0.314478	0.026134	0.058454
Cortisol-vs-Coprobacillus	0.612293	2.3E-06	7.14E-05
Cortisol-vs-Selenomonas	0.418295	0.002503	0.008926
Cortisol-vs-Anaerostipes	0.371525	0.007897	0.022376
Cortisol-vs-Peptoclostridium	0.228139	0.111053	0.180134
Cortisol-vs-Dysgonomonas	0.430684	0.001795	0.006843
Cortisol-vs-Capnocytophaga	0.101753	0.481975	0.544412
Cortisol-vs-Flavobacterium	0.215174	0.133442	0.205709
16-hydroxypalmitic acid-vs-Alistipes	-0.06055	0.67615	0.715601
16-hydroxypalmitic acid-vs-Faecalibacterium	-0.45796	0.000825	0.003637
16-hydroxypalmitic acid-vs-Oscillibacter	-0.12423	0.39005	0.461559
16-hydroxypalmitic acid-vs-Subdoligranulum	-0.0739	0.610021	0.659087
16-hydroxypalmitic acid-vs-Bilophila	-0.13489	0.350334	0.426755
16-hydroxypalmitic acid-vs-Butyricicoccus	-0.34137	0.01526	0.038111
16-hydroxypalmitic acid-vs-Paraprevotella	-0.03164	0.827307	0.848827
16-hydroxypalmitic acid-vs-Collinsella	-0.34415	0.014395	0.036427
16-hydroxypalmitic acid-vs-Parasutterella	-0.21815	0.128023	0.199836
16-hydroxypalmitic acid-vs-Pyramidobacter	0.076399	0.597966	0.649168
16-hydroxypalmitic acid-vs-Intestinimonas	0.088019	0.543301	0.598981
16-hydroxypalmitic acid-vs-Pseudoflavonifractor	0.013493	0.925901	0.936452
16-hydroxypalmitic acid-vs-Holdemania	-0.0521	0.719345	0.752662
16-hydroxypalmitic acid-vs-Oribacterium	-0.10204	0.480728	0.543251
16-hydroxypalmitic acid-vs-Eubacterium	0.229964	0.108147	0.177156
16-hydroxypalmitic acid-vs-Veillonella	0.629676	9.64E-07	5.32E-05
16-hydroxypalmitic acid-vs-Lactobacillus	0.586267	7.69E-06	0.000128

16-hydroxypalmitic acid-vs-Agathobacter	0.187323	0.192699	0.271
16-hydroxypalmitic acid-vs-Enterococcus	0.550252	3.48E-05	0.000353
16-hydroxypalmitic acid-vs-Blautia	0.274046	0.054126	0.104427
16-hydroxypalmitic acid-vs-Streptococcus	0.494934	0.000258	0.001476
16-hydroxypalmitic acid-vs-Erysipelatoclostridium	0.508571	0.000163	0.001031
16-hydroxypalmitic acid-vs-Lachnospira	-0.03914	0.787287	0.813814
16-hydroxypalmitic acid-vs-Fusobacterium	0.48898	0.000314	0.001705
16-hydroxypalmitic acid-vs-Bacillus	0.455462	0.000889	0.003881
16-hydroxypalmitic acid-vs-Dorea	0.216807	0.130449	0.202604
16-hydroxypalmitic acid-vs-Tyzzerella	0.237071	0.097384	0.16564
16-hydroxypalmitic acid-vs-Butyrivibrio	0.333397	0.017982	0.043343
16-hydroxypalmitic acid-vs-Coprobacillus	0.558511	0.000025	0.000277
16-hydroxypalmitic acid-vs-Selenomonas	0.469292	0.000586	0.002785
16-hydroxypalmitic acid-vs-Anaerostipes	0.315534	0.025609	0.057591
16-hydroxypalmitic acid-vs-Peptoclostridium	0.140936	0.328941	0.405466
16-hydroxypalmitic acid-vs-Dysgonomonas	0.53575	6.09E-05	0.000529
16-hydroxypalmitic acid-vs-Capnocytophaga	0.141417	0.327279	0.404219
16-hydroxypalmitic acid-vs-Flavobacterium	0.19539	0.173887	0.250243
Retinyl acetate-vs-Alistipes	-0.12144	0.400846	0.470082
Retinyl acetate-vs-Faecalibacterium	-0.51059	0.000152	0.000997
Retinyl acetate-vs-Oscillibacter	-0.28221	0.047077	0.094116
Retinyl acetate-vs-Subdoligranulum	-0.30353	0.032127	0.069422
Retinyl acetate-vs-Bilophila	-0.24908	0.081108	0.143762
Retinyl acetate-vs-Butyricicoccus	-0.37969	0.006537	0.019247
Retinyl acetate-vs-Paraprevotella	-0.12336	0.393382	0.463515

Retinyl acetate-vs-Collinsella	-0.3746	0.007358	0.021113
Retinyl acetate-vs-Parasutterella	-0.29546	0.037238	0.07842
Retinyl acetate-vs-Pyramidobacter	-0.18886	0.189008	0.267039
Retinyl acetate-vs-Intestinimonas	-0.13143	0.362933	0.437598
Retinyl acetate-vs-Pseudoflavonifractor	-0.14593	0.311916	0.390287
Retinyl acetate-vs-Holdemania	-0.16706	0.246221	0.326151
Retinyl acetate-vs-Oribacterium	-0.09417	0.515396	0.574847
Retinyl acetate-vs-Eubacterium	0.304586	0.031503	0.068311
Retinyl acetate-vs-Veillonella	0.621609	1.45E-06	5.93E-05
Retinyl acetate-vs-Lactobacillus	0.533733	6.57E-05	0.000557
Retinyl acetate-vs-Agathobacter	0.231501	0.105746	0.174488
Retinyl acetate-vs-Enterococcus	0.525666	8.85E-05	0.000683
Retinyl acetate-vs-Blautia	0.303914	0.031899	0.069109
Retinyl acetate-vs-Streptococcus	0.472941	0.000524	0.002562
Retinyl acetate-vs-Erysipelatoclostridium	0.480048	0.000419	0.002129
Retinyl acetate-vs-Lachnospira	0.068235	0.637753	0.682523
Retinyl acetate-vs-Fusobacterium	0.403794	0.003638	0.01215
Retinyl acetate-vs-Bacillus	0.331765	0.018588	0.044415
Retinyl acetate-vs-Dorea	0.211044	0.141239	0.214666
Retinyl acetate-vs-Tyzzerella	0.232557	0.104119	0.172952
Retinyl acetate-vs-Butyrivibrio	0.220456	0.123944	0.19568
Retinyl acetate-vs-Coprobacillus	0.538439	0.000055	0.000488
Retinyl acetate-vs-Selenomonas	0.439712	0.001398	0.005612
Retinyl acetate-vs-Anaerostipes	0.323217	0.022049	0.051448
Retinyl acetate-vs-Peptoclostridium	0.123938	0.391159	0.461772

Retinyl acetate-vs-Dysgonomonas	0.514238	0.000133	0.000901
Retinyl acetate-vs-Capnocytophaga	0.136999	0.342769	0.418977
Retinyl acetate-vs-Flavobacterium	0.278944	0.049801	0.097599
Pregnane-3,20-dione-vs-Alistipes	-0.14449	0.316768	0.395165
Pregnane-3,20-dione-vs-Faecalibacterium	-0.46699	0.000629	0.002938
Pregnane-3,20-dione-vs-Oscillibacter	-0.23016	0.107845	0.176894
Pregnane-3,20-dione-vs-Subdoligranulum	-0.15784	0.27363	0.353598
Pregnane-3,20-dione-vs-Bilophila	-0.37479	0.007326	0.021071
Pregnane-3,20-dione-vs-Butyricicoccus	-0.4158	0.002673	0.009395
Pregnane-3,20-dione-vs-Paraprevotella	-0.09349	0.518413	0.577103
Pregnane-3,20-dione-vs-Collinsella	-0.32053	0.023244	0.053385
Pregnane-3,20-dione-vs-Parasutterella	-0.20768	0.147832	0.22157
Pregnane-3,20-dione-vs-Pyramidobacter	-0.04643	0.748809	0.777597
Pregnane-3,20-dione-vs-Intestinimonas	-0.05902	0.683932	0.721993
Pregnane-3,20-dione-vs-Pseudoflavonifractor	-0.09887	0.49453	0.556067
Pregnane-3,20-dione-vs-Holdemania	-0.10406	0.472052	0.534906
Pregnane-3,20-dione-vs-Oribacterium	-0.10944	0.44933	0.516221
Pregnane-3,20-dione-vs-Eubacterium	0.350492	0.012582	0.032603
Pregnane-3,20-dione-vs-Veillonella	0.654454	2.54E-07	0.000035
Pregnane-3,20-dione-vs-Lactobacillus	0.635054	7.29E-07	4.96E-05
Pregnane-3,20-dione-vs-Agathobacter	0.365858	0.008979	0.024903
Pregnane-3,20-dione-vs-Enterococcus	0.611236	2.42E-06	7.24E-05
Pregnane-3,20-dione-vs-Blautia	0.334742	0.017496	0.042335
Pregnane-3,20-dione-vs-Streptococcus	0.577239	1.14E-05	0.000166
Pregnane-3,20-dione-vs-Erysipelatoclostridium	0.521825	0.000102	0.000748

Pregnane-3,20-dione-vs-Lachnospira	0.105306	0.466724	0.531048
Pregnane-3,20-dione-vs-Fusobacterium	0.373445	0.007556	0.021534
Pregnane-3,20-dione-vs-Bacillus	0.373061	0.007623	0.021675
Pregnane-3,20-dione-vs-Dorea	0.30545	0.031	0.067397
Pregnane-3,20-dione-vs-Tyzzerella	0.258103	0.070342	0.12834
Pregnane-3,20-dione-vs-Butyrivibrio	0.337911	0.016394	0.040296
Pregnane-3,20-dione-vs-Coprobacillus	0.603938	3.43E-06	8.88E-05
Pregnane-3,20-dione-vs-Selenomonas	0.343289	0.014659	0.036982
Pregnane-3,20-dione-vs-Anaerostipes	0.391693	0.004908	0.015285
Pregnane-3,20-dione-vs-Peptoclostridium	0.231405	0.105895	0.174618
Pregnane-3,20-dione-vs-Dysgonomonas	0.467371	0.000622	0.002909
Pregnane-3,20-dione-vs-Capnocytophaga	0.14036	0.330943	0.407528
Pregnane-3,20-dione-vs-Flavobacterium	0.241873	0.090595	0.155475
4-Oxo-4-(3-pyridinyl)butanal-vs-Alistipes	-0.19962	0.164581	0.240578
4-Oxo-4-(3-pyridinyl)butanal-vs-Faecalibacterium	-0.5223	0.0001	0.000744
4-Oxo-4-(3-pyridinyl)butanal-vs-Oscillibacter	-0.30257	0.032703	0.070422
4-Oxo-4-(3-pyridinyl)butanal-vs-Subdoligranulum	-0.23333	0.102948	0.172158
4-Oxo-4-(3-pyridinyl)butanal-vs-Bilophila	-0.234	0.101932	0.170918
4-Oxo-4-(3-pyridinyl)butanal-vs-Butyricicoccus	-0.37095	0.008001	0.022595
4-Oxo-4-(3-pyridinyl)butanal-vs-Paraprevotella	-0.07822	0.589224	0.640517
4-Oxo-4-(3-pyridinyl)butanal-vs-Collinsella	-0.38439	0.00585	0.017493
4-Oxo-4-(3-pyridinyl)butanal-vs-Parasutterella	-0.30804	0.029531	0.064884
4-Oxo-4-(3-pyridinyl)butanal-vs-Pyramidobacter	-0.08053	0.578264	0.630534
4-Oxo-4-(3-pyridinyl)butanal-vs-Intestinimonas	-0.08783	0.544184	0.599157
4-Oxo-4-(3-pyridinyl)butanal-vs-Pseudoflavonifractor	-0.16562	0.250376	0.330422

4-Oxo-4-(3-pyridinyl)butanal-vs-Holdemania	-0.19443	0.176054	0.252595
4-Oxo-4-(3-pyridinyl)butanal-vs-Oribacterium	-0.27952	0.049311	0.097021
4-Oxo-4-(3-pyridinyl)butanal-vs-Eubacterium	0.078607	0.587391	0.639083
4-Oxo-4-(3-pyridinyl)butanal-vs-Veillonella	0.646291	3.99E-07	4.07E-05
4-Oxo-4-(3-pyridinyl)butanal-vs-Lactobacillus	0.467851	0.000613	0.002878
4-Oxo-4-(3-pyridinyl)butanal-vs-Agathobacter	0.006867	0.962252	0.967704
4-Oxo-4-(3-pyridinyl)butanal-vs-Enterococcus	0.479472	0.000427	0.002155
4-Oxo-4-(3-pyridinyl)butanal-vs-Blautia	0.256567	0.072089	0.130666
4-Oxo-4-(3-pyridinyl)butanal-vs-Streptococcus	0.462377	0.000723	0.00325
4-Oxo-4-(3-pyridinyl)butanal-vs-Erysipelatoclostridium	0.431837	0.00174	0.006682
4-Oxo-4-(3-pyridinyl)butanal-vs-Lachnospira	-0.05767	0.690768	0.727664
4-Oxo-4-(3-pyridinyl)butanal-vs-Fusobacterium	0.445186	0.001197	0.004942
4-Oxo-4-(3-pyridinyl)butanal-vs-Bacillus	0.352221	0.012123	0.031611
4-Oxo-4-(3-pyridinyl)butanal-vs-Dorea	0.108475	0.453343	0.519372
4-Oxo-4-(3-pyridinyl)butanal-vs-Tyzzerella	0.144298	0.317419	0.395778
4-Oxo-4-(3-pyridinyl)butanal-vs-Butyrivibrio	0.123938	0.391159	0.461772
4-Oxo-4-(3-pyridinyl)butanal-vs-Coprobacillus	0.479664	0.000424	0.002146
4-Oxo-4-(3-pyridinyl)butanal-vs-Selenomonas	0.389676	0.005154	0.015911
4-Oxo-4-(3-pyridinyl)butanal-vs-Anaerostipes	0.175222	0.223562	0.302423
4-Oxo-4-(3-pyridinyl)butanal-vs-Peptoclostridium	0.054406	0.707471	0.742113
4-Oxo-4-(3-pyridinyl)butanal-vs-Dysgonomonas	0.561777	2.19E-05	0.000257
4-Oxo-4-(3-pyridinyl)butanal-vs-Capnocytophaga	0.055558	0.70156	0.736534
4-Oxo-4-(3-pyridinyl)butanal-vs-Flavobacterium	0.102521	0.478655	0.541647
2-Hydroxy-2-phenyl-1,3-propanediyl dicarbamate-vs-Alistipes	-0.16475	0.252892	0.332506

2-Hydroxy-2-phenyl-1,3-propanediyl dicarbamate-vs-Faecalibacterium	-0.51904	0.000112	0.000794
2-Hydroxy-2-phenyl-1,3-propanediyl dicarbamate-vs-Oscillibacter	-0.23236	0.104414	0.173325
2-Hydroxy-2-phenyl-1,3-propanediyl dicarbamate-vs-Subdoligranulum	-0.18108	0.208218	0.287457
2-Hydroxy-2-phenyl-1,3-propanediyl dicarbamate-vs-Bilophila	-0.16917	0.240212	0.319383
2-Hydroxy-2-phenyl-1,3-propanediyl dicarbamate-vs-Butyricicoccus	-0.35491	0.011437	0.030172
2-Hydroxy-2-phenyl-1,3-propanediyl dicarbamate-vs-Paraprevotella	-0.06872	0.635383	0.680866
2-Hydroxy-2-phenyl-1,3-propanediyl dicarbamate-vs-Collinsella	-0.40206	0.003799	0.012522
2-Hydroxy-2-phenyl-1,3-propanediyl dicarbamate-vs-Parasutterella	-0.31371	0.026522	0.059215
2-Hydroxy-2-phenyl-1,3-propanediyl dicarbamate-vs-Pyramidobacter	-0.02732	0.850602	0.869139
2-Hydroxy-2-phenyl-1,3-propanediyl dicarbamate-vs-Intestinimonas	-0.01253	0.931161	0.940624
2-Hydroxy-2-phenyl-1,3-propanediyl dicarbamate-vs-Pseudoflavonifractor	-0.10415	0.471641	0.534906
2-Hydroxy-2-phenyl-1,3-propanediyl dicarbamate-vs-Holdemania	-0.14948	0.30015	0.379386

2-Hydroxy-2-phenyl-1,3-propanediyl dicarbamate-vs-Oribacterium	-0.22574	0.114966	0.185272
2-Hydroxy-2-phenyl-1,3-propanediyl dicarbamate-vs-Eubacterium	0.140936	0.328941	0.405466
2-Hydroxy-2-phenyl-1,3-propanediyl dicarbamate-vs-Veillonella	0.621801	1.44E-06	5.93E-05
2-Hydroxy-2-phenyl-1,3-propanediyl dicarbamate-vs-Lactobacillus	0.51443	0.000133	0.0009
2-Hydroxy-2-phenyl-1,3-propanediyl dicarbamate-vs-Agathobacter	0.082449	0.569202	0.622565
2-Hydroxy-2-phenyl-1,3-propanediyl dicarbamate-vs-Enterococcus	0.51491	0.00013	0.000892
2-Hydroxy-2-phenyl-1,3-propanediyl dicarbamate-vs-Blautia	0.265498	0.062396	0.116846
2-Hydroxy-2-phenyl-1,3-propanediyl dicarbamate-vs-Streptococcus	0.465258	0.000663	0.003051
2-Hydroxy-2-phenyl-1,3-propanediyl dicarbamate-vs-Erysipelatoclostridium	0.466218	0.000644	0.002991
2-Hydroxy-2-phenyl-1,3-propanediyl dicarbamate-vs-Lachnospira	-0.02166	0.881333	0.896852
2-Hydroxy-2-phenyl-1,3-propanediyl dicarbamate-vs-Fusobacterium	0.478319	0.000442	0.002226
2-Hydroxy-2-phenyl-1,3-propanediyl dicarbamate-vs-Bacillus	0.416951	0.002594	0.009168

2-Hydroxy-2-phenyl-1,3-propanediyl dicarbamate-vs-Dorea	0.156303	0.278387	0.35733
2-Hydroxy-2-phenyl-1,3-propanediyl dicarbamate-vs-Tyzzerella	0.164946	0.252331	0.33212
2-Hydroxy-2-phenyl-1,3-propanediyl dicarbamate-vs-Butyrivibrio	0.213157	0.137208	0.209952
2-Hydroxy-2-phenyl-1,3-propanediyl dicarbamate-vs-Coprobacillus	0.525186	9.01E-05	0.000687
2-Hydroxy-2-phenyl-1,3-propanediyl dicarbamate-vs-Selenomonas	0.405234	0.003508	0.011795
2-Hydroxy-2-phenyl-1,3-propanediyl dicarbamate-vs-Anaerostipes	0.207203	0.148792	0.222741
2-Hydroxy-2-phenyl-1,3-propanediyl dicarbamate-vs-Peptoclostridium	0.089652	0.535818	0.592044
2-Hydroxy-2-phenyl-1,3-propanediyl dicarbamate-vs-Dysgonomonas	0.547659	3.85E-05	0.00038
2-Hydroxy-2-phenyl-1,3-propanediyl dicarbamate-vs-Capnocytophaga	0.094454	0.514106	0.573666
2-Hydroxy-2-phenyl-1,3-propanediyl dicarbamate-vs-Flavobacterium	0.174358	0.22589	0.304908
2,5-Dihydroxy-3-undecyl-1,4-benzoquinone-vs-Alistipes	-0.11731	0.417171	0.485559
2,5-Dihydroxy-3-undecyl-1,4-benzoquinone-vs-Faecalibacterium	-0.46238	0.000723	0.00325
2,5-Dihydroxy-3-undecyl-1,4-benzoquinone-vs-Oscillibacter	-0.22055	0.123776	0.195664

2,5-Dihydroxy-3-undecyl-1,4-benzoquinone-vs-Subdoligranulum	-0.0934	0.518844	0.577103
2,5-Dihydroxy-3-undecyl-1,4-benzoquinone-vs-Bilophila	-0.21854	0.127337	0.19939
2,5-Dihydroxy-3-undecyl-1,4-benzoquinone-vs-Butyricicoccus	-0.3795	0.006566	0.019311
2,5-Dihydroxy-3-undecyl-1,4-benzoquinone-vs-Paraprevotella	-0.07352	0.611884	0.66024
2,5-Dihydroxy-3-undecyl-1,4-benzoquinone-vs-Collinsella	-0.38651	0.005562	0.016897
2,5-Dihydroxy-3-undecyl-1,4-benzoquinone-vs-Parasutterella	-0.21671	0.130624	0.202679
2,5-Dihydroxy-3-undecyl-1,4-benzoquinone-vs-Pyramidobacter	-0.01455	0.920117	0.930982
2,5-Dihydroxy-3-undecyl-1,4-benzoquinone-vs-Intestinimonas	-0.03453	0.811858	0.835736
2,5-Dihydroxy-3-undecyl-1,4-benzoquinone-vs-Pseudoflavonifractor	-0.05998	0.679064	0.718074
2,5-Dihydroxy-3-undecyl-1,4-benzoquinone-vs-Holdemania	-0.08523	0.55618	0.610472
2,5-Dihydroxy-3-undecyl-1,4-benzoquinone-vs-Oribacterium	-0.15957	0.268343	0.347798
2,5-Dihydroxy-3-undecyl-1,4-benzoquinone-vs-Eubacterium	0.210852	0.14161	0.214967
2,5-Dihydroxy-3-undecyl-1,4-benzoquinone-vs-Veillonella	0.650132	3.23E-07	3.82E-05

2,5-Dihydroxy-3-undecyl-1,4-benzoquinone-vs-Lactobacillus	0.576375	1.18E-05	0.000169
2,5-Dihydroxy-3-undecyl-1,4-benzoquinone-vs-Agathobacter	0.182809	0.203835	0.282662
2,5-Dihydroxy-3-undecyl-1,4-benzoquinone-vs-Enterococcus	0.542377	4.73E-05	0.000437
2,5-Dihydroxy-3-undecyl-1,4-benzoquinone-vs-Blautia	0.319664	0.02364	0.053994
2,5-Dihydroxy-3-undecyl-1,4-benzoquinone-vs-Streptococcus	0.520192	0.000108	0.000777
2,5-Dihydroxy-3-undecyl-1,4-benzoquinone-vs-Erysipelatoclostridium	0.523361	9.63E-05	0.00072
2,5-Dihydroxy-3-undecyl-1,4-benzoquinone-vs-Lachnospira	0.003409	0.981253	0.983231
2,5-Dihydroxy-3-undecyl-1,4-benzoquinone-vs-Fusobacterium	0.483601	0.000374	0.001957
2,5-Dihydroxy-3-undecyl-1,4-benzoquinone-vs-Bacillus	0.42617	0.00203	0.007584
2,5-Dihydroxy-3-undecyl-1,4-benzoquinone-vs-Dorea	0.22497	0.11624	0.186841
2,5-Dihydroxy-3-undecyl-1,4-benzoquinone-vs-Tyzzerella	0.241969	0.090463	0.155475
2,5-Dihydroxy-3-undecyl-1,4-benzoquinone-vs-Butyrivibrio	0.282305	0.046998	0.094035
2,5-Dihydroxy-3-undecyl-1,4-benzoquinone-vs-Coprobacillus	0.594622	5.28E-06	0.000103
2,5-Dihydroxy-3-undecyl-1,4-benzoquinone-vs-Selenomonas	0.392461	0.004817	0.015096

2,5-Dihydroxy-3-undecyl-1,4-benzoquinone-vs-Anaerostipes	0.288067	0.042494	0.087415
2,5-Dihydroxy-3-undecyl-1,4-benzoquinone-vs-Peptoclostridium	0.1612	0.263412	0.343972
2,5-Dihydroxy-3-undecyl-1,4-benzoquinone-vs-Dysgonomonas	0.525282	8.98E-05	0.000687
2,5-Dihydroxy-3-undecyl-1,4-benzoquinone-vs-Capnocytophaga	0.058727	0.685395	0.72323
2,5-Dihydroxy-3-undecyl-1,4-benzoquinone-vs-Flavobacterium	0.144778	0.315794	0.394346
(3beta,7beta,22E,24xi)-Ergosta-5,22-diene-3,7,8-triol-vs-Alistipes	-0.24418	0.087469	0.151577
(3beta,7beta,22E,24xi)-Ergosta-5,22-diene-3,7,8-triol-vs-Faecalibacterium	-0.52221	0.0001	0.000744
(3beta,7beta,22E,24xi)-Ergosta-5,22-diene-3,7,8-triol-vs-Oscillibacter	-0.35433	0.011581	0.030455
(3beta,7beta,22E,24xi)-Ergosta-5,22-diene-3,7,8-triol-vs-Subdoligranulum	-0.30343	0.032184	0.069485
(3beta,7beta,22E,24xi)-Ergosta-5,22-diene-3,7,8-triol-vs-Bilophila	-0.32139	0.022854	0.052782
(3beta,7beta,22E,24xi)-Ergosta-5,22-diene-3,7,8-triol-vs-Butyricicoccus	-0.43174	0.001744	0.006689
(3beta,7beta,22E,24xi)-Ergosta-5,22-diene-3,7,8-triol-vs-Paraprevotella	-0.11136	0.441363	0.509187

(3beta,7beta,22E,24xi)-Ergosta-5,22-diene-3,7,8-triol-vs-Collinsella	-0.41532	0.002707	0.009474
(3beta,7beta,22E,24xi)-Ergosta-5,22-diene-3,7,8-triol-vs-Parasutterella	-0.22046	0.123944	0.19568
(3beta,7beta,22E,24xi)-Ergosta-5,22-diene-3,7,8-triol-vs-Pyramidobacter	-0.11741	0.416787	0.48534
(3beta,7beta,22E,24xi)-Ergosta-5,22-diene-3,7,8-triol-vs-Intestinimonas	-0.13998	0.332281	0.408974
(3beta,7beta,22E,24xi)-Ergosta-5,22-diene-3,7,8-triol-vs-Pseudoflavonifractor	-0.22122	0.122606	0.194557
(3beta,7beta,22E,24xi)-Ergosta-5,22-diene-3,7,8-triol-vs-Holdemania	-0.23102	0.106492	0.175138
(3beta,7beta,22E,24xi)-Ergosta-5,22-diene-3,7,8-triol-vs-Oribacterium	-0.2363	0.098506	0.166749
(3beta,7beta,22E,24xi)-Ergosta-5,22-diene-3,7,8-triol-vs-Eubacterium	0.190012	0.186274	0.263906
(3beta,7beta,22E,24xi)-Ergosta-5,22-diene-3,7,8-triol-vs-Veillonella	0.621705	1.45E-06	5.93E-05
(3beta,7beta,22E,24xi)-Ergosta-5,22-diene-3,7,8-triol-vs-Lactobacillus	0.51904	0.000112	0.000794
(3beta,7beta,22E,24xi)-Ergosta-5,22-diene-3,7,8-triol-vs-Agathobacter	0.179832	0.211424	0.290752
(3beta,7beta,22E,24xi)-Ergosta-5,22-diene-3,7,8-triol-vs-Enterococcus	0.543721	4.49E-05	0.000423

(3beta,7beta,22E,24xi)-Ergosta-5,22-diene-3,7,8-triol-vs-Blautia	0.258103	0.070342	0.12834
(3beta,7beta,22E,24xi)-Ergosta-5,22-diene-3,7,8-triol-vs-Streptococcus	0.458631	0.000809	0.00359
(3beta,7beta,22E,24xi)-Ergosta-5,22-diene-3,7,8-triol-vs-Erysipelatoclostridium	0.486387	0.000342	0.001819
(3beta,7beta,22E,24xi)-Ergosta-5,22-diene-3,7,8-triol-vs-Lachnospira	-0.02896	0.841786	0.861193
(3beta,7beta,22E,24xi)-Ergosta-5,22-diene-3,7,8-triol-vs-Fusobacterium	0.283073	0.046376	0.09294
(3beta,7beta,22E,24xi)-Ergosta-5,22-diene-3,7,8-triol-vs-Bacillus	0.297575	0.035838	0.076118
(3beta,7beta,22E,24xi)-Ergosta-5,22-diene-3,7,8-triol-vs-Dorea	0.12048	0.404609	0.4736
(3beta,7beta,22E,24xi)-Ergosta-5,22-diene-3,7,8-triol-vs-Tyzzerella	0.191837	0.182002	0.259035
(3beta,7beta,22E,24xi)-Ergosta-5,22-diene-3,7,8-triol-vs-Butyrivibrio	0.192701	0.180004	0.25737
(3beta,7beta,22E,24xi)-Ergosta-5,22-diene-3,7,8-triol-vs-Coprobacillus	0.53575	6.09E-05	0.000529
(3beta,7beta,22E,24xi)-Ergosta-5,22-diene-3,7,8-triol-vs-Selenomonas	0.403313	0.003682	0.012265
(3beta,7beta,22E,24xi)-Ergosta-5,22-diene-3,7,8-triol-vs-Anaerostipes	0.26108	0.067051	0.123516

(3beta,7beta,22E,24xi)-Ergosta-5,22-diene-3,7,8-triol-vs-Peptoclostridium	0.125666	0.384533	0.456771
(3beta,7beta,22E,24xi)-Ergosta-5,22-diene-3,7,8-triol-vs-Dysgonomonas	0.442497	0.001292	0.005282
(3beta,7beta,22E,24xi)-Ergosta-5,22-diene-3,7,8-triol-vs-Capnocytophaga	0.115678	0.423728	0.492499
(3beta,7beta,22E,24xi)-Ergosta-5,22-diene-3,7,8-triol-vs-Flavobacterium	0.181176	0.207973	0.287278
5-acetamido-6-formamido-3-methyluracil-vs-Alistipes	-0.15669	0.277192	0.356718
5-acetamido-6-formamido-3-methyluracil-vs-Faecalibacterium	-0.46074	0.00076	0.003389
5-acetamido-6-formamido-3-methyluracil-vs-Oscillibacter	-0.34867	0.013083	0.033655
5-acetamido-6-formamido-3-methyluracil-vs-Subdoligranulum	-0.31285	0.026963	0.060039
5-acetamido-6-formamido-3-methyluracil-vs-Bilophila	-0.30968	0.028636	0.063253
5-acetamido-6-formamido-3-methyluracil-vs-Butyricicoccus	-0.43328	0.001672	0.006452
5-acetamido-6-formamido-3-methyluracil-vs-Paraprevotella	-0.12288	0.39524	0.465484
5-acetamido-6-formamido-3-methyluracil-vs-Collinsella	-0.43212	0.001726	0.006639
5-acetamido-6-formamido-3-methyluracil-vs-Parasutterella	-0.28643	0.043733	0.089372
5-acetamido-6-formamido-3-methyluracil-vs-Pyramidobacter	-0.16303	0.257974	0.337758

5-acetamido-6-formamido-3-methyluracil-vs-Intestinimonas	-0.16533	0.251213	0.331174
5-acetamido-6-formamido-3-methyluracil-vs-Pseudoflavonifractor	-0.22228	0.120784	0.19265
5-acetamido-6-formamido-3-methyluracil-vs-Holdemania	-0.19952	0.164788	0.240598
5-acetamido-6-formamido-3-methyluracil-vs-Oribacterium	-0.23227	0.104561	0.173454
5-acetamido-6-formamido-3-methyluracil-vs-Eubacterium	0.219784	0.125123	0.196917
5-acetamido-6-formamido-3-methyluracil-vs-Veillonella	0.676062	7.13E-08	0.000035
5-acetamido-6-formamido-3-methyluracil-vs-Lactobacillus	0.567251	1.74E-05	0.000219
5-acetamido-6-formamido-3-methyluracil-vs-Agathobacter	0.242929	0.089152	0.153742
5-acetamido-6-formamido-3-methyluracil-vs-Enterococcus	0.511164	0.000149	0.000985
5-acetamido-6-formamido-3-methyluracil-vs-Blautia	0.27395	0.054213	0.104434
5-acetamido-6-formamido-3-methyluracil-vs-Streptococcus	0.494742	0.00026	0.001482
5-acetamido-6-formamido-3-methyluracil-vs-Erysipelatoclostridium	0.428956	0.001882	0.00713
5-acetamido-6-formamido-3-methyluracil-vs-Lachnospira	-0.02665	0.854237	0.872136
5-acetamido-6-formamido-3-methyluracil-vs-Fusobacterium	0.257431	0.071102	0.129442
5-acetamido-6-formamido-3-methyluracil-vs-Bacillus	0.262521	0.065504	0.122022
5-acetamido-6-formamido-3-methyluracil-vs-Dorea	0.153998	0.285624	0.364736
5-acetamido-6-formamido-3-methyluracil-vs-Tyzzerella	0.209892	0.143475	0.217002

5-acetamido-6-formamido-3-methyluracil-vs-Butyrivibrio	0.20509	0.153072	0.227639
5-acetamido-6-formamido-3-methyluracil-vs-Coprobacillus	0.503193	0.000196	0.001186
5-acetamido-6-formamido-3-methyluracil-vs-Selenomonas	0.371333	0.007931	0.022448
5-acetamido-6-formamido-3-methyluracil-vs-Anaerostipes	0.312845	0.026963	0.060039
5-acetamido-6-formamido-3-methyluracil-vs-Peptoclostridium	0.079472	0.583276	0.635441
5-acetamido-6-formamido-3-methyluracil-vs-Dysgonomonas	0.486771	0.000338	0.001801
5-acetamido-6-formamido-3-methyluracil-vs-Capnocytophaga	0.122305	0.397477	0.467009
5-acetamido-6-formamido-3-methyluracil-vs-Flavobacterium	0.142857	0.322325	0.39949
(2R,3S)-2,3,4-Trihydroxy-3-methylbutyl dihydrogen phosphate-vs-Alistipes	-0.27395	0.054213	0.104434
(2R,3S)-2,3,4-Trihydroxy-3-methylbutyl dihydrogen phosphate-vs-Faecalibacterium	-0.42569	0.002056	0.00766
(2R,3S)-2,3,4-Trihydroxy-3-methylbutyl dihydrogen phosphate-vs-Oscillibacter	-0.33455	0.017565	0.04246
(2R,3S)-2,3,4-Trihydroxy-3-methylbutyl dihydrogen phosphate-vs-Subdoligranulum	-0.28624	0.043881	0.08938
(2R,3S)-2,3,4-Trihydroxy-3-methylbutyl dihydrogen phosphate-vs-Bilophila	-0.26655	0.061322	0.115182

(2R,3S)-2,3,4-Trihydroxy-3-methylbutyl dihydrogen phosphate-vs-Butyricicoccus	-0.34118	0.015321	0.038225
(2R,3S)-2,3,4-Trihydroxy-3-methylbutyl dihydrogen phosphate-vs-Paraprevotella	-0.07006	0.628766	0.675523
(2R,3S)-2,3,4-Trihydroxy-3-methylbutyl dihydrogen phosphate-vs-Collinsella	-0.44125	0.001339	0.005436
(2R,3S)-2,3,4-Trihydroxy-3-methylbutyl dihydrogen phosphate-vs-Parasutterella	-0.3091	0.028949	0.063832
(2R,3S)-2,3,4-Trihydroxy-3-methylbutyl dihydrogen phosphate-vs-Pyramidobacter	-0.14843	0.303618	0.382796
(2R,3S)-2,3,4-Trihydroxy-3-methylbutyl dihydrogen phosphate-vs-Intestinimonas	-0.17253	0.23086	0.309767
(2R,3S)-2,3,4-Trihydroxy-3-methylbutyl dihydrogen phosphate-vs-Pseudoflavonifractor	-0.26867	0.059219	0.111738
(2R,3S)-2,3,4-Trihydroxy-3-methylbutyl dihydrogen phosphate-vs-Holdemania	-0.12826	0.374721	0.448114
(2R,3S)-2,3,4-Trihydroxy-3-methylbutyl dihydrogen phosphate-vs-Oribacterium	-0.15688	0.276597	0.356321
(2R,3S)-2,3,4-Trihydroxy-3-methylbutyl dihydrogen phosphate-vs-Eubacterium	0.227275	0.112449	0.181807
(2R,3S)-2,3,4-Trihydroxy-3-methylbutyl dihydrogen phosphate-vs-Veillonella	0.630252	9.36E-07	5.32E-05
(2R,3S)-2,3,4-Trihydroxy-3-methylbutyl dihydrogen phosphate-vs-Lactobacillus	0.500408	0.000215	0.001278

(2R,3S)-2,3,4-Trihydroxy-3-methylbutyl dihydrogen phosphate-vs-Agathobacter	0.228331	0.110744	0.179869
(2R,3S)-2,3,4-Trihydroxy-3-methylbutyl dihydrogen phosphate-vs-Enterococcus	0.570228	1.54E-05	0.000205
(2R,3S)-2,3,4-Trihydroxy-3-methylbutyl dihydrogen phosphate-vs-Blautia	0.246675	0.08418	0.14773
(2R,3S)-2,3,4-Trihydroxy-3-methylbutyl dihydrogen phosphate-vs-Streptococcus	0.564082	1.99E-05	0.000241
(2R,3S)-2,3,4-Trihydroxy-3-methylbutyl dihydrogen phosphate-vs-Erysipelatoclostridium	0.521921	0.000101	0.000748
(2R,3S)-2,3,4-Trihydroxy-3-methylbutyl dihydrogen phosphate-vs-Lachnospira	0.173589	0.227974	0.306888
(2R,3S)-2,3,4-Trihydroxy-3-methylbutyl dihydrogen phosphate-vs-Fusobacterium	0.427611	0.001952	0.007316
(2R,3S)-2,3,4-Trihydroxy-3-methylbutyl dihydrogen phosphate-vs-Bacillus	0.405234	0.003508	0.011795
(2R,3S)-2,3,4-Trihydroxy-3-methylbutyl dihydrogen phosphate-vs-Dorea	0.194526	0.175836	0.252428
(2R,3S)-2,3,4-Trihydroxy-3-methylbutyl dihydrogen phosphate-vs-Tyzzerella	0.166002	0.249263	0.329303
(2R,3S)-2,3,4-Trihydroxy-3-methylbutyl dihydrogen phosphate-vs-Butyrivibrio	0.249172	0.080987	0.14365
(2R,3S)-2,3,4-Trihydroxy-3-methylbutyl dihydrogen phosphate-vs-Coprobacillus	0.596735	4.79E-06	9.76E-05

(2R,3S)-2,3,4-Trihydroxy-3-methylbutyl dihydrogen phosphate-vs-Selenomonas	0.509916	0.000155	0.001002
(2R,3S)-2,3,4-Trihydroxy-3-methylbutyl dihydrogen phosphate-vs-Anaerostipes	0.293157	0.038815	0.081055
(2R,3S)-2,3,4-Trihydroxy-3-methylbutyl dihydrogen phosphate-vs-Peptoclostridium	0.10982	0.447731	0.514621
(2R,3S)-2,3,4-Trihydroxy-3-methylbutyl dihydrogen phosphate-vs-Dysgonomonas	0.4109	0.003035	0.010476
(2R,3S)-2,3,4-Trihydroxy-3-methylbutyl dihydrogen phosphate-vs-Capnocytophaga	0.243697	0.088113	0.152374
(2R,3S)-2,3,4-Trihydroxy-3-methylbutyl dihydrogen phosphate-vs-Flavobacterium	0.2291	0.109516	0.178575
3-(1H-Imidazol-4-yl)-2-oxopropyl dihydrogen phosphate-vs-Alistipes	-0.24473	0.086739	0.150837
3-(1H-Imidazol-4-yl)-2-oxopropyl dihydrogen phosphate-vs-Faecalibacterium	-0.52703	8.42E-05	0.000662
3-(1H-Imidazol-4-yl)-2-oxopropyl dihydrogen phosphate-vs-Oscillibacter	-0.39634	0.00438	0.013955
3-(1H-Imidazol-4-yl)-2-oxopropyl dihydrogen phosphate-vs-Subdoligranulum	-0.30936	0.028805	0.06357
3-(1H-Imidazol-4-yl)-2-oxopropyl dihydrogen phosphate-vs-Bilophila	-0.34402	0.014437	0.036459
3-(1H-Imidazol-4-yl)-2-oxopropyl dihydrogen phosphate-vs-Butyricicoccus	-0.50968	0.000156	0.001007

3-(1H-Imidazol-4-yl)-2-oxopropyl dihydrogen phosphate-vs-Paraprevotella	-0.14565	0.312845	0.391057
3-(1H-Imidazol-4-yl)-2-oxopropyl dihydrogen phosphate-vs-Collinsella	-0.424	0.002151	0.007976
3-(1H-Imidazol-4-yl)-2-oxopropyl dihydrogen phosphate-vs-Parasutterella	-0.24874	0.081534	0.144208
3-(1H-Imidazol-4-yl)-2-oxopropyl dihydrogen phosphate-vs-Pyramidobacter	-0.21414	0.13536	0.208278
3-(1H-Imidazol-4-yl)-2-oxopropyl dihydrogen phosphate-vs-Intestinimonas	-0.23437	0.101375	0.170329
3-(1H-Imidazol-4-yl)-2-oxopropyl dihydrogen phosphate-vs-Pseudoflavonifractor	-0.28556	0.04441	0.090162
3-(1H-Imidazol-4-yl)-2-oxopropyl dihydrogen phosphate-vs-Holdemania	-0.13465	0.351202	0.427602
3-(1H-Imidazol-4-yl)-2-oxopropyl dihydrogen phosphate-vs-Oribacterium	-0.2008	0.162035	0.237556
3-(1H-Imidazol-4-yl)-2-oxopropyl dihydrogen phosphate-vs-Eubacterium	0.249498	0.080578	0.143332
3-(1H-Imidazol-4-yl)-2-oxopropyl dihydrogen phosphate-vs-Veillonella	0.644486	4.4E-07	4.08E-05
3-(1H-Imidazol-4-yl)-2-oxopropyl dihydrogen phosphate-vs-Lactobacillus	0.572473	0.000014	0.000193
3-(1H-Imidazol-4-yl)-2-oxopropyl dihydrogen phosphate-vs-Agathobacter	0.249444	0.080646	0.143351

3-(1H-Imidazol-4-yl)-2-oxopropyl dihydrogen phosphate-vs-Enterococcus	0.569707	1.57E-05	0.000207
3-(1H-Imidazol-4-yl)-2-oxopropyl dihydrogen phosphate-vs-Blautia	0.340816	0.015436	0.038475
3-(1H-Imidazol-4-yl)-2-oxopropyl dihydrogen phosphate-vs-Streptococcus	0.585813	7.85E-06	0.00013
3-(1H-Imidazol-4-yl)-2-oxopropyl dihydrogen phosphate-vs-Erysipelatoclostridium	0.545197	4.24E-05	0.000408
3-(1H-Imidazol-4-yl)-2-oxopropyl dihydrogen phosphate-vs-Lachnospira	0.195379	0.173911	0.250243
3-(1H-Imidazol-4-yl)-2-oxopropyl dihydrogen phosphate-vs-Fusobacterium	0.484354	0.000365	0.001922
3-(1H-Imidazol-4-yl)-2-oxopropyl dihydrogen phosphate-vs-Bacillus	0.378883	0.006661	0.019497
3-(1H-Imidazol-4-yl)-2-oxopropyl dihydrogen phosphate-vs-Dorea	0.233175	0.103176	0.172182
3-(1H-Imidazol-4-yl)-2-oxopropyl dihydrogen phosphate-vs-Tyzzerella	0.216691	0.130661	0.202679
3-(1H-Imidazol-4-yl)-2-oxopropyl dihydrogen phosphate-vs-Butyrivibrio	0.265386	0.062511	0.116973
3-(1H-Imidazol-4-yl)-2-oxopropyl dihydrogen phosphate-vs-Coprobacillus	0.58809	7.09E-06	0.000121
3-(1H-Imidazol-4-yl)-2-oxopropyl dihydrogen phosphate-vs-Selenomonas	0.46429	0.000683	0.003107

3-(1H-Imidazol-4-yl)-2-oxopropyl dihydrogen phosphate-vs-Anaerostipes	0.329211	0.01957	0.046449
3-(1H-Imidazol-4-yl)-2-oxopropyl dihydrogen phosphate-vs-Peptoclostridium	0.134049	0.35336	0.429298
3-(1H-Imidazol-4-yl)-2-oxopropyl dihydrogen phosphate-vs-Dysgonomonas	0.489506	0.000309	0.001686
3-(1H-Imidazol-4-yl)-2-oxopropyl dihydrogen phosphate-vs-Capnocytophaga	0.167669	0.244474	0.32401
3-(1H-Imidazol-4-yl)-2-oxopropyl dihydrogen phosphate-vs-Flavobacterium	0.192017	0.181584	0.258587
3,4-Dihydroxyphenylglycolic acid-vs-Alistipes	-0.13527	0.348951	0.425487
3,4-Dihydroxyphenylglycolic acid-vs-Faecalibacterium	-0.45719	0.000844	0.003714
3,4-Dihydroxyphenylglycolic acid-vs-Oscillibacter	-0.23534	0.099923	0.168573
3,4-Dihydroxyphenylglycolic acid-vs-Subdoligranulum	-0.18396	0.20095	0.27944
3,4-Dihydroxyphenylglycolic acid-vs-Bilophila	-0.20672	0.149757	0.223915
3,4-Dihydroxyphenylglycolic acid-vs-Butyricicoccus	-0.39697	0.004312	0.01381
3,4-Dihydroxyphenylglycolic acid-vs-Paraprevotella	-0.07409	0.60909	0.658368
3,4-Dihydroxyphenylglycolic acid-vs-Collinsella	-0.43894	0.001428	0.005707
3,4-Dihydroxyphenylglycolic acid-vs-Parasutterella	-0.29248	0.039285	0.081694
3,4-Dihydroxyphenylglycolic acid-vs-Pyramidobacter	-0.08984	0.534941	0.591338
3,4-Dihydroxyphenylglycolic acid-vs-Intestinimonas	-0.06785	0.639652	0.683965
3,4-Dihydroxyphenylglycolic acid-vs-Pseudoflavonifractor	-0.12941	0.370408	0.44424
3,4-Dihydroxyphenylglycolic acid-vs-Holdemania	-0.14776	0.305838	0.385204
3,4-Dihydroxyphenylglycolic acid-vs-Oribacterium	-0.20884	0.145547	0.219202

3,4-Dihydroxyphenylglycolic acid-vs-Eubacterium	0.164562	0.253453	0.333067
3,4-Dihydroxyphenylglycolic acid-vs-Veillonella	0.667323	1.21E-07	0.000035
3,4-Dihydroxyphenylglycolic acid-vs-Lactobacillus	0.564082	1.99E-05	0.000241
3,4-Dihydroxyphenylglycolic acid-vs-Agathobacter	0.17455	0.225371	0.304539
3,4-Dihydroxyphenylglycolic acid-vs-Enterococcus	0.490324	0.000301	0.001661
3,4-Dihydroxyphenylglycolic acid-vs-Blautia	0.299784	0.034422	0.073701
3,4-Dihydroxyphenylglycolic acid-vs-Streptococcus	0.510492	0.000152	0.000997
3,4-Dihydroxyphenylglycolic acid-vs-Erysipelatoclostridium	0.488307	0.000321	0.001724
3,4-Dihydroxyphenylglycolic acid-vs-Lachnospira	0.000144	0.999208	0.999208
3,4-Dihydroxyphenylglycolic acid-vs-Fusobacterium	0.481104	0.000405	0.00208
3,4-Dihydroxyphenylglycolic acid-vs-Bacillus	0.394766	0.004553	0.014305
3,4-Dihydroxyphenylglycolic acid-vs-Dorea	0.186939	0.193629	0.272001
3,4-Dihydroxyphenylglycolic acid-vs-Tyzzerella	0.197791	0.168554	0.244373
3,4-Dihydroxyphenylglycolic acid-vs-Butyrivibrio	0.233229	0.103094	0.17217
3,4-Dihydroxyphenylglycolic acid-vs-Coprobacillus	0.551309	3.33E-05	0.000344
3,4-Dihydroxyphenylglycolic acid-vs-Selenomonas	0.401777	0.003827	0.012579
3,4-Dihydroxyphenylglycolic acid-vs-Anaerostipes	0.270684	0.057266	0.108962
3,4-Dihydroxyphenylglycolic acid-vs-Peptoclostridium	0.102521	0.478655	0.541647
3,4-Dihydroxyphenylglycolic acid-vs-Dysgonomonas	0.532581	6.86E-05	0.00057
3,4-Dihydroxyphenylglycolic acid-vs-Capnocytophaga	0.036351	0.802109	0.827414
3,4-Dihydroxyphenylglycolic acid-vs-Flavobacterium	0.114334	0.429171	0.49743
Sphingosine 1-phosphate-vs-Alistipes	-0.10723	0.458589	0.523229
Sphingosine 1-phosphate-vs-Faecalibacterium	-0.50454	0.000187	0.001147
Sphingosine 1-phosphate-vs-Oscillibacter	-0.19866	0.166663	0.242908

Sphingosine 1-phosphate-vs-Subdoligranulum	-0.18406	0.200711	0.279264
Sphingosine 1-phosphate-vs-Bilophila	-0.13191	0.361167	0.436103
Sphingosine 1-phosphate-vs-Butyricicoccus	-0.33167	0.018624	0.044459
Sphingosine 1-phosphate-vs-Paraprevotella	-0.06689	0.644409	0.688755
Sphingosine 1-phosphate-vs-Collinsella	-0.39765	0.004241	0.013635
Sphingosine 1-phosphate-vs-Parasutterella	-0.33743	0.016557	0.040576
Sphingosine 1-phosphate-vs-Pyramidobacter	0.017143	0.90594	0.919633
Sphingosine 1-phosphate-vs-Intestinimonas	0.022905	0.874545	0.891039
Sphingosine 1-phosphate-vs-Pseudoflavonifractor	-0.07457	0.606765	0.656426
Sphingosine 1-phosphate-vs-Holdemania	-0.1442	0.317744	0.395985
Sphingosine 1-phosphate-vs-Oribacterium	-0.2219	0.121444	0.193579
Sphingosine 1-phosphate-vs-Eubacterium	0.138631	0.336992	0.413544
Sphingosine 1-phosphate-vs-Veillonella	0.627179	1.1E-06	5.56E-05
Sphingosine 1-phosphate-vs-Lactobacillus	0.5103	0.000153	0.000997
Sphingosine 1-phosphate-vs-Agathobacter	0.073037	0.614217	0.66247
Sphingosine 1-phosphate-vs-Enterococcus	0.501561	0.000207	0.001238
Sphingosine 1-phosphate-vs-Blautia	0.230828	0.106792	0.175398
Sphingosine 1-phosphate-vs-Streptococcus	0.463529	0.000699	0.003162
Sphingosine 1-phosphate-vs-Erysipelatoclostridium	0.469388	0.000584	0.002782
Sphingosine 1-phosphate-vs-Lachnospira	-0.09724	0.50172	0.562369
Sphingosine 1-phosphate-vs-Fusobacterium	0.454982	0.000901	0.003902
Sphingosine 1-phosphate-vs-Bacillus	0.396399	0.004374	0.013954
Sphingosine 1-phosphate-vs-Dorea	0.110876	0.443348	0.510765
Sphingosine 1-phosphate-vs-Tyzzerella	0.192029	0.181557	0.258587
Sphingosine 1-phosphate-vs-Butyrivibrio	0.204418	0.154453	0.22928

Sphingosine 1-phosphate-vs-Coprobacillus	0.509052	0.00016	0.001017
Sphingosine 1-phosphate-vs-Selenomonas	0.429724	0.001843	0.007003
Sphingosine 1-phosphate-vs-Anaerostipes	0.192413	0.180668	0.258023
Sphingosine 1-phosphate-vs-Peptoclostridium	0.053349	0.712905	0.746552
Sphingosine 1-phosphate-vs-Dysgonomonas	0.561681	0.000022	0.000257
Sphingosine 1-phosphate-vs-Capnocytophaga	0.122209	0.397851	0.467009
Sphingosine 1-phosphate-vs-Flavobacterium	0.173685	0.227712	0.306702
1,3,4-Trihydroxy-5-oxocyclohexanecarboxylic acid-vs-Alistipes	-0.21152	0.140315	0.213523
1,3,4-Trihydroxy-5-oxocyclohexanecarboxylic acid-vs-Faecalibacterium	-0.46583	0.000651	0.003015
1,3,4-Trihydroxy-5-oxocyclohexanecarboxylic acid-vs-Oscillibacter	-0.31073	0.028069	0.062112
1,3,4-Trihydroxy-5-oxocyclohexanecarboxylic acid-vs-Subdoligranulum	-0.24648	0.084429	0.147855
1,3,4-Trihydroxy-5-oxocyclohexanecarboxylic acid-vs-Bilophila	-0.15649	0.277789	0.357116
1,3,4-Trihydroxy-5-oxocyclohexanecarboxylic acid-vs-Butyricicoccus	-0.5102	0.000154	0.000997
1,3,4-Trihydroxy-5-oxocyclohexanecarboxylic acid-vs-Paraprevotella	-0.12	0.406498	0.475587
1,3,4-Trihydroxy-5-oxocyclohexanecarboxylic acid-vs-Collinsella	-0.37489	0.00731	0.021048
1,3,4-Trihydroxy-5-oxocyclohexanecarboxylic acid-vs-Parasutterella	-0.21796	0.128368	0.200247

1,3,4-Trihydroxy-5-oxocyclohexanecarboxylic acid-vs-Pyramidobacter	-0.18406	0.200711	0.279264
1,3,4-Trihydroxy-5-oxocyclohexanecarboxylic acid-vs-Intestinimonas	-0.16331	0.257122	0.336998
1,3,4-Trihydroxy-5-oxocyclohexanecarboxylic acid-vs-Pseudoflavonifractor	-0.24706	0.083682	0.146961
1,3,4-Trihydroxy-5-oxocyclohexanecarboxylic acid-vs-Holdemania	-0.08783	0.544184	0.599157
1,3,4-Trihydroxy-5-oxocyclohexanecarboxylic acid-vs-Oribacterium	-0.14218	0.324631	0.401747
1,3,4-Trihydroxy-5-oxocyclohexanecarboxylic acid-vs-Eubacterium	0.267803	0.060072	0.113262
1,3,4-Trihydroxy-5-oxocyclohexanecarboxylic acid-vs-Veillonella	0.57455	1.28E-05	0.00018
1,3,4-Trihydroxy-5-oxocyclohexanecarboxylic acid-vs-Lactobacillus	0.534118	6.47E-05	0.000553
1,3,4-Trihydroxy-5-oxocyclohexanecarboxylic acid-vs-Agathobacter	0.276447	0.051969	0.100972
1,3,4-Trihydroxy-5-oxocyclohexanecarboxylic acid-vs-Enterococcus	0.608547	2.75E-06	7.77E-05
1,3,4-Trihydroxy-5-oxocyclohexanecarboxylic acid-vs-Blautia	0.337431	0.016557	0.040576
1,3,4-Trihydroxy-5-oxocyclohexanecarboxylic acid-vs-Streptococcus	0.530756	7.34E-05	0.000594

1,3,4-Trihydroxy-5-oxocyclohexanecarboxylic acid-vs-Erysipelatoclostridium	0.575702	1.22E-05	0.000173
1,3,4-Trihydroxy-5-oxocyclohexanecarboxylic acid-vs-Lachnospira	0.228908	0.109822	0.178839
1,3,4-Trihydroxy-5-oxocyclohexanecarboxylic acid-vs-Fusobacterium	0.417623	0.002548	0.009046
1,3,4-Trihydroxy-5-oxocyclohexanecarboxylic acid-vs-Bacillus	0.368643	0.008432	0.023596
1,3,4-Trihydroxy-5-oxocyclohexanecarboxylic acid-vs-Dorea	0.312653	0.027062	0.060152
1,3,4-Trihydroxy-5-oxocyclohexanecarboxylic acid-vs-Tyzzerella	0.306026	0.030668	0.066851
1,3,4-Trihydroxy-5-oxocyclohexanecarboxylic acid-vs-Butyrivibrio	0.279232	0.049555	0.097348
1,3,4-Trihydroxy-5-oxocyclohexanecarboxylic acid-vs-Coprobacillus	0.596543	4.83E-06	9.77E-05
1,3,4-Trihydroxy-5-oxocyclohexanecarboxylic acid-vs-Selenomonas	0.468139	0.000607	0.002858
1,3,4-Trihydroxy-5-oxocyclohexanecarboxylic acid-vs-Anaerostipes	0.401008	0.003901	0.012756
1,3,4-Trihydroxy-5-oxocyclohexanecarboxylic acid-vs-Peptoclostridium	0.210756	0.141796	0.214986
1,3,4-Trihydroxy-5-oxocyclohexanecarboxylic acid-vs-Dysgonomonas	0.39563	0.004458	0.01413

1,3,4-Trihydroxy-5-oxocyclohexanecarboxylic acid-vs-Capnocytophaga	0.190492	0.185143	0.262603
1,3,4-Trihydroxy-5-oxocyclohexanecarboxylic acid-vs-Flavobacterium	0.297287	0.036027	0.076453
5-(3-Hydroxy-3,7,11,15-tetramethylhexadecyl)-2,3-dimethyl-1,4-benzoquinone-vs-Alistipes	-0.18463	0.19928	0.278052
5-(3-Hydroxy-3,7,11,15-tetramethylhexadecyl)-2,3-dimethyl-1,4-benzoquinone-vs-Faecalibacterium	-0.49858	0.000229	0.001337
5-(3-Hydroxy-3,7,11,15-tetramethylhexadecyl)-2,3-dimethyl-1,4-benzoquinone-vs-Oscillibacter	-0.3067	0.030285	0.066132
5-(3-Hydroxy-3,7,11,15-tetramethylhexadecyl)-2,3-dimethyl-1,4-benzoquinone-vs-Subdoligranulum	-0.25397	0.075117	0.135461
5-(3-Hydroxy-3,7,11,15-tetramethylhexadecyl)-2,3-dimethyl-1,4-benzoquinone-vs-Bilophila	-0.33753	0.016524	0.040576
5-(3-Hydroxy-3,7,11,15-tetramethylhexadecyl)-2,3-dimethyl-1,4-benzoquinone-vs-Butyricicoccus	-0.4013	0.003873	0.012698
5-(3-Hydroxy-3,7,11,15-tetramethylhexadecyl)-2,3-dimethyl-1,4-benzoquinone-vs-Paraprevotella	-0.11164	0.440175	0.508288
5-(3-Hydroxy-3,7,11,15-tetramethylhexadecyl)-2,3-dimethyl-1,4-benzoquinone-vs-Collinsella	-0.4206	0.002356	0.008596
5-(3-Hydroxy-3,7,11,15-tetramethylhexadecyl)-2,3-dimethyl-1,4-benzoquinone-vs-Parasutterella	-0.26252	0.065504	0.122022
5-(3-Hydroxy-3,7,11,15-tetramethylhexadecyl)-2,3-dimethyl-1,4-benzoquinone-vs-Pyramidobacter	-0.05978	0.680037	0.718797

5-(3-Hydroxy-3,7,11,15-tetramethylhexadecyl)-2,3-dimethyl-1,4-benzoquinone-vs-Intestinimonas	-0.11241	0.437015	0.505108
5-(3-Hydroxy-3,7,11,15-tetramethylhexadecyl)-2,3-dimethyl-1,4-benzoquinone-vs-Pseudoflavonifractor	-0.15966	0.268051	0.347798
5-(3-Hydroxy-3,7,11,15-tetramethylhexadecyl)-2,3-dimethyl-1,4-benzoquinone-vs-Holdemania	-0.19635	0.171739	0.247835
5-(3-Hydroxy-3,7,11,15-tetramethylhexadecyl)-2,3-dimethyl-1,4-benzoquinone-vs-Oribacterium	-0.19453	0.175836	0.252428
5-(3-Hydroxy-3,7,11,15-tetramethylhexadecyl)-2,3-dimethyl-1,4-benzoquinone-vs-Eubacterium	0.221609	0.121941	0.193997
5-(3-Hydroxy-3,7,11,15-tetramethylhexadecyl)-2,3-dimethyl-1,4-benzoquinone-vs-Veillonella	0.646291	3.99E-07	4.07E-05
5-(3-Hydroxy-3,7,11,15-tetramethylhexadecyl)-2,3-dimethyl-1,4-benzoquinone-vs-Lactobacillus	0.557839	2.57E-05	0.000281
5-(3-Hydroxy-3,7,11,15-tetramethylhexadecyl)-2,3-dimethyl-1,4-benzoquinone-vs-Agathobacter	0.234766	0.10078	0.169674
5-(3-Hydroxy-3,7,11,15-tetramethylhexadecyl)-2,3-dimethyl-1,4-benzoquinone-vs-Enterococcus	0.541321	4.92E-05	0.000448
5-(3-Hydroxy-3,7,11,15-tetramethylhexadecyl)-2,3-dimethyl-1,4-benzoquinone-vs-Blautia	0.198175	0.167712	0.243579
5-(3-Hydroxy-3,7,11,15-tetramethylhexadecyl)-2,3-dimethyl-1,4-benzoquinone-vs-Streptococcus	0.511261	0.000148	0.000984
5-(3-Hydroxy-3,7,11,15-tetramethylhexadecyl)-2,3-dimethyl-1,4-benzoquinone-vs-Erysipelatoclostridium	0.436735	0.001519	0.005984

5-(3-Hydroxy-3,7,11,15-tetramethylhexadecyl)-2,3-dimethyl-1,4-benzoquinone-vs-Lachnospira	-0.01945	0.893362	0.908349
5-(3-Hydroxy-3,7,11,15-tetramethylhexadecyl)-2,3-dimethyl-1,4-benzoquinone-vs-Fusobacterium	0.284802	0.045001	0.090695
5-(3-Hydroxy-3,7,11,15-tetramethylhexadecyl)-2,3-dimethyl-1,4-benzoquinone-vs-Bacillus	0.283169	0.046299	0.09286
5-(3-Hydroxy-3,7,11,15-tetramethylhexadecyl)-2,3-dimethyl-1,4-benzoquinone-vs-Dorea	0.143433	0.320357	0.397646
5-(3-Hydroxy-3,7,11,15-tetramethylhexadecyl)-2,3-dimethyl-1,4-benzoquinone-vs-Tyzzerella	0.17042	0.236709	0.315909
5-(3-Hydroxy-3,7,11,15-tetramethylhexadecyl)-2,3-dimethyl-1,4-benzoquinone-vs-Butyrivibrio	0.227467	0.112138	0.181539
5-(3-Hydroxy-3,7,11,15-tetramethylhexadecyl)-2,3-dimethyl-1,4-benzoquinone-vs-Coprobacillus	0.482449	0.000388	0.002013
5-(3-Hydroxy-3,7,11,15-tetramethylhexadecyl)-2,3-dimethyl-1,4-benzoquinone-vs-Selenomonas	0.363842	0.009394	0.025852
5-(3-Hydroxy-3,7,11,15-tetramethylhexadecyl)-2,3-dimethyl-1,4-benzoquinone-vs-Anaerostipes	0.261657	0.066429	0.122824
5-(3-Hydroxy-3,7,11,15-tetramethylhexadecyl)-2,3-dimethyl-1,4-benzoquinone-vs-Peptoclostridium	0.048643	0.737278	0.767226
5-(3-Hydroxy-3,7,11,15-tetramethylhexadecyl)-2,3-dimethyl-1,4-benzoquinone-vs-Dysgonomonas	0.5006	0.000214	0.001273
5-(3-Hydroxy-3,7,11,15-tetramethylhexadecyl)-2,3-dimethyl-1,4-benzoquinone-vs-Capnocytophaga	0.212869	0.137753	0.210656

5-(3-Hydroxy-3,7,11,15-tetramethylhexadecyl)-2,3-dimethyl-1,4-benzoquinone-vs-Flavobacterium	0.180312	0.210187	0.289371
Naphthalen-2-amine-vs-Alistipes	-0.16571	0.250098	0.33023
Naphthalen-2-amine-vs-Faecalibacterium	-0.43011	0.001824	0.006941
Naphthalen-2-amine-vs-Oscillibacter	-0.39861	0.004142	0.013366
Naphthalen-2-amine-vs-Subdoligranulum	-0.28788	0.042638	0.087639
Naphthalen-2-amine-vs-Bilophila	-0.2291	0.109516	0.178575
Naphthalen-2-amine-vs-Butyricicoccus	-0.51904	0.000112	0.000794
Naphthalen-2-amine-vs-Paraprevotella	-0.10819	0.454551	0.520055
Naphthalen-2-amine-vs-Collinsella	-0.57801	0.000011	0.000163
Naphthalen-2-amine-vs-Parasutterella	-0.35136	0.012351	0.032138
Naphthalen-2-amine-vs-Pyramidobacter	-0.25647	0.0722	0.13077
Naphthalen-2-amine-vs-Intestinimonas	-0.20989	0.143475	0.217002
Naphthalen-2-amine-vs-Pseudoflavonifractor	-0.34588	0.01388	0.035376
Naphthalen-2-amine-vs-Holdemania	-0.24303	0.089021	0.15373
Naphthalen-2-amine-vs-Oribacterium	-0.21325	0.137027	0.209804
Naphthalen-2-amine-vs-Eubacterium	0.248691	0.081594	0.144211
Naphthalen-2-amine-vs-Veillonella	0.672797	8.7E-08	0.000035
Naphthalen-2-amine-vs-Lactobacillus	0.472749	0.000527	0.002572
Naphthalen-2-amine-vs-Agathobacter	0.299592	0.034543	0.07381
Naphthalen-2-amine-vs-Enterococcus	0.510108	0.000154	0.000998
Naphthalen-2-amine-vs-Blautia	0.301897	0.033111	0.071116
Naphthalen-2-amine-vs-Streptococcus	0.599712	4.18E-06	9.26E-05
Naphthalen-2-amine-vs-Erysipelatoclostridium	0.476783	0.000464	0.002327
Naphthalen-2-amine-vs-Lachnospira	0.066699	0.645362	0.689181

Naphthalen-2-amine-vs-Fusobacterium	0.376903	0.006976	0.020252
Naphthalen-2-amine-vs-Bacillus	0.269628	0.058282	0.110474
Naphthalen-2-amine-vs-Dorea	0.220264	0.12428	0.196086
Naphthalen-2-amine-vs-Tyzzerella	0.252917	0.076379	0.137338
Naphthalen-2-amine-vs-Butyrivibrio	0.237935	0.096134	0.163849
Naphthalen-2-amine-vs-Coprobacillus	0.558896	2.46E-05	0.000277
Naphthalen-2-amine-vs-Selenomonas	0.320624	0.023201	0.053334
Naphthalen-2-amine-vs-Anaerostipes	0.380168	0.006464	0.019099
Naphthalen-2-amine-vs-Peptoclostridium	0.138151	0.338685	0.415006
Naphthalen-2-amine-vs-Dysgonomonas	0.465258	0.000663	0.003051
Naphthalen-2-amine-vs-Capnocytophaga	0.225642	0.115125	0.185408
Naphthalen-2-amine-vs-Flavobacterium	0.061897	0.669368	0.70933
Hecogenin-vs-Alistipes	-0.13671	0.343794	0.420024
Hecogenin-vs-Faecalibacterium	-0.47169	0.000544	0.002642
Hecogenin-vs-Oscillibacter	-0.22833	0.110744	0.179869
Hecogenin-vs-Subdoligranulum	-0.1393	0.334631	0.411256
Hecogenin-vs-Bilophila	-0.11962	0.408013	0.476687
Hecogenin-vs-Butyricicoccus	-0.42291	0.002215	0.008168
Hecogenin-vs-Paraprevotella	-0.09148	0.527515	0.584951
Hecogenin-vs-Collinsella	-0.3988	0.004122	0.013337
Hecogenin-vs-Parasutterella	-0.27693	0.051546	0.100386
Hecogenin-vs-Pyramidobacter	-0.04788	0.741282	0.770103
Hecogenin-vs-Intestinimonas	-0.0327	0.821634	0.844401
Hecogenin-vs-Pseudoflavonifractor	-0.09561	0.508962	0.568692
Hecogenin-vs-Holdemania	-0.11001	0.446932	0.514417

Hecogenin-vs-Oribacterium	-0.20134	0.16088	0.236281
Hecogenin-vs-Eubacterium	0.157071	0.276001	0.356108
Hecogenin-vs-Veillonella	0.639184	5.86E-07	4.55E-05
Hecogenin-vs-Lactobacillus	0.522593	0.000099	0.000739
Hecogenin-vs-Agathobacter	0.150828	0.295774	0.375
Hecogenin-vs-Enterococcus	0.53018	0.000075	0.000601
Hecogenin-vs-Blautia	0.31515	0.025799	0.057913
Hecogenin-vs-Streptococcus	0.492245	0.000282	0.001577
Hecogenin-vs-Erysipelatoclostridium	0.483986	0.000369	0.001941
Hecogenin-vs-Lachnospira	-0.04797	0.740782	0.769905
Hecogenin-vs-Fusobacterium	0.466699	0.000635	0.002959
Hecogenin-vs-Bacillus	0.411573	0.002983	0.010324
Hecogenin-vs-Dorea	0.190972	0.184017	0.261603
Hecogenin-vs-Tyzzerella	0.25407	0.075003	0.135452
Hecogenin-vs-Butyrivibrio	0.271837	0.056173	0.107625
Hecogenin-vs-Coprobacillus	0.558607	2.49E-05	0.000277
Hecogenin-vs-Selenomonas	0.448932	0.001075	0.004536
Hecogenin-vs-Anaerostipes	0.245906	0.085181	0.148858
Hecogenin-vs-Peptoclostridium	0.155822	0.279884	0.358882
Hecogenin-vs-Dysgonomonas	0.481777	0.000396	0.002044
Hecogenin-vs-Capnocytophaga	0.04922	0.734279	0.764746
Hecogenin-vs-Flavobacterium	0.136423	0.344822	0.421072
Methyl alpha-aspartylphenylalaninate-vs-Alistipes	-0.11942	0.408772	0.477348
Methyl alpha-aspartylphenylalaninate-vs-Faecalibacterium	-0.52605	8.73E-05	0.000682
Methyl alpha-aspartylphenylalaninate-vs-Oscillibacter	-0.23121	0.106193	0.174878

Methyl alpha-aspartylphenylalaninate-vs-Subdoligranulum	-0.22411	0.117686	0.188435
Methyl alpha-aspartylphenylalaninate-vs-Bilophila	-0.3284	0.01989	0.047073
Methyl alpha-aspartylphenylalaninate-vs-Butyricicoccus	-0.40072	0.003929	0.012814
Methyl alpha-aspartylphenylalaninate-vs-Paraprevotella	-0.0909	0.53013	0.587065
Methyl alpha-aspartylphenylalaninate-vs-Collinsella	-0.36442	0.009274	0.025606
Methyl alpha-aspartylphenylalaninate-vs-Parasutterella	-0.2195	0.125632	0.197466
Methyl alpha-aspartylphenylalaninate-vs-Pyramidobacter	-0.06199	0.668884	0.70912
Methyl alpha-aspartylphenylalaninate-vs-Intestinimonas	-0.10761	0.456971	0.521863
Methyl alpha-aspartylphenylalaninate-vs-Pseudoflavonifractor	-0.12595	0.383436	0.455903
Methyl alpha-aspartylphenylalaninate-vs-Holdemania	-0.10771	0.456568	0.521863
Methyl alpha-aspartylphenylalaninate-vs-Oribacterium	-0.12595	0.383436	0.455903
Methyl alpha-aspartylphenylalaninate-vs-Eubacterium	0.357311	0.010854	0.029002
Methyl alpha-aspartylphenylalaninate-vs-Veillonella	0.580312	9.99E-06	0.000152
Methyl alpha-aspartylphenylalaninate-vs-Lactobacillus	0.615558	1.96E-06	6.49E-05
Methyl alpha-aspartylphenylalaninate-vs-Agathobacter	0.302281	0.032877	0.070675
Methyl alpha-aspartylphenylalaninate-vs-Enterococcus	0.590972	6.23E-06	0.000116
Methyl alpha-aspartylphenylalaninate-vs-Blautia	0.312461	0.027162	0.060319
Methyl alpha-aspartylphenylalaninate-vs-Streptococcus	0.519424	0.000111	0.000792
Methyl alpha-aspartylphenylalaninate-vs-Erysipelatoclostridium	0.488595	0.000318	0.001715
Methyl alpha-aspartylphenylalaninate-vs-Lachnospira	0.069868	0.62971	0.676244
Methyl alpha-aspartylphenylalaninate-vs-Fusobacterium	0.359712	0.010296	0.027993
Methyl alpha-aspartylphenylalaninate-vs-Bacillus	0.37623	0.007086	0.020522
Methyl alpha-aspartylphenylalaninate-vs-Dorea	0.275582	0.052737	0.102225

Methyl alpha-aspartylphenylalaninate-vs-Tyzzerella	0.241873	0.090595	0.155475
Methyl alpha-aspartylphenylalaninate-vs-Butyrivibrio	0.319088	0.023907	0.054553
Methyl alpha-aspartylphenylalaninate-vs-Coprobacillus	0.558992	2.45E-05	0.000277
Methyl alpha-aspartylphenylalaninate-vs-Selenomonas	0.375462	0.007213	0.020818
Methyl alpha-aspartylphenylalaninate-vs-Anaerostipes	0.349244	0.012923	0.033347
Methyl alpha-aspartylphenylalaninate-vs-Peptoclostridium	0.207779	0.147641	0.22155
Methyl alpha-aspartylphenylalaninate-vs-Dysgonomonas	0.45988	0.000779	0.003471
Methyl alpha-aspartylphenylalaninate-vs-Capnocytophaga	0.173205	0.22902	0.30813
Methyl alpha-aspartylphenylalaninate-vs-Flavobacterium	0.297959	0.035589	0.075653
Alphalin-vs-Alistipes	-0.14372	0.319376	0.396824
Alphalin-vs-Faecalibacterium	-0.52567	8.85E-05	0.000683
Alphalin-vs-Oscillibacter	-0.26982	0.058096	0.110206
Alphalin-vs-Subdoligranulum	-0.24562	0.085559	0.149203
Alphalin-vs-Bilophila	-0.35837	0.010605	0.028675
Alphalin-vs-Butyricicoccus	-0.41849	0.002491	0.008919
Alphalin-vs-Paraprevotella	-0.15813	0.272744	0.352821
Alphalin-vs-Collinsella	-0.39861	0.004142	0.013366
Alphalin-vs-Parasutterella	-0.27846	0.050212	0.09825
Alphalin-vs-Pyramidobacter	-0.10857	0.452941	0.519169
Alphalin-vs-Intestinimonas	-0.13748	0.341063	0.417714
Alphalin-vs-Pseudoflavonifractor	-0.16331	0.257122	0.336998
Alphalin-vs-Holdemania	-0.16062	0.265145	0.345147
Alphalin-vs-Oribacterium	-0.2023	0.158851	0.233992
Alphalin-vs-Eubacterium	0.297191	0.036089	0.076456
Alphalin-vs-Veillonella	0.627467	1.08E-06	5.56E-05

Alphalin-vs-Lactobacillus	0.563986	0.00002	0.000241
Alphalin-vs-Agathobacter	0.335222	0.017325	0.042044
Alphalin-vs-Enterococcus	0.595774	5.01E-06	0.0001
Alphalin-vs-Blautia	0.306699	0.030285	0.066132
Alphalin-vs-Streptococcus	0.553229	3.09E-05	0.000325
Alphalin-vs-Erysipelatoclostridium	0.502713	0.000199	0.001202
Alphalin-vs-Lachnospira	0.070156	0.628295	0.675308
Alphalin-vs-Fusobacterium	0.33551	0.017223	0.04192
Alphalin-vs-Bacillus	0.315054	0.025846	0.057968
Alphalin-vs-Dorea	0.220072	0.124617	0.196244
Alphalin-vs-Tyzzerella	0.229964	0.108147	0.177156
Alphalin-vs-Butyrivibrio	0.256567	0.072089	0.130666
Alphalin-vs-Coprobacillus	0.568884	1.63E-05	0.000212
Alphalin-vs-Selenomonas	0.325426	0.021107	0.049668
Alphalin-vs-Anaerostipes	0.323217	0.022049	0.051448
Alphalin-vs-Peptoclostridium	0.166194	0.248708	0.328745
Alphalin-vs-Dysgonomonas	0.497335	0.000238	0.001384
Alphalin-vs-Capnocytophaga	0.083505	0.564246	0.61796
Alphalin-vs-Flavobacterium	0.215654	0.132556	0.204725
Protirelin-vs-Alistipes	-0.11904	0.410291	0.478449
Protirelin-vs-Faecalibacterium	-0.45479	0.000906	0.003917
Protirelin-vs-Oscillibacter	-0.24293	0.089152	0.153742
Protirelin-vs-Subdoligranulum	-0.14382	0.319049	0.396815
Protirelin-vs-Bilophila	-0.19693	0.17046	0.246582
Protirelin-vs-Butyricicoccus	-0.38699	0.005499	0.016766

Protirelin-vs-Paraprevotella	-0.06401	0.658763	0.700182
Protirelin-vs-Collinsella	-0.40783	0.003284	0.011149
Protirelin-vs-Parasutterella	-0.25926	0.069053	0.126453
Protirelin-vs-Pyramidobacter	-0.04067	0.779141	0.806736
Protirelin-vs-Intestinimonas	-0.05297	0.714884	0.74831
Protirelin-vs-Pseudoflavonifractor	-0.08994	0.534503	0.591117
Protirelin-vs-Holdemania	-0.12259	0.396358	0.466356
Protirelin-vs-Oribacterium	-0.18636	0.195031	0.273352
Protirelin-vs-Eubacterium	0.170612	0.236173	0.315532
Protirelin-vs-Veillonella	0.673661	8.26E-08	0.000035
Protirelin-vs-Lactobacillus	0.566963	1.77E-05	0.00022
Protirelin-vs-Agathobacter	0.147275	0.30743	0.386425
Protirelin-vs-Enterococcus	0.523553	9.56E-05	0.000718
Protirelin-vs-Blautia	0.315918	0.02542	0.057218
Protirelin-vs-Streptococcus	0.510396	0.000153	0.000997
Protirelin-vs-Erysipelatoclostridium	0.49916	0.000224	0.001314
Protirelin-vs-Lachnospira	-0.0377	0.794945	0.820705
Protirelin-vs-Fusobacterium	0.471116	0.000554	0.002669
Protirelin-vs-Bacillus	0.405714	0.003465	0.0117
Protirelin-vs-Dorea	0.203745	0.155842	0.230379
Protirelin-vs-Tyzzerella	0.24994	0.080025	0.142655
Protirelin-vs-Butyrivibrio	0.238415	0.095445	0.16301
Protirelin-vs-Coprobacillus	0.570132	1.55E-05	0.000205
Protirelin-vs-Selenomonas	0.410612	0.003058	0.010536
Protirelin-vs-Anaerostipes	0.271741	0.056263	0.107632

Protirelin-vs-Peptoclostridium	0.127587	0.37725	0.450056
Protirelin-vs-Dysgonomonas	0.530564	7.39E-05	0.000596
Protirelin-vs-Capnocytophaga	0.05431	0.707964	0.742317
Protirelin-vs-Flavobacterium	0.129316	0.370766	0.444455
2-Oxo-3-(phosphonooxy)propanoic acid-vs-Alistipes	-0.23616	0.098718	0.166995
2-Oxo-3-(phosphonooxy)propanoic acid-vs-Faecalibacterium	-0.51347	0.000137	0.000923
2-Oxo-3-(phosphonooxy)propanoic acid-vs-Oscillibacter	-0.39548	0.004475	0.014142
2-Oxo-3-(phosphonooxy)propanoic acid-vs-Subdoligranulum	-0.28626	0.043864	0.08938
2-Oxo-3-(phosphonooxy)propanoic acid-vs-Bilophila	-0.33534	0.017284	0.042003
2-Oxo-3-(phosphonooxy)propanoic acid-vs-Butyricicoccus	-0.56597	1.84E-05	0.000227
2-Oxo-3-(phosphonooxy)propanoic acid-vs-Paraprevotella	-0.12689	0.37988	0.452976
2-Oxo-3-(phosphonooxy)propanoic acid-vs-Collinsella	-0.50003	0.000218	0.001289
2-Oxo-3-(phosphonooxy)propanoic acid-vs-Parasutterella	-0.19809	0.167897	0.243705
2-Oxo-3-(phosphonooxy)propanoic acid-vs-Pyramidobacter	-0.24126	0.091446	0.156612
2-Oxo-3-(phosphonooxy)propanoic acid-vs-Intestinimonas	-0.24348	0.088408	0.152778
2-Oxo-3-(phosphonooxy)propanoic acid-vs-Pseudoflavonifractor	-0.28729	0.043077	0.088395
2-Oxo-3-(phosphonooxy)propanoic acid-vs-Holdemania	-0.12629	0.382143	0.454801
2-Oxo-3-(phosphonooxy)propanoic acid-vs-Oribacterium	-0.1854	0.197385	0.276028
2-Oxo-3-(phosphonooxy)propanoic acid-vs-Eubacterium	0.228566	0.110368	0.179492
2-Oxo-3-(phosphonooxy)propanoic acid-vs-Veillonella	0.630062	9.45E-07	5.32E-05

2-Oxo-3-(phosphonooxy)propanoic acid-vs-Lactobacillus	0.562278	2.14E-05	0.000254
2-Oxo-3-(phosphonooxy)propanoic acid-vs-Agathobacter	0.279051	0.049709	0.097515
2-Oxo-3-(phosphonooxy)propanoic acid-vs-Enterococcus	0.552355	0.000032	0.000334
2-Oxo-3-(phosphonooxy)propanoic acid-vs-Blautia	0.344612	0.014257	0.036115
2-Oxo-3-(phosphonooxy)propanoic acid-vs-Streptococcus	0.579739	1.02E-05	0.000155
2-Oxo-3-(phosphonooxy)propanoic acid-vs-Erysipelatoclostridium	0.54704	3.95E-05	0.000386
2-Oxo-3-(phosphonooxy)propanoic acid-vs-Lachnospira	0.157095	0.275926	0.356108
2-Oxo-3-(phosphonooxy)propanoic acid-vs-Fusobacterium	0.434574	0.001613	0.006264
2-Oxo-3-(phosphonooxy)propanoic acid-vs-Bacillus	0.357518	0.010805	0.028973
2-Oxo-3-(phosphonooxy)propanoic acid-vs-Dorea	0.235345	0.099919	0.168573
2-Oxo-3-(phosphonooxy)propanoic acid-vs-Tyzzerella	0.209966	0.143329	0.217002
2-Oxo-3-(phosphonooxy)propanoic acid-vs-Butyrivibrio	0.299766	0.034433	0.073701
2-Oxo-3-(phosphonooxy)propanoic acid-vs-Coprobacillus	0.599369	4.24E-06	9.31E-05
2-Oxo-3-(phosphonooxy)propanoic acid-vs-Selenomonas	0.397157	0.004293	0.013766
2-Oxo-3-(phosphonooxy)propanoic acid-vs-Anaerostipes	0.37769	0.006849	0.019977
2-Oxo-3-(phosphonooxy)propanoic acid-vs-Peptoclostridium	0.182202	0.205366	0.284468
2-Oxo-3-(phosphonooxy)propanoic acid-vs-Dysgonomonas	0.453499	0.000941	0.004026
2-Oxo-3-(phosphonooxy)propanoic acid-vs-Capnocytophaga	0.19359	0.177965	0.254975
2-Oxo-3-(phosphonooxy)propanoic acid-vs-Flavobacterium	0.143864	0.318891	0.396815

2,4,5-Trimethoxybenzaldehyde-vs-Alistipes	-0.12375	0.391899	0.462426
2,4,5-Trimethoxybenzaldehyde-vs-Faecalibacterium	-0.41782	0.002535	0.009013
2,4,5-Trimethoxybenzaldehyde-vs-Oscillibacter	-0.20423	0.154849	0.229457
2,4,5-Trimethoxybenzaldehyde-vs-Subdoligranulum	-0.14132	0.327611	0.404428
2,4,5-Trimethoxybenzaldehyde-vs-Bilophila	-0.24418	0.087469	0.151577
2,4,5-Trimethoxybenzaldehyde-vs-Butyricicoccus	-0.43616	0.001544	0.006042
2,4,5-Trimethoxybenzaldehyde-vs-Paraprevotella	-0.09061	0.53144	0.588253
2,4,5-Trimethoxybenzaldehyde-vs-Collinsella	-0.37287	0.007657	0.021746
2,4,5-Trimethoxybenzaldehyde-vs-Parasutterella	-0.18944	0.187638	0.265535
2,4,5-Trimethoxybenzaldehyde-vs-Pyramidobacter	-0.03309	0.819574	0.842632
2,4,5-Trimethoxybenzaldehyde-vs-Intestinimonas	-0.04999	0.730287	0.762185
2,4,5-Trimethoxybenzaldehyde-vs-Pseudoflavonifractor	-0.08792	0.543742	0.599157
2,4,5-Trimethoxybenzaldehyde-vs-Holdemania	-0.07515	0.60398	0.654268
2,4,5-Trimethoxybenzaldehyde-vs-Oribacterium	-0.08043	0.578719	0.630753
2,4,5-Trimethoxybenzaldehyde-vs-Eubacterium	0.321008	0.023027	0.052984
2,4,5-Trimethoxybenzaldehyde-vs-Veillonella	0.598848	4.35E-06	9.31E-05
2,4,5-Trimethoxybenzaldehyde-vs-Lactobacillus	0.608067	2.82E-06	7.78E-05
2,4,5-Trimethoxybenzaldehyde-vs-Agathobacter	0.358271	0.010628	0.028675
2,4,5-Trimethoxybenzaldehyde-vs-Enterococcus	0.601441	3.85E-06	9.12E-05
2,4,5-Trimethoxybenzaldehyde-vs-Blautia	0.326291	0.020747	0.048962
2,4,5-Trimethoxybenzaldehyde-vs-Streptococcus	0.51443	0.000133	0.0009
2,4,5-Trimethoxybenzaldehyde-vs-Erysipelatoclostridium	0.534982	6.27E-05	0.000539
2,4,5-Trimethoxybenzaldehyde-vs-Lachnospira	0.029244	0.840232	0.859957
2,4,5-Trimethoxybenzaldehyde-vs-Fusobacterium	0.329556	0.019435	0.046173
2,4,5-Trimethoxybenzaldehyde-vs-Bacillus	0.39976	0.004025	0.01304

2,4,5-Trimethoxybenzaldehyde-vs-Dorea	0.281729	0.04747	0.094445
2,4,5-Trimethoxybenzaldehyde-vs-Tyzzerella	0.305834	0.030778	0.067033
2,4,5-Trimethoxybenzaldehyde-vs-Butyrivibrio	0.388715	0.005275	0.01624
2,4,5-Trimethoxybenzaldehyde-vs-Coprobacillus	0.603649	3.47E-06	8.88E-05
2,4,5-Trimethoxybenzaldehyde-vs-Selenomonas	0.418295	0.002503	0.008926
2,4,5-Trimethoxybenzaldehyde-vs-Anaerostipes	0.396591	0.004354	0.013906
2,4,5-Trimethoxybenzaldehyde-vs-Peptoclostridium	0.263866	0.064085	0.119649
2,4,5-Trimethoxybenzaldehyde-vs-Dysgonomonas	0.394958	0.004532	0.014256
2,4,5-Trimethoxybenzaldehyde-vs-Capnocytophaga	0.134022	0.353458	0.429298
2,4,5-Trimethoxybenzaldehyde-vs-Flavobacterium	0.234958	0.100494	0.169306
4-(Phosphonooxy)-L-threonine-vs-Alistipes	-0.21262	0.138218	0.211238
4-(Phosphonooxy)-L-threonine-vs-Faecalibacterium	-0.4715	0.000548	0.002652
4-(Phosphonooxy)-L-threonine-vs-Oscillibacter	-0.34526	0.014063	0.035732
4-(Phosphonooxy)-L-threonine-vs-Subdoligranulum	-0.18876	0.189237	0.267039
4-(Phosphonooxy)-L-threonine-vs-Bilophila	-0.32872	0.019763	0.046861
4-(Phosphonooxy)-L-threonine-vs-Butyricicoccus	-0.50903	0.00016	0.001017
4-(Phosphonooxy)-L-threonine-vs-Paraprevotella	-0.16398	0.255152	0.335124
4-(Phosphonooxy)-L-threonine-vs-Collinsella	-0.34277	0.01482	0.037312
4-(Phosphonooxy)-L-threonine-vs-Parasutterella	-0.23182	0.105253	0.17402
4-(Phosphonooxy)-L-threonine-vs-Pyramidobacter	-0.15959	0.268276	0.347798
4-(Phosphonooxy)-L-threonine-vs-Intestinimonas	-0.21051	0.142275	0.215581
4-(Phosphonooxy)-L-threonine-vs-Pseudoflavonifractor	-0.22732	0.112378	0.181807
4-(Phosphonooxy)-L-threonine-vs-Holdemania	-0.04973	0.731647	0.763285
4-(Phosphonooxy)-L-threonine-vs-Oribacterium	-0.13898	0.335755	0.412433
4-(Phosphonooxy)-L-threonine-vs-Eubacterium	0.285396	0.044536	0.090299

4-(Phosphonooxy)-L-threonine-vs-Veillonella	0.602406	3.68E-06	8.88E-05
4-(Phosphonooxy)-L-threonine-vs-Lactobacillus	0.536792	5.85E-05	0.000514
4-(Phosphonooxy)-L-threonine-vs-Agathobacter	0.311804	0.027503	0.061023
4-(Phosphonooxy)-L-threonine-vs-Enterococcus	0.598014	4.52E-06	9.45E-05
4-(Phosphonooxy)-L-threonine-vs-Blautia	0.354156	0.011626	0.030508
4-(Phosphonooxy)-L-threonine-vs-Streptococcus	0.577787	1.12E-05	0.000163
4-(Phosphonooxy)-L-threonine-vs-Erysipelatoclostridium	0.567321	1.74E-05	0.000219
4-(Phosphonooxy)-L-threonine-vs-Lachnospira	0.215226	0.133345	0.205687
4-(Phosphonooxy)-L-threonine-vs-Fusobacterium	0.489452	0.000309	0.001686
4-(Phosphonooxy)-L-threonine-vs-Bacillus	0.421397	0.002306	0.008465
4-(Phosphonooxy)-L-threonine-vs-Dorea	0.299441	0.034639	0.07395
4-(Phosphonooxy)-L-threonine-vs-Tyzzerella	0.258011	0.070445	0.128435
4-(Phosphonooxy)-L-threonine-vs-Butyrivibrio	0.332736	0.018226	0.043675
4-(Phosphonooxy)-L-threonine-vs-Coprobacillus	0.636352	6.8E-07	4.83E-05
4-(Phosphonooxy)-L-threonine-vs-Selenomonas	0.467761	0.000614	0.00288
4-(Phosphonooxy)-L-threonine-vs-Anaerostipes	0.350143	0.012677	0.032814
4-(Phosphonooxy)-L-threonine-vs-Peptoclostridium	0.195542	0.173546	0.250152
4-(Phosphonooxy)-L-threonine-vs-Dysgonomonas	0.492543	0.00028	0.001572
4-(Phosphonooxy)-L-threonine-vs-Capnocytophaga	0.177647	0.217118	0.297025
4-(Phosphonooxy)-L-threonine-vs-Flavobacterium	0.227915	0.111413	0.180601
2-Amino-6-[(3-carboxypropanoyl)amino]heptanedioic acid-vs-Alistipes	-0.12432	0.389681	0.461559
2-Amino-6-[(3-carboxypropanoyl)amino]heptanedioic acid-vs-Faecalibacterium	-0.52211	0.000101	0.000745

2-Amino-6-[(3-carboxypropanoyl)amino]heptanedioic acid-vs-Oscillibacter	-0.18876	0.189238	0.267039
2-Amino-6-[(3-carboxypropanoyl)amino]heptanedioic acid-vs-Subdoligranulum	-0.15467	0.2835	0.362396
2-Amino-6-[(3-carboxypropanoyl)amino]heptanedioic acid-vs-Bilophila	-0.12855	0.37364	0.447252
2-Amino-6-[(3-carboxypropanoyl)amino]heptanedioic acid-vs-Butyricicoccus	-0.32341	0.021966	0.051399
2-Amino-6-[(3-carboxypropanoyl)amino]heptanedioic acid-vs-Paraprevotella	-0.09589	0.50768	0.56777
2-Amino-6-[(3-carboxypropanoyl)amino]heptanedioic acid-vs-Collinsella	-0.32245	0.022385	0.052037
2-Amino-6-[(3-carboxypropanoyl)amino]heptanedioic acid-vs-Parasutterella	-0.29248	0.039285	0.081694
2-Amino-6-[(3-carboxypropanoyl)amino]heptanedioic acid-vs-Pyramidobacter	-0.00562	0.969112	0.973027
2-Amino-6-[(3-carboxypropanoyl)amino]heptanedioic acid-vs-Intestinimonas	0.021753	0.88081	0.896687
2-Amino-6-[(3-carboxypropanoyl)amino]heptanedioic acid-vs-Pseudoflavonifractor	-0.05834	0.687347	0.724674
2-Amino-6-[(3-carboxypropanoyl)amino]heptanedioic acid-vs-Holdemania	-0.0981	0.497907	0.559357
2-Amino-6-[(3-carboxypropanoyl)amino]heptanedioic acid-vs-Oribacterium	-0.1709	0.235371	0.31463

2-Amino-6-[(3-carboxypropanoyl)amino]heptanedioic acid-vs-Eubacterium	0.180312	0.210187	0.289371
2-Amino-6-[(3-carboxypropanoyl)amino]heptanedioic acid-vs-Veillonella	0.627467	1.08E-06	5.56E-05
2-Amino-6-[(3-carboxypropanoyl)amino]heptanedioic acid-vs-Lactobacillus	0.519232	0.000112	0.000794
2-Amino-6-[(3-carboxypropanoyl)amino]heptanedioic acid-vs-Agathobacter	0.072077	0.618895	0.666358
2-Amino-6-[(3-carboxypropanoyl)amino]heptanedioic acid-vs-Enterococcus	0.545546	4.18E-05	0.000404
2-Amino-6-[(3-carboxypropanoyl)amino]heptanedioic acid-vs-Blautia	0.281825	0.047391	0.09444
2-Amino-6-[(3-carboxypropanoyl)amino]heptanedioic acid-vs-Streptococcus	0.471116	0.000554	0.002669
2-Amino-6-[(3-carboxypropanoyl)amino]heptanedioic acid-vs-Erysipelatoclostridium	0.470444	0.000566	0.002709
2-Amino-6-[(3-carboxypropanoyl)amino]heptanedioic acid-vs-Lachnospira	-0.03673	0.80006	0.825644
2-Amino-6-[(3-carboxypropanoyl)amino]heptanedioic acid-vs-Fusobacterium	0.503385	0.000194	0.001184
2-Amino-6-[(3-carboxypropanoyl)amino]heptanedioic acid-vs-Bacillus	0.447875	0.001108	0.004645
2-Amino-6-[(3-carboxypropanoyl)amino]heptanedioic acid-vs-Dorea	0.172725	0.230334	0.309394

2-Amino-6-[(3-carboxypropanoyl)amino]heptanedioic acid-vs-Tyzzerella	0.220648	0.123608	0.195523
2-Amino-6-[(3-carboxypropanoyl)amino]heptanedioic acid-vs-Butyrivibrio	0.244178	0.087469	0.151577
2-Amino-6-[(3-carboxypropanoyl)amino]heptanedioic acid-vs-Coprobacillus	0.532869	6.78E-05	0.000569
2-Amino-6-[(3-carboxypropanoyl)amino]heptanedioic acid-vs-Selenomonas	0.438848	0.001432	0.005713
2-Amino-6-[(3-carboxypropanoyl)amino]heptanedioic acid-vs-Anaerostipes	0.197695	0.168765	0.244537
2-Amino-6-[(3-carboxypropanoyl)amino]heptanedioic acid-vs-Peptoclostridium	0.13383	0.354155	0.429934
2-Amino-6-[(3-carboxypropanoyl)amino]heptanedioic acid-vs-Dysgonomonas	0.569652	1.58E-05	0.000207
2-Amino-6-[(3-carboxypropanoyl)amino]heptanedioic acid-vs-Capnocytophaga	0.074574	0.606765	0.656426
2-Amino-6-[(3-carboxypropanoyl)amino]heptanedioic acid-vs-Flavobacterium	0.22084	0.123273	0.195242
3,4-dihydroxyphenylacetic acid-vs-Alistipes	-0.18992	0.186501	0.264076
3,4-dihydroxyphenylacetic acid-vs-Faecalibacterium	-0.43914	0.001421	0.005686
3,4-dihydroxyphenylacetic acid-vs-Oscillibacter	-0.30872	0.02916	0.064182
3,4-dihydroxyphenylacetic acid-vs-Subdoligranulum	-0.20365	0.156041	0.230537
3,4-dihydroxyphenylacetic acid-vs-Bilophila	-0.35875	0.010516	0.028467
3,4-dihydroxyphenylacetic acid-vs-Butyricicoccus	-0.38862	0.005287	0.01624
3,4-dihydroxyphenylacetic acid-vs-Paraprevotella	-0.14286	0.322325	0.39949

3,4-dihydroxyphenylacetic acid-vs-Collinsella	-0.40571	0.003465	0.0117
3,4-dihydroxyphenylacetic acid-vs-Parasutterella	-0.17474	0.224853	0.304004
3,4-dihydroxyphenylacetic acid-vs-Pyramidobacter	-0.06363	0.660686	0.701327
3,4-dihydroxyphenylacetic acid-vs-Intestinimonas	-0.13258	0.358703	0.433972
3,4-dihydroxyphenylacetic acid-vs-Pseudoflavonifractor	-0.17215	0.231916	0.310969
3,4-dihydroxyphenylacetic acid-vs-Holdemania	-0.2001	0.163547	0.239348
3,4-dihydroxyphenylacetic acid-vs-Oribacterium	-0.17964	0.211921	0.291274
3,4-dihydroxyphenylacetic acid-vs-Eubacterium	0.287107	0.043219	0.088541
3,4-dihydroxyphenylacetic acid-vs-Veillonella	0.64437	4.43E-07	4.08E-05
3,4-dihydroxyphenylacetic acid-vs-Lactobacillus	0.647923	3.65E-07	4.07E-05
3,4-dihydroxyphenylacetic acid-vs-Agathobacter	0.325042	0.021268	0.050001
3,4-dihydroxyphenylacetic acid-vs-Enterococcus	0.547467	3.88E-05	0.000381
3,4-dihydroxyphenylacetic acid-vs-Blautia	0.281345	0.047786	0.094846
3,4-dihydroxyphenylacetic acid-vs-Streptococcus	0.559088	2.44E-05	0.000277
3,4-dihydroxyphenylacetic acid-vs-Erysipelatoclostridium	0.49964	0.000221	0.001299
3,4-dihydroxyphenylacetic acid-vs-Lachnospira	0.103289	0.475348	0.538395
3,4-dihydroxyphenylacetic acid-vs-Fusobacterium	0.335798	0.017122	0.041796
3,4-dihydroxyphenylacetic acid-vs-Bacillus	0.292005	0.039624	0.082261
3,4-dihydroxyphenylacetic acid-vs-Dorea	0.209412	0.144414	0.218157
3,4-dihydroxyphenylacetic acid-vs-Tyzzerella	0.216134	0.131675	0.203998
3,4-dihydroxyphenylacetic acid-vs-Butyrivibrio	0.276831	0.051631	0.100393
3,4-dihydroxyphenylacetic acid-vs-Coprobacillus	0.531813	7.05E-05	0.000581
3,4-dihydroxyphenylacetic acid-vs-Selenomonas	0.316879	0.024953	0.056423
3,4-dihydroxyphenylacetic acid-vs-Anaerostipes	0.341369	0.01526	0.038111
3,4-dihydroxyphenylacetic acid-vs-Peptoclostridium	0.12509	0.386734	0.458509

3,4-dihydroxyphenylacetic acid-vs-Dysgonomonas	0.471693	0.000544	0.002642
3,4-dihydroxyphenylacetic acid-vs-Capnocytophaga	0.115102	0.426056	0.49428
3,4-dihydroxyphenylacetic acid-vs-Flavobacterium	0.124034	0.390789	0.461772
(4Z,7Z,10Z,13Z,16Z,19Z)-4,7,10,13,1 6,19-Docosahexaenoic acid-vs-Alistipes	-0.03501	0.809289	0.833437
(4Z,7Z,10Z,13Z,16Z,19Z)-4,7,10,13,1 6,19-Docosahexaenoic acid-vs-Faecalibacterium	-0.50819	0.000165	0.001042
(4Z,7Z,10Z,13Z,16Z,19Z)-4,7,10,13,1 6,19-Docosahexaenoic acid-vs-Oscillibacter	-0.14209	0.324961	0.401956
(4Z,7Z,10Z,13Z,16Z,19Z)-4,7,10,13,1 6,19-Docosahexaenoic acid-vs-Subdoligranulum	-0.14987	0.298896	0.378185
(4Z,7Z,10Z,13Z,16Z,19Z)-4,7,10,13,1 6,19-Docosahexaenoic acid-vs-Bilophila	-0.15438	0.284409	0.363371
(4Z,7Z,10Z,13Z,16Z,19Z)-4,7,10,13,1 6,19-Docosahexaenoic acid-vs-Butyricicoccus	-0.37508	0.007277	0.020979
(4Z,7Z,10Z,13Z,16Z,19Z)-4,7,10,13,1 6,19-Docosahexaenoic acid-vs-Paraprevotella	-0.07208	0.618895	0.666358
(4Z,7Z,10Z,13Z,16Z,19Z)-4,7,10,13,1 6,19-Docosahexaenoic acid-vs-Collinsella	-0.31006	0.028429	0.062852
(4Z,7Z,10Z,13Z,16Z,19Z)-4,7,10,13,1 6,19-Docosahexaenoic acid-vs-Parasutterella	-0.31486	0.025942	0.05813
(4Z,7Z,10Z,13Z,16Z,19Z)-4,7,10,13,1 6,19-Docosahexaenoic acid-vs-Pyramidobacter	-0.02348	0.871415	0.888944
(4Z,7Z,10Z,13Z,16Z,19Z)-4,7,10,13,1 6,19-Docosahexaenoic acid-vs-Intestinimonas	-0.00168	0.990758	0.991556

(4Z,7Z,10Z,13Z,16Z,19Z)-4,7,10,13,1 6,19-Docosahexaenoic acid-vs-Pseudoflavonifractor	-0.04922	0.734279	0.764746
(4Z,7Z,10Z,13Z,16Z,19Z)-4,7,10,13,1 6,19-Docosahexaenoic acid-vs-Holdemania	-0.06852	0.636331	0.681294
(4Z,7Z,10Z,13Z,16Z,19Z)-4,7,10,13,1 6,19-Docosahexaenoic acid-vs-Oribacterium	-0.1491	0.301408	0.380396
(4Z,7Z,10Z,13Z,16Z,19Z)-4,7,10,13,1 6,19-Docosahexaenoic acid-vs-Eubacterium	0.215942	0.132027	0.204289
(4Z,7Z,10Z,13Z,16Z,19Z)-4,7,10,13,1 6,19-Docosahexaenoic acid-vs-Veillonella	0.624874	1.23E-06	5.93E-05
(4Z,7Z,10Z,13Z,16Z,19Z)-4,7,10,13,1 6,19-Docosahexaenoic acid-vs-Lactobacillus	0.556591	0.000027	0.000294
(4Z,7Z,10Z,13Z,16Z,19Z)-4,7,10,13,1 6,19-Docosahexaenoic acid-vs-Agathobacter	0.156591	0.277491	0.356917
(4Z,7Z,10Z,13Z,16Z,19Z)-4,7,10,13,1 6,19-Docosahexaenoic acid-vs-Enterococcus	0.54497	4.28E-05	0.000409
(4Z,7Z,10Z,13Z,16Z,19Z)-4,7,10,13,1 6,19-Docosahexaenoic acid-vs-Blautia	0.287107	0.043219	0.088541
(4Z,7Z,10Z,13Z,16Z,19Z)-4,7,10,13,1 6,19-Docosahexaenoic acid-vs-Streptococcus	0.492053	0.000284	0.00158
(4Z,7Z,10Z,13Z,16Z,19Z)-4,7,10,13,1 6,19-Docosahexaenoic acid-vs-Erysipelatoclostridium	0.452773	0.000962	0.004085
(4Z,7Z,10Z,13Z,16Z,19Z)-4,7,10,13,1 6,19-Docosahexaenoic acid-vs-Lachnospira	0.003794	0.979141	0.981907

(4Z,7Z,10Z,13Z,16Z,19Z)-4,7,10,13,1 6,19-Docosahexaenoic acid-vs-Fusobacterium	0.506939	0.000172	0.001074
(4Z,7Z,10Z,13Z,16Z,19Z)-4,7,10,13,1 6,19-Docosahexaenoic acid-vs-Bacillus	0.442209	0.001303	0.005308
(4Z,7Z,10Z,13Z,16Z,19Z)-4,7,10,13,1 6,19-Docosahexaenoic acid-vs-Dorea	0.212197	0.13903	0.212087
(4Z,7Z,10Z,13Z,16Z,19Z)-4,7,10,13,1 6,19-Docosahexaenoic acid-vs-Tyzzerella	0.220072	0.124617	0.196244
(4Z,7Z,10Z,13Z,16Z,19Z)-4,7,10,13,1 6,19-Docosahexaenoic acid-vs-Butyrivibrio	0.281248	0.047865	0.094928
(4Z,7Z,10Z,13Z,16Z,19Z)-4,7,10,13,1 6,19-Docosahexaenoic acid-vs-Coprobacillus	0.533157	6.71E-05	0.000565
(4Z,7Z,10Z,13Z,16Z,19Z)-4,7,10,13,1 6,19-Docosahexaenoic acid-vs-Selenomonas	0.420792	0.002344	0.008564
(4Z,7Z,10Z,13Z,16Z,19Z)-4,7,10,13,1 6,19-Docosahexaenoic acid-vs-Anaerostipes	0.204994	0.153269	0.227795
(4Z,7Z,10Z,13Z,16Z,19Z)-4,7,10,13,1 6,19-Docosahexaenoic acid-vs-Peptoclostridium	0.125186	0.386367	0.458292
(4Z,7Z,10Z,13Z,16Z,19Z)-4,7,10,13,1 6,19-Docosahexaenoic acid-vs-Dysgonomonas	0.599904	4.14E-06	9.26E-05
(4Z,7Z,10Z,13Z,16Z,19Z)-4,7,10,13,1 6,19-Docosahexaenoic acid-vs-Capnocytophaga	0.09916	0.493267	0.555149
(4Z,7Z,10Z,13Z,16Z,19Z)-4,7,10,13,1 6,19-Docosahexaenoic acid-vs-Flavobacterium	0.250324	0.079547	0.141905

Supplementary Table S7. Spearman's correlation coefficients between plasma metabolites and microbial genera. Spearman's correlations coefficients between marker CAGs and p values were calculated and p values were corrected for multiple testing as q values with Benjamin & Hochberg method. SCC = Spearman's correlation coefficient.
